# Iron-catalyzed cyanomethylation of 4*H*-pyrido[1,2-*a*]pyrimidin-4-ones and quinolin-4(1*H*)-ones with alkyl nitriles

**DOI:** 10.1039/d6ra04026h

**Published:** 2026-07-06

**Authors:** Amol B. Gadekar, Tarun Jangir, Krishnan Rangan, Anil Kumar

**Affiliations:** a Department of Chemistry, Birla Institute of Technology and Science Pilani, Pilani Campus, Vidya Vihar Pilani Rajasthan 333031 India anilkumar@pilani.bits-pilani.ac.in; b Department of Chemistry, Birla Institute of Technology and Science Pilani, Hyderabad Campus, Jawahar Nagar, Kapra Mandal, Medchal District Secunderabad Telangana 500078 India

## Abstract

An iron(iii)-catalyzed C3-selective cyanomethylation of 4*H*-pyrido[1,2-*a*]pyrimidin-4-ones and quinolin-4(1*H*)-ones has been developed using alkyl nitriles as a cyanomethyl source. This protocol exhibits broad substrate scope and good functional-group tolerance, affording the corresponding C3-(hetero)arylacetonitriles in good to high yields. The reaction proceeds under mild oxidative conditions with di-*tert*-butyl peroxide (DTBP) and involves the generation of a cyanomethyl radical, enabling direct C–H functionalization without substrate pre-activation.

## Abstract 2

An iron(iii)-catalyzed C3-selective cyanomethylation of 4*H*-pyrido[1,2-*a*]pyrimidin-4-ones and quinolin-4(1*H*)-ones has been developed using alkyl nitriles as a cyanomethyl source.

## Graphical abstract

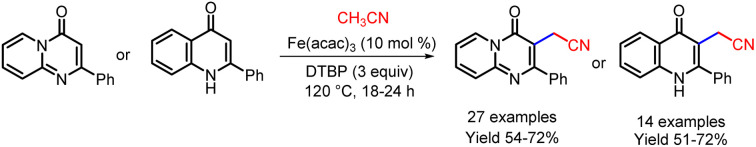

## Introduction

**Scheme 1:**
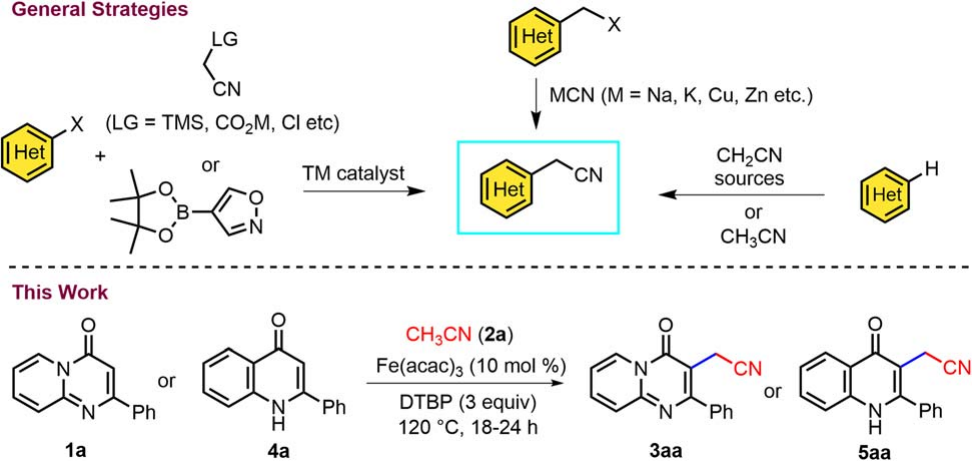
Strategies for cyanomethylation of (hetero)arenes and this work.

Cyanomethyl (–CH_2_CN) group containing compounds exhibit excellent biological activity and are highly versatile building blocks in medicinal chemistry.^1–3^ 3-Cyanomethylimidazo[1,2-*a*]pyridine is an important intermediate in the synthesis of the insomnia drug Zolpidem.^4^ Many heterocyclic compounds can be easily achieved from cyanomethyl pyridinium and isoquinolinium salts.^5^ 2-Cyanomethyl indanes and 2-(cyanomethyl)benzimidazoles have been used as intermediates for the synthesis of compounds to treat bacterial infection.^6,7^ (Hetero)arylacetonitriles are useful synthetic intermediates to construct various more complex fused heterocyclic systems.^8^ They can be easily transformed to various functional groups such as α-(hetero)aryl amides, α-(hetero)aryl carboxylic acids and β-(hetero)aryl amines.

(Hetero)arylacetonitriles are traditionally prepared by cyanation of a benzylic halide with MCN or dehydration of amides or arylacetaldoximes (Scheme 1).^9,10^ These methods suffer with several limitations including reagent toxicity, substrate scope and harsh reaction conditions. To overcome these limitations, transition metal-catalyzed cyanomethylation of (hetero)aryl halides or pseudo (hetero)aryl halides with an acetonitrile anion equivalent was developed as an alternative route (Scheme 1).^11–13^ However, requirement of pre-functionalized substrate, reagent accessibility, limited substrate scope, use of excess oxidants, and high reaction temperatures hamper synthetic utility of this approach.

In recent years, radical-triggered cross-dehydrogenative coupling (CDC) of alkyl nitriles has become valuable method for the synthesis of functionalized nitriles (Scheme 1) as these reactions have the advantages of operation simplicity, high efficiency, atom- and step-economy and mild reaction conditions.^14–17^ Rao and Xu group developed a Fe(ii)-catalyzed dehydrogenative C(sp^2^)–H/C(sp^3^)–H coupling of imidazo[1,2-*a*]pyridines with acetonitrile using DCP oxidant to synthesise corresponding heteroaryl-acetonitriles.^18^ Guo group reported synthesis of 2-cyanomethylindoles and 2-cyanomethylpyrroles by Fe(ii)-catalyzed coupling of indoles and pyrroles with acetonitrile.^19^ Later, Yan group developed a FeCl_2_-catalyzed amine-directed cyanomethylation of aminopyridines and anilines with acetonitrile by using DTBP as oxidant.^20^ These methods leverages Fe(ii)'s ability to facilitate single-electron transfer (SET), generating cyanomethyl radicals from alkyl nitriles. Kozlowski group described a *tert*-butyl hydroperoxide (TBHP)-promoted cyanomethylation of fluorenes and oxindoles with alkyl nitriles to generate corresponding cyanomethylated products in high (70–95%) yields.^21^ Foroumadi^22^ and Tan^23^ groups independently developed a cross-dehydrogenative coupling (CDC) reaction of 8-aminoquinoline amides with alkyl nitriles for the synthesis of C-5 cyanomethylated quinolines using di-*tert*-butyl peroxide (DTBP) and *tert*-butyl peroxybenzoate (TBPB) as the oxidant, respectively. Liu group described a CuI-catalyzed site-specific CDC reaction of electron-rich heterocycles (indoles, pyrroles, furans, thiophenes) with alkyl nitriles using dicumylperoxide (DCP) as oxidant.^24^ Zhang *et al.* reported a TBPB-mediated CDC reaction between coumarins and acetonitriles to prepare 3-cyanomethylcoumarins.^25^ Despite this progress, several challenges persist in expanding these methods to other heterocycles. Consequently, it is highly desirable to develop more efficient and simple methods to access (hetero)arylacetonitriles. With our continuous interest in C–H bond functionalization of (hetero)arenes,^26–28^ we herein report a Fe(acac)_3_-catalyzed site-selective C3-cyanomethylation of 4*H*-pyrido[1,2-*a*]pyrimidin-4-ones and quinolin-4(1*H*)-ones with alkyl nitriles using DTBP as oxidant (Scheme 1).

## Results and discussion

We initiated our investigation using 2-phenyl-4*H*-pyrido[1,2-*a*]pyrimidin-4-one (1a) and acetonitrile (2a) as the model substrates. Initially, reaction of 1a with 2a in the presence of DTBP (3 equiv.) at 120 °C for 24 h generated 2-(4-oxo-2-phenyl-4*H*-pyrido[1,2-*a*]pyrimidin-3-yl)acetonitrile (3aa) in 15% yield (Table 1, entry 1). Interestingly, yield of 3aa increased significantly on using Fe(acac)_3_ (10 mol%) as a catalyst (Table 1, entry 2). To improve the reaction efficiency, different metal catalysts including FeCl_3_, Fe(NO_3_)_3_, FeCl_2_, CuI and Mn(OAc)_2_ were tested and the results showed that none of them could match the efficiency of Fe(acac)_3_ (Table 1, entries 3–7). Subsequently, we screened different oxidants such as DCP, TBHP, DDQ, TBPB and K_2_S_2_O_8_ with Fe(acac)_3_ (10 mol%) as a catalyst (Table 1, entries 8–12). Only TBPB gave the product 3aa in 48% yield, while desired product was not obtained using DCP, TBHP, DDQ and K_2_S_2_O_8_.

**Table 1:** Optimization of the reaction conditions for 3aa^a^

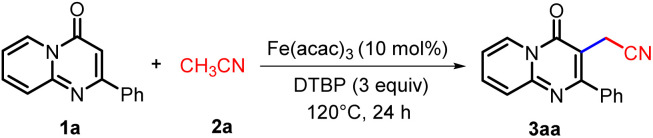		
Entry	Deviation from standard conditions	% Yield^b^ of 3aa
1	No catalyst	15
2	None	70
3	FeCl_3_ used as catalyst	38
4	Fe(NO_3_)_3_ used as catalyst	26
5	FeCl_2_ used as catalyst	39
6	CuI used as catalyst	12
7	Mn(OAc)_2_ used as catalyst	Traces
8	DCP used as oxidant	Traces
9	TBHP used as oxidant	—
10	DDQ used as oxidant	—
11	K_2_S_2_O_8_ used as oxidant	—
12	TBPB used as oxidant	48
13	Reaction temp. 100 °C	50
14	Reaction temp. 130 °C	68
15	5 mol% of Fe(acac)_3_	54
16	2 equiv. of DTBP	59
17	Open flask instead of sealed tube	38
18	No DTBP	—

a Reaction conditions: 1a (1 equiv., 0.22 mmol), 2a (2 mL), oxidant (3 equiv.), catalyst (10 mol%), sealed tube, heated in an oil bath 120 °C for 24 h.

b Isolated yield.

Next, we examined model reaction at different temperatures and the results showed that 120 °C was the most suitable reaction temperature (Table 1, entries 13 and 14). It was found decreasing catalyst loading (Table 1, entry 15) or the amount of oxidant (Table 1, entry 16) resulted decrease in the yield of 3aa. Further, product 3aa was obtained in 38% yield when the reaction was conducted in an open flask at reflux set-up (Table 1, entry 17). Finally, control experiments showed that 3aa could not be formed in the absence of the DTBP (Table 1, entry 17).

After having established the optimal reaction conditions, the generality of the developed protocol was then explored (Table 2). Reaction of 2-aryl-4*H*-pyrido[1,2-*a*]pyrimidin-4-ones bearing various substituents on the *para*-, *meta*- or *ortho*-position of C2-aryl ring 1a–j with 2a gave desired products 3aa–3ja in moderate to good (54–66%) yields. Similarly, 2-phenyl-4*H*-pyrido[1,2-*a*]pyrimidin-4-ones with substituent at the C7- and C8-position of the pyrimidine ring 1k–1m reacted smoothly with 2a to produce the desired products 3ka–3ma in 57–70% yield. Next, 2-aryl-4*H*-pyrido[1,2-*a*]pyrimidin-4-ones having substituent on both the C2-aryl ring and pyrimidine ring 1n–x took part in reaction with 2a to afford the desired C3-cyanomethylated products 3na–xa in 56–73% yields. Notably, the reaction of 4*H*-pyrido[1,2-*a*]pyrimidin-4-ones having 2-(furan-2-yl) 1y, 2-(thiophen-2-yl) 1z–1α and 2-methyl 1β with 2a under optimized conditions produced the corresponding C3-cyanomethylated products 3ya–3βa in low to good (37–64%) yields. Further, 7-phenyl-5*H*-thiazolo[3,2-*a*]pyrimidin-5-one (1γ) also participated in the reaction, resulting in the corresponding C3-cyanomethylated product 3γa in 72% yield. Finally, reaction of 1a with propionitrile (2b) and butyronitrile (2c) under standard conditions produced corresponding cyanomethylated products 3ab and 3ac in 68% and 66% yields, respectively, highlighting that other alkyl nitriles can also be used as suitable substrates for this reaction. The structure of 3ma was determined by single crystal X-ray diffraction analysis (CCDC 2543349).

**Table 2:** Substrate scope for 2-aryl-4*H*-pyrido[1,2-*a*]pyrimidin-4-ones^a^^,^^b^

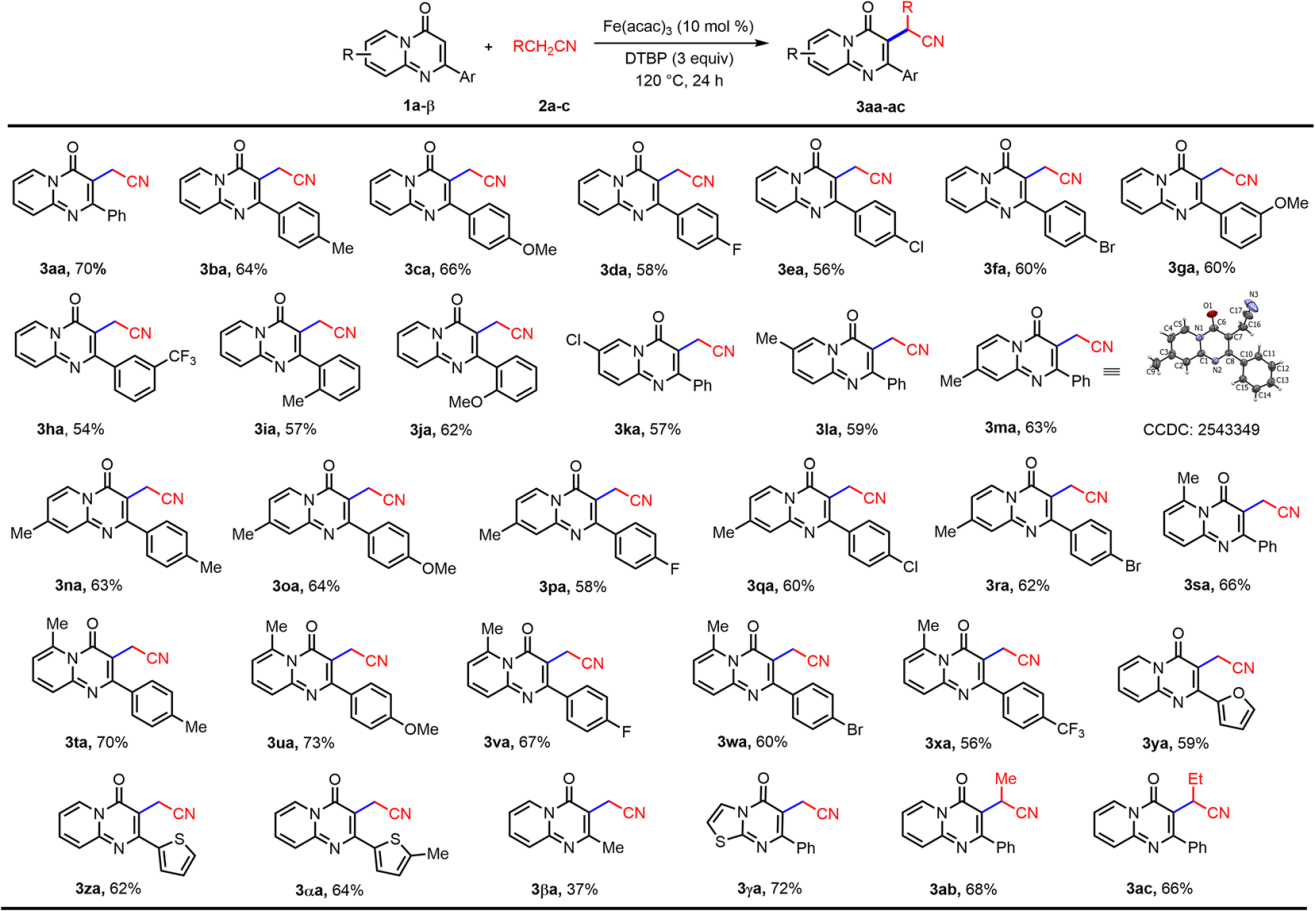

a Reaction condition: 1 (1 equiv., 0.22 mmol), 2 (2 mL), DTBP (3 equiv.), Fe(acac)_3_ (10 mol%), sealed tube, heated in an oil bath at 120 °C for 24 h.

b Isolated yield.

Furthermore, we were glad to find that the developed protocol was not restricted to 4*H*-pyrido[1,2-*a*]pyrimidin-4-ones but biologically important quinolin-4(1*H*)-ones (4) were also suitable substrates for this reaction (Table 3). Quinolin-4(1*H*)-ones bearing electron-donating (*e.g.* H, Me, OMe; 4a–c), halogens (F, Cl, Br, I; 4d–g) and electron-withdrawing (NO_2_; 4h) groups at the *para*-position of the C2-aryl ring smoothly reacted to form the desired products 5aa–5ha in 58–72% yields. Remarkably, quinolin-4(1*H*)-ones with substituent at *ortho*-position (Me, 4i) and *meta*-position (Cl, 4j–k) of the C2-aryl ring also afforded C3-cyanomethylated product 5ia–ka in 60–68% yields. Gratifyingly, 2-(5-methylthiophen-2-yl)quinolin-4(1*H*)-one (4l) is also amenable to this reaction and afforded desired C3-cyanomethylated product 5la in 67% yield. Furthermore, 2-phenylquinolin-4(1*H*)-ones with a substituent on quinolinone scaffold 4m–n and *N*-methyl-2-phenylquinolin-4(1*H*)-one (4o) also participated in the reaction, furnishing the C3-cyanomethylation products 5ma–oa in 51–71% yields. Interestingly, reaction of propionitrile (2b) and butyronitrile (2c) with 4a under standard conditions produced desired products 5ab and 5ac in 61% and 66% yields, respectively. The structure of 5ba was determined by single crystal X-ray diffraction analysis (CCDC 2543354).

**Table 3:** Substrate scope for 2-arylquinolin-4(1*H*)-one^a^^,^^b^

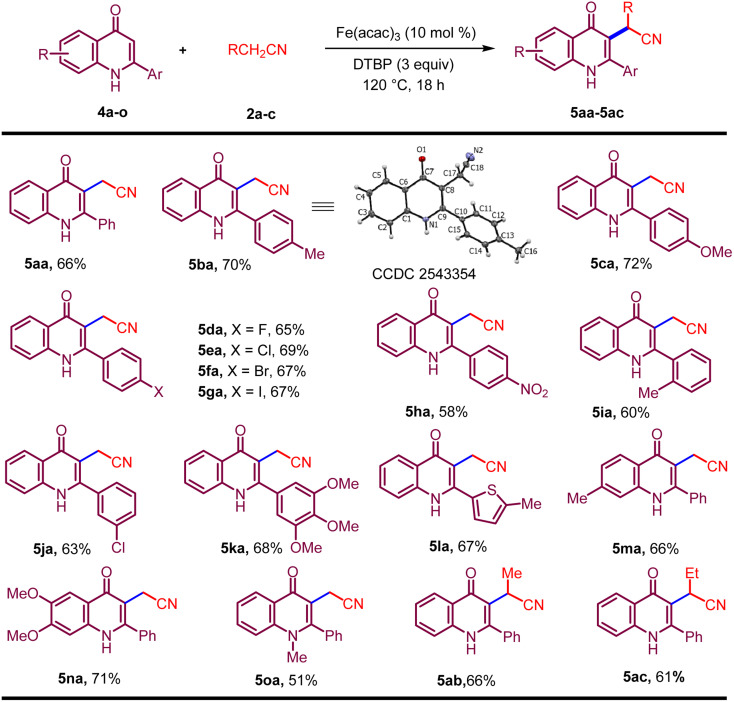

a Reaction condition: 1 (1 equiv., 0.22 mmol), 2 (2 mL), DTBP (3 equiv.), Fe(acac)_3_ (10 mol%), sealed tube, heated in an oil bath at 120 °C for 18 h.

b Isolated yield.

**Scheme 2:**
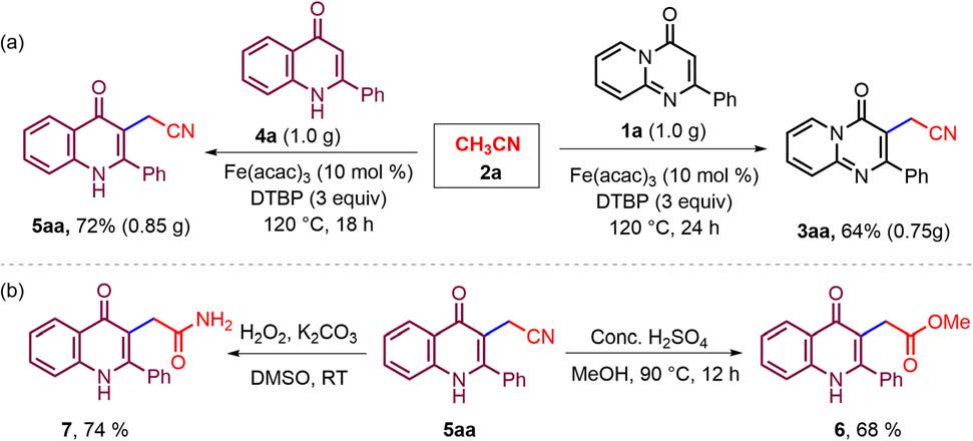
(a) Gram-scale synthesis of 3aa and 5aa and (b) synthetic transformation of 5aa.

The developed method can be scaled up, as demonstrated by the gram scale synthesis of products 3aa and 5aa in 64% (0.75 g) and 72% (0.85 g) yields, respectively, without further optimization (Scheme 2a). Further, synthetic utility of the products was demonstrated by successful transformation of 5aa to corresponding amide 7 (74%) by reaction with H_2_O_2_/K_2_CO_3_ in DMSO^29^ and ester 6 (68%) by reaction with H_2_SO_4_ in methanol^4^ (Scheme 2b).

**Scheme 3:**
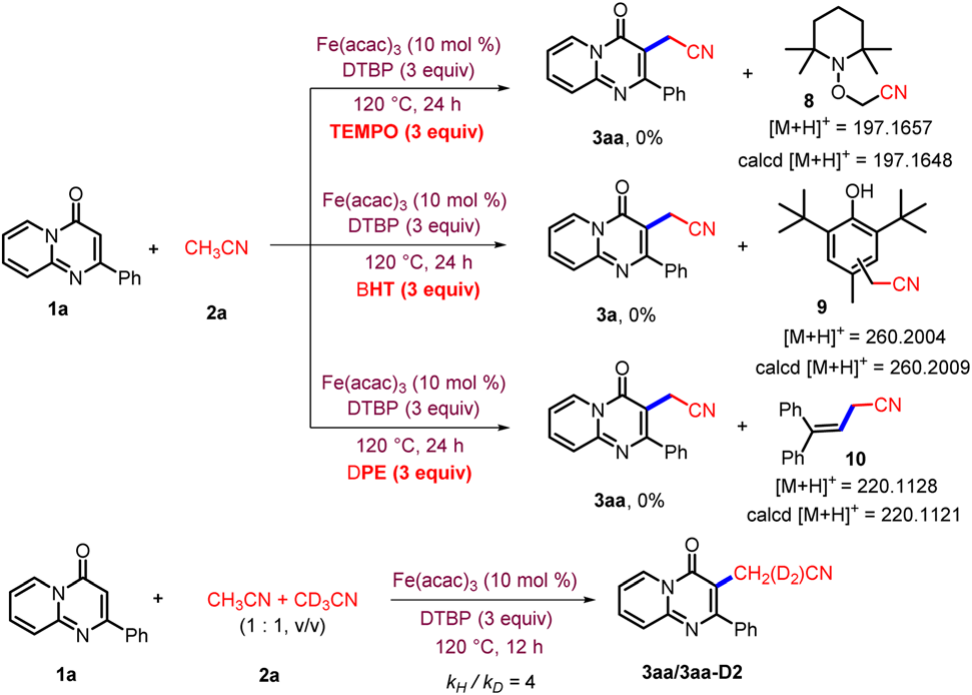
Control experiments.

To gain insight into the reaction mechanism, a few control experiments were performed (Scheme 3). Initially, effect of the radical scavengers, TEMPO, BHT, or 1,1-diphenylethylene (DPE), was examined on the reaction of 1a with 2a under standard reaction conditions. Product formation completely quenched in the presence of these scavengers, suggesting that a radical pathway is likely involved. We could not isolate the trapped adducts, but HRMS analysis of the reaction mixture revealed the formation of the TEMPO–CH_2_CN adduct (8, calcd *m*/*z* = 197.1648, found *m*/*z* = 197.1657), BHT–CH_2_CN adduct (9, calcd *m*/*z* = 260.2009, found *m*/*z* = 260.2004) and DPE–CH_2_CN adduct (10, calcd *m*/*z* = 220.1121, found *m*/*z* = 220.1128). Detection of these adducts suggested that the cyanomethyl radical could be a possible intermediate in this transformation. Finally, an intermolecular competitive kinetic isotope effect (KIE) experiment was conducted using CH_3_CN and CD_3_CN (Scheme 3), which revealed a *k*_H_/*k*_D_ value of approx. 4. This significant isotope effect indicated that the C(sp^3^)–H bond cleavage is involved in the rate determining step in this reaction.

**Scheme 4:**
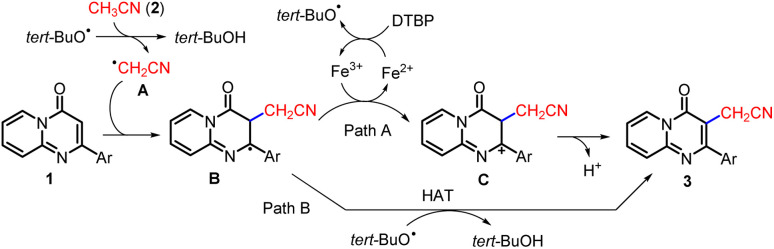
Proposed reaction mechanism.

On the basis of the control experiment and previous literature reports,^21,30^ a plausible mechanism of the reaction is described in Scheme 4. Initially, thermolysis of DTBP generated the *tert*-butoxy radical (*tert*-BuO˙) which on reaction with acetonitrile produced cyanomethyl radical (˙CH_2_CN) intermediate A. Addition of intermediate A at the C3-position of 1 afforded radical intermediate B, which subsequently underwent conversion to the desired product 3 either by path A or path B. In path A, Fe(iii)-mediated single-electron oxidation of B produced intermediate C which on loss of proton gave product 3. In path B, direct hydrogen atom transfer (HAT) mediated by the *tert*-BuO˙ radical produces product 3.^31^

## Conclusions

In summary, an iron(iii)-catalyzed oxidative site-selective cyanomethylation of 4*H*-pyrido[1,2-*a*]pyrimidin-4-ones and quinolin-4(1*H*)-ones has been developed using di-*tert*-butyl peroxide (DTBP) as the oxidant and readily available alkyl nitriles as cyanomethyl source. The method afforded (hetero)arylacetonitrile in good to excellent yields with high regioselectivity and exhibited broad substrate scope and diverse functional groups compatibility. The reaction proceeds *via* a cyanomethyl radical pathway, enabling direct C3–H functionalization under mild conditions. The synthetic utility and robustness of the method is further highlighted by a successful gram-scale reaction, underscoring its potential applicability in medicinal and heterocyclic chemistry.

## Author contributions

A. K. designed and developed this project, A. B. G. performed the experiments and collected data. A. K., A. B. G. discussed and analyzed data and wrote the manuscript with the help of A. B. G.

## Conflicts of interest

There are no conflicts to declare.

## Supplementary Material

RA-OLF-D6RA04026H-s001

RA-OLF-D6RA04026H-s002

## Data Availability

CCDC 2543349 (3ma) and 2543354 (5ba) contain the supplementary crystallographic data for this paper.^32*a*,*b*^ The data supporting this article have been included as part of the supplementary information (SI). Supplementary information: experimental procedures, spectral data and copies of ^1^H, ^13^C{^1^H}, ^19^F NMR spectra of products 3aa–3ac and 5aa–5ac, and single crystal X-ray data for 3ma and 5ba. See DOI: https://doi.org/10.1039/d6ra04026h.

## References

[cit1] Zhang J., Wang L., Ji Q., Liu F. (2023). Org. Lett..

[cit2] Martín-Encinas E., Fuertes M., Delgado-Hernández S., García-Tellado F., Tejedor D., Alonso C. (2024). Front. Pharmacol.

[cit3] Clark J. D., Flanagan M. E., Telliez J.-B. (2014). J. Med. Chem..

[cit4] Chang Q., Liu Z., Liu P., Yu L., Sun P. (2017). J. Org. Chem..

[cit5] Rajmane A., Kumbhar A. (2024). Chem. Pap..

[cit6] Cumming J. G. , Kramer C., Kreis L., Kuehne H. and Schnider P., US20230167127A1, 2023

[cit7] AlNeyadi S. S., Salem A. A., Ghattas M. A., Atatreh N., Abdou I. M. (2017). Eur. J. Med. Chem..

[cit8] Lindsay-Scott P. J., Gallagher P. T. (2017). Tetrahedron Lett..

[cit9] Lourenço N. M. T., Afonso C. A. M. (2003). Tetrahedron.

[cit10] Kalir A., Mualem R. (1987). Synthesis.

[cit11] Wu L., Hartwig J. F. (2005). J. Am. Chem. Soc..

[cit12] Shang R., Ji D.-S., Chu L., Fu Y., Liu L. (2011). Angew. Chem., Int. Ed. Engl..

[cit13] Velcicky J., Soicke A., Steiner R., Schmalz H.-G. (2011). J. Am. Chem. Soc..

[cit14] Chu X.-Q., Ge D., Shen Z.-L., Loh T.-P. (2018). ACS Catal..

[cit15] Lan X.-W., Wang N.-X., Bai C.-B., Lan C.-L., Zhang T., Chen S.-L., Xing Y. (2016). Org. Lett..

[cit16] Chand S., Kumar S., Sharma A. K., Singh K. N. (2024). Org. Lett..

[cit17] Zhong P., Zhang L., Luo N., Liu J. (2023). Catalysts.

[cit18] Su H., Wang L., Rao H., Xu H. (2017). Org. Lett..

[cit19] Qiao K., Zhang D., Zhang K., Yuan X., Zheng M.-W., Guo T.-F., Fang Z., Wan L., Guo K. (2018). Org. Chem. Front..

[cit20] Yao H., Zhong X., Wang B., Lin S., Yan Z. (2022). Org. Lett..

[cit21] Hong G., Nahide P. D., Kozlowski M. C. (2020). Org. Lett..

[cit22] Doraghi F., Kianmehr E., Foroumadi A. (2021). Org. Chem. Front..

[cit23] Liu D., Xia Z., Xiao Y., Yu Y., Yu L., Song Z., Wu Q., Zhang J., Tan Z. (2021). Eur. J. Org Chem..

[cit24] Liu Z.-Q., Li Z. (2016). Commun. Chem..

[cit25] Zhang R., Jin S., Liu Q., Lin S., Yan Z. (2018). J. Org. Chem..

[cit26] Gadekar A. B., Sonam, Shinde V. N., Bhawani, Rangan K., Kumar A. (2024). Adv. Synth. Catal..

[cit27] Sikdar P., Choudhuri T., Paul S., Das S., Kumar A., Bagdi A. K. (2023). Synthesis.

[cit28] Jangir T., Nipate D. S., Dhyani V., Khichar N., Rangan K., Kumar A. (2025). Org. Lett..

[cit29] Turnbull B. W. H., Evans P. A. (2015). J. Am. Chem. Soc..

[cit30] Pan C., Zhang H., Zhu C. (2015). Org. Biomol. Chem..

[cit31] Zhang H., Zhu C. (2017). Org. Chem. Front..

[cit32] (a) CCDC 2543349: Experimental Crystal Structure Determination, 2026, 10.5517/ccdc.csd.cc2rckj7.

