# Supplementary material for: Iron-catalyzed cyanomethylation of 4H-pyrido[1,2-a]pyrimidin-4-ones and quinolin-4(1H)-ones with alkyl nitriles
Source: RSC Adv. 2026 Jul 6;16(36):38160–4. doi: 10.1039/d6ra04026h (PMC13335681; doi:10.1039/d6ra04026h)
Supplement: Supplementary file 1 [file RA-016-D6RA04026H-s001.pdf]

Supplementary Information (SI) for RSC Advances.
This journal is © The Royal Society of Chemistry 2026

Supporting Information

Iron-catalyzed cyanomethylation of 4*H*-pyrido[1,2-*a*]pyrimidin-4-ones and quinolin-4(1*H*)-ones with alkyl nitriles

Amol B. Gadekar,[a] Tarun Jangir,[a] Krishnan Rangan[b] and Anil Kumar[a,*]

[a]Department of Chemistry, Birla Institute of Technology and Science, Pilani, Pilani Campus, Vidya Vihar, Pilani, Rajasthan, 333031, India

[b]Department of Chemistry, Birla Institute of Technology and Science, Pilani, Hyderabad Campus, Jawahar Nagar, Kapra Mandal, Medchal District, Secunderabad, Telangana 500078, India

E-mail: anilkumar@pilani.bits-pilani.ac.in

Contents

1	General information	S2
2	Experimental procedure<br>a) General procedure for the synthesis of **3**<br>b) General procedure for the synthesis of **5**<br>c) Experimental procedure for gram scale synthesis of **3aa**<br>d) Experimental procedure for gram scale synthesis of **5aa**	S2-S3
3	Physical and spectral data of products **3aa-5ac**, **6** and **7**	S3-S14
4	Copies of ^{1}H, ^{13}C{^{1}H} NMR spectra of products **3aa-5ac**, **6** and **7**	S15-S66
5	Copies of HRMS DATA of adducts **8-10**	S67
6	Kinetic isotope effect experiment	S67-68
7	X-ray crystallographic data of **3la** and **5ba**	S68-S70
8	References	S70

1. General information:

The substrate 2-aryl-4*H*-pyrido[1,2-*a*]pyrimidin-4-ones[1] and 2-arylquinolin-4(1*H*)-ones[2] were synthesized by following the reported procedure. All other chemicals and solvents were purchased from commercial suppliers and used without purification unless otherwise noted. All reactions were monitored by thin-layer chromatography (TLC) on pre-coated silica gel 60 F254 aluminium foils and visualized under a UV lamp (366 or 254 nm). Products were isolated by column chromatography (silica gel 100-200 mesh size) using a gradient of ethyl acetate and hexane as mobile phase. The ^{1}H and ^{13}C{^{1}H} NMR spectra were recorded on a Bruker Avance III 400MHz NMR spectrometer. Chemical shifts (δ) are reported in parts per million (ppm), and coupling constants (*J*) are reported in hertz (Hz). High-resolution mass spectra (HRMS) were recorded on an Agilent 6545 Q-TOF LC/MS mass spectrometer. X-ray analysis was performed on a Rigaku Oxford XtaLAB AFC12 (RINC) single-crystal X-ray diffractometer

2. Experimental Procedure

a) General procedure for the synthesis of 3

A 15 mL oven-dried sealed tube was charged with compound **1** (0.22 mmol, 1.0 equiv), **2** (2 mL), DTBP (3.0 equiv) and $Fe(acac)_3$ (10 mol %) at room temperature. The reaction tube was capped tightly, and the reaction mixture was stirred at 120 °C in an oil bath for 24 h. After completion of the reaction (monitored by TLC), the reaction mixture was allowed to attain room temperature, added water (5 mL) and then extracted with ethyl acetate (3 × 5 mL). The combined organic layer was dried over anhydrous Na_2SO_4 and concentrated under reduced pressure. The resulting crude was purified by column chromatography (silica gel 100−200 mesh) using EtOAc-hexane as the eluent to afford the desired products.

b) General procedure for the synthesis of 5

A 15 mL oven-dried sealed tube was charged with compound **4** (0.22 mmol, 1.0 equiv), **2** (2 mL), DTBP (3.0 equiv) and $Fe(acac)_3$ (10 mol %) at room temperature. The reaction tube was capped tightly, and the reaction mixture was stirred at 120 °C in an oil bath for 18 h. After completion of the reaction (monitored by TLC), the reaction mixture was allowed to attain room temperature, added water (5 mL) and then extracted with ethyl acetate (3 × 5 mL). The combined organic layer was dried over anhydrous Na_2SO_4 and concentrated under reduced pressure. The resulting crude was purified by column chromatography (silica gel 100−200 mesh) using EtOAc-hexane as the eluent to afford the desired products.

c) Experimental procedure for gram scale synthesis of 3aa

An oven-dried sealed tube was charged with compound **1a** (4.5 mmol, 1.0 equiv), **2a** (7 mL), DTBP (3.0 equiv), $Fe(acac)_3$ (10 mol %) at room temperature, and the reaction mixture was stirred at 120 °C in an oil bath for 24 h. After completion of the reaction (monitored by TLC), the reaction mixture was allowed to attain room temperature, added water (15 mL) and then extracted with ethyl acetate (3 × 15 mL). The combined organic layer was dried over anhydrous Na_2SO_4 and evaporated under a vacuum. The resulting crude was purified by column chromatography (silica gel 100-200 mesh) using EtOAc-hexane as an eluent to afford **3aa**.

d) Experimental procedure for gram scale synthesis of 5aa

An oven-dried sealed tube was charged with compound **4a** (4.5 mmol, 1.0 equiv.), **2a** (7 mL), DTBP (3.0 equiv.), $Fe(acac)_3$ (10 mol %) at room temperature, and the reaction mixture was stirred at 120 °C in an oil bath for 18 h. After completion of the reaction (monitored by TLC), the reaction mixture was allowed to attain room temperature, added water (15 mL) and then extracted with ethyl acetate (3 × 15 mL). The combined organic layer was dried over anhydrous Na_2SO_4 and evaporated under a vacuum. The resulting crude was purified by column chromatography (silica gel 100-200 mesh) using EtOAc-hexane as an eluent to afford **5aa**.

3. Physical and spectral data of products 3aa-5ac, 6 and 7

2-(4-Oxo-2-phenyl-4*H*-pyrido[1,2-*a*]pyrimidin-3-yl)acetonitrile) (3aa): The title compound was purified by column chromatography on silica gel using EtOAc/ hexanes (2: 3, *v/v*) as an eluent; white solid (41 mg, 70%); mp = 176-177°C; ^{1}H NMR (400 MHz, $CDCl_3$) δ 9.12 (d, J = 7.2 Hz, 1H), 7.86 – 7.83 (m, 1H), 7.76 (d, J = 8.8 Hz, 1H), 7.66 – 7.64 (m, 2H), 7.58 – 7.54 (m, 3H), 7.26 (d, J = 6.4 Hz, 1H), 3.73 (s, 2H); ^{13}C{^{1}H} NMR (100 MHz, $CDCl_3$) δ 163.4, 157.9, 149.8, 137.7, 136.8, 130.0, 129.0, 128.3, 127.3, 126.7, 117.6, 116.1, 104.8, 17.3; HRMS (ESI) *m/z*: [M + H]$^+$ Calcd for $C_{16}H_{12}N_3O^+$ 262.0975; Found 262.0972.

2-(4-Oxo-2-(*p*-tolyl)-4*H*-pyrido[1,2-*a*]pyrimidin-3-yl)acetonitrile (3ba): The title compound was purified by column chromatography on silica gel using EtOAc/ hexanes (2: 3, *v/v*) as an eluent; white solid (37 mg, 64%); mp = 160-161 °C; ^{1}H NMR (400 MHz, $CDCl_3$) δ 9.11 (d, J = 7.2 Hz, 1H), 7.85 – 7.81 (m, 1H), 7.75 (d, J = 8.8 Hz, 1H), 7.56 (d, J = 8.0 Hz, 2H), 7.37 (d, J = 7.6 Hz, 2H), 7.25 (t, J = 7.0 Hz, 1H), 3.75 (s, 2H), 2.47 (s, 3H); ^{13}C{^{1}H} NMR (100 MHz, $CDCl_3$) δ 163.4, 157.9, 149.7, 140.2, 136.7, 134.8, 129.6, 128.4, 127.3, 126.7, 117.8, 116.0, 104.6, 21.5, 17.4; HRMS (ESI) *m/z*: [M + H]$^+$ Calcd for $C_{17}H_{14}N_3O^+$ 276.1131; Found 276.1133.

2-(2-(4-Methoxyphenyl)-4-oxo-4*H*-pyrido[1,2-*a*]pyrimidin-3-yl)acetonitrile (3ca): The title compound was purified by column chromatography on silica gel using EtOAc/ hexanes (2: 3, *v/v*) as an eluent; white solid (38 mg, 66%); mp = 239-240 °C; ^{1}H NMR (400 MHz, $CDCl_3$) δ 9.10 (d, *J* = 7.2 Hz, 1H), 7.85 – 7.80 (m, 1H), 7.74 (d, *J* = 8.8 Hz, 1H), 7.65 (d, *J* = 8.4 Hz, 2H), 7.26 – 7.22 (m, 1H), 7.09 (d, *J* = 8.8 Hz, 2H), 3.91 (s, 3H), 3.77 (s, 2H); ^{13}C{^{1}H} NMR (100 MHz, $CDCl_3$) δ 162.9, 161.0, 158.0, 149.6, 136.6, 130.1, 130.0, 127.3, 126.7, 117.8, 115.9, 114.4, 104.3, 55.5, 17.5; HRMS (ESI) *m/z*: [M + H]$^+$ Calcd for $C_{17}H_{14}N_3O_2^+$ 292.1081; Found 292.1079.

2-(2-(4-Fluorophenyl)-4-oxo-4*H*-pyrido[1,2-*a*]pyrimidin-3-yl)acetonitrile (3da): The title compound was purified by column chromatography on silica gel using EtOAc/ hexanes (2: 3, *v/v*) as an eluent; white solid (34 mg, 58%); mp = 206-207 °C; ^{1}H NMR (400 MHz, $CDCl_3$) δ 9.13 – 9.11 (m, 1H), 7.88 – 7.84 (m, 1H), 7.77 – 7.74 (m, 1H), 7.70 – 7.66 (m, 2H), 7.30 – 7.29 (m, 1H), 7.28 – 7.27 (m, 1H), 7.26 – 7.24 (m, 1H), 3.73 (s, 2H); ^{13}C{^{1}H} NMR (100 MHz, $CDCl_3$) δ 163.7 (d, $^1J_{C\text{-}F}$ = 249.2 Hz), 162.3, 157.9, 149.8, 137.0, 133.8 (d, $^4J_{C\text{-}F}$ = 3.4 Hz), 130.5 (d, $^3J_{C\text{-}F}$ = 8.5 Hz), 127.4, 126.7, 117.5, 116.2, 116.1 (d, $^2J_{C\text{-}F}$ = 18.8 Hz), 104.7, 17.2; ^{19}F NMR (376 MHz, $CDCl_3$) δ = -110.22 (s, 1F); HRMS (ESI) *m/z*: [M + H]$^+$ Calcd for $C_{16}H_{11}FN_3O^+$ 208.0881; Found 208.0883.

2-(2-(4-Chlorophenyl)-4-oxo-4*H*-pyrido[1,2-*a*]pyrimidin-3-yl)acetonitrile (3ea): The title compound was purified by column chromatography on silica gel using EtOAc/ hexanes (2: 3, *v/v*) as an eluent; white solid (32 mg, 56%); mp = 249-250 °C; ^{1}H NMR (400 MHz, $CDCl_3$) δ 9.12 (d, *J* = 7.2 Hz, 1H), 7.86 (t, *J* = 7.4 Hz, 1H), 7.75 (d, *J* = 8.8 Hz, 1H), 7.62 (d, *J* = 8.4 Hz, 2H), 7.55 (d, *J* = 8.4 Hz, 2H), 7.30 – 7.27 (m, 1H), 3.72 (s, 2H); ^{13}C{^{1}H} NMR (100 MHz, $CDCl_3$) δ 162.1, 157.8, 149.8, 137.0, 136.3, 136.1, 129.8, 129.3, 127.4, 126.7, 117.5, 116.3, 104.8, 17.2; HRMS (ESI) *m/z*: [M + H]$^+$ Calcd for $C_{16}H_{11}ClN_3O_2^+$ 296.0585; Found 296.084.

2-(2-(4-Bromophenyl)-4-oxo-4*H*-pyrido[1,2-*a*]pyrimidin-3-yl)acetonitrile (3fa): The title compound was purified by column chromatography on silica gel using EtOAc/ hexanes (2: 3, *v/v*) as an eluent; white solid (34 mg, 60%); mp = 232-233 °C; ^{1}H NMR (400 MHz, $CDCl_3$) δ 9.12 (d, *J* = 7.2 Hz, 1H), 7.88 – 7.85 (m, 1H), 7.75 (d, *J* = 9.2 Hz, 1H), 7.71 (d, *J* = 8.4 Hz, 2H), 7.55 (d, *J* = 8.4 Hz, 2H), 7.30 – 7.27 (m, 1H), 3.72 (s, 2H); ^{13}C{^{1}H} NMR (100 MHz, $CDCl_3$) δ 162.2, 157.8, 149.8, 137.0, 136.5, 132.2, 130.0, 127.4, 126.7, 124.6, 117.4, 116.3, 104.7, 17.2; HRMS (ESI) *m/z*: [M + H]$^+$ Calcd for $C_{16}H_{11}BrN_3O^+$ 340.0080; Found 340.0077.

2-(2-(3-Methoxyphenyl)-4-oxo-4*H*-pyrido[1,2-*a*]pyrimidin-3-yl)acetonitrile (3ga): The title compound was purified by column chromatography on silica gel using EtOAc/ hexanes (2: 3, *v/v*) as an eluent; white solid (35 mg, 60%); mp = 181-182 °C; ^{1}H NMR (400 MHz, $CDCl_3$) δ 9.12 (d, *J* = 6.8 Hz, 1H), 7.87 – 7.83 (m, 1H), 7.77 (d, *J* = 8.8 Hz, 1H), 7.47 (*t*, *J* = 7.8 Hz, 1H), 7.26 (d, *J* = 6.8 Hz, 1H), 7.22 – 7.18 (m, 2H), 7.08 (d, *J* = 8.0 Hz, 1H), 3.90 (s, 3H), 3.74 (s, 2H); $^{13}C\{^1H\}$ NMR (100 MHz, $CDCl_3$) δ 163.3, 159.9, 157.9, 149.8, 138.9, 136.8, 130.1, 127.4, 126.7, 120.5, 117.7, 116.2, 116.1, 113.4, 104.8, 55.5, 17.3; HRMS (ESI) *m/z*: $[M + H]^+$ Calcd for $C_{17}H_{14}N_3O_2^+$ 292.1081; Found 292.1084.

2-(4-Oxo-2-(3-(trifluoromethyl)phenyl)-4*H*-pyrido[1,2-*a*]pyrimidin-3-yl)acetonitrile (3ha): The title compound was purified by column chromatography on silica gel using EtOAc/ hexanes (2: 3, *v/v*) as an eluent; white solid (31 mg, 54%); mp = 211-212 °C; ^{1}H NMR (400 MHz, $CDCl_3$) δ 9.14 (d, *J* = 7.2 Hz, 1H), 7.96 (s, 1H), 7.91 – 7.85 (m, 2H), 7.83 – 7.77 (m, 2H), 7.72 (t, *J* = 7.8 Hz, 1H), 7.31 (t, *J* = 6.8 Hz, 1H), 3.72 (s, 2H); $^{13}C\{^1H\}$ NMR (100 MHz, $CDCl_3$) δ 161.7, 157.8, 149.9, 138.4, 137.2, 131.7, 131.6 (q, $^2J_{C-F}$ = 32.8 Hz), 129.5, 127.4, 126.8, 126.7 (q, $^3J_{C-F}$ = 3.5 Hz), 125.5 (q, $^3J_{C-F}$ = 4.1 Hz), 123.7. (q, $^1J_{C-F}$ = 270.9 Hz), 117.2, 116.5, 105.0 17.1; ^{19}F NMR (376 MHz, $CDCl_3$) δ = -62.70 (s, 3F); HRMS (ESI) *m/z*: $[M + H]^+$ Calcd for $C_{17}H_{11}F_3N_3O^+$ 330.0849; Found 330.0845.

2-(4-Oxo-2-(o-tolyl)-4*H*-pyrido[1,2-*a*]pyrimidin-3-yl)acetonitrile (3ia): The title compound was purified by column chromatography on silica gel using EtOAc/ hexanes (2:3, *v/v*) as an eluent; white solid (33 mg, 57%); mp = 159-160 °C; ^{1}H NMR (400 MHz, $CDCl_3$) δ 9.16 (d, *J* = 6.8 Hz, 1H), 7.85 (t, *J* = 7.6 Hz, 1H), 7.75 (d, *J* = 8.8 Hz, 1H), 7.43 – 7.34 (m, 4H), 7.31 – 7.27 (m, 1H), 3.53 (s, 2H), 2.27 (s, 3H); $^{13}C\{^1H\}$ NMR (100 MHz, $CDCl_3$) δ 164.5, 157.6, 149.8, 136.9, 135.1, 131.0, 129.5, 127.6, 127.4, 126.7, 126.4, 117.0, 116.3, 105.8, 19.5, 16.5; HRMS (ESI) *m/z*: $[M + H]^+$ Calcd for $C_{17}H_{14}N_3O^+$ 276.1131; Found 276.1130.

2-(2-(2-Methoxyphenyl)-4-oxo-4*H*-pyrido[1,2-*a*]pyrimidin-3-yl)acetonitrile (3ja): The title compound was purified by column chromatography on silica gel using EtOAc/ hexanes (2:3, *v/v*) as an eluent; white solid (36 mg, 62%); mp = 196-197 °C; ^{1}H NMR (400 MHz, $CDCl_3$) δ 9.13 (d, *J* = 6.8 Hz, 1H), 7.81 (t, *J* = 8.0 Hz, 1H), 7.75 (d, *J* = 8.8 Hz, 1H), 7.50 (t, *J* = 8.2 Hz, 1H), 7.45 (d, *J* = 7.6 Hz, 1H), 7.24 (d, *J* = 7.4 Hz, 1H), 7.14 (t, *J* = 7.6 Hz, 1H), 7.06 (d, *J* = 8.4 Hz, 1H), 3.88 (s, 3H), 3.52 (s, 2H); $^{13}C\{^1H\}$ NMR (100 MHz, $CDCl_3$) δ 160.9, 157.3, 155.8, 150.0, 136.4, 131.4, 130.1, 127.3, 126.7, 126.4, 121.3, 117.4, 116.0, 111.0, 106.8, 55.4, 17.2. HRMS (ESI) *m/z*: $[M + H]^+$ Calcd for $C_{17}H_{14}N_3O_2^+$ 292.1081; Found 292.1084.

2-(7-Chloro-4-oxo-2-phenyl-4*H*-pyrido[1,2-*a*]pyrimidin-3-yl)acetonitrile (3ka): The title compound was purified by column chromatography on silica gel using EtOAc/ hexanes (2:3, *v/v*) as an eluent; white solid (33 mg, 57%); mp = 235-236 °C; ^{1}H NMR (400 MHz, $CDCl_3$) δ 9.13 (s, 1H), 7.79 – 7.76 (m, 1H), 7.71 (d, J = 9.6 Hz, 1H), 7.66 – 7.64 (m, 2H), 7.60 – 7.57 (m, 3H), 3.74 (s, 2H); ^{13}C{^{1}H} NMR (100 MHz, $CDCl_3$) δ 163.2, 157.0, 148.1, 138.1, 137.3, 130.2, 129.0, 128.4, 127.7, 125.1, 124.9, 117.3, 105.6, 17.3; HRMS (ESI) *m/z*: $[M + H]^+$ Calcd for $C_{16}H_{11}ClN_3O^+$ 296.0585; Found 296.0579.

2-(7-Methyl-4-oxo-2-phenyl-4*H*-pyrido[1,2-*a*]pyrimidin-3-yl)acetonitrile (3la): The title compound was purified by column chromatography on silica gel using EtOAc/ hexanes (2: 3, *v/v*) as an eluent; white solid (34 mg, 59%); mp = 192-193 °C; ^{1}H NMR (400 MHz, $CDCl_3$) δ 8.93 (s, 1H), 7.700 – 7.697 (m, 2H), 7.66 – 7.63 (m, 2H), 7.59 – 7.53 (m, 3H), 3.73 (s, 2H), 2.5 (s, 3H); ^{13}C{^{1}H} NMR (100 MHz, $CDCl_3$) δ 162.9, 157.7, 148.7, 139.8, 137.8, 129.9, 129.0, 128.3, 126.6, 126.2, 124.7, 117.7, 104.4, 18.5, 17.3; HRMS (ESI) *m/z*: $[M + H]^+$ Calcd for $C_{17}H_{14}N_3O^+$ 276.1131; Found 276.1133.

2-(8-Methyl-4-oxo-2-phenyl-4*H*-pyrido[1,2-*a*]pyrimidin-3-yl)acetonitrile (3ma): The title compound was purified by column chromatography on silica gel using EtOAc/ hexanes (2: 3, *v/v*) as an eluent; white solid (37 mg, 63%); mp = 202-203 °C; ^{1}H NMR (400 MHz, $CDCl_3$) δ 9.01 (d, J = 7.2 Hz, 1H), 7.65 – 7.63 (m, 2H), 7.58 – 7.54 (m, 4H), 7.09 (d, J = 6.8 Hz, 1H), 3.71 (s, 2H), 2.54 (s, 3H); ^{13}C{^{1}H} NMR (100 MHz, $CDCl_3$) δ 163.6, 157.9, 149.7, 149.2, 137.9, 129.9, 128.9, 128.3, 126.6, 124.8, 118.8, 117.8, 103.8, 21.6, 17.2; HRMS (ESI) *m/z*: $[M + H]^+$ Calcd for $C_{17}H_{14}N_3O^+$ 276.1131; Found 276.1132.

2-(8-Methyl-4-oxo-2-(*p*-tolyl)-4*H*-pyrido[1,2-*a*]pyrimidin-3-yl)acetonitrile (3na): The title compound was purified by column chromatography on silica gel using EtOAc/ hexanes (2: 3, *v/v*) as an eluent; white solid (37 mg, 63%); mp = 200-201 °C; ^{1}H NMR (400 MHz, $CDCl_3$) δ 9.00 (d, J = 7.2 Hz, 1H), 7.55 – 7.52 (m, 3H), 7.36 (d, J = 7.6 Hz, 2H), 7.08 – 7.06 (m, 1H), 3.72 (s, 2H), 2.53 (s, 3H), 2.46 (s, 3H); ^{13}C{^{1}H} NMR (100 MHz, $CDCl_3$) δ 163.7, 157.9, 149.7, 149.0, 140.0, 135.0, 129.6, 128.3, 126.6, 124.7, 118.7, 117.9, 103.6, 21.6, 21.4, 17.2.; HRMS (ESI) *m/z*: $[M + H]^+$ Calcd for $C_{18}H_{16}N_3O^+$ 290.1288; Found 290.1292.

2-(2-(4-Methoxyphenyl)-8-methyl-4-oxo-4*H*-pyrido[1,2-*a*]pyrimidin-3-yl)acetonitrile (3oa): The title compound was purified by column chromatography on silica gel using EtOAc/ hexanes (2: 3, *v/v*) as an eluent; white solid (36 mg, 64%); mp = 196-197 °C; ^{1}H NMR (400 MHz, $CDCl_3$) δ 8.98 (d, J = 7.6 Hz, 1H), 7.63 (d, J = 8.0 Hz, 2H), 7.51 (s, 1H), 7.08 – 7.06 (m,

3H), 3.91 (s, 3H), 3.74 (s, 2H), 2.53 (s, 3H); $^{13}C\{^1H\}$ NMR (100 MHz, $CDCl_3$) δ 163.2, 160.9, 158.0, 149.6, 149.0, 130.2, 130.1, 126.6, 124.7, 118.6, 118.0, 114.3, 103.3, 55.5, 21.6, 17.4; HRMS (ESI) *m/z*: $[M + H]^+$ Calcd for $C_{18}H_{16}N_3O_2^+$ 206.1237; Found 206.1235.

2-(2-(4-Fluorophenyl)-8-methyl-4-oxo-4*H*-pyrido[1,2-*a*]pyrimidin-3-yl)acetonitrile (3pa): The title compound was purified by column chromatography on silica gel using EtOAc/ hexanes (2: 3, *v/v*) as an eluent; white solid (33 mg, 58%); mp = 216-217 °C; ^1H NMR (400 MHz, $CDCl_3$) δ 9.00 (d, J = 7.2 Hz, 1H), 7.67 – 7.64 (m, 2H), 7.52 (s, 1H), 7.24 (t, J = 8.6 Hz, 2H), 7.10 (d, J = 7.2 Hz, 1H), 3.70 (s, 2H), 2.55 (s, 3H); $^{13}C\{^1H\}$ NMR (100 MHz, $CDCl_3$) δ 163.6 (d, $^1J_{C\text{-}F}$ = 248.8 Hz) 162.5, 157.9, 149.7, 149.4, 134.0 (d,$^4J_{C\text{-}F}$ = 3.3 Hz), 130.5 (d, $^3J_{C\text{-}F}$ = 8.5 Hz), 126.6, 124.7, 118.9, 117.7, 116.1 (d, $^2J_{C\text{-}F}$ = 21.7 Hz), 103.7, 21.6, 17.2; ^{19}F NMR (376 MHz, $CDCl_3$) δ = -110.33 (s, 1F); HRMS (ESI) *m/z*: $[M + H]^+$ Calcd for $C_{17}H_{13}FN_3O^+$ 294.1037; Found 294.1036.

2-(2-(4-Chlorophenyl)-8-methyl-4-oxo-4*H*-pyrido[1,2-*a*]pyrimidin-3-yl)acetonitrile (3qa): The title compound was purified by column chromatography on silica gel using EtOAc/ hexanes (2: 3, *v/v*) as an eluent; white solid (34 mg, 60%); mp = 247-248 °C; ^1H NMR (400 MHz, $CDCl_3$) δ 9.00 (d, J = 7.2 Hz, 1H), 7.60 (d, J = 8.1 Hz, 2H), 7.55 – 7.52 (m, 3H), 7.10 (d, J = 7.0 Hz, 1H), 3.69 (s, 2H), 2.55 (s, 3H); $^{13}C\{^1H\}$ NMR (100 MHz, $CDCl_3$) δ 162.4, 157.8, 149.8, 149.5, 136.3, 136.1, 129.8, 129.2, 126.7, 124.7, 119.0, 117.6, 103.7, 21.6, 17.1; HRMS (ESI) *m/z*: $[M + H]^+$ Calcd for $C_{17}H_{13}ClN_3O^+$ 310.0742; Found 310.0745.

2-(2-(4-Bromophenyl)-8-methyl-4-oxo-4*H*-pyrido[1,2-*a*]pyrimidin-3-yl)acetonitrile (3ra): The title compound was purified by column chromatography on silica gel using EtOAc/ hexanes (2: 3, *v/v*) as an eluent; white solid (35 mg, 62%); mp = 188-189 °C; ^1H NMR (400 MHz, $CDCl_3$) δ 9.00 (d, J = 7.2 Hz, 1H), 7.70 (d, J = 8.4 Hz, 2H), 7.54 – 7.52 (m, 3H), 7.12 – 7.09 (m, 1H), 3.69 (s, 2H), 2.55 (s, 3H); $^{13}C\{^1H\}$ NMR (100 MHz, $CDCl_3$) δ 162.4, 157.8, 149.8, 149.5, 136.7, 132.2, 130.0, 126.7, 124.7, 124.4, 119.0, 117.6, 103.7, 21.6, 17.1; HRMS (ESI) *m/z*: $[M + H]^+$ Calcd for $C_{17}H_{13}BrN_3O^+$ 354.0237; Found 354.0235.

2-(6-Methyl-4-oxo-2-phenyl-4*H*-pyrido[1,2-*a*]pyrimidin-3-yl)acetonitrile (3sa): The title compound was purified by column chromatography on silica gel using EtOAc/ hexanes (2:3, *v/v*) as an eluent; white solid (38 mg, 66%); mp = 180-181 °C; ^1H NMR (400 MHz, $CDCl_3$) δ 7.66 – 7.65 (m, 1H), 7.64 – 7.63 (m, 1H), 7.56 – 7.51 (m, 5H), 6.79 – 6.78 (m, 1H), 3.62 (s, 2H), 3.17 (s, 3H); $^{13}C\{^1H\}$ NMR (100 MHz, $CDCl_3$) δ 161.8, 152.2, 144.0, 137.5, 135.8, 129.9,

128.9, 128.3, 125.6, 118.8, 117.9, 106.4, 24.7, 17.3; HRMS (ESI) *m/z*: $[M + H]^+$ Calcd for $C_{17}H_{14}N_3O^+$ 276.1131; Found 276.1134.

2-(6-Methyl-4-oxo-2-(*p*-tolyl)-4*H*-pyrido[1,2-*a*]pyrimidin-3-yl)acetonitrile (3ta): The title compound was purified by column chromatography on silica gel using EtOAc/ hexanes (2:3, *v/v*) as an eluent; white solid (41 mg, 70%); mp = 204-205 °C; ^{1}H NMR (400 MHz, $CDCl_3$) δ 7.54 (d, J = 8.0 Hz, 2H), 7.52 – 7.50 (m, 1H), 7.49 – 7.46 (m, 1H), 7.35 (d, J = 8.0 Hz, 2H), 6.77 – 6.75 (m, 1H), 3.63 (s, 2H), 3.16 (s, 3H), 2.45 (s, 3H); $^{13}C\{^1H\}$ NMR (100 MHz, $CDCl_3$) δ 161.8, 152.1, 144.0, 140.1, 135.7, 134.6, 129.6, 128.8, 128.4, 125.6, 118.7, 118.0, 106.2, 24.7, 21.4, 17.4; HRMS (ESI) *m/z*: $[M + H]^+$ Calcd for $C_{18}H_{16}N_3O^+$ 290.1288; Found 290.1283.

2-(2-(4-Methoxyphenyl)-6-methyl-4-oxo-4*H*-pyrido[1,2-*a*]pyrimidin-3-yl)acetonitrile (3ua): The title compound was purified by column chromatography on silica gel using EtOAc/ hexanes (2:3, *v/v*) as an eluent; white solid (42 mg, 73%); mp = 190-191 °C; ^{1}H NMR (400 MHz, $CDCl_3$) δ 7.63 (d, J = 8.4 Hz, 2H), 7.53 – 7.46 (m, 2H), 7.06 (d, J = 8.8 Hz, 2H), 6.75 (d, J = 6.0 Hz, 1H), 3.90 (s, 3H), 3.65 (s, 2H), 3.16 (s, 3H); $^{13}C\{^1H\}$ NMR (100 MHz, $CDCl_3$) δ 161.9, 161.3, 161.0, 152.0, 143.9, 135.6, 130.1, 129.8, 125.6, 118.6, 118.1, 114.3, 105.9, 55.5, 24.7, 17.5; HRMS (ESI) *m/z*: $[M + H]^+$ Calcd for $C_{18}H_{16}N_3O_2^+$ 306.1237; Found 306.1241.

2-(2-(4-Fluorophenyl)-6-methyl-4-oxo-4*H*-pyrido[1,2-*a*]pyrimidin-3-yl)acetonitrile (3va): The title compound was purified by column chromatography on silica gel using EtOAc/ hexanes (2: 3, *v/v*) as an eluent; white solid (39 mg, 67%); mp = 243-244 °C; ^{1}H NMR (400 MHz, $CDCl_3$) δ 7.68 – 7.64 (m, 2H), 7.56 – 7.52 (m, 1H), 7.49 – 7.46 (m, 1H), 7.26 – 7.22 (m, 2H), 6.79 (d, J = 6.8 Hz, 1H), 3.61 (s, 2H), 3.16 (s, 3H); $^{13}C\{^1H\}$ NMR (100 MHz, $CDCl_3$) δ 163.6 (d, $^1J_{C\text{-}F}$ = 250.6 Hz), 161.7, 160.7, 152.2, 144.1, 136.0, 133.6 (d, $^4J_{C\text{-}F}$ = 3.4 Hz), 130.5 (d, $^3J_{C\text{-}F}$ = 8.5 Hz), 125.6, 118.9, 117.8, 116.0 (d, $^2J_{C\text{-}F}$ = 21.8 Hz), 106.3, 24.7, 17.3; ^{19}F NMR (376 MHz, $CDCl_3$) δ = -110.00 (s, 1F); HRMS (ESI) *m/z*: $[M + H]^+$ Calcd for $C_{17}H_{13}FN_3O^+$ 294.1037; Found 294.1040.

2-(2-(4-Bromophenyl)-6-methyl-4-oxo-4*H*-pyrido[1,2-*a*]pyrimidin-3-yl)acetonitrile (3wa): The title compound was purified by column chromatography on silica gel using EtOAc/ hexanes (2:3, *v/v*) as an eluent; white solid (34 mg, 60%); mp = 214-216 °C; ^{1}H NMR (400 MHz, $CDCl_3$) 7.69 (d, J = 8.0 Hz, 2H), 7.57 – 7.53 (m, 3H), 7.48 (d, J = 8.8 Hz, 1H), 6.80 (d, J = 6.8 Hz, 1H), 3.60 (s, 2H), 3.17 (s, 3H); $^{13}C\{^1H\}$ NMR (100 MHz, $CDCl_3$) δ 161.7, 160.6,

152.3, 144.1, 136.3, 136.1, 132.2, 130.0, 125.6, 124.5, 119.0, 117.7, 106.3, 24.7, 17.2; HRMS (ESI) *m/z*: $[M + H]^+$ Calcd for $C_{17}H_{13}BrN_3O^+$ 354.0237; Found 354.0241.

2-(6-Methyl-4-oxo-2-(4-(trifluoromethyl)phenyl)-4*H*-pyrido[1,2-*a*]pyrimidin-3-yl)acetonitrile (3xa): The title compound was purified by column chromatography on silica gel using EtOAc/ hexanes (2: 3, *v/v*) as an eluent; white solid (32 mg, 56%); mp = 228-229 °C; ^1H NMR (400 MHz, $CDCl_3$) δ 7.83 (d, J = 8.4 Hz, 2H), 7.77 (d, J = 8.4 Hz, 2H), 7.59 – 7.48 (m, 1H), 7.49 (d, J = 8.7 Hz, 1H), 6.83 (d, J = 6.8 Hz, 1H), 3.59 (s, 2H), 3.18 (s, 3H); $^{13}C\{^1H\}$ NMR (100 MHz, $CDCl_3$) δ 161.6, 160.3, 152.4, 144.2, 141.0, 136.3, 131.9 (q, $^2J_{C-F}$ = 32.0 Hz), 128.8, 125.9 (q, $^3J_{C-F}$ = 3.7 Hz), 125.6, 123.8 (q, ^1J = 270.4 Hz), 119.2, 117.6, 106.6, 24.8, 17.1; ^{19}F NMR (376 MHz, $CDCl_3$) δ = -62.84 (s, 3F); HRMS (ESI) *m/z*: $[M + H]^+$ Calcd for $C_{18}H_{13}F_3N_3O^+$ 344.1005; Found 344.1008.

2-(2-(Furan-2-yl)-4-oxo-4*H*-pyrido[1,2-*a*]pyrimidin-3-yl)acetonitrile (3ya): The title compound was purified by column chromatography on silica gel using EtOAc/ hexanes (2: 3, *v/v*) as an eluent; white solid (35 mg, 59%); mp = 187-188°C; ^1H NMR (400 MHz, $CDCl_3$) δ 9.01 (d, J = 7.2 Hz, 1H), 7.76 (t, J = 7.8 Hz, 2H), 7.66 (d, J = 8.8 Hz, 1H), 7.46 (d, J = 3.6 Hz, 1H), 7.15 (t, J = 6.8 Hz, 1H), 6.69 – 6.65 (m, 1H), 4.25 (s, 2H); $^{13}C\{^1H\}$ NMR (100 MHz, $CDCl_3$) δ 158.5, 152.1, 150.2, 149.8, 145.9, 136.5, 127.4, 126.4, 118.0, 116.3, 115.4, 112.5, 101.5, 15.5; HRMS (ESI) *m/z*: $[M + H]^+$ Calcd for $C_{14}H_{10}N_3O_2^+$ 252.0768; Found 252.0767.

2-(4-Oxo-2-(thiophen-2-yl)-4*H*-pyrido[1,2-*a*]pyrimidin-3-yl)acetonitrile (3za): The title compound was purified by column chromatography on silica gel using EtOAc/ hexanes (2: 3, *v/v*) as an eluent; white solid (36 mg, 62%); mp = 174-175°C; ^1H NMR (400 MHz, $CDCl_3$) δ 9.04 (d, J = 7.2 Hz, 1H), 7.80 (t, J = 7.8 Hz 1H), 7.73 (d, J = 4.0 Hz, 1H), 7.71 (d, J = 9.6 Hz, 1H), 7.66 (d, J = 5.2 Hz, 1H), 7.26 (t, J = 4.4 Hz, 1H), 7.20 (t, J = 7.0 Hz, 1H), 4.04 (s, 2H); $^{13}C\{^1H\}$ NMR (100 MHz, $CDCl_3$) δ 158.2, 155.4, 149.4, 141.2, 136.8, 130.8, 129.9, 128.5, 127.4, 126.5, 117.5, 115.8, 102.6, 17.0; HRMS (ESI) *m/z*: $[M + H]^+$ Calcd for $C_{14}H_{10}N_3OS^+$ 268.0539; Found 268.0541.

2-(2-(5-Methylthiophen-2-yl)-4-oxo-4*H*-pyrido[1,2-*a*]pyrimidin-3-yl)acetonitrile (3αa): The title compound was purified by column chromatography on silica gel using EtOAc/ hexanes (2: 3, *v/v*) as an eluent; white solid (37 mg, 64%); mp = 183-184°C; ^1H NMR (400 MHz, $CDCl_3$) δ 9.01 (d, J = 7.2 Hz, 1H), 7.77 (t, J = 7.8 Hz 1H), 7.66 (d, J = 9.2 Hz, 1H), 7.54 (d, J = 3.6 Hz, 1H), 7.17 (t, J = 6.8 Hz, 1H), 6.92 (d, J = 3.6 Hz, 1H), 4.04 (s, 2H), 2.59 (s, 3H); $^{13}C\{^1H\}$ NMR (100 MHz, $CDCl_3$) δ 158.2, 155.4, 149.3, 146.3, 138.7, 136.6, 130.4, 127.4,

127.0, 126.4, 117.6, 115.6, 101.9, 16.9, 15.6; HRMS (ESI) *m/z*: $[M + H]^+$ Calcd for $C_{15}H_{11}N_3OS^+$ 281.0623; Found 243.0588.

2-(2-Methyl-4-oxo-4*H*-pyrido[1,2-*a*]pyrimidin-3-yl)acetonitrile (3βa): The title compound was purified by column chromatography on silica gel using EtOAc/ hexanes (2:3, *v/v*) as an eluent; white solid (23 mg, 37%); mp = 180-181 °C; ^{1}H NMR (400 MHz, $CDCl_3$) δ 9.04 (d, *J* = 6.8 Hz, 1H), 7.80 (t, *J* = 7.8 Hz, 1H), 7.64 (d, *J* = 8.8 Hz, 1H), 7.21 (t, *J* = 7.0 Hz, 1H), 3.85 (s, 2H), 2.63 (s, 3H); $^{13}C\{^1H\}$ NMR (100 MHz, $CDCl_3$) δ 163.2, 157.1, 149.6, 136.7, 127.5, 126.0, 117.0, 115.8, 104.7, 22.9, 15.1; HRMS (ESI) *m/z*: $[M + H]^+$ Calcd for $C_{11}H_{10}N_3O^+$ 200.0818; Found 200.0816.

2-(5-Oxo-7-phenyl-5*H*-thiazolo[3,2-*a*]pyrimidin-6-yl)acetonitrile (3γa): The title compound was purified by column chromatography on silica gel using EtOAc/hexanes (2:3, *v/v*) as an eluent; white solid (42 mg, 72%); mp = 235-236 °C; ^{1}H NMR (400 MHz, $CDCl_3$) δ 8.08 (d, *J* = 4.8 Hz, 1H), 7.63 – 7.60 (m, 2H), 7.57 – 7.53 (m, 3H), 7.15 (d, *J* = 5.2 Hz, 1H), 3.66 (s, 2H); $^{13}C\{^1H\}$ NMR (100 MHz, $CDCl_3$) δ 162.2, 161.6, 158.3, 137.0, 130.1, 129.0, 128.3, 121.9, 117.5, 112.7, 105.9, 16.6; HRMS (ESI) *m/z*: $[M + H]^+$ Calcd for $C_{14}H_{10}N_3OS^+$ 268.0539; Found 268.0541.

2-(4-Oxo-2-phenyl-4*H*-pyrido[1,2-*a*]pyrimidin-3-yl)propanenitrile (3ab): The title compound was purified by column chromatography on silica gel using EtOAc/ hexanes (2:3, *v/v*) as an eluent; white solid (42 mg, 68%); mp = 185-186 °C; ^{1}H NMR (400 MHz, $CDCl_3$) δ 9.15 (d, *J* = 6.4 Hz, 1H), 7.85 – 7.81 (m, 1H), 7.73 (d, *J* = 8.8 Hz, 2H), 7.56 – 7.51 (m, 5H), 7.28 – 7.24 (m, 1H), 4.10 (q, *J* = 7.1 Hz, 1H), 1.72 (d, *J* = 7.0 Hz, 3H); $^{13}C\{^1H\}$ NMR (100 MHz, $CDCl_3$) δ 162.8, 156.7, 149.6, 138.0, 136.8, 129.7, 129.0, 128.0, 127.2, 126.6, 120.6, 116.0, 110.3, 25.6, 17.1. HRMS (ESI) *m/z*: $[M + H]^+$ Calcd for $C_{17}H_{14}N_3O^+$ 276.1131; Found 276.1133.

2-(4-Oxo-2-phenyl-4*H*-pyrido[1,2-*a*]pyrimidin-3-yl)butanenitrile (3ac): The title compound was purified by column chromatography on silica gel using EtOAc/ hexanes (2:3, *v/v*) as an eluent; white solid (43 mg, 66%); mp = 169-170 °C; ^{1}H NMR (400 MHz, $CDCl_3$) δ 9.14 (d, *J* = 6.8 Hz, 1H), 7.83 (t, *J* = 7.6 Hz, 1H), 7.74 (d, *J* = 8.8 Hz, 1H), 7.56 – 7.50 (m, 5H), 7.26 (t, *J* = 7.0 Hz, 1H), 3.87 (t, *J* = 7.0 Hz, 1H), 2.43 – 2.32 (m, 1H), 1.97 – 1.86 (m, 1H), 0.99 (t, *J* = 7.4 Hz, 3H); $^{13}C\{^1H\}$ NMR (100 MHz, $CDCl_3$) δ 163.4, 156.8, 149.7, 138.1, 136.8, 129.7, 129.0, 128.1, 127.2, 126.6, 119.7, 116.0, 109.3, 33.0, 24.3, 12.2; HRMS (ESI) *m/z*: $[M + H]^+$ Calcd for $C_{18}H_{16}N_3O^+$ 290.1288; Found 290.1286.

2-(4-Oxo-2-phenyl-1,4-dihydroquinolin-3-yl)acetonitrile (5aa): The title compound was purified by column chromatography on silica gel using EtOAc/ hexanes (2:3, *v/v*) as an eluent; off white solid (39 mg, 66%); mp = 207-208 °C; ^{1}H NMR (400 MHz, DMSO-d$_6$) δ 12.05 (s, 1H), 8.18 (d, *J* = 8.0 Hz, 1H), 7.70 – 7.68 (m, 2H), 7.66 – 7.65 (m, 3H), 7.63 – 7.61 (m, 2H), 7.42 – 7.38 (m, 1H), 3.47 (s, 2H); ^{13}C{^{1}H} NMR (100 MHz, DMSO- d$_6$) δ 175.7, 150.3, 140.0, 134.0, 132.7, 130.7, 129.5, 129.0, 125.3, 124.2, 123.9, 119.4, 119.0, 109.2, 15.4; HRMS (ESI) *m/z*: $[M + H]^+$ Calcd for $C_{17}H_{13}N_2O^+$ 261.1022; Found 261.1023.

2-(4-Oxo-2-(*p*-tolyl)-1,4-dihydroquinolin-3-yl)acetonitrile (5ba): The title compound was purified by column chromatography on silica gel using EtOAc/ hexanes (2:3, *v/v*) as an eluent; white solid (41 mg, 70%); mp = 250-251 °C; ^{1}H NMR (400 MHz, DMSO-d$_6$) δ 12.00 (s, 1H), 8.17 (d, *J* = 8.0 Hz, 1H), 7.73 – 7.68 (m, 2H), 7.66 – 7.65 (d, *J* = 7.6 Hz, 2H), 7.46 (d, *J* = 8.0 Hz, 2H), 7.40 (t, *J* = 7.4 Hz, 1H), 3.48 (s, 2H), 2.44 (s, 3H); ^{13}C{^{1}H} NMR (100 MHz, DMSO-d$_6$) δ 175.7, 150.3, 140.5, 140.0, 132.6, 131.1, 130.0, 128.9, 125.3, 124.2, 123.8, 119.5, 119.0, 109.2, 21.4, 15.4; HRMS (ESI) *m/z*: $[M + H]^+$ Calcd for $C_{18}H_{15}N_2O^+$ 275.1179; Found 275.1177.

2-(2-(4-Methoxyphenyl)-4-oxo-1,4-dihydroquinolin-3-yl)acetonitrile (5ca): The title compound was purified by column chromatography on silica gel using EtOAc/ hexanes (2:3, *v/v*) as an eluent; white solid (42 mg, 72%); mp = 207-208 °C; ^{1}H NMR (400 MHz, DMSO-d$_6$) δ 11.97 (s, 1H), 8.16 (d, *J* = 8.0 Hz, 1H), 7.73 – 7.67 (m, 2H), 7.56 (d, *J* = 8.4 Hz, 2H), 7.39 (t, *J* = 6.8 Hz, 1H), 7.21 (d, *J* = 8.4 Hz, 2H), 3.88 (s, 3H), 3.50 (s, 2H); ^{13}C{^{1}H} NMR (100 MHz, DMSO-d$_6$) δ 175.7, 161.1, 150.1, 140.0, 132.6, 130.6, 126.2, 125.3, 124.1, 123.8, 119.5, 119.0, 114.9, 109.2, 55.9, 15.5; HRMS (ESI) *m/z*: $[M + H]^+$ Calcd for $C_{18}H_{15}N_2O_2^+$ 291.1128; Found 291.1131.

2-(2-(4-Fluorophenyl)-4-oxo-1,4-dihydroquinolin-3-yl)acetonitrile (5da): The title compound was purified by column chromatography on silica gel using EtOAc/ hexanes (2:3, *v/v*) as an eluent; white solid (38 mg, 65%); mp = 240-241 °C; ^{1}H NMR (400 MHz, DMSO-d$_6$) δ 12.06 (s, 1H), 8.17 (d, *J* = 8.4 Hz, 1H), 7.74 – 7.65 (m, 4H), 7.51 (t, *J* = 8.6 Hz, 2H), 7.41 (t, *J* = 7.8 Hz, 1H), 3.50 (s, 2H); ^{13}C{^{1}H} NMR (100 MHz, DMSO-d$_6$) δ 175.7, 163.5 (d, $^{1}J_{C-F}$ = 247.6 Hz), 149.3, 140.0, 132.7, 131.6 (d, $^{3}J_{C-F}$ = 8.8 Hz), 130.4 (d, $^{4}J_{C-F}$= 3.2 Hz), 125.4, 124.2, 123.9, 119.3, 119.0, 116.5 (d, $^{2}J_{C-F}$ = 21.9 Hz), 109.5, 15.2; ^{19}F NMR (376 MHz, DMSO-d$_6$) δ = -110.49 (s, 1F); HRMS (ESI) *m/z*: $[M + H]^+$ Calcd for $C_{17}H_{12}FN_2O^+$ 279.0928; Found 279.0931.

2-(2-(4-Chlorophenyl)-4-oxo-1,4-dihydroquinolin-3-yl)acetonitrile (5ea): The title compound was purified by column chromatography on silica gel using EtOAc/ hexanes (2:3, *v/v*) as an eluent; white solid (40 mg, 69%); mp = 260-261 °C; ^{1}H NMR (400 MHz, DMSO-d$_6$) δ 12.06 (s, 1H), 8.17 (d, *J* = 7.6 Hz, 1H), 7.74 – 7.70 (m, 3H), 7.66 – 7.64 (m, 3H), 7.41 (t, *J* = 7.6 Hz, 1H), 3.50 (s, 2H); ^{13}C{^{1}H} NMR (100 MHz, DMSO-d$_6$) δ 175.6, 149.1, 140.0, 135.5, 132.8, 132.7, 131.1, 129.5, 125.4, 124.3, 123.8, 119.3, 119.0, 109.4, 15.2; HRMS (ESI) *m/z*: [M + H]$^+$ Calcd for $C_{17}H_{12}ClN_2O^+$ 295.0633; Found 295.0635.

2-(2-(4-Bromophenyl)-4-oxo-1,4-dihydroquinolin-3-yl)acetonitrile (5fa): The title compound was purified by column chromatography on silica gel using EtOAc/ hexanes (2:3, *v/v*) as an eluent; white solid (38 mg, 67%); mp = 235-236 °C; ^{1}H NMR (400 MHz, DMSO-d$_6$) δ 12.08 (s, 1H), 8.17 (d, *J* = 8.4 Hz, 1H), 7.87 (d, *J* = 8.0 Hz, 2H), 7.73 (t, *J* = 7.8 Hz, 1H), 7.66 (d, *J* = 8.4 Hz, 1H), 7.59 (d, *J* = 8.0 Hz, 2H), 7.41 (t, *J* = 7.4 Hz, 1H), 3.50 (s, 2H); ^{13}C{^{1}H} NMR (100 MHz, DMSO-d$_6$) δ 175.7, 149.1, 140.0, 133.0, 132.8, 132.5, 131.3, 125.4, 124.3, 124.3, 123.9, 119.3, 119.0, 109.4, 15.2; HRMS (ESI) *m/z*: [M + H]$^+$ Calcd for $C_{17}H_{12}BrN_2O^+$ 339.0128; Found 339.0131.

2-(2-(4-Iodophenyl)-4-oxo-1,4-dihydroquinolin-3-yl)acetonitrile (5ga): The title compound was purified by column chromatography on silica gel using EtOAc/ hexanes (2:3, *v/v*) as an eluent; white solid (37 mg, 67%); mp = 235-236 °C; ^{1}H NMR (400 MHz, DMSO-d$_6$) δ 12.05 (s, 1H), 8.17 (d, *J* = 8.0 Hz, 1H), 8.04 (d, *J* = 8.0 Hz, 2H), 7.72 (t, *J* = 7.6 Hz, 1H), 7.65 (d, *J* = 8.4 Hz, 1H), 7.43 – 7.39(m, 3H), 3.49 (s, 2H); ^{13}C{^{1}H} NMR (100 MHz, DMSO-d$_6$) δ 175.6, 149.3, 140.0, 138.3, 133.3, 132.8, 131.1, 125.4, 124.3, 123.9, 119.3, 119.0, 109.3, 97.8, 15.2; HRMS (ESI) *m/z*: [M + H]$^+$ Calcd for $C_{17}H_{12}IN_2O^+$ 386.9989; Found 386.9985.

2-(2-(4-Nitrophenyl)-4-oxo-1,4-dihydroquinolin-3-yl)acetonitrile (5ha): The title compound was purified by column chromatography on silica gel using EtOAc/ hexanes (2:3, *v/v*) as an eluent; white solid (33 mg, 58%); mp = 252-253 °C; ^{1}H NMR (400 MHz, DMSO-d$_6$) δ 12.22 (s, 1H), 8.49 (d, *J* = 8.4 Hz, 2H), 8.19 (d, *J* = 8.0 Hz, 1H), 7.93 (d, *J* = 8.4 Hz, 2H), 7.74 (t, *J* = 7.6 Hz, 1H), 7.66 (d, *J* = 8.4 Hz, 1H), 7.43 (t, *J* = 7.4 Hz, 1H), 3.50 (s, 2H); ^{13}C{^{1}H} NMR (100 MHz, DMSO-d$_6$) δ 175.6, 148.9, 148.2, 140.0, 139.9, 132.9, 131.0, 125.4, 124.5, 124.5, 123.9, 119.1, 119.1.2, 109.6, 15.0; HRMS (ESI) *m/z*: [M + H]$^+$ Calcd for $C_{17}H_{12}N_3O_3^+$ 306.0873; Found 306.0871.

2-(4-Oxo-2-(*o*-tolyl)-1,4-dihydroquinolin-3-yl)acetonitrile (5ia): The title compound was purified by column chromatography on silica gel using EtOAc/ hexanes (2:3, *v/v*) as an eluent;

white solid (35 mg, 60%); mp = 246-247 °C; ^{1}H NMR (400 MHz, DMSO-d_6) δ 11.99 (s, 1H), 8.16 (d, *J* = 8.0 Hz, 1H), 7.73 – 7.66 (m, 2H), 7.51 – 7.45 (m, 4H), 7.40 (t, *J* = 7.0 Hz, 1H), 3.47 (s, 2H), 2.44 (s, 3H); ^{13}C{^{1}H} NMR (100 MHz, DMSO-d_6) δ 175.7, 150.3, 140.5, 140.0, 132.6, 131.1, 130.0, 128.9, 125.3, 124.2, 123.8, 119.5, 119.0, 109.2, 21.4, 15.4; HRMS (ESI) *m/z*: $[M + H]^+$ Calcd for $C_{18}H_{15}N_2O^+$ 275.1179; Found 275.1178.

2-(2-(3-Chlorophenyl)-4-oxo-1,4-dihydroquinolin-3-yl)acetonitrile (5ja): The title compound was purified by column chromatography on silica gel using EtOAc/ hexanes (2:3, *v/v*) as an eluent; white solid (37 mg, 63%); mp = 215-216 °C; ^{1}H NMR (400 MHz, DMSO-d_6) δ 12.10 (s, 1H), 8.18 (d, *J* = 8.0 Hz, 1H), 7.76 (t, *J* = 1.6 Hz, 1H), 7.74 – 7.70 (m, 2H), 7.68 – 7.65 (m, 2H), 7.59 (d, *J* = 7.2 Hz, 1H), 7.41 (t, *J* = 7.4 Hz, 1H), 3.51 (s, 2H); ^{13}C{^{1}H} NMR (100 MHz, DMSO-d_6) δ 175.7, 148.7, 140.0, 135.8, 134.1, 132.8, 131.4, 130.7, 129.0, 128.0, 125.4, 124.3, 123.9, 119.3, 119.0, 109.4, 15.2; HRMS (ESI) *m/z*: $[M + H]^+$ Calcd for $C_{17}H_{12}ClN_2O^+$ 295.0633; Found 295.0635.

2-(4-Oxo-2-(3,4,5-trimethoxyphenyl)-1,4-dihydroquinolin-3-yl)acetonitrile (5ka): The title compound was purified by column chromatography on silica gel using EtOAc/ hexanes (2:3, *v/v*) as an eluent; white solid (38 mg, 68%); mp = 255-256 °C; ^{1}H NMR (400 MHz, DMSO-d_6) δ 12.01 (s, 1H), 8.17 (d, *J* = 8.0 Hz, 1H), 7.74 – 7.66 (m, 2H), 7.40 (t, *J* = 7.0 Hz, 1H), 6.94 (s, 2H), 3.87 (s, 6H), 3.77 (s, 3H), 3.54 (s, 2H); ^{13}C{^{1}H} NMR (100 MHz, DMSO-d_6) δ 175.7, 153.6, 150.2, 139.9, 139.2, 132.6, 129.3, 125.3, 124.2, 123.8, 119.8, 119.0, 109.1, 106.7, 60.6, 56.7, 15.6; HRMS (ESI) *m/z*: $[M + H]^+$ Calcd for $C_{20}H_{19}N_2O_4^+$ 351.1339; Found 351.1343.

2-(2-(5-Methylthiophen-2-yl)-4-oxo-1,4-dihydroquinolin-3-yl)acetonitrile (5la): The title compound was purified by column chromatography on silica gel using EtOAc/ hexanes (2:3, *v/v*) as an eluent; white solid (39 mg, 67%); mp = 226-227 °C; ^{1}H NMR (400 MHz, DMSO-d_6) δ 11.99 (s, 1H), 8.14 (d, *J* = 8.0 Hz, 1H), 7.70 (d, *J* = 3.1 Hz, 1H), 7.41 – 7.37 (m, 3H), 7.06 (d, *J* = 2.4 Hz, 1H), 3.68 (s, 2H), 2.59 (s, 3H); ^{13}C{^{1}H} NMR (100 MHz, DMSO-d_6) δ 175.6, 144.1, 143.5, 140.1, 132.8, 131.0, 126.9, 125.3, 124.3, 123.7, 119.4, 119.0, 110.0, 15.6, 15.4; HRMS (ESI) *m/z*: $[M + H]^+$ Calcd for $C_{16}H_{13}N_2OS^+$ 281.0744; Found 281.0744.

2-(7-Methyl-4-oxo-2-phenyl-1,4-dihydroquinolin-3-yl)acetonitrile (5ma): The title compound was purified by column chromatography on silica gel using EtOAc/ hexanes (2:3, *v/v*) as an eluent; white solid (38 mg, 66%); mp = 225-226 °C; ^{1}H NMR (400 MHz, DMSO-d_6) δ 11.93 (s, 1H), 8.06 (d, *J* = 8.0 Hz, 1H), 7.66 – 7.59 (m, 5H), 7.43 (s, 1H), 7.23 (d, *J* = 8.0

Hz, 1H), 3.44 (s, 2H), 2.44 (s, 3H); $^{13}C\{^{1}H\}$ NMR (100 MHz, DMSO-d_6) δ 175.5, 150.0, 142.9, 140.2, 134.0, 130.6, 129.5, 129.0, 126.0, 125.3, 121.9, 119.4, 118.2, 109.0, 21.9, 15.3; HRMS (ESI) *m/z*: $[M + H]^+$ Calcd for $C_{18}H_{15}N_2O^+$ 275.1179; Found 275.1180.

2-(6,7-Dimethoxy-4-oxo-2-phenyl-1,4-dihydroquinolin-3-yl)acetonitrile (5na): The title compound was purified by column chromatography on silica gel using EtOAc/ hexanes (2:3, *v/v*) as an eluent; white solid (40 mg, 71%); mp = 249-250 °C; ^{1}H NMR (400 MHz, DMSO-d_6) δ 11.87 (s, 1H), 7.66 – 7.62 (m, 3H), 7.62 – 7.56 (m, 2H), 7.50 (s, 1H), 7.12 (s, 1H), 3.88 (s, 3H), 3.86 (s, 3H), 3.46 (s, 2H); $^{13}C\{^{1}H\}$ NMR (100 MHz, DMSO-d_6) δ 174.5, 153.7, 148.6, 147.5, 135.8, 134.1, 130.6, 129.5, 129.0, 119.6, 117.9, 108.1, 104.4, 99.7, 56.1, 56.0, 15.4; HRMS (ESI) *m/z*: $[M + H]^+$ Calcd for $C_{19}H_{17}N_2O_3^+$ 321.1234; Found 321.1237.

2-(1-Methyl-4-oxo-2-phenyl-1,4-dihydroquinolin-3-yl)acetonitrile (5oa): The title compound was purified by column chromatography on silica gel using EtOAc/ hexanes (2:3, *v/v*) as an eluent; white solid (30 mg, 51%); mp = 205-206 °C; ^{1}H NMR (400 MHz, DMSO-d_6) δ 8.31 (d, J = 8.0 Hz, 1H), 7.85 (d, J = 4.0 Hz, 2H), 7.69 – 7.64 (m, 3H), 7.54 – 7.52 (m, 1H), 7.51 – 7.48 (m, 2H), 3.48 (s, 3H), 3.24 (s, 2H); $^{13}C\{^{1}H\}$ NMR (100 MHz, DMSO-d_6) δ 179.5, 158.4, 146.1, 138.7, 138.0, 135.2, 134.7, 133.4, 130.7, 129.9, 129.3, 123.8, 122.7, 115.8, 42.8, 21.1; HRMS (ESI) *m/z*: $[M + H]^+$ Calcd for $C_{18}H_{15}N_2O^+$ 275.1179; Found 275.1177.

2-(4-Oxo-2-phenyl-1,4-dihydroquinolin-3-yl)propanenitrile (5ab): The title compound was purified by column chromatography on silica gel using EtOAc/ hexanes (2:3, *v/v*) as an eluent; white solid (41 mg, 66%); mp = 199-200 °C; ^{1}H NMR (400 MHz, DMSO-d_6) δ 11.96 (s, 1H), 8.17 (d, J = 8.0 Hz, 1H), 7.70 (t, J = 7.6 Hz, 1H), 7.65 – 7.63 (m, 4H), 7.59 – 7.56 (m, 2H), 7.40 (t, J = 7.4 Hz, 1H), 3.65 (q, J = 7.0 Hz, 1H), 1.49 (d, J = 6.8 Hz, 3H); $^{13}C\{^{1}H\}$ NMR (100 MHz, DMSO-d_6) δ 175.3, 149.7, 139.8, 134.1, 132.6, 130.6, 129.5, 129.0, 125.3, 124.5, 124.1, 121.9, 118.9, 114.3, 24.3, 17.5; HRMS (ESI) *m/z*: $[M + H]^+$ Calcd for $C_{18}H_{15}N_2O^+$ 275.1179; Found 275.1176.

2-(4-Oxo-2-phenyl-1,4-dihydroquinolin-3-yl)butanenitrile (5ac): The title compound was purified by column chromatography on silica gel using EtOAc/ hexanes (2:3, *v/v*) as an eluent; white solid (40 mg, 61%); mp = 237-238 °C; ^{1}H NMR (400 MHz, DMSO-d_6) δ 11.99 (s, 1H), 8.16 (d, J = 8.4 Hz, 1H), 7.70 (t, J = 7.6 Hz, 1H), 7.65 – 7.63 (m, 4H), 7.57 – 7.54 (m, 2H), 7.39 (t, J = 7.4 Hz, 1H), 3.42 (t, J = 7.8 Hz, 1H), 2.13 – 2.02 (m, 1H), 1.85 – 1.74 (m, 1H), 0.79 (t, J = 7.2 Hz, 3H); $^{13}C\{^{1}H\}$ NMR (100 MHz, DMSO) δ 175.4, 150.3, 139.8, 134.1, 132.6,

130.6, 129.5, 129.1, 125.3, 124.5, 124.1, 121.0, 118.9, 113.0, 31.7, 24.2, 12.4; HRMS (ESI) *m/z*: $[M + H]^+$ Calcd for $C_{19}H_{17}N_2O^+$ 289.1335; Found 289.1330.

Methyl 2-(4-oxo-2-phenyl-1,4-dihydroquinolin-3-yl)acetate (6): The title compound was purified by column chromatography on silica gel using EtOAc/ hexanes (2:3, *v/v*) as an eluent; white solid (38 mg, 68%); mp = 219-220°C; ^1H NMR (400 MHz, $CDCl_3$) δ 9.79 (s, 1H), 8.19 (d, J = 8.1 Hz, 1H), 7.59 – 7.56 (m, 2H), 7.50 – 7.48 (m, 2H), 7.40 – 7.38 (m, 3H), 7.30 – 7.26 (m, 1H), 3.59 (s, 3H), 3.41 (s, 2H); $^{13}C\{^1H\}$ NMR (100 MHz, $CDCl_3$) δ 177.9, 172.9, 149.3, 139.4, 134.6, 132.0, 129.9, 128.8, 128.4, 126.0, 124.0, 123.7, 117.7, 114.2, 51.9, 32.5; HRMS (ESI) *m/z*: $[M + H]^+$ Calcd for $C_{18}H_{16}INO_3^+$ 294.1125; Found 294.1126.

2-(4-Oxo-2-phenyl-1,4-dihydroquinolin-3-yl)acetamide (7): The title compound was purified by column chromatography on silica gel using EtOAc/ hexanes (2:3, *v/v*) as an eluent; white solid (39 mg, 74%); mp = 294-295 °C; ^1H NMR (400 MHz, DMSO-d_6) δ 11.73 (s, 1H), 8.13 (d, J = 8.4 Hz, 1H), 7.66 – 7.61 (m, 4H), 7.58 – 7.56 (m, 3H), 7.36 – 7.31 (m, 1H), 7.27 (s, 1H), 6.75 (s, 1H), 3.12 (s, 2H); $^{13}C\{^1H\}$ NMR (100 MHz, DMSO-d_6) δ 176.9, 173.4, 149.7, 140.1, 135.2, 132.0, 130.0, 129.4, 129.0, 125.4, 123.9, 123.4, 118.7, 114.6, 34.0; HRMS (ESI) *m/z*: $[M + H]^+$ Calcd for $C_{17}H_{15}IN_2O_2^+$ 279.1128; Found 279.1125.

4. Copies of ^{1}H, ^{13}C{^{1}H} NMR spectra of products 3aa-5ac. 6 and 7

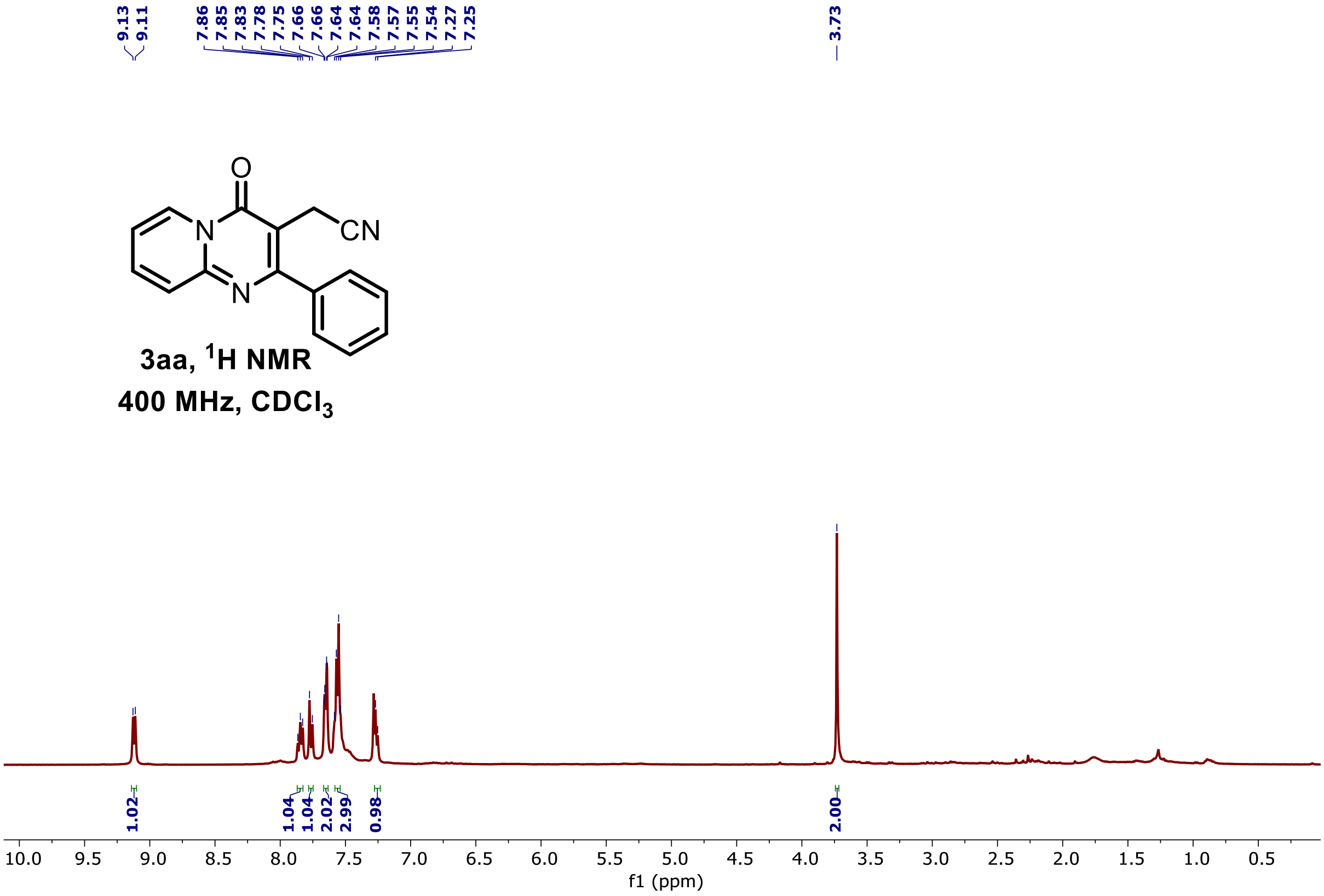

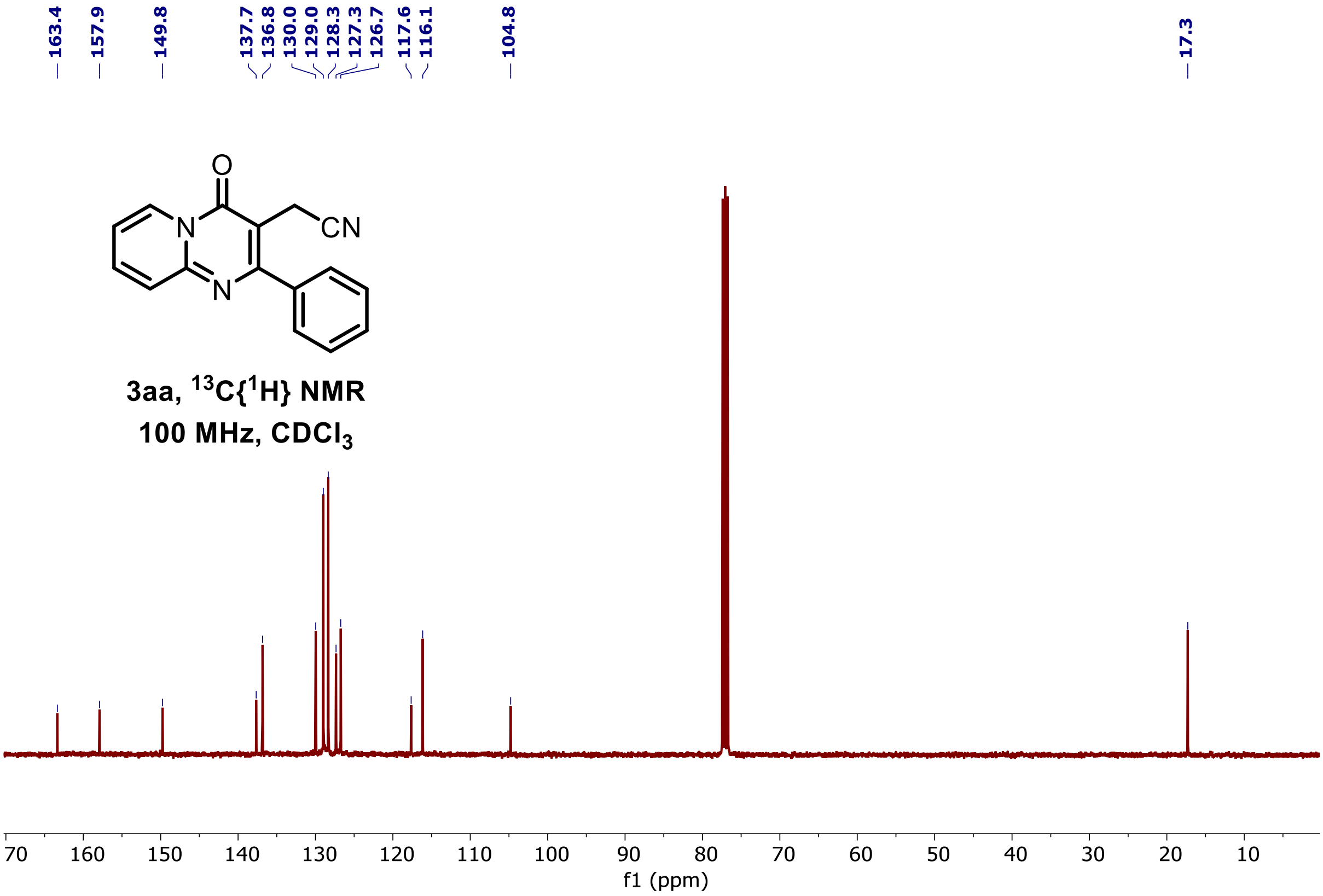

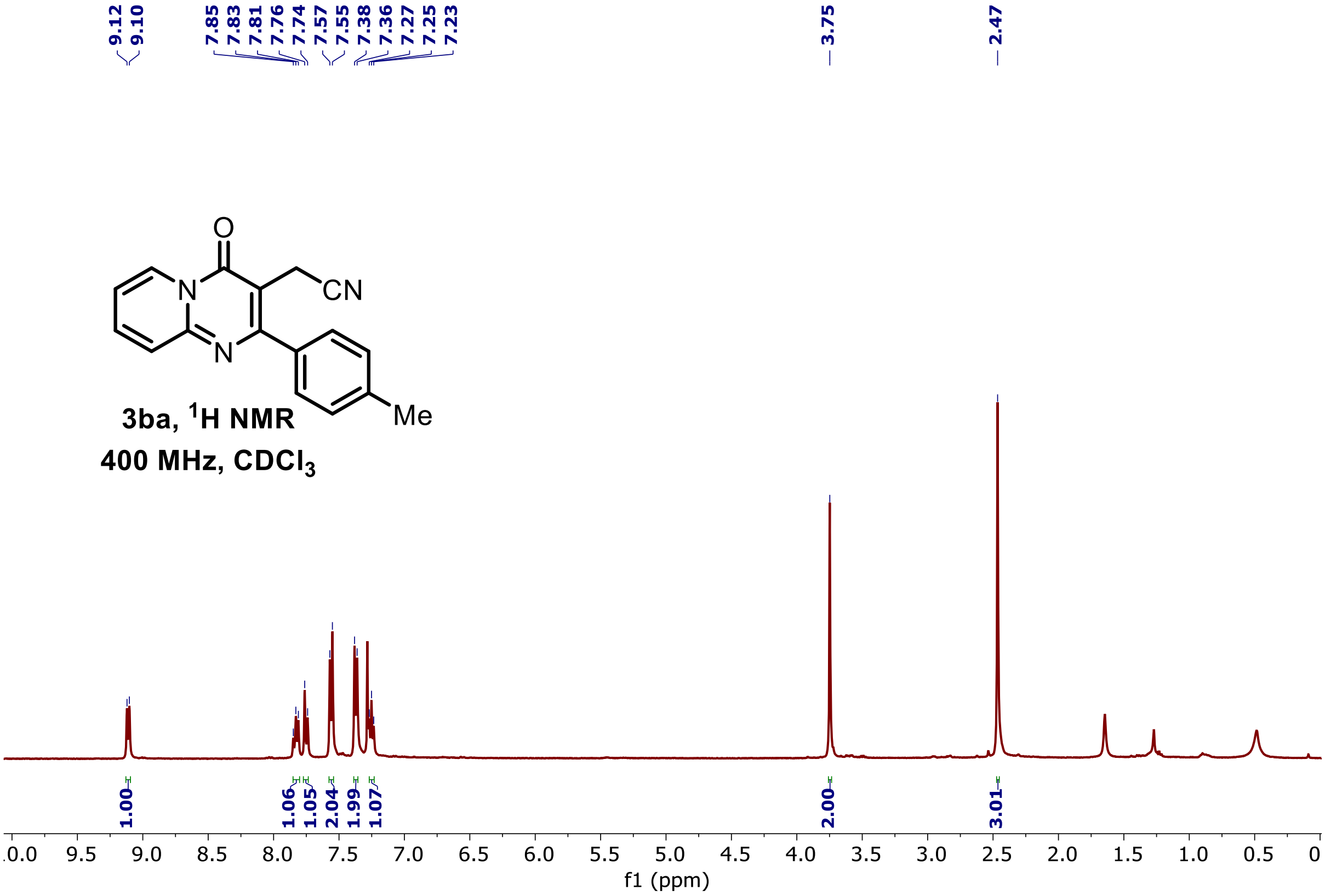
9.12
9.10
7.85
7.83
7.81
7.76
7.74
7.57
7.55
7.38
7.36
7.27
7.25
7.23
3.75
2.47
O
N
CN
N
Me
3ba, ^{1}H NMR
400 MHz, CDCl$_3$
1.00
1.06
1.05
2.04
1.99
1.07
2.00
3.01
f1 (ppm)

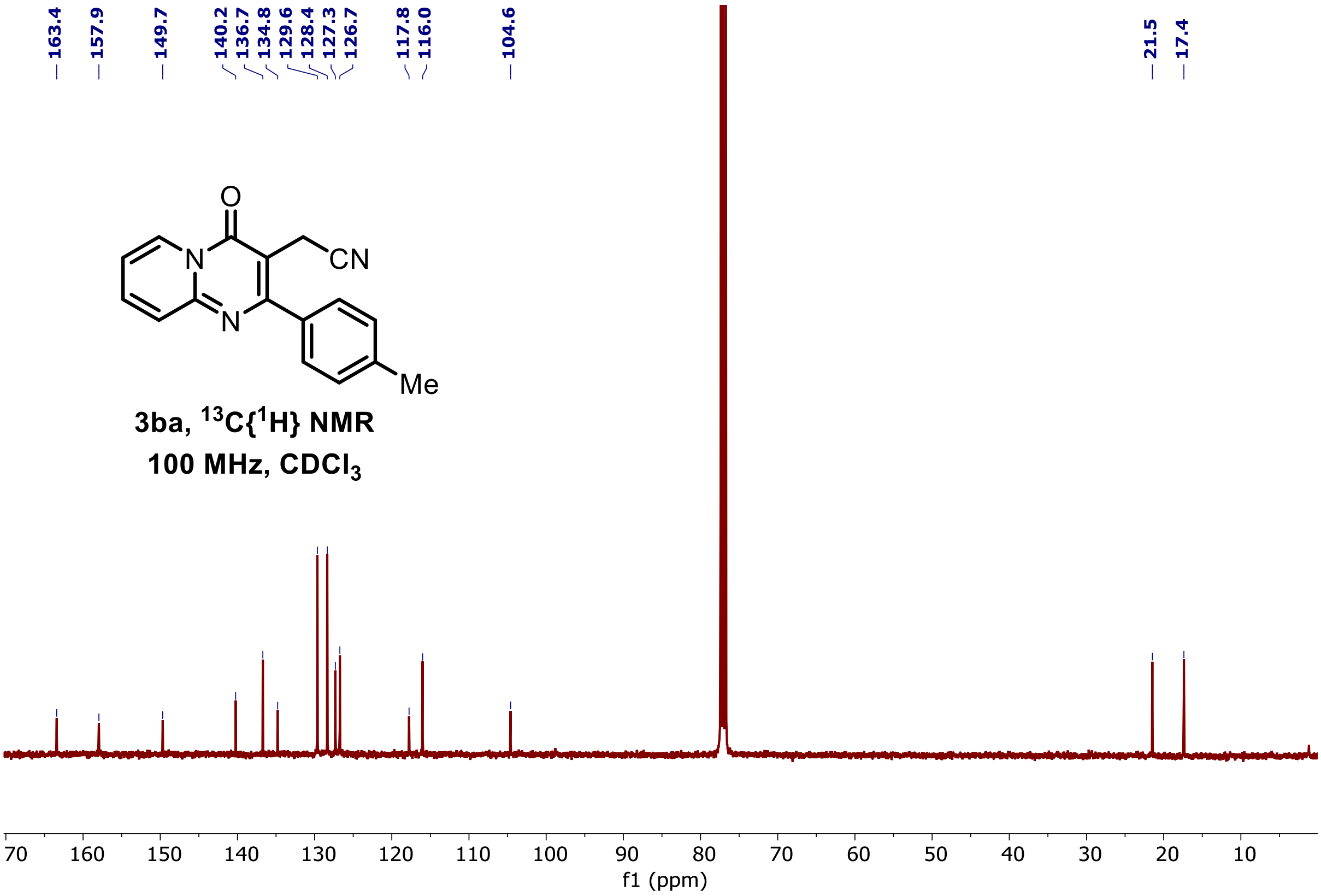
163.4
157.9
149.7
140.2
136.7
134.8
129.6
128.4
127.3
126.7
117.8
116.0
104.6
21.5
17.4
O
N
CN
N
Me
3ba, ^{13}C{^{1}H} NMR
100 MHz, CDCl$_3$
f1 (ppm)

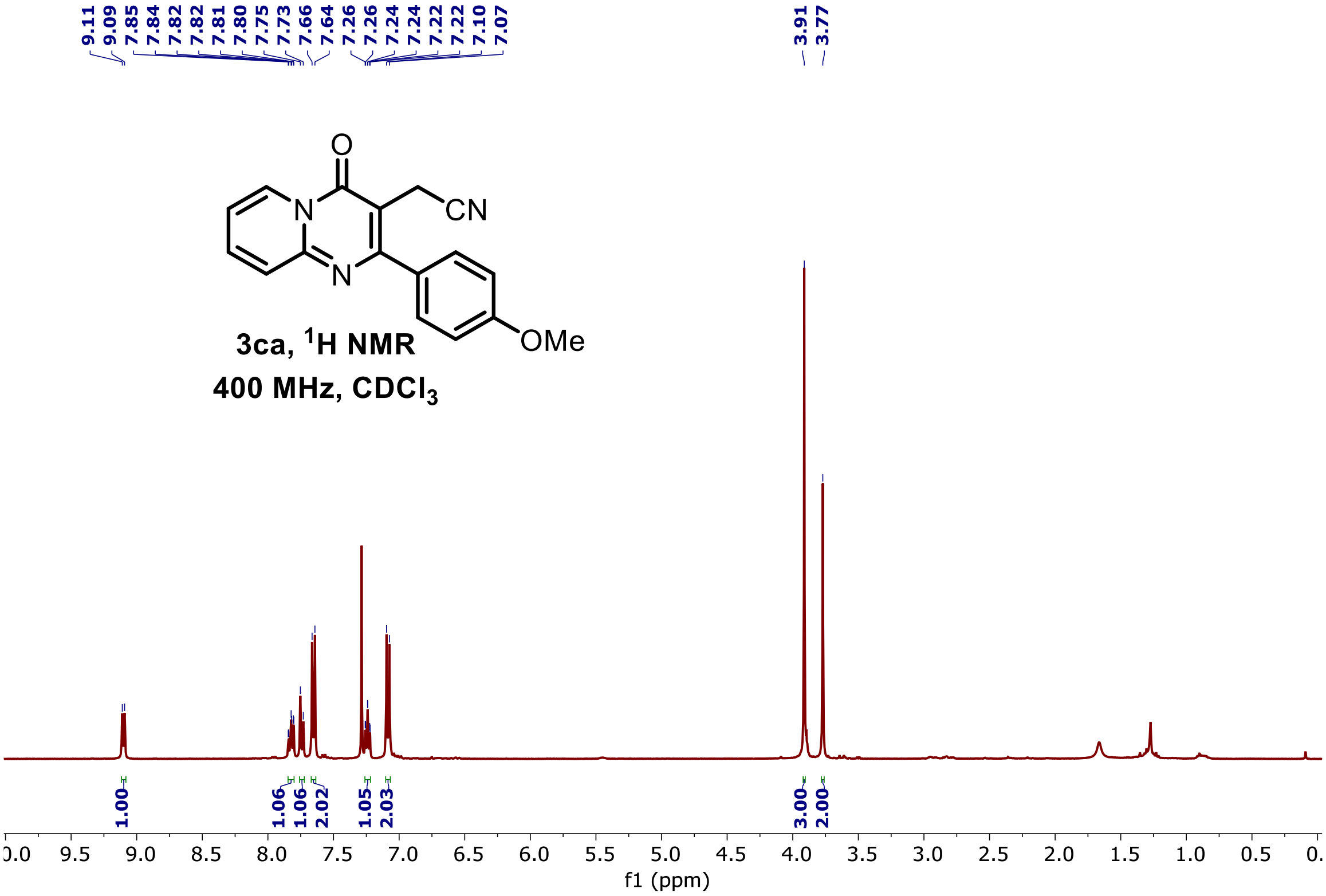
9.11
9.09
7.85
7.84
7.82
7.82
7.81
7.80
7.75
7.73
7.66
7.64
7.26
7.26
7.24
7.24
7.22
7.22
7.10
7.07
3.91
3.77
O
N
N
CN
OMe
3ca, ^{1}H NMR
400 MHz, $CDCl_3$
1.00
1.06
1.06
2.02
1.05
2.03
3.00
2.00
f1 (ppm)

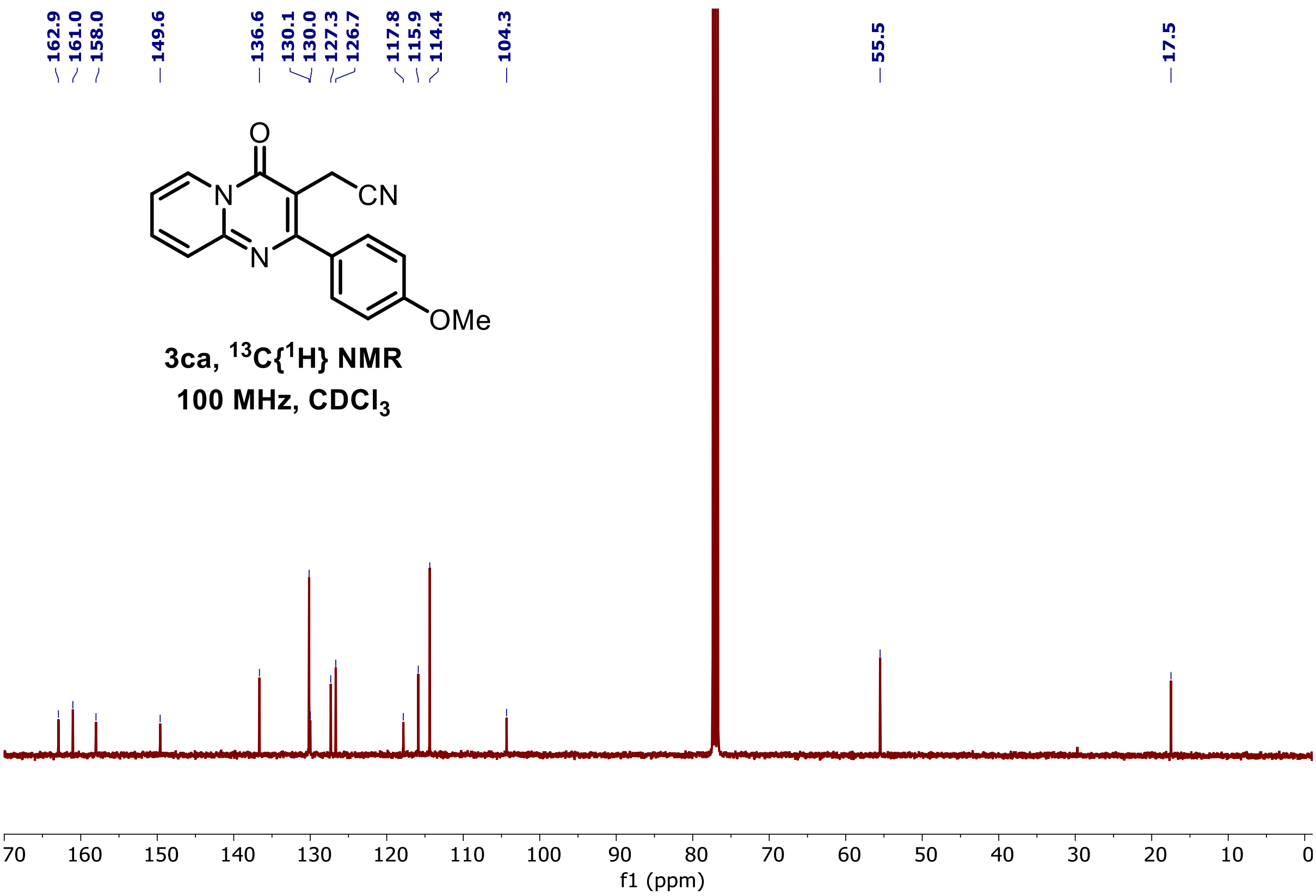
162.9
161.0
158.0
149.6
136.6
130.1
130.0
127.3
126.7
117.8
115.9
114.4
104.3
55.5
17.5
O
N
N
CN
OMe
3ca, ^{13}C{^{1}H} NMR
100 MHz, $CDCl_3$
f1 (ppm)

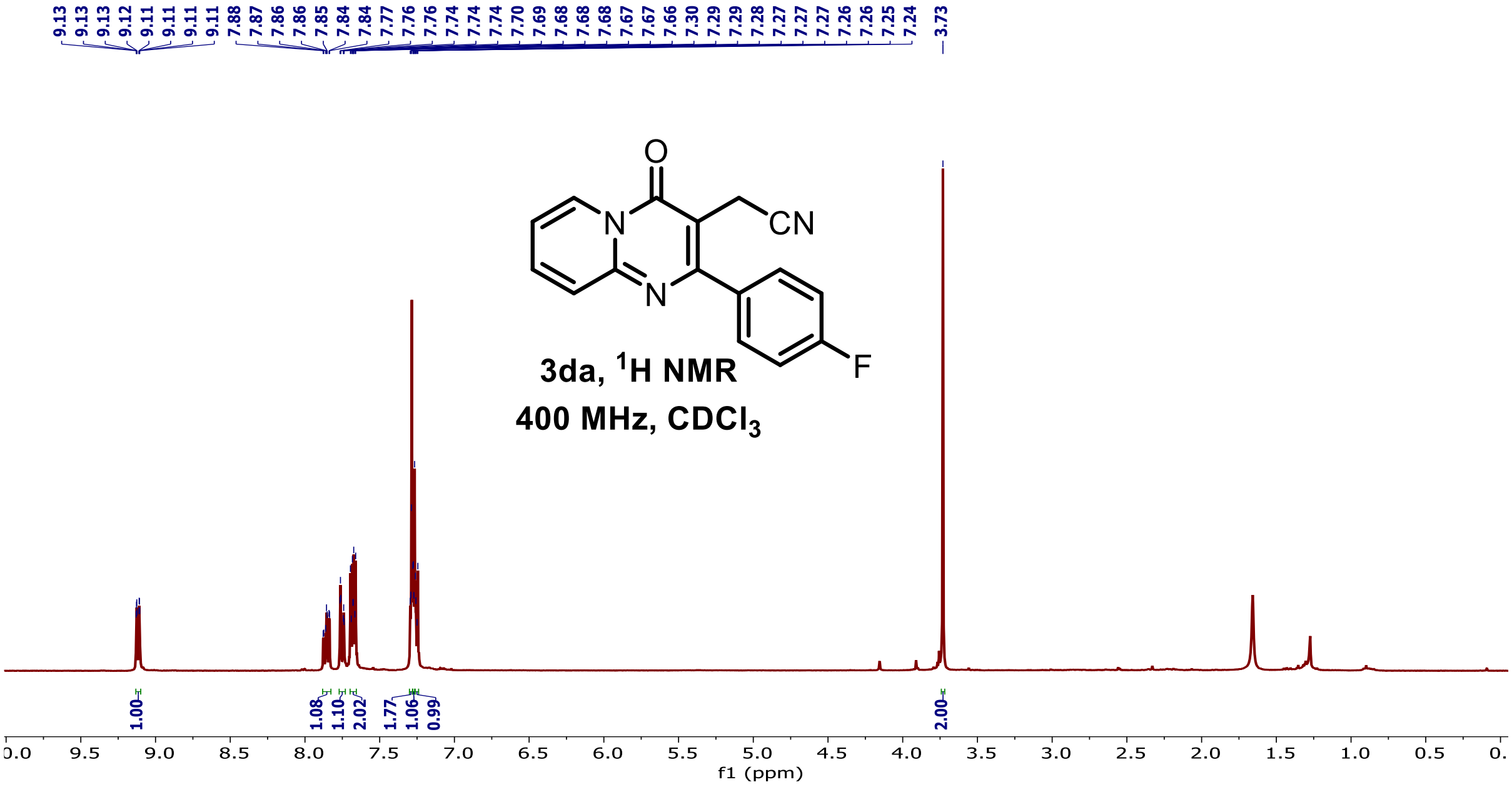
9.13 9.13 9.13 9.12 9.11 9.11 9.11 9.11 9.11 7.88 7.87 7.86 7.86 7.85 7.84 7.84 7.77 7.76 7.76 7.74 7.74 7.74 7.70 7.69 7.68 7.68 7.68 7.67 7.67 7.66 7.30 7.29 7.29 7.28 7.27 7.27 7.27 7.26 7.26 7.25 7.24
3.73
O
N
CN
N
F
3da, 1H NMR
400 MHz, CDCl3
1.00
1.08
1.10
2.02
1.77
1.06
0.99
2.00
f1 (ppm)

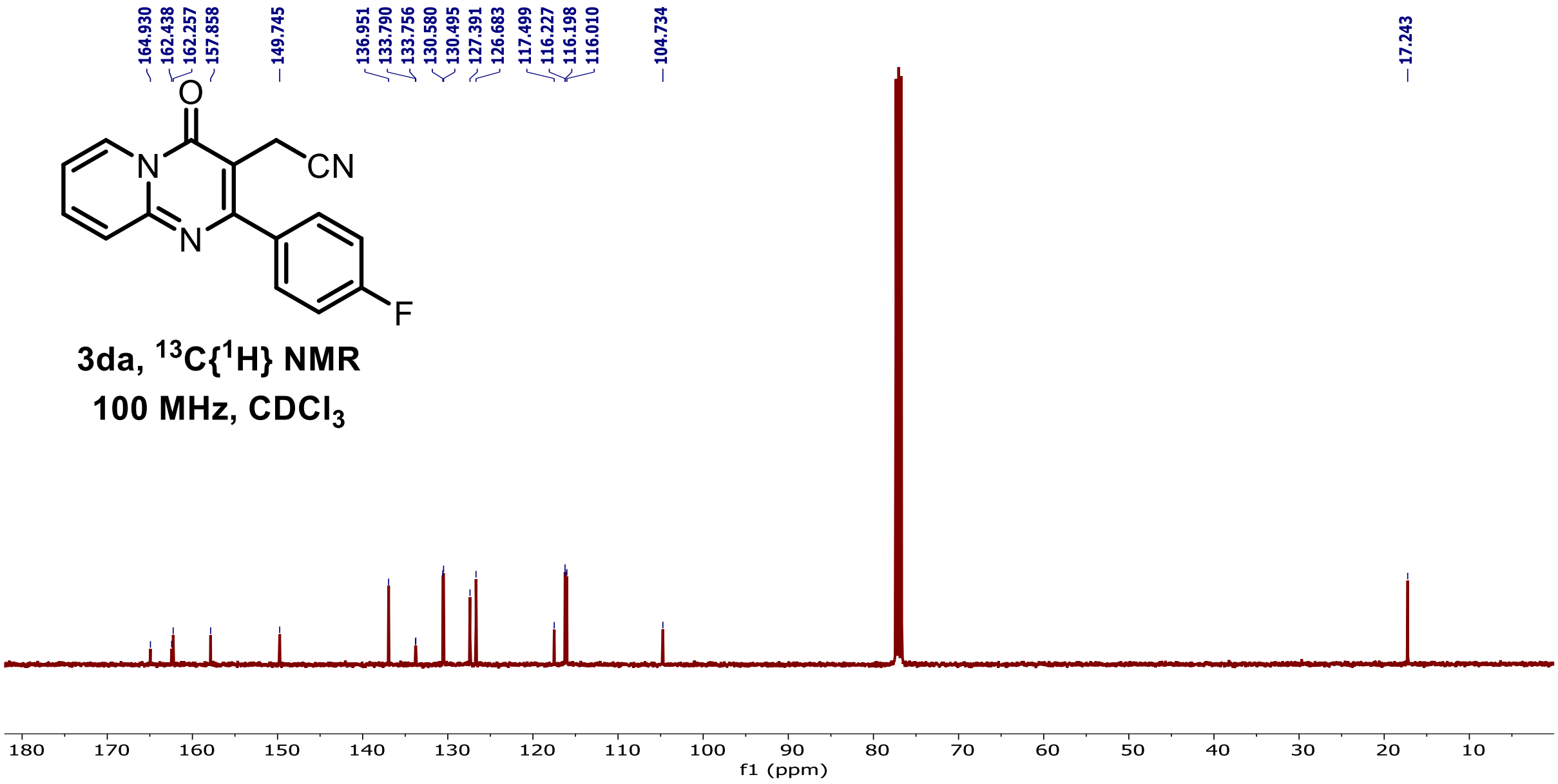
164.930 162.438 162.257 157.858
149.745
136.951 133.790 133.756 130.580 130.495 127.391 126.683 117.499 116.227 116.198 116.010
104.734
17.243
O
N
CN
N
F
3da, 13C{1H} NMR
100 MHz, CDCl3
f1 (ppm)

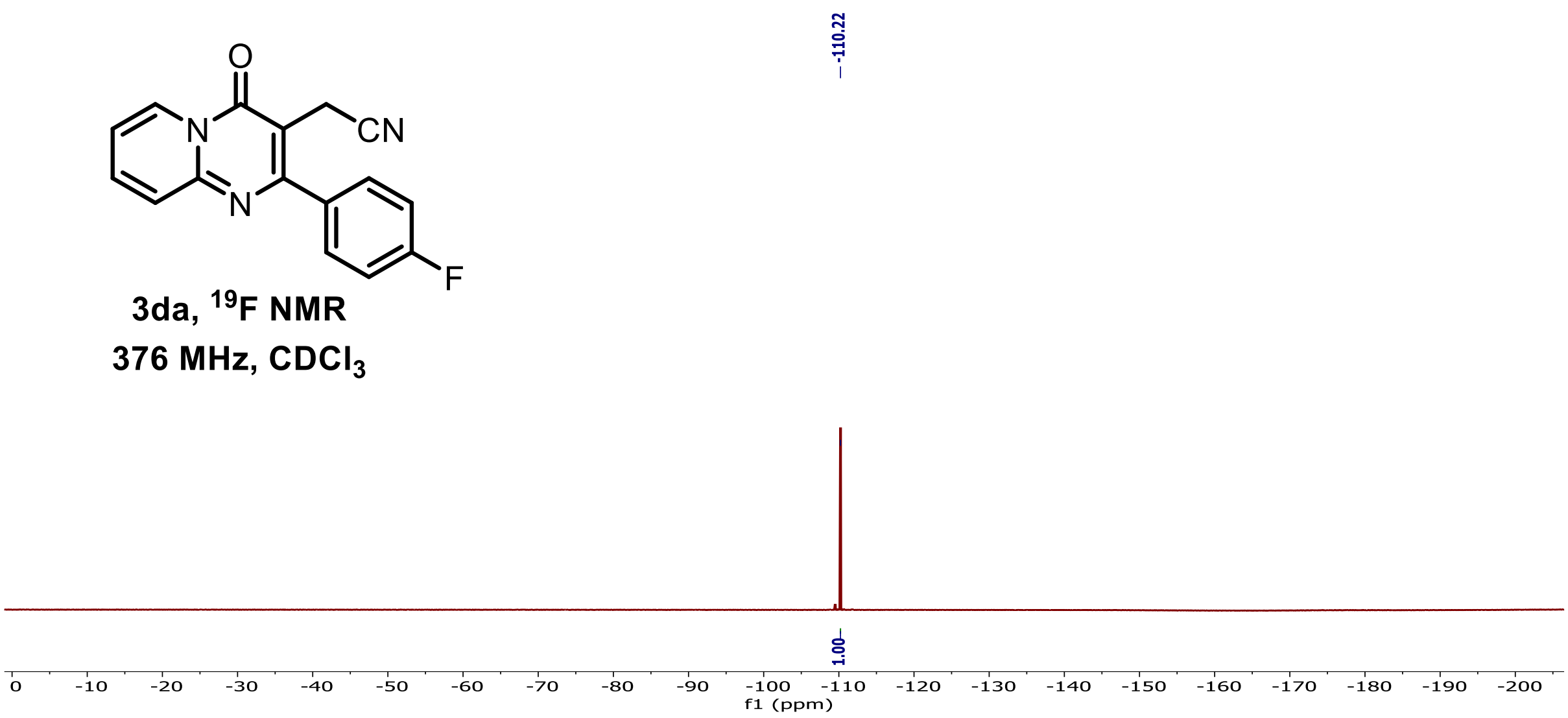
-110.22
O
N
CN
N
F
3da, 19F NMR
376 MHz, CDCl3
1.00
f1 (ppm)

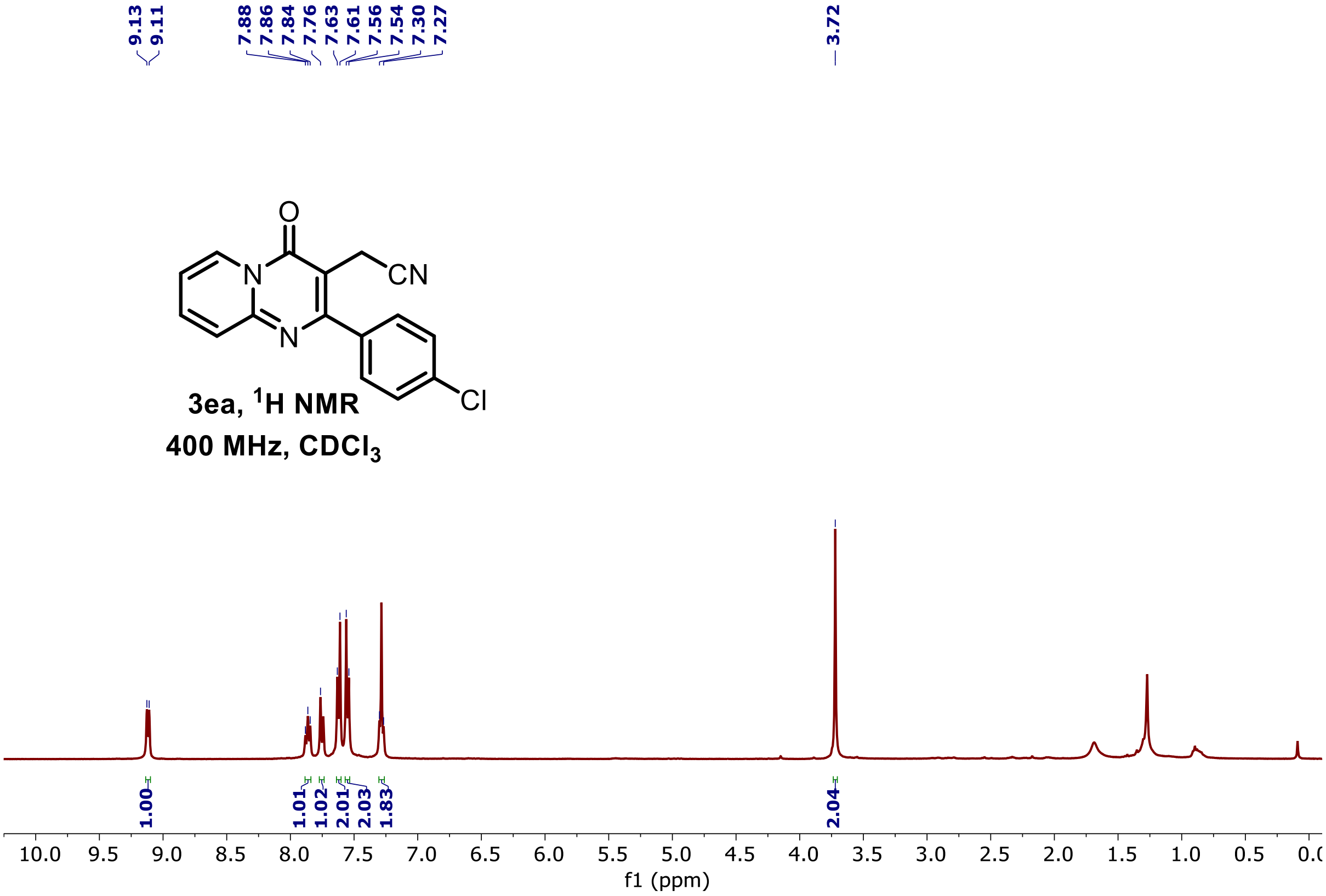
9.13
9.11
7.88
7.86
7.84
7.76
7.63
7.61
7.56
7.54
7.30
7.27
3.72
O
N
CN
N
Cl
3ea, ¹H NMR
400 MHz, CDCl₃
1.00
1.01
1.02
2.01
2.03
1.83
2.04
f1 (ppm)

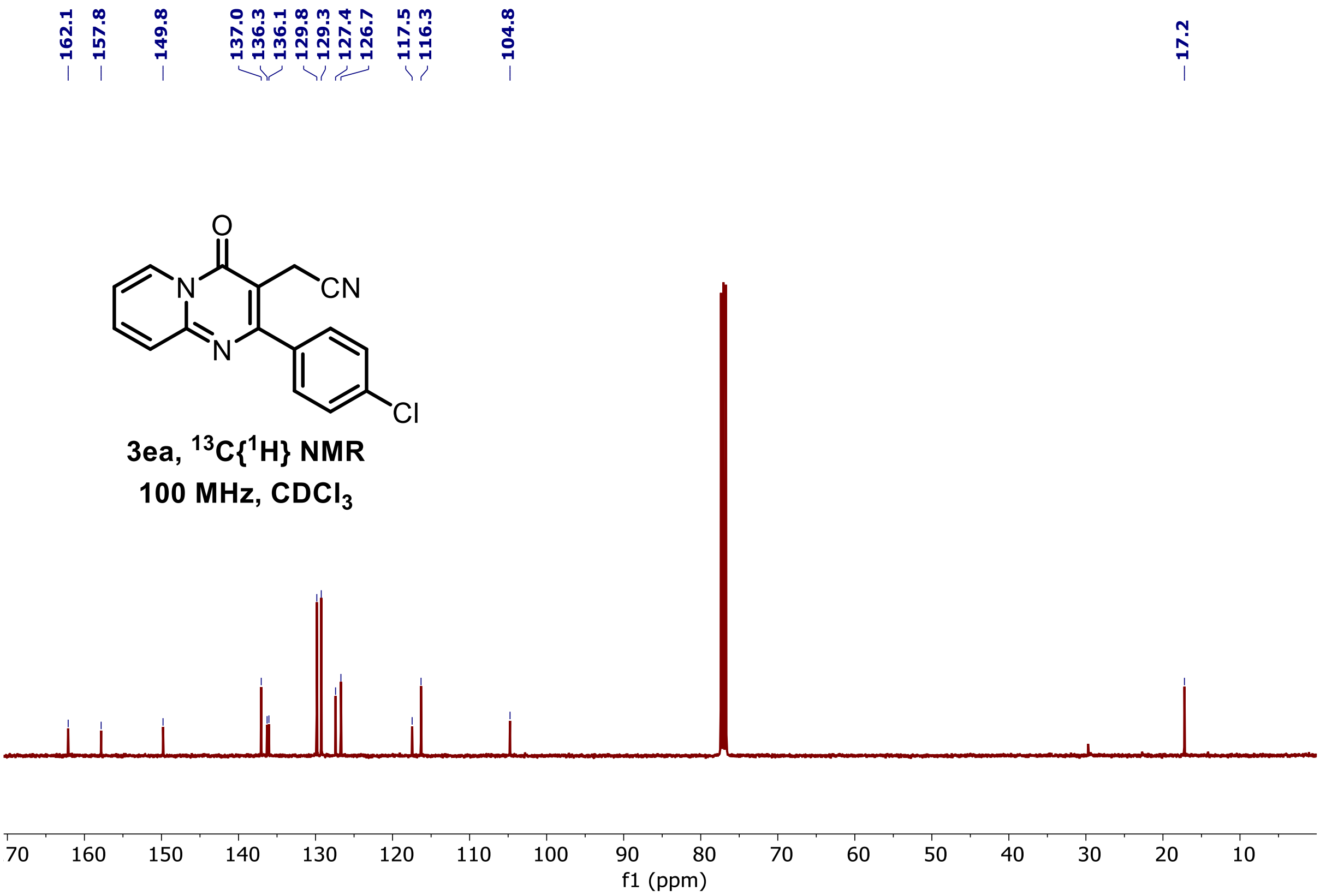
162.1
157.8
149.8
137.0
136.3
136.1
129.8
129.3
127.4
126.7
117.5
116.3
104.8
17.2
O
N
CN
N
Cl
3ea, ¹³C{¹H} NMR
100 MHz, CDCl₃
f1 (ppm)

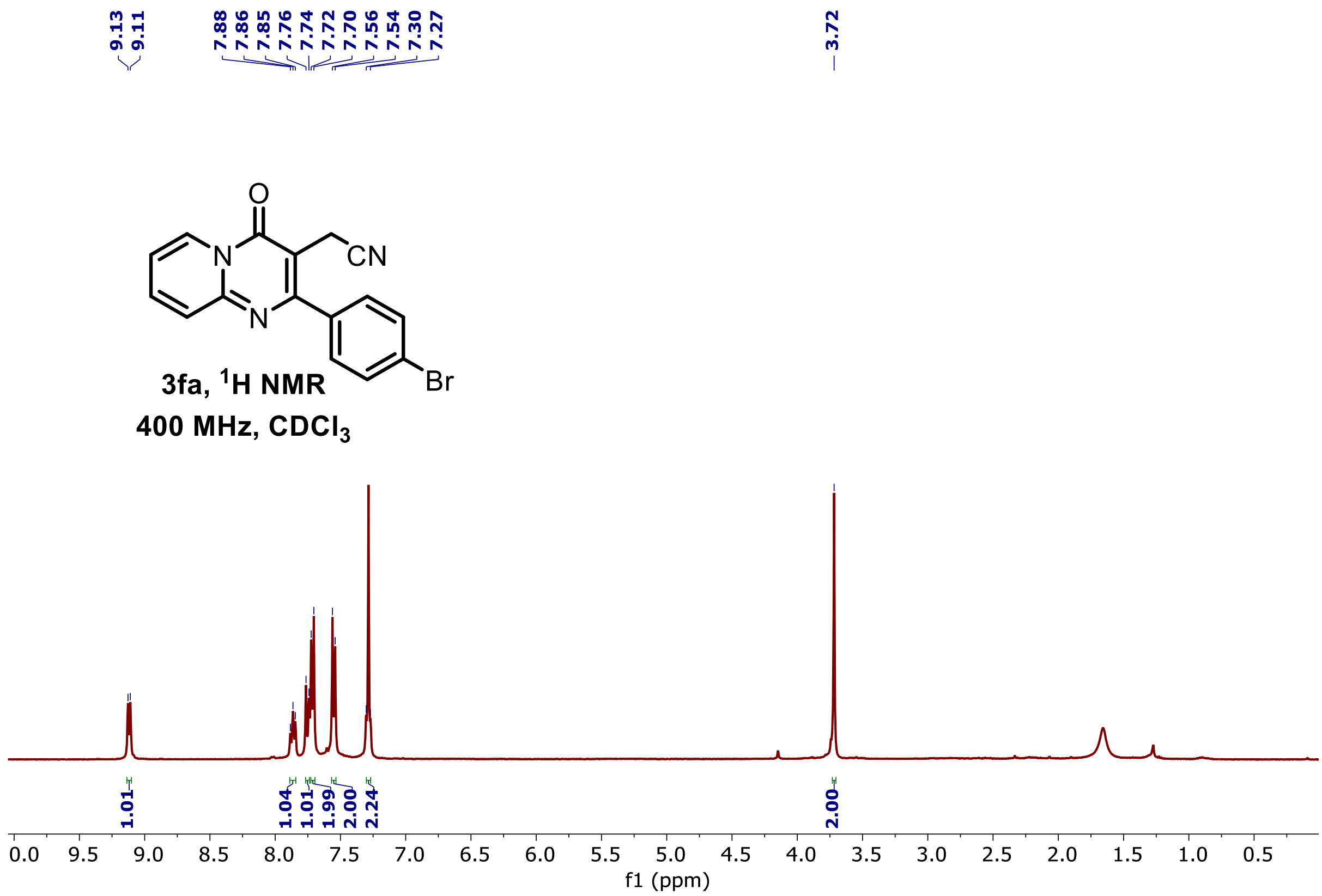
9.13
9.11
7.88
7.86
7.85
7.76
7.74
7.72
7.70
7.56
7.54
7.30
7.27
3.72
O
N
N
CN
Br
3fa, 1H NMR
400 MHz, CDCl3
1.01
1.04
1.01
1.99
2.00
2.24
2.00
f1 (ppm)

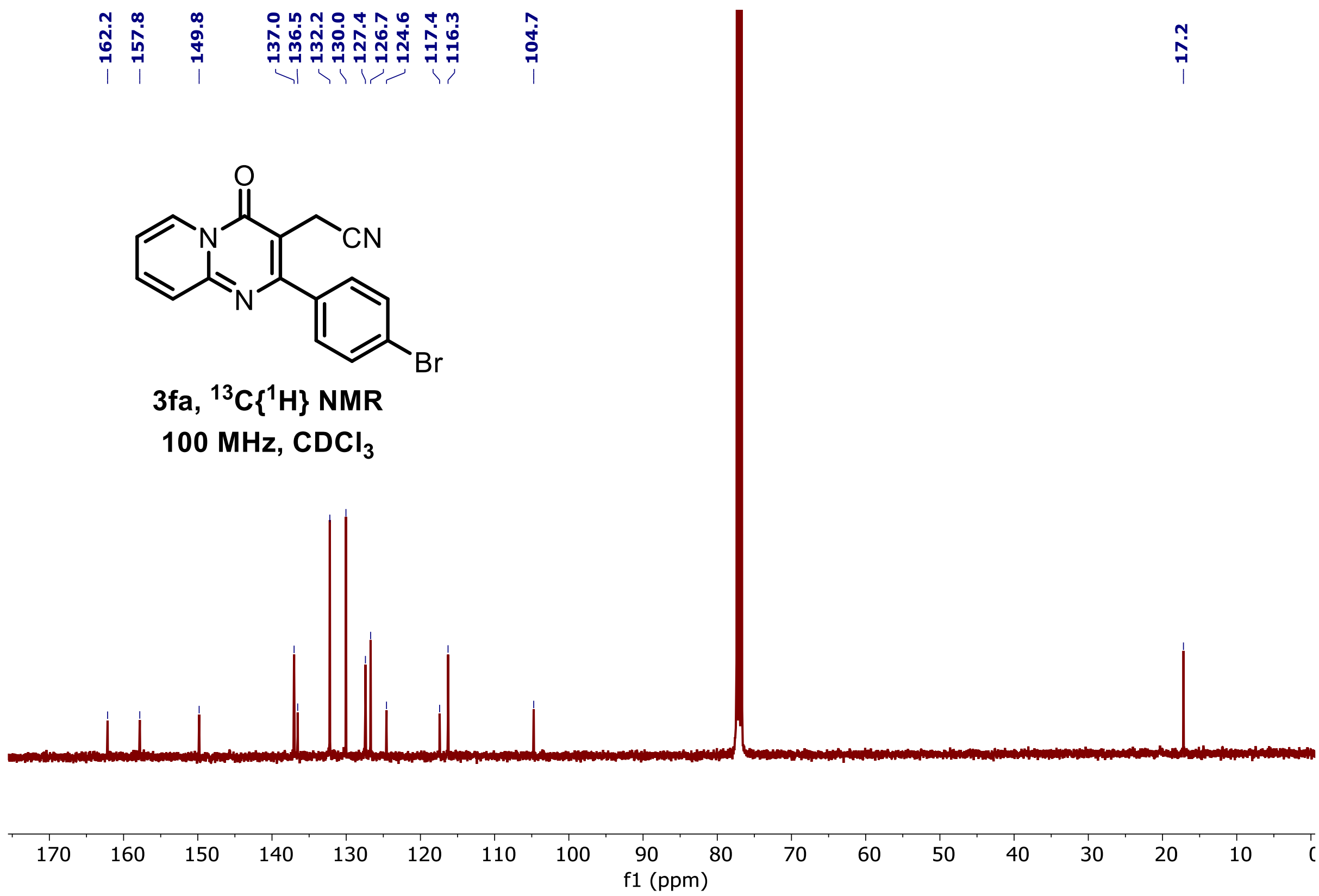
162.2
157.8
149.8
137.0
136.5
132.2
130.0
127.4
126.7
124.6
117.4
116.3
104.7
17.2
O
N
N
CN
Br
3fa, 13C{1H} NMR
100 MHz, CDCl3
f1 (ppm)

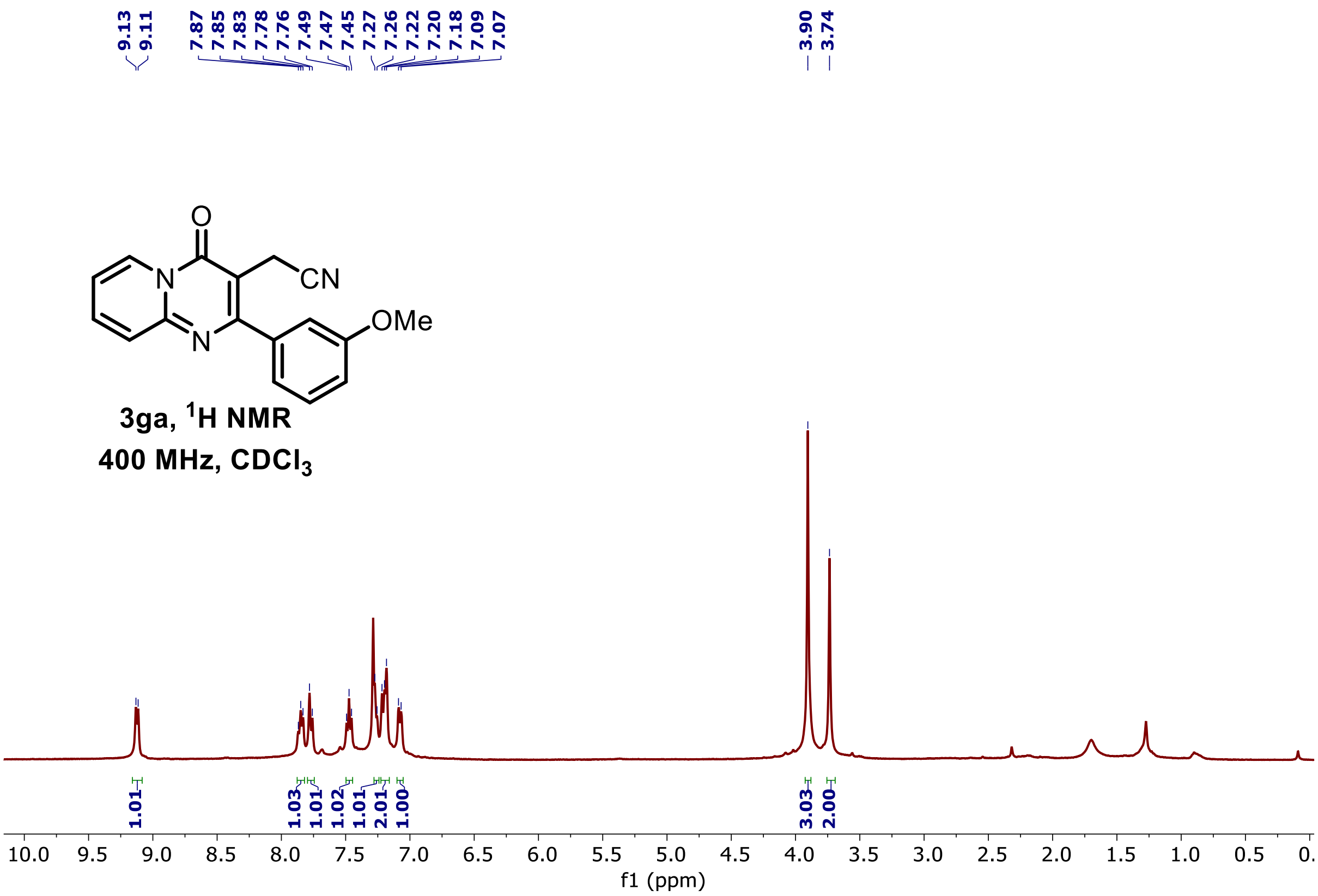
3ga, ^{1}H NMR
400 MHz, $CDCl_3$
f1 (ppm)

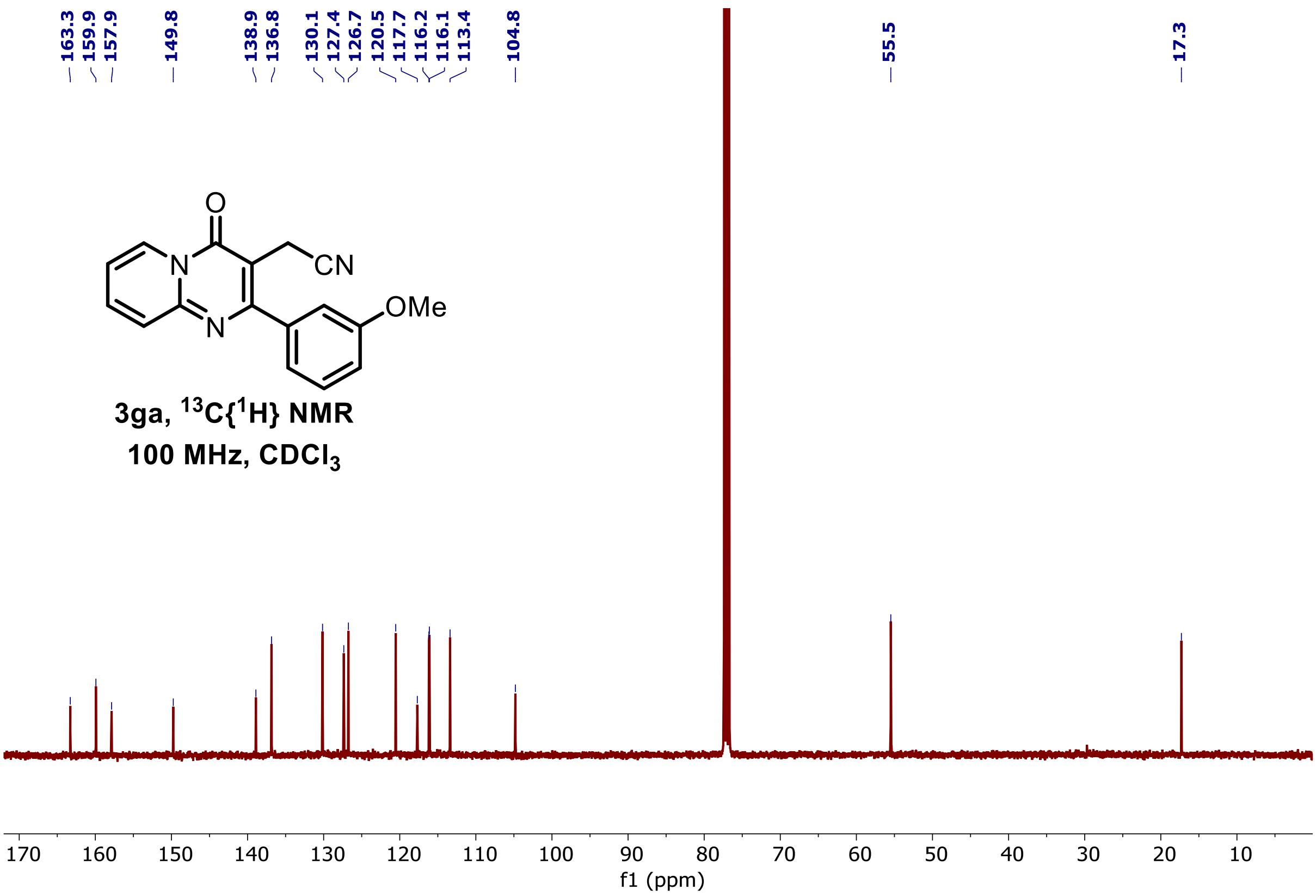
3ga, $^{13}C\{^{1}H\}$ NMR
100 MHz, $CDCl_3$
f1 (ppm)

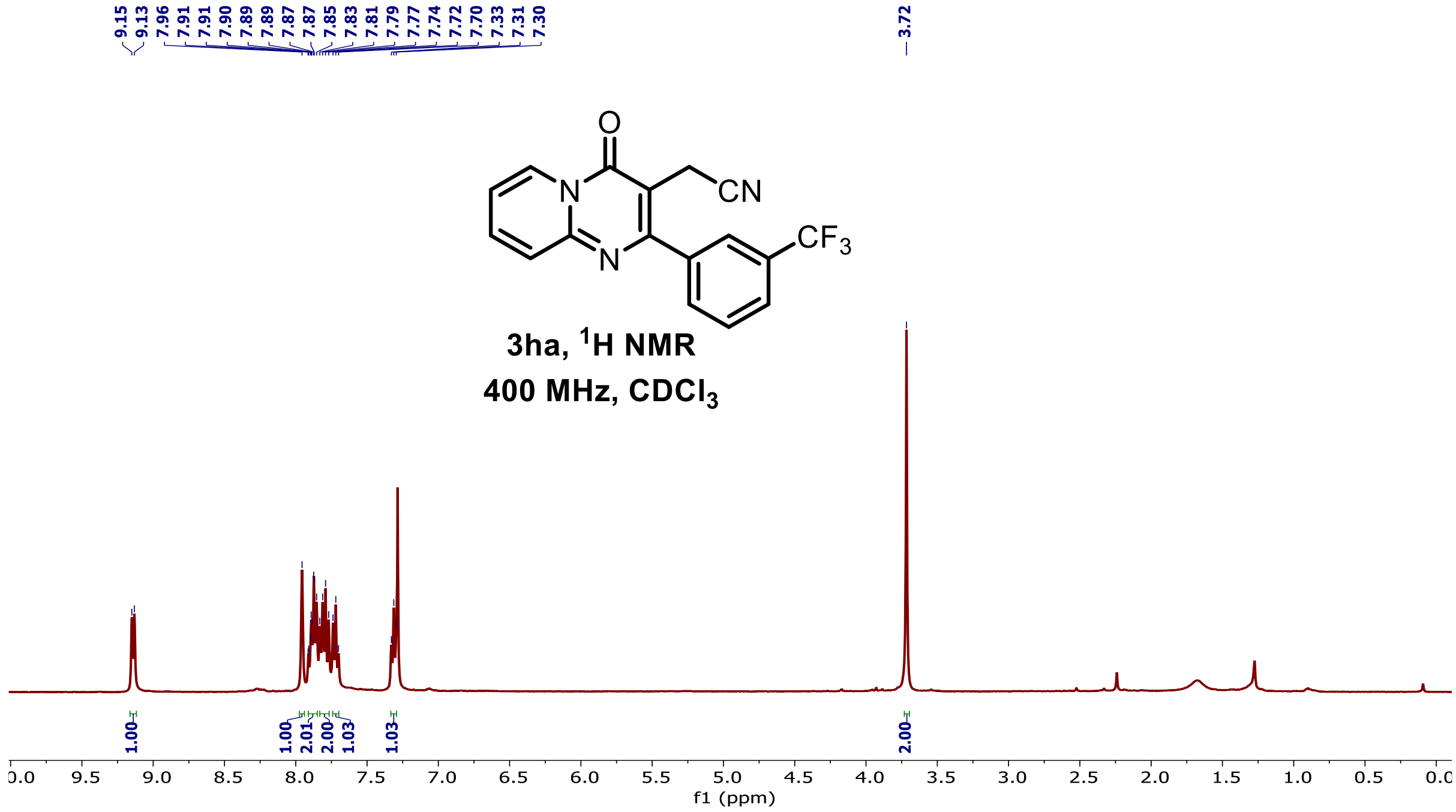
9.15
9.13
7.96
7.91
7.90
7.89
7.89
7.87
7.87
7.85
7.83
7.81
7.79
7.77
7.74
7.72
7.70
7.33
7.31
7.30
3.72
O
N
CN
N
CF3
3ha, 1H NMR
400 MHz, CDCl3
1.00
1.00
2.01
2.00
1.03
1.03
2.00
f1 (ppm)

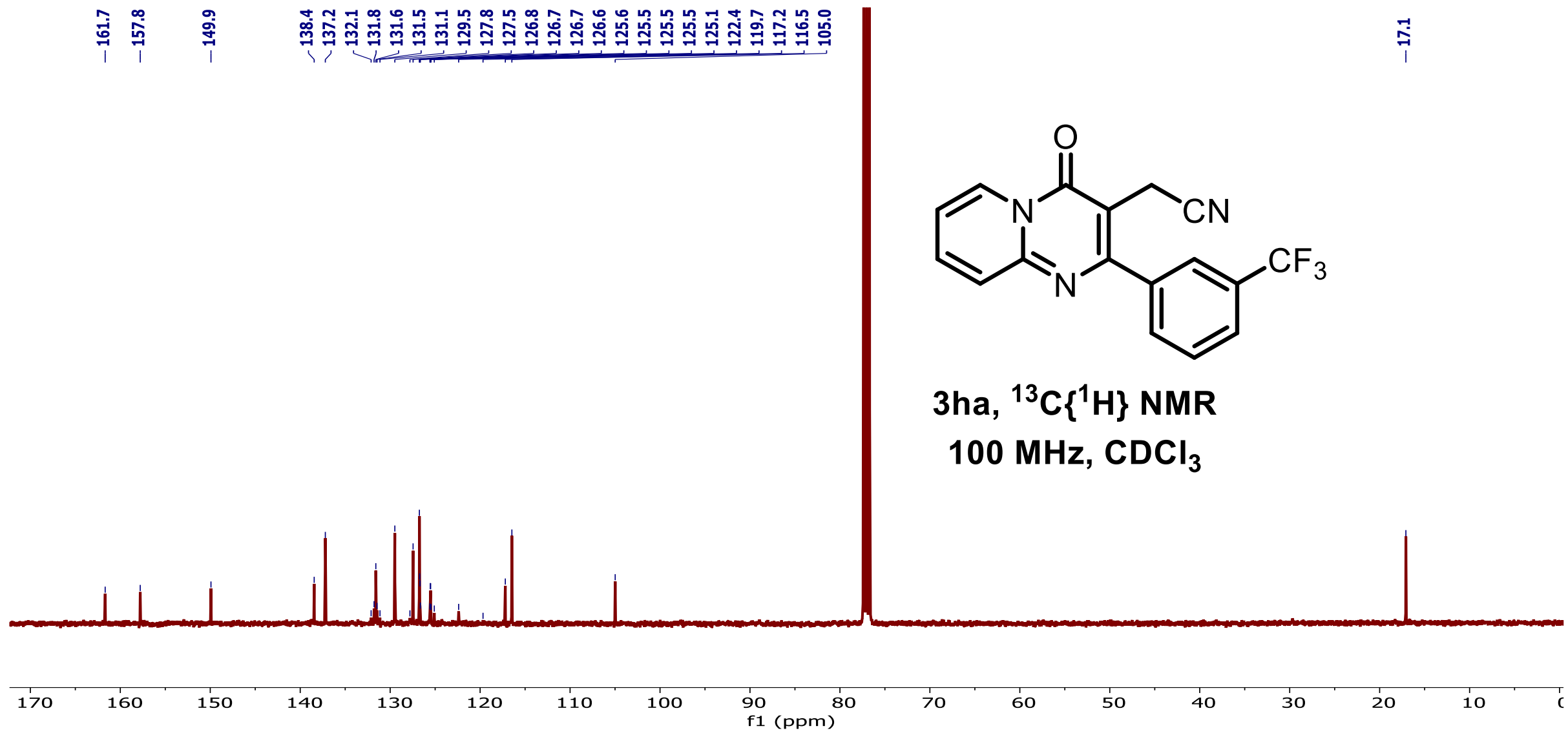
161.7
157.8
149.9
138.4
137.2
132.1
131.8
131.6
131.5
131.1
129.5
127.8
127.5
126.8
126.7
126.7
126.6
125.6
125.5
125.5
125.5
125.1
122.4
119.7
117.2
116.5
105.0
17.1
O
N
CN
N
CF3
3ha, 13C{1H} NMR
100 MHz, CDCl3
f1 (ppm)

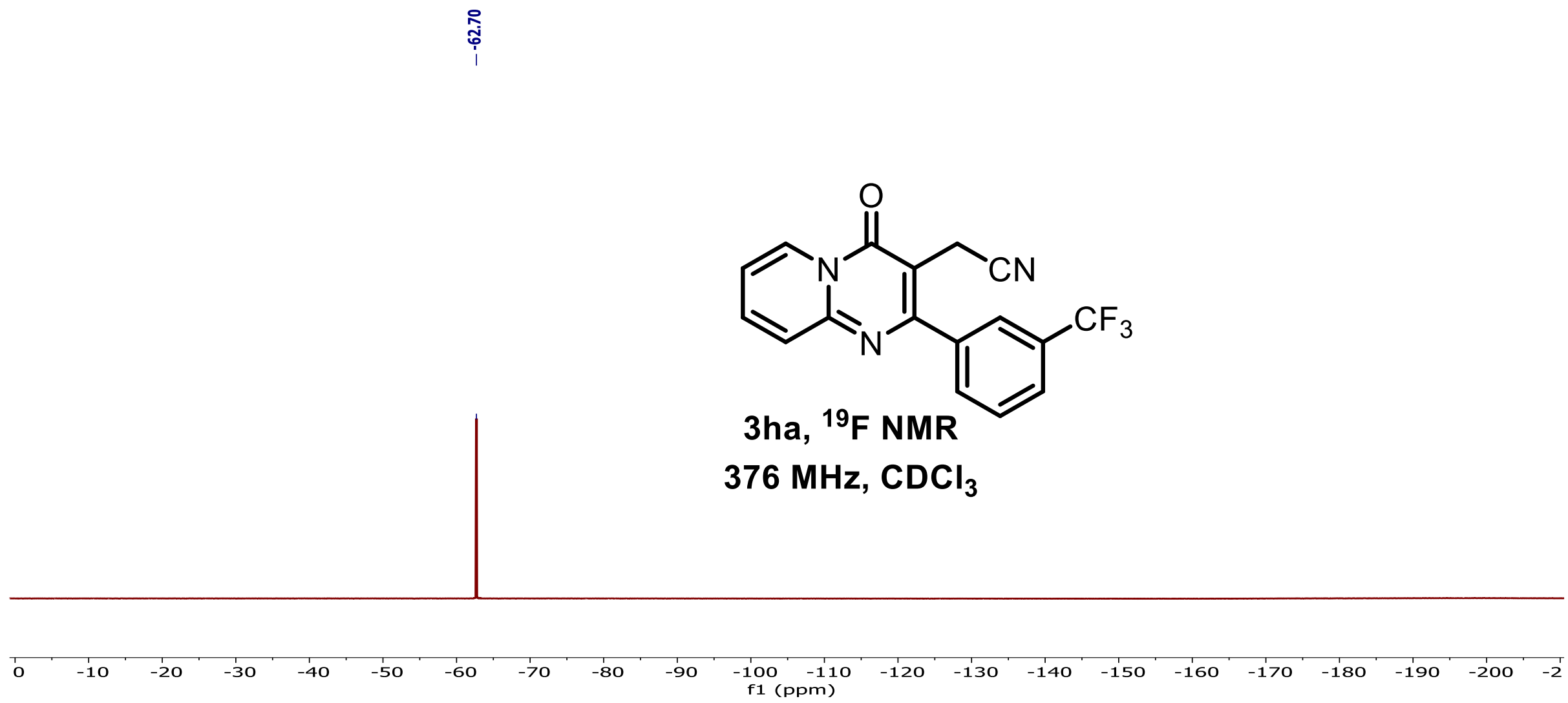
-62.70
O
N
CN
N
CF3
3ha, 19F NMR
376 MHz, CDCl3
f1 (ppm)

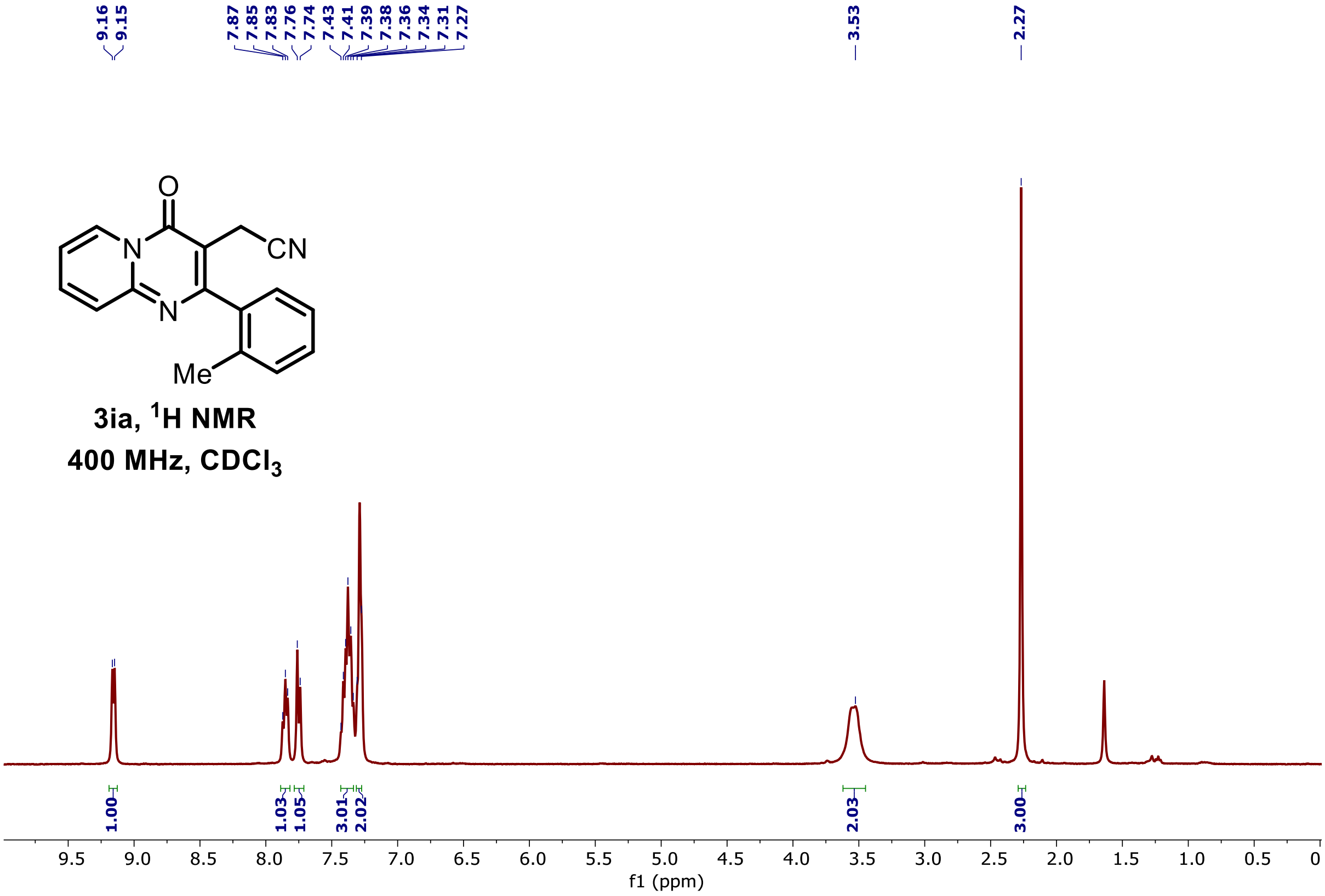
9.16
9.15
7.87
7.85
7.83
7.76
7.74
7.43
7.41
7.39
7.38
7.36
7.34
7.31
7.27
3.53
2.27
O
N
CN
N
Me
3ia, 1H NMR
400 MHz, CDCl3
1.00
1.03
1.05
3.01
2.02
2.03
3.00
9.5
9.0
8.5
8.0
7.5
7.0
6.5
6.0
5.5
5.0
4.5
4.0
3.5
3.0
2.5
2.0
1.5
1.0
0.5
0
f1 (ppm)

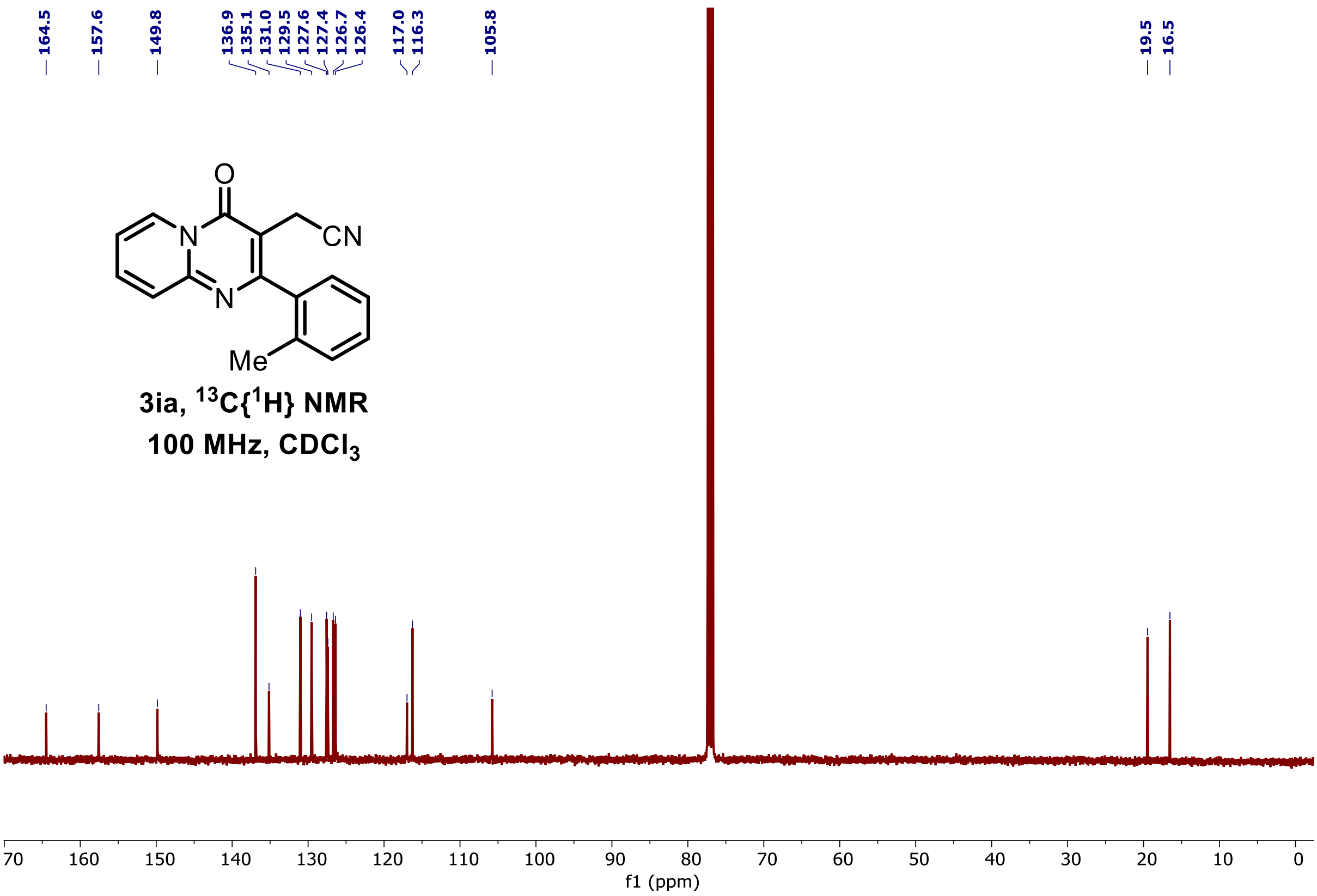
164.5
157.6
149.8
136.9
135.1
131.0
129.5
127.6
127.4
126.7
126.4
117.0
116.3
105.8
19.5
16.5
O
N
CN
N
Me
3ia, 13C{1H} NMR
100 MHz, CDCl3
70
160
150
140
130
120
110
100
90
80
70
60
50
40
30
20
10
0
f1 (ppm)

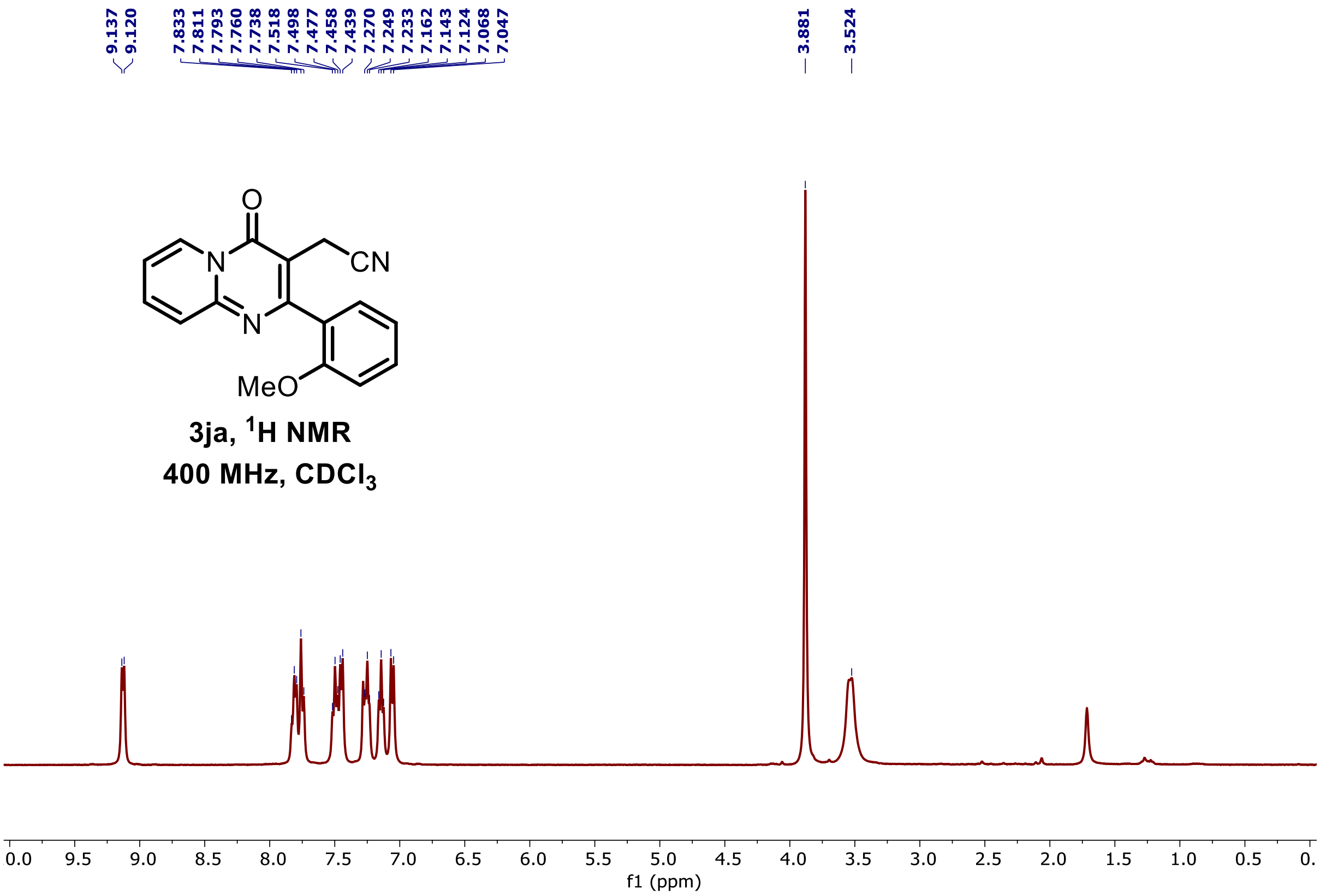
9.137
9.120
7.833
7.811
7.793
7.760
7.738
7.518
7.498
7.477
7.458
7.439
7.270
7.249
7.233
7.162
7.143
7.124
7.068
7.047
3.881
3.524
O
N
CN
N
MeO
3ja, ¹H NMR
400 MHz, CDCl₃
f1 (ppm)

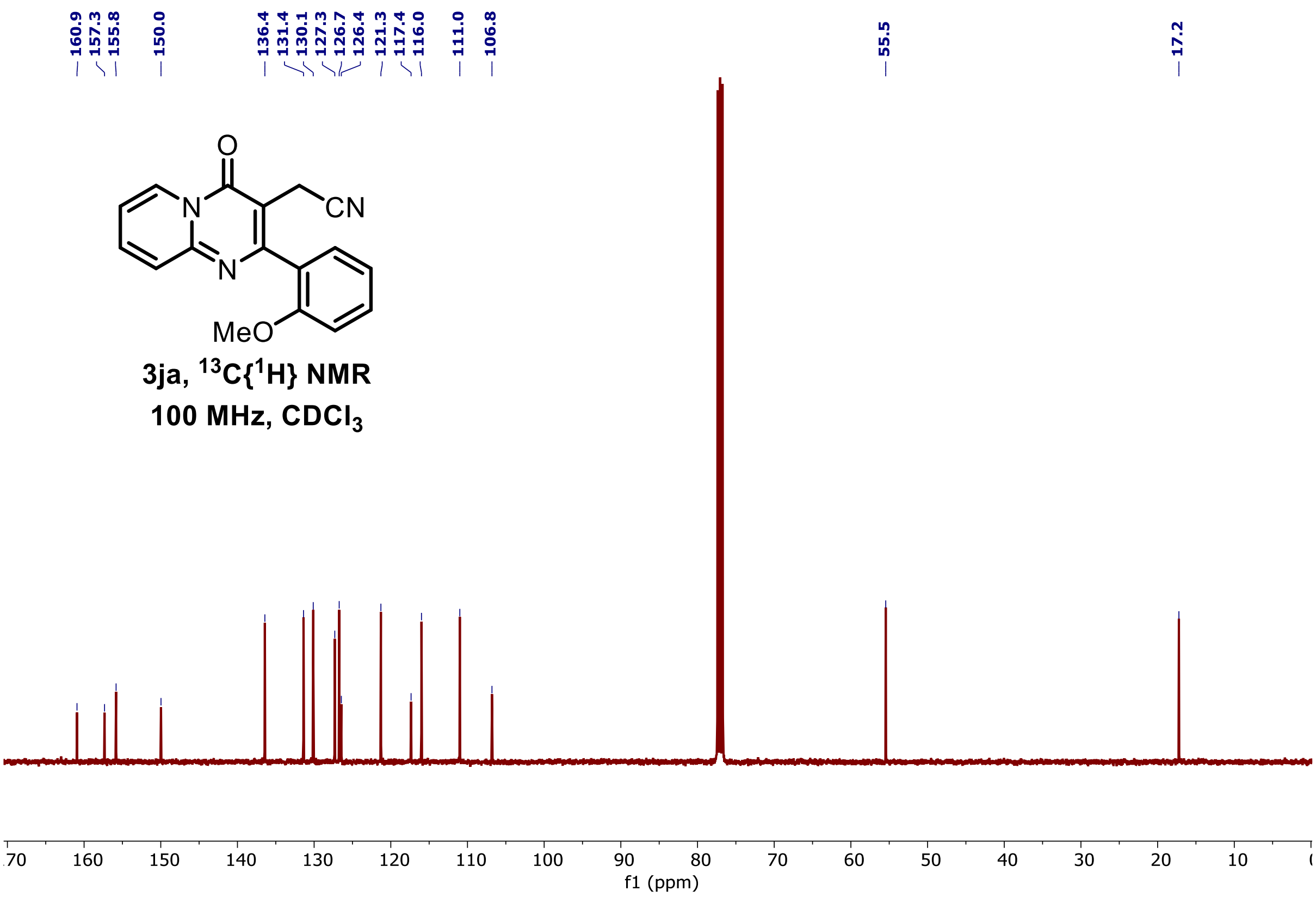
160.9
157.3
155.8
150.0
136.4
131.4
130.1
127.3
126.7
126.4
121.3
117.4
116.0
111.0
106.8
55.5
17.2
O
N
CN
N
MeO
3ja, ¹³C{¹H} NMR
100 MHz, CDCl₃
f1 (ppm)

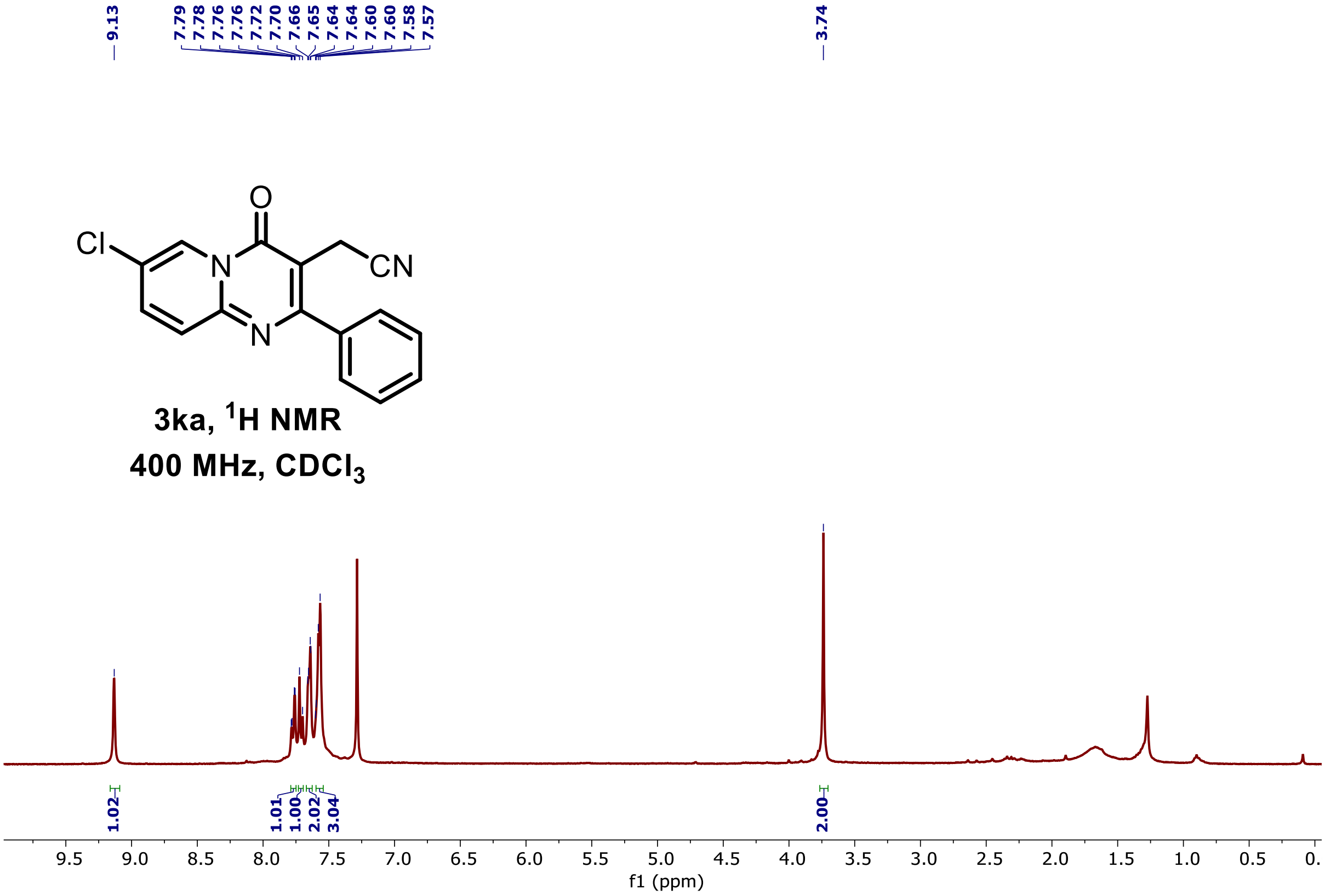
3ka, 1H NMR
400 MHz, CDCl3
f1 (ppm)

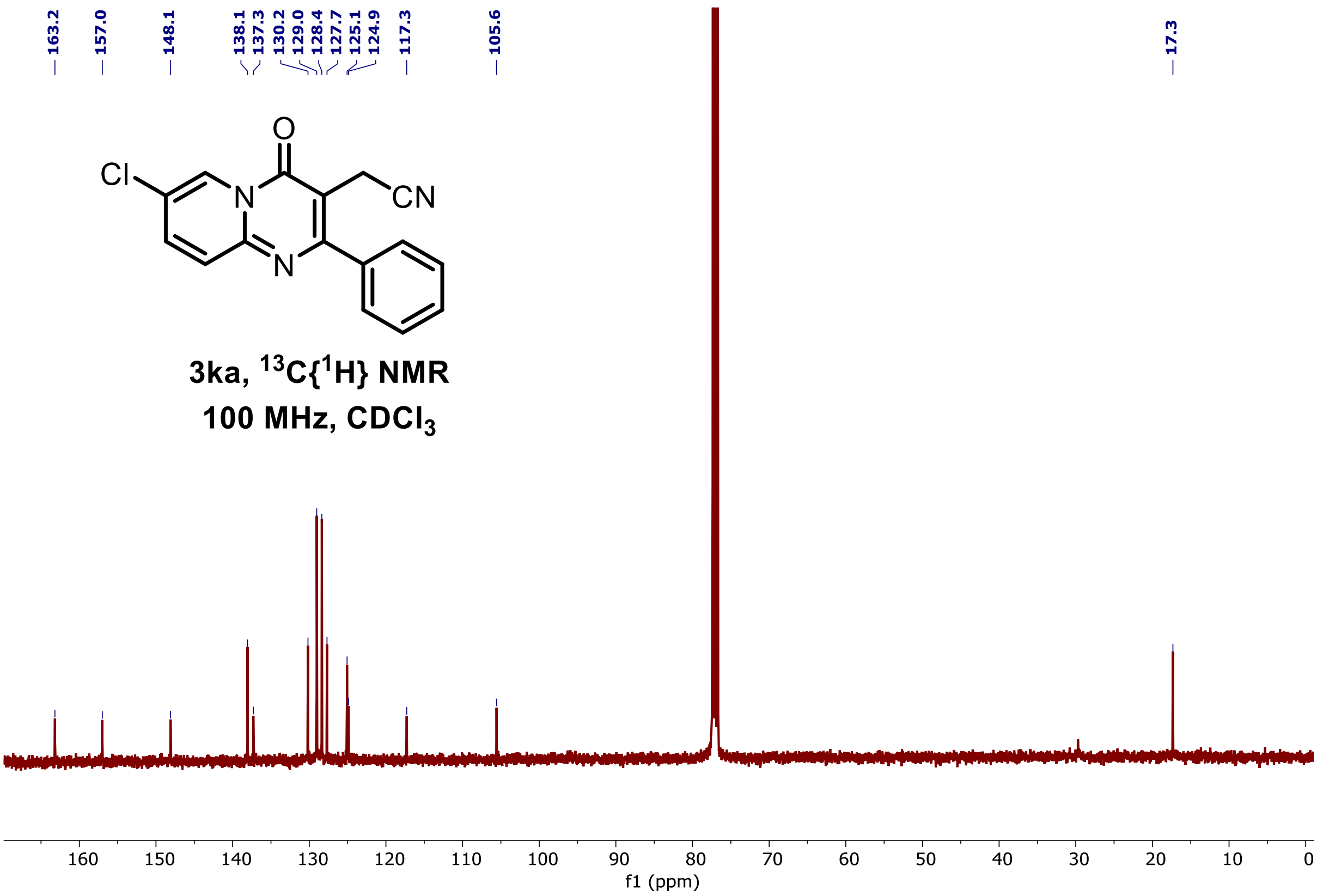
3ka, 13C{1H} NMR
100 MHz, CDCl3
f1 (ppm)

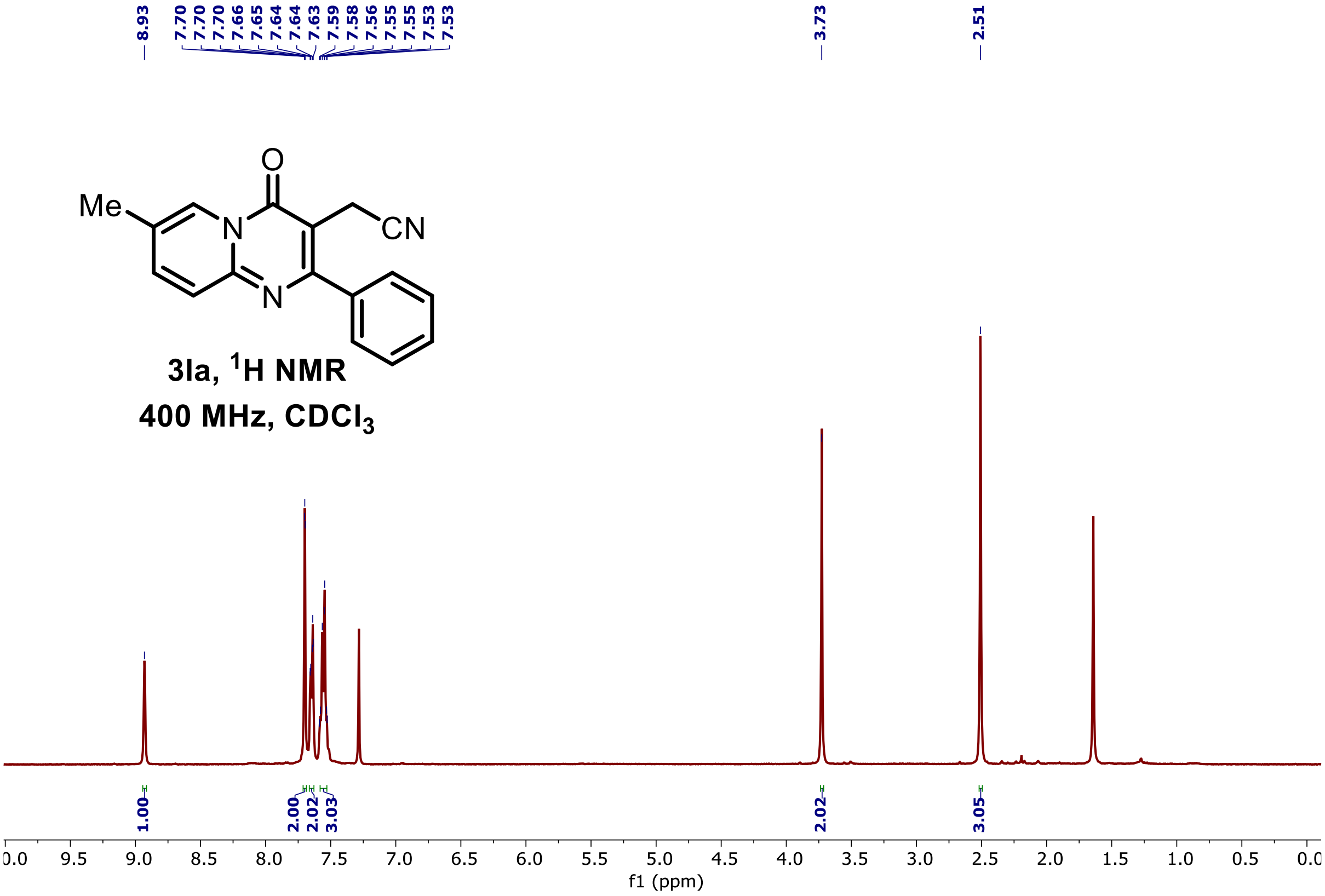
8.93
7.70
7.70
7.70
7.66
7.65
7.64
7.64
7.63
7.59
7.58
7.56
7.55
7.55
7.53
7.53
3.73
2.51
Me
O
N
CN
N
3la, ¹H NMR
400 MHz, CDCl3
1.00
2.00
2.02
3.03
2.02
3.05
f1 (ppm)

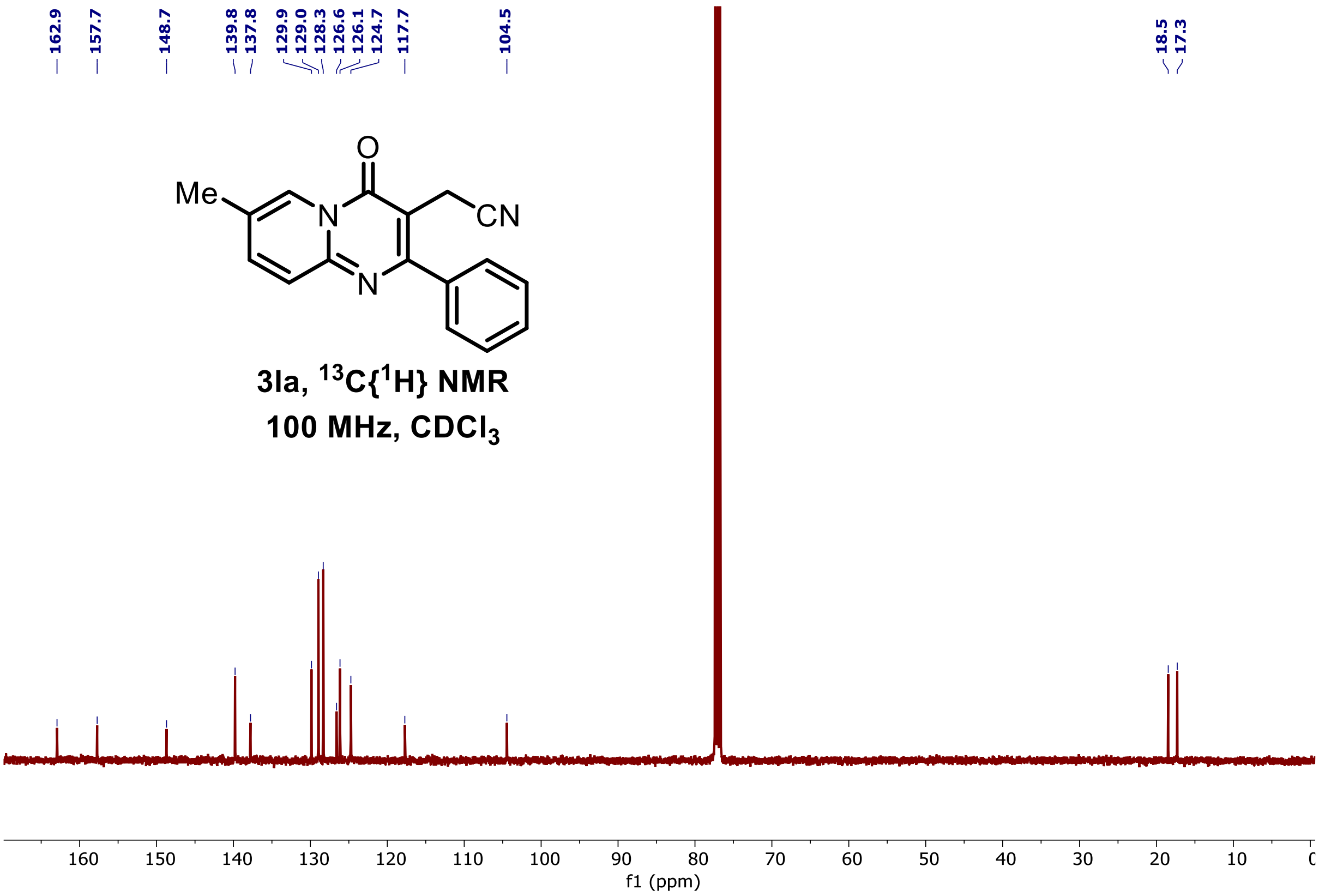
162.9
157.7
148.7
139.8
137.8
129.9
129.0
128.3
126.6
126.1
124.7
117.7
104.5
18.5
17.3
Me
O
N
CN
N
3la, ¹³C{¹H} NMR
100 MHz, CDCl3
f1 (ppm)

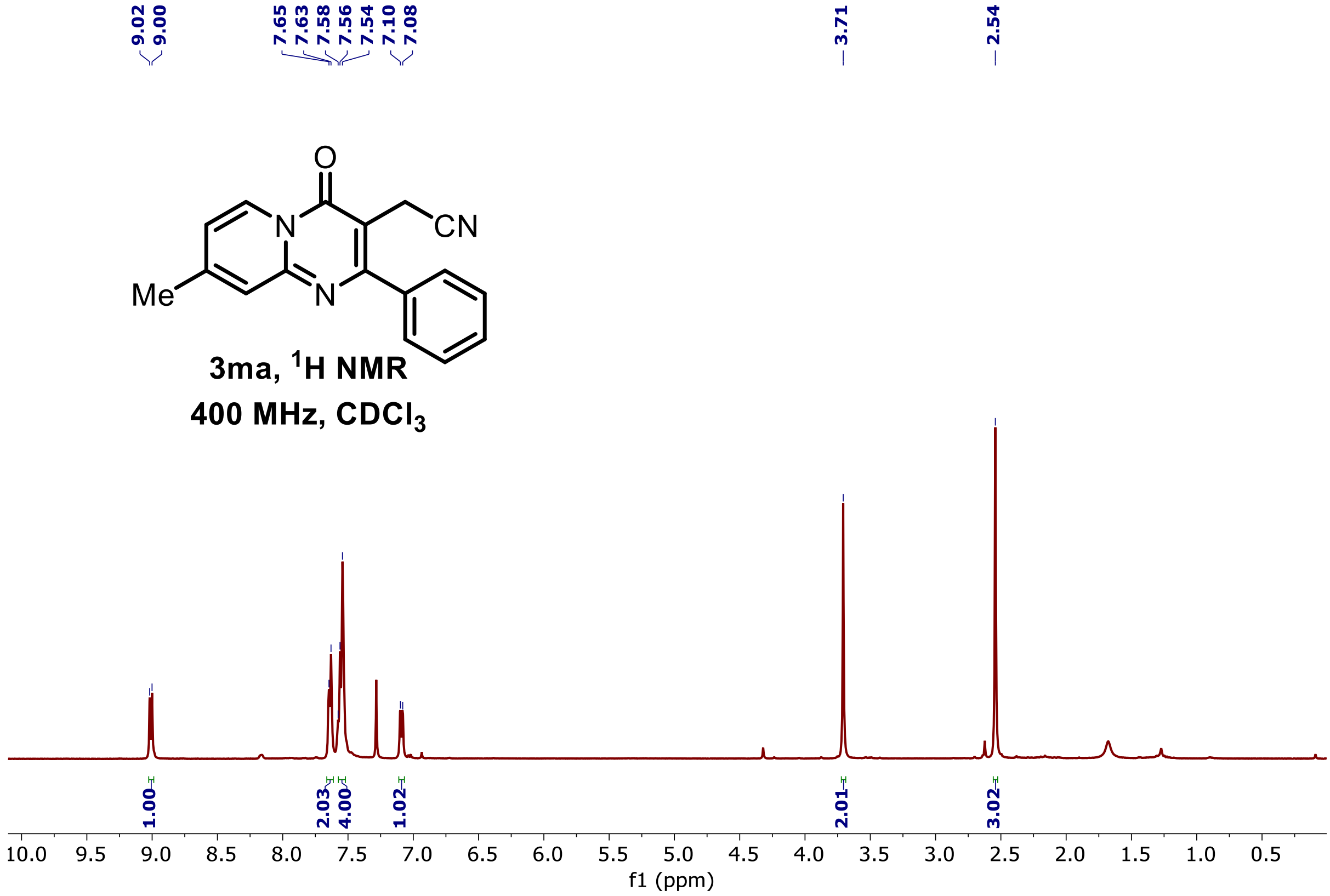
9.02
9.00
7.65
7.63
7.58
7.56
7.54
7.10
7.08
3.71
2.54
O
N
CN
Me
N
3ma, 1H NMR
400 MHz, CDCl3
1.00
2.03
4.00
1.02
2.01
3.02
10.0 9.5 9.0 8.5 8.0 7.5 7.0 6.5 6.0 5.5 5.0 4.5 4.0 3.5 3.0 2.5 2.0 1.5 1.0 0.5
f1 (ppm)

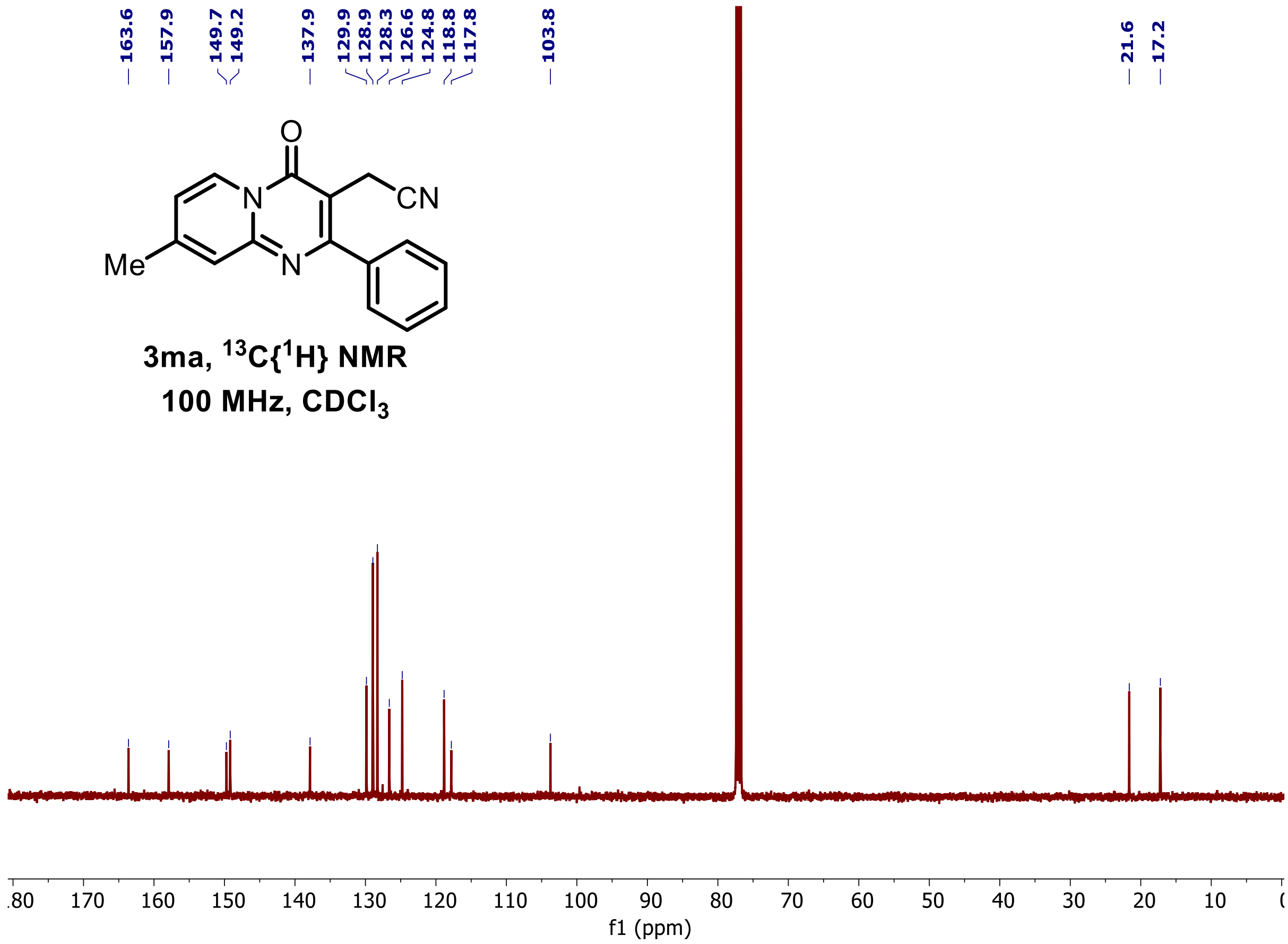
163.6
157.9
149.7
149.2
137.9
129.9
128.9
128.3
126.6
124.8
118.8
117.8
103.8
21.6
17.2
O
N
CN
Me
N
3ma, 13C{1H} NMR
100 MHz, CDCl3
180 170 160 150 140 130 120 110 100 90 80 70 60 50 40 30 20 10 0
f1 (ppm)

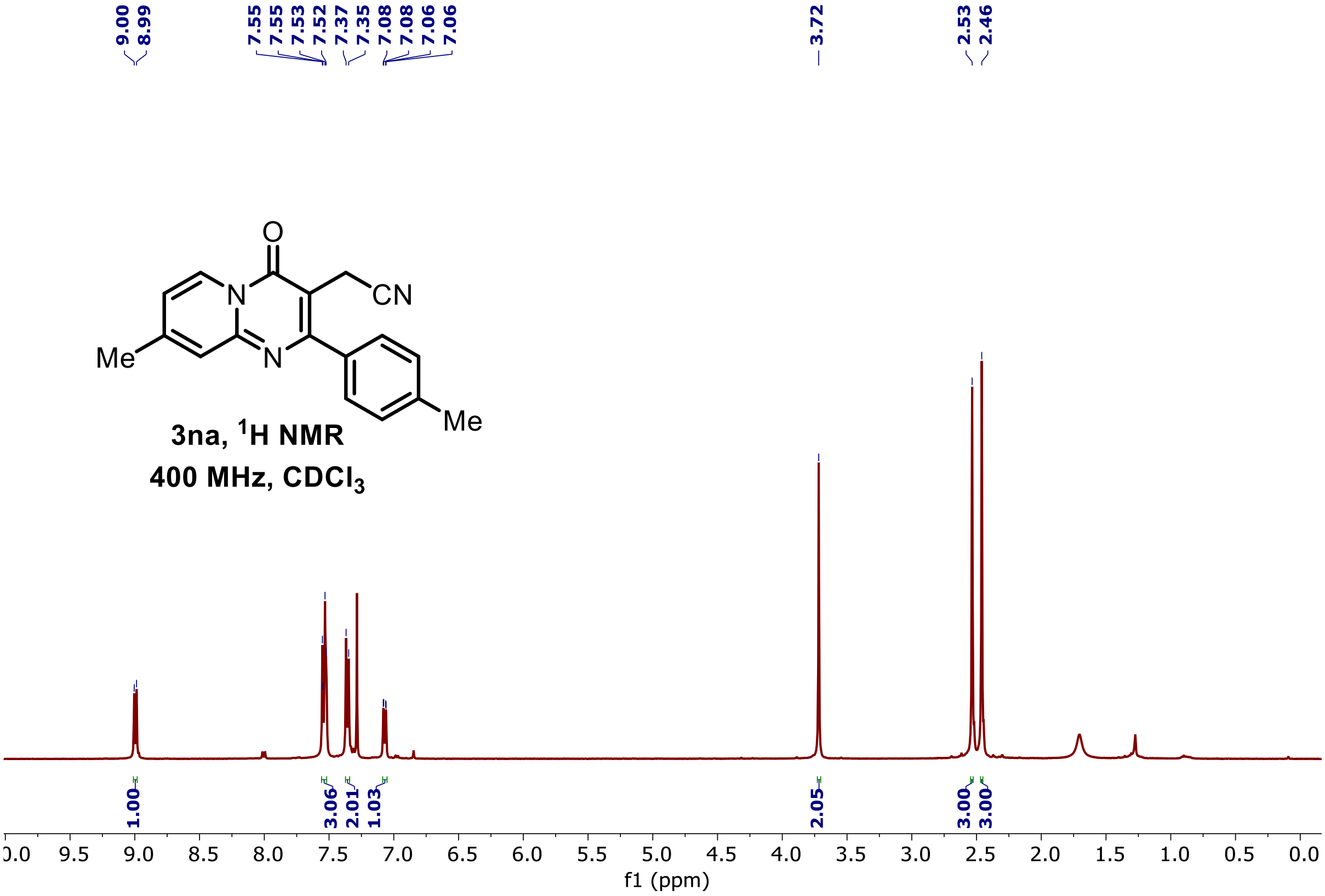
9.00
8.99
7.55
7.55
7.53
7.52
7.37
7.35
7.08
7.08
7.06
7.06
3.72
2.53
2.46
O
N
CN
Me
N
Me
3na, ¹H NMR
400 MHz, CDCl3
1.00
3.06
2.01
1.03
2.05
3.00
3.00
f1 (ppm)

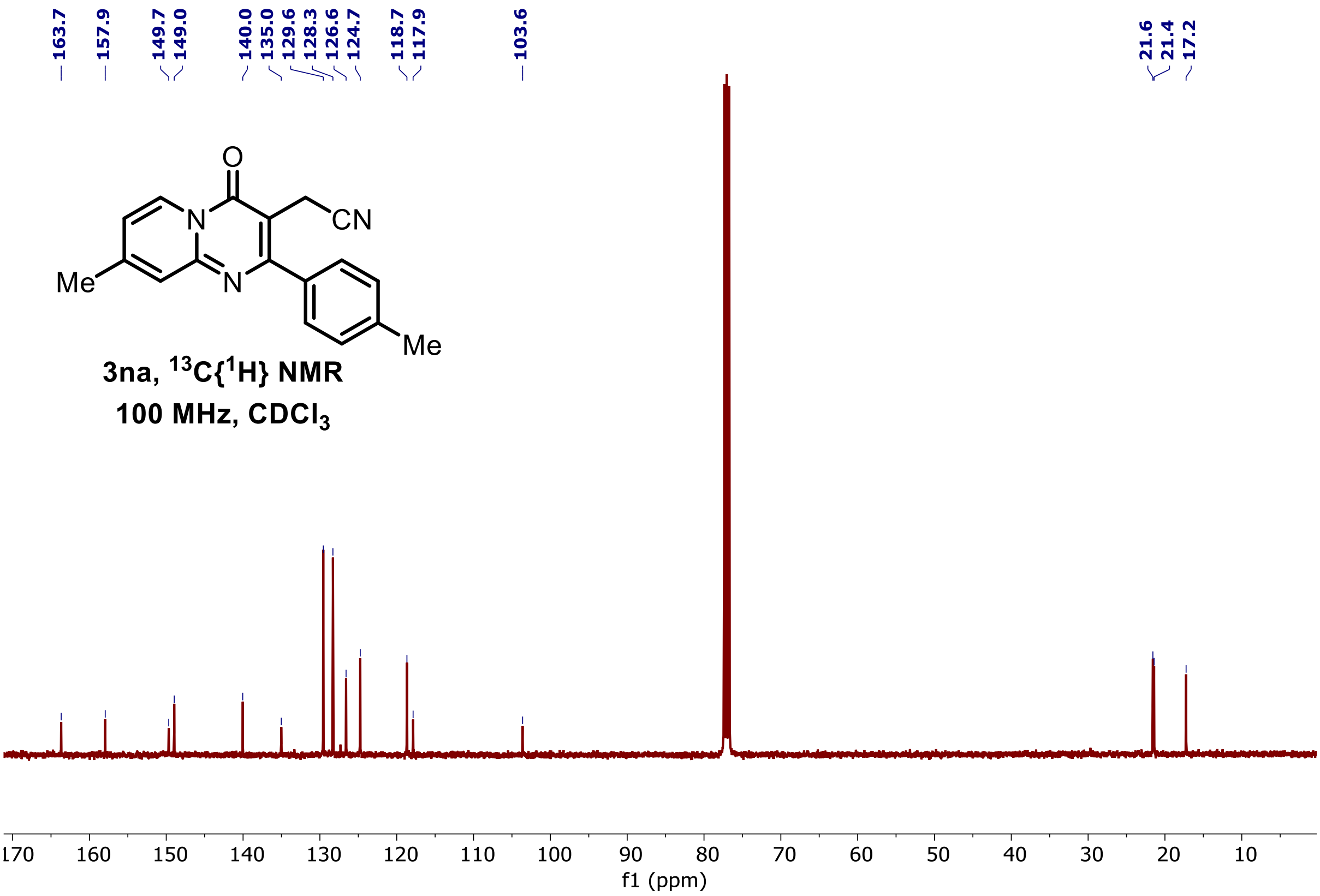
163.7
157.9
149.7
149.0
140.0
135.0
129.6
128.3
126.6
124.7
118.7
117.9
103.6
21.6
21.4
17.2
O
N
CN
Me
N
Me
3na, ¹³C{¹H} NMR
100 MHz, CDCl3
f1 (ppm)

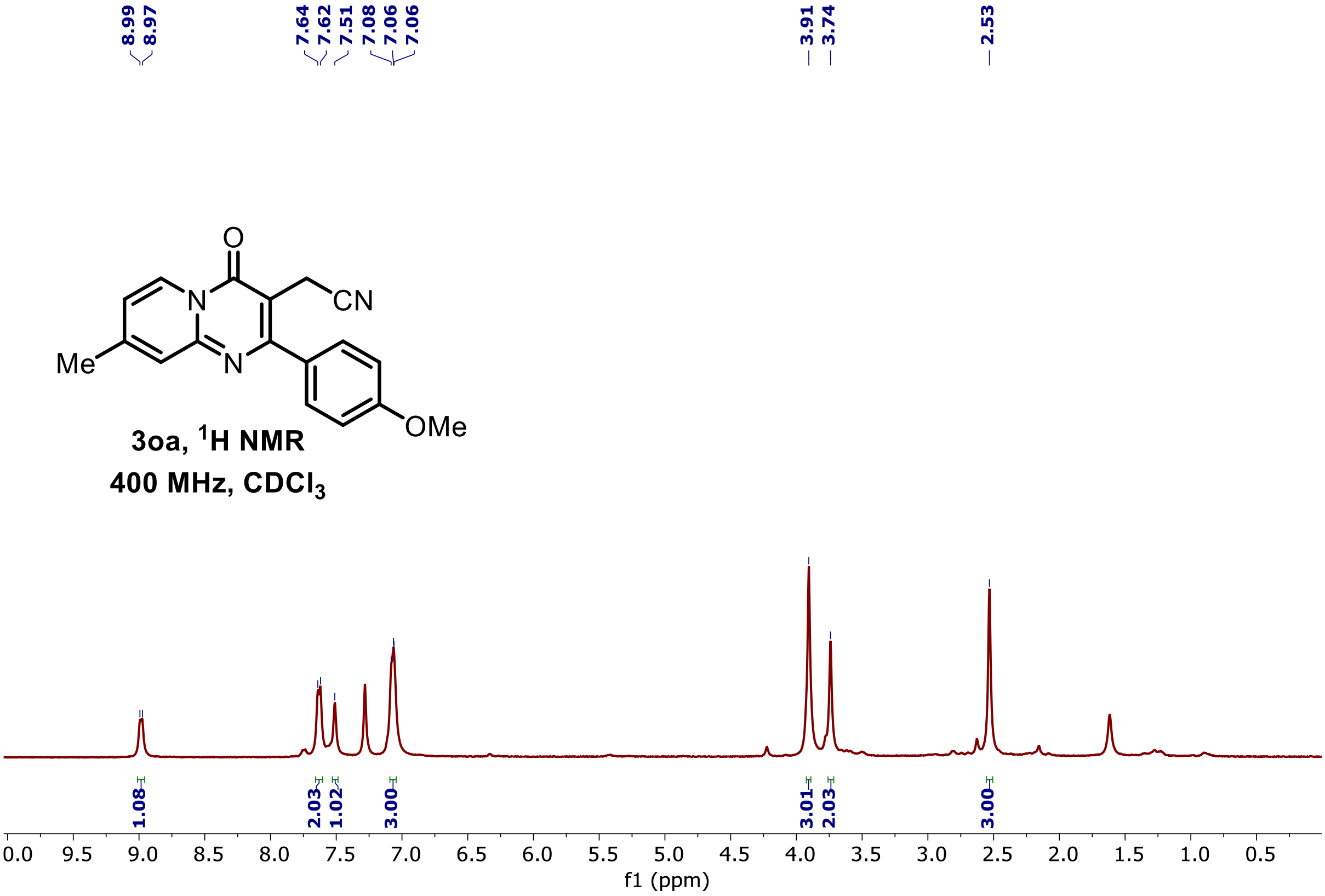
8.99
8.97
7.64
7.62
7.51
7.08
7.06
7.06
3.91
3.74
2.53
O
N
CN
Me
N
OMe
3oa, 1H NMR
400 MHz, CDCl3
1.08
2.03
1.02
3.00
3.01
2.03
3.00
f1 (ppm)

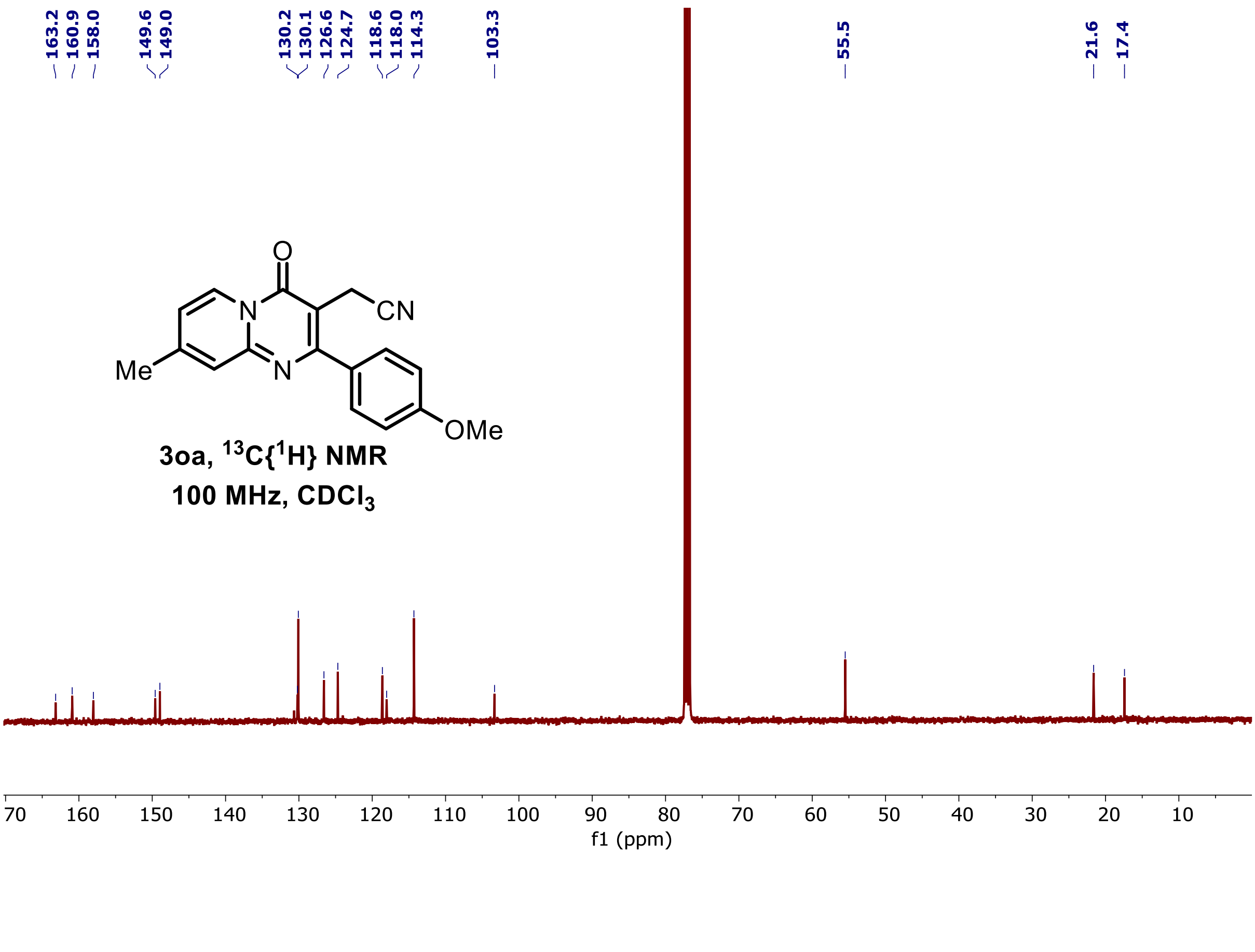
163.2
160.9
158.0
149.6
149.0
130.2
130.1
126.6
124.7
118.6
118.0
114.3
103.3
55.5
21.6
17.4
O
N
CN
Me
N
OMe
3oa, 13C{1H} NMR
100 MHz, CDCl3
f1 (ppm)

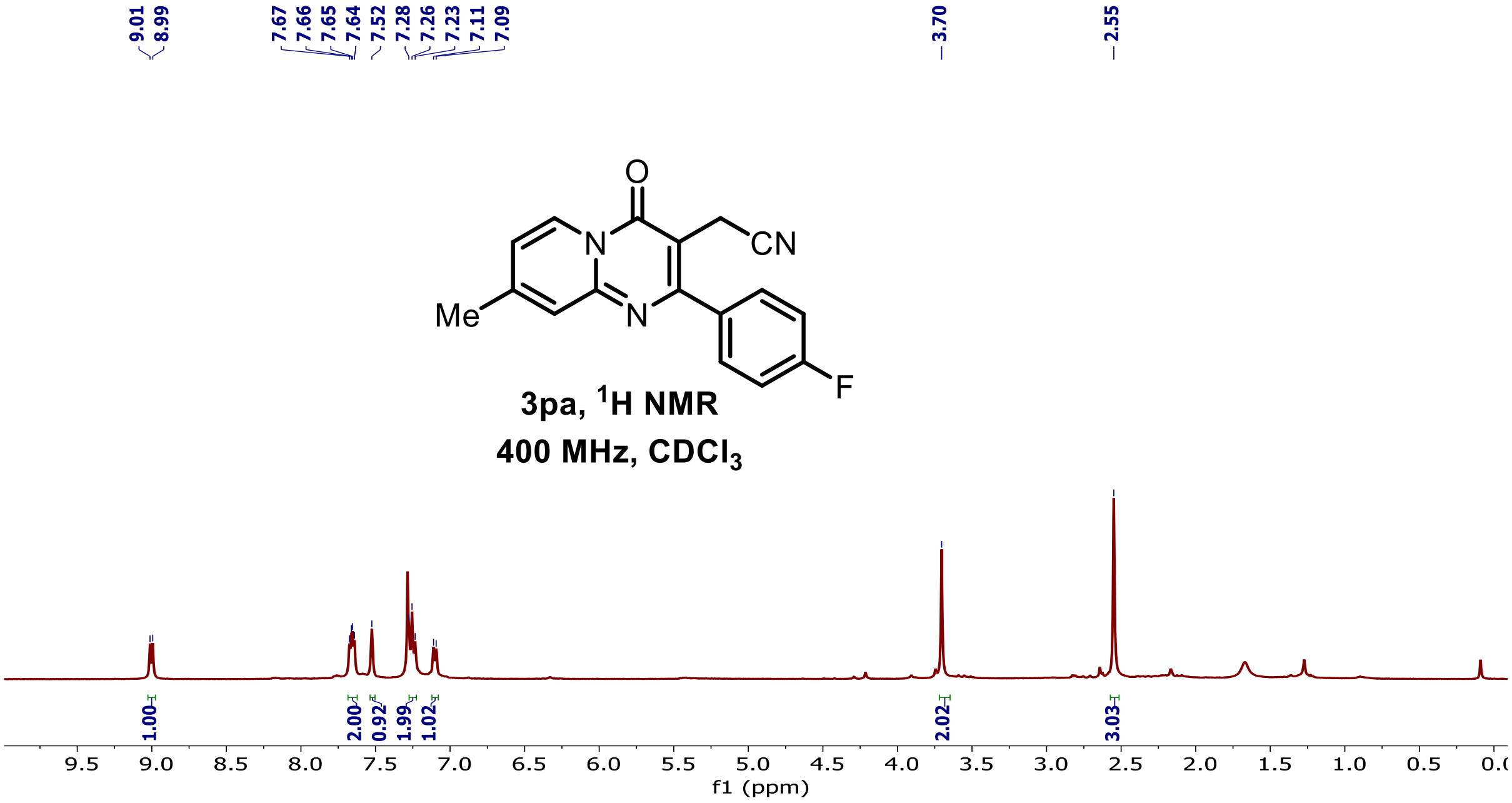

3pa, ^{1}H NMR
400 MHz, $CDCl_3$

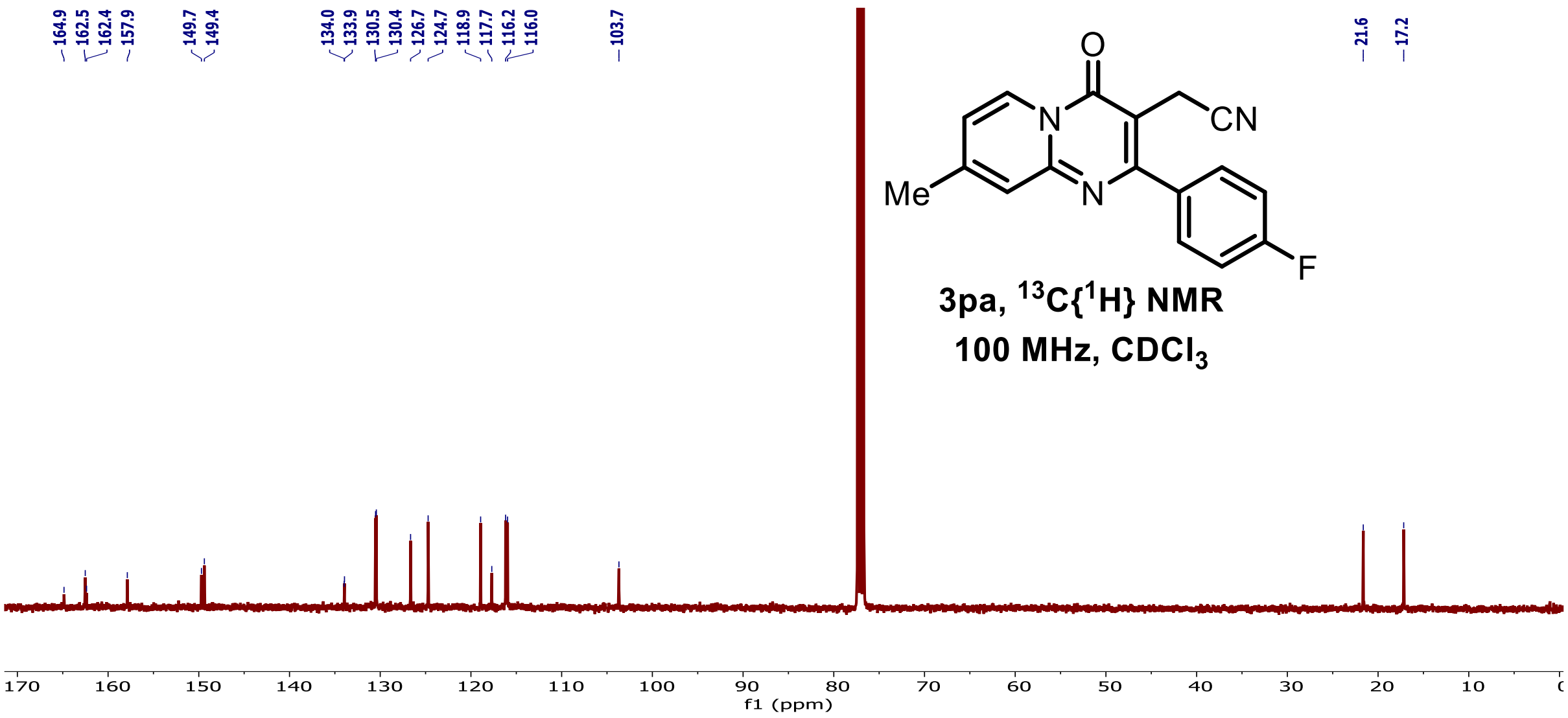

3pa, $^{13}C\{^{1}H\}$ NMR
100 MHz, $CDCl_3$

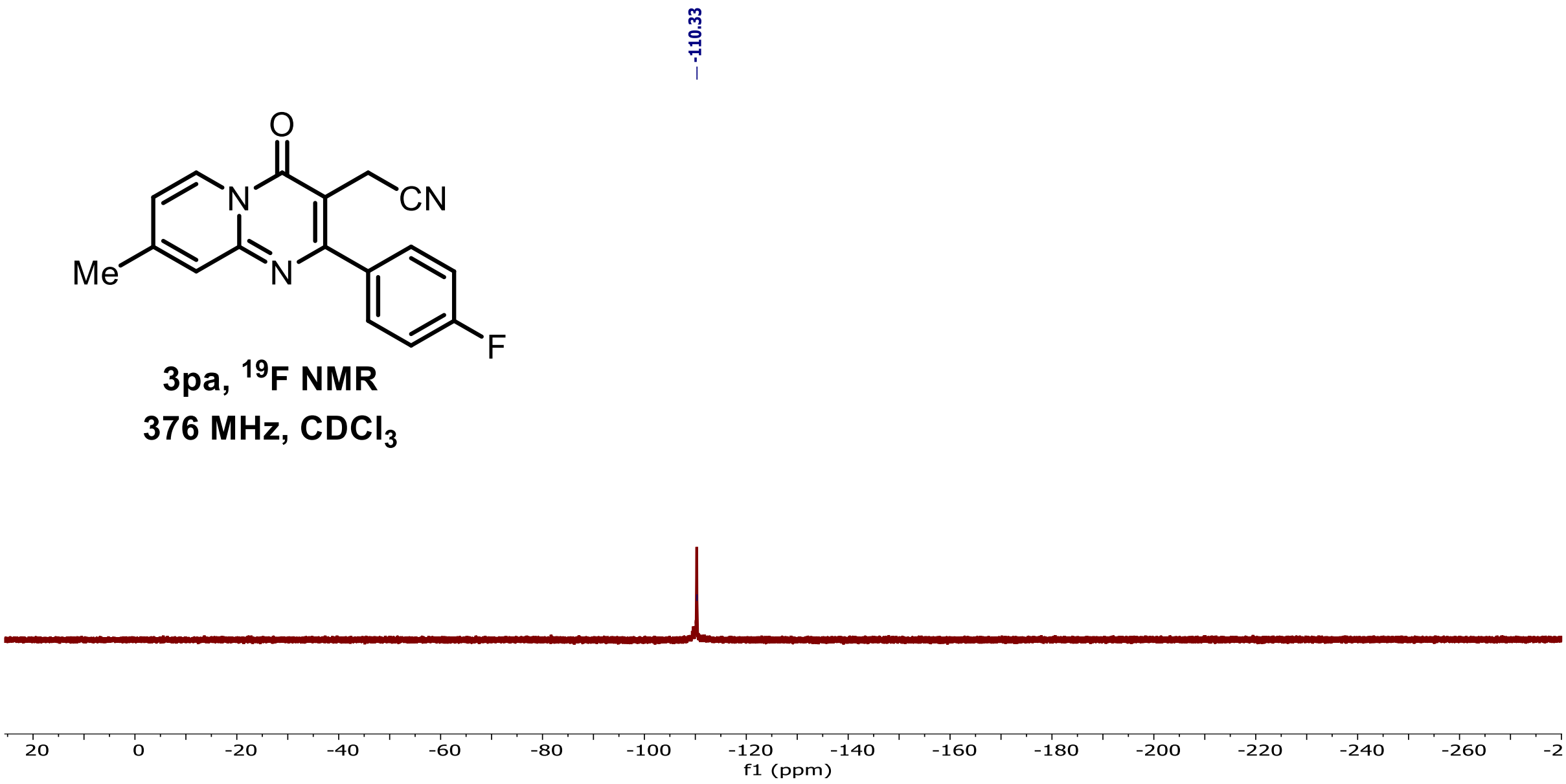

3pa, ^{19}F NMR
376 MHz, $CDCl_3$

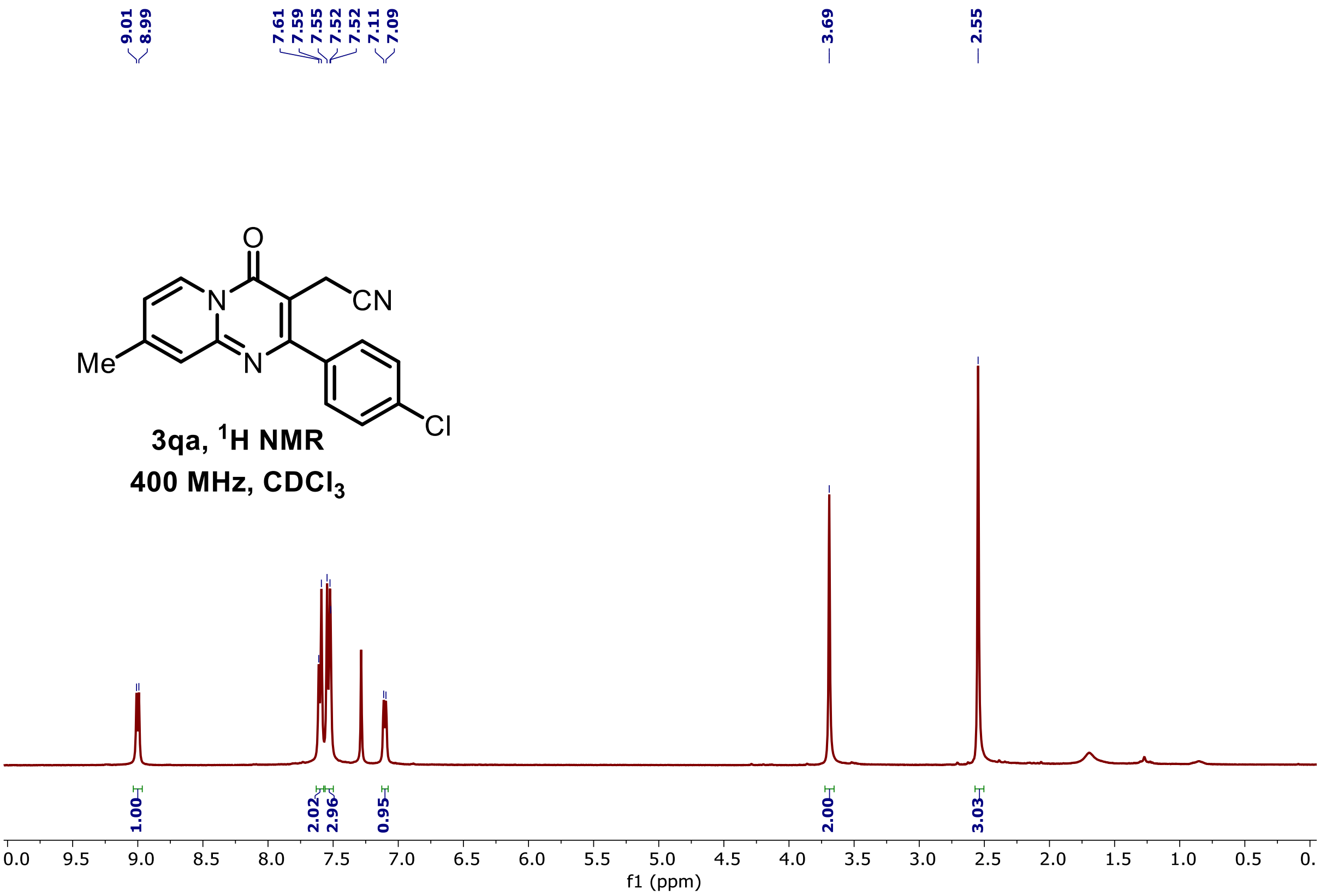
9.01
8.99
7.61
7.59
7.55
7.52
7.52
7.11
7.09
3.69
2.55
O
N
CN
Me
N
Cl
3qa, ¹H NMR
400 MHz, CDCl₃
1.00
2.02
2.96
0.95
2.00
3.03
f1 (ppm)

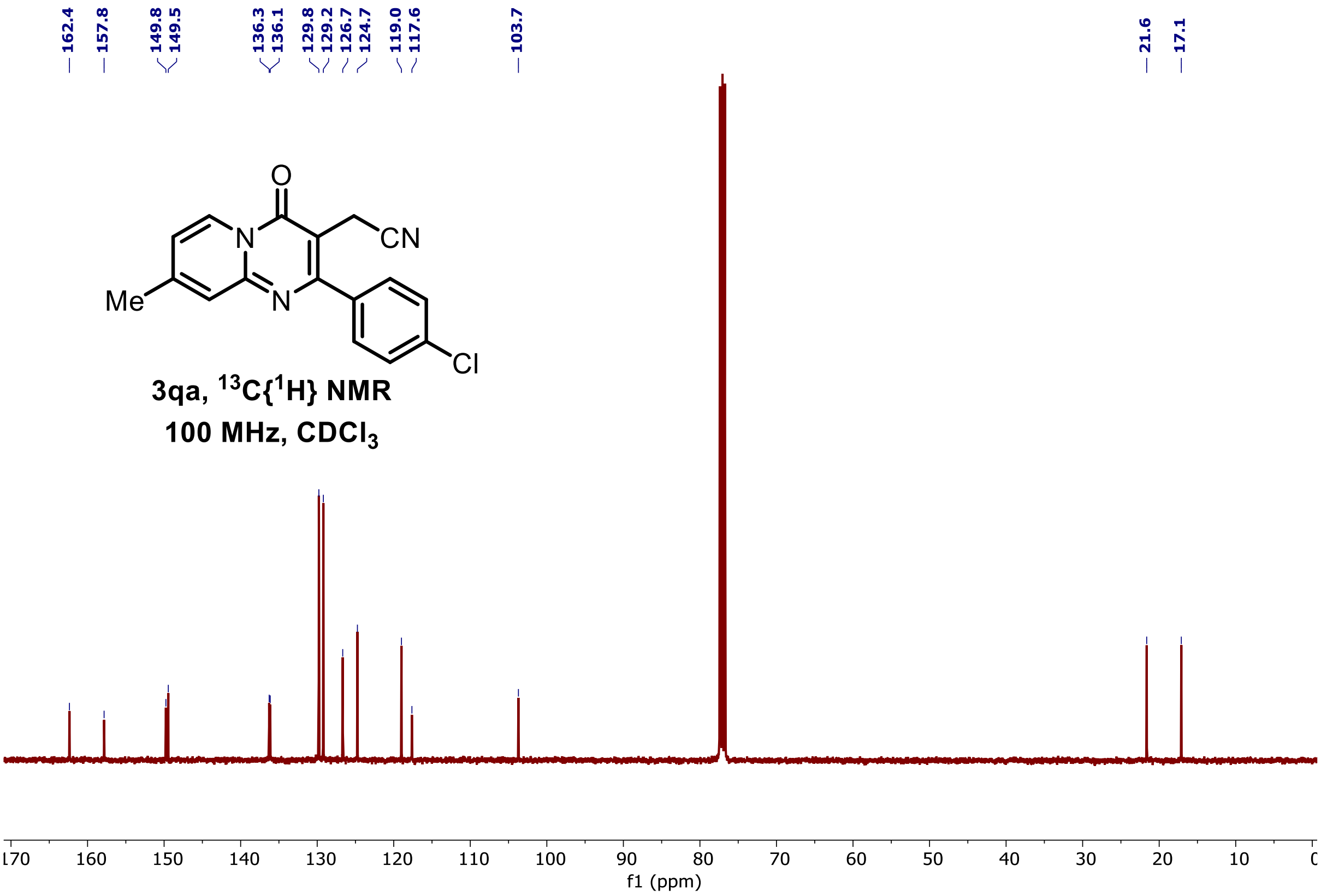
162.4
157.8
149.8
149.5
136.3
136.1
129.8
129.2
126.7
124.7
119.0
117.6
103.7
21.6
17.1
O
N
CN
Me
N
Cl
3qa, ¹³C{¹H} NMR
100 MHz, CDCl₃
f1 (ppm)

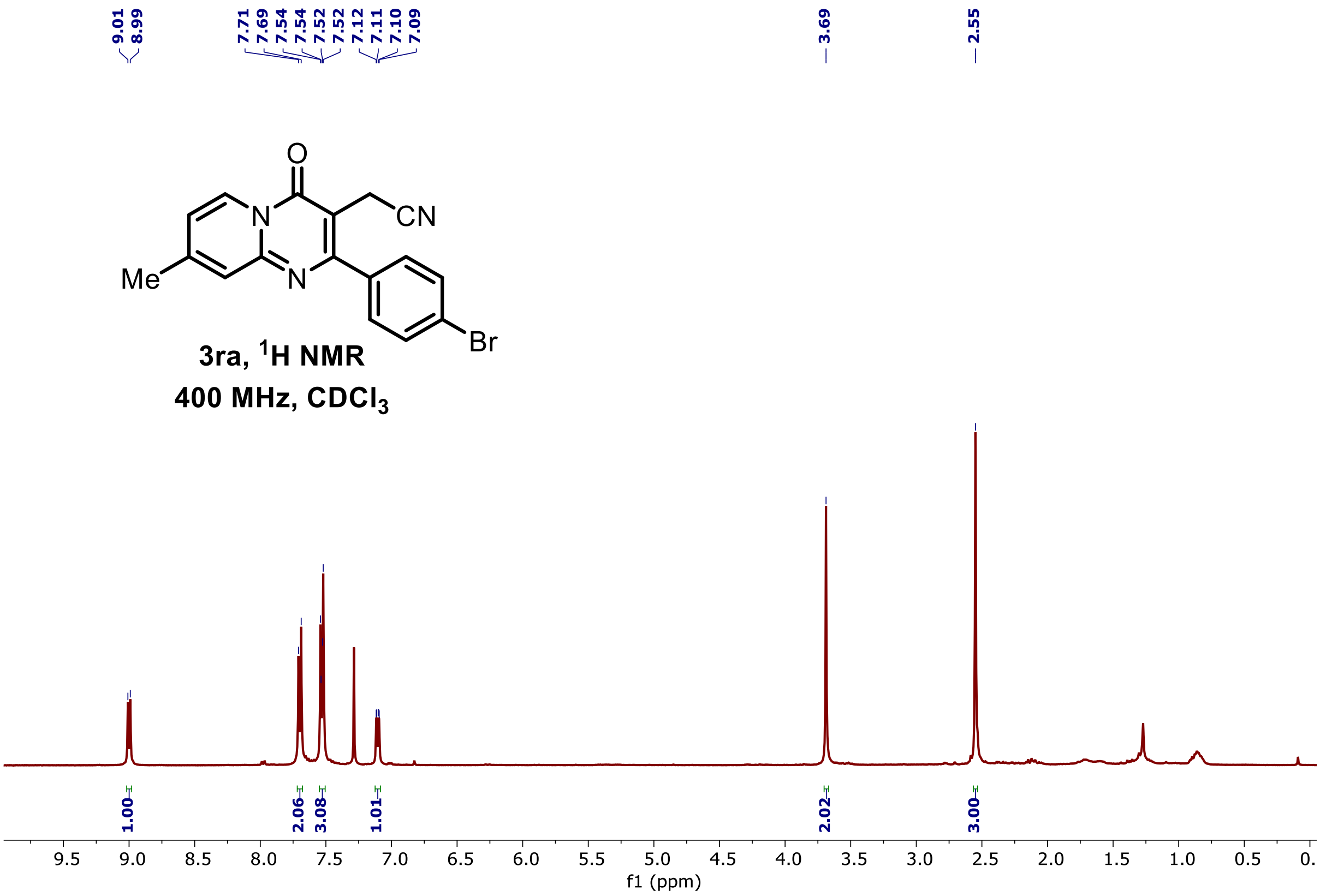
9.01
8.99
7.71
7.69
7.54
7.54
7.52
7.52
7.12
7.11
7.10
7.09
3.69
2.55
O
N
CN
Me
N
Br
3ra, 1H NMR
400 MHz, CDCl3
1.00
2.06
3.08
1.01
2.02
3.00
f1 (ppm)

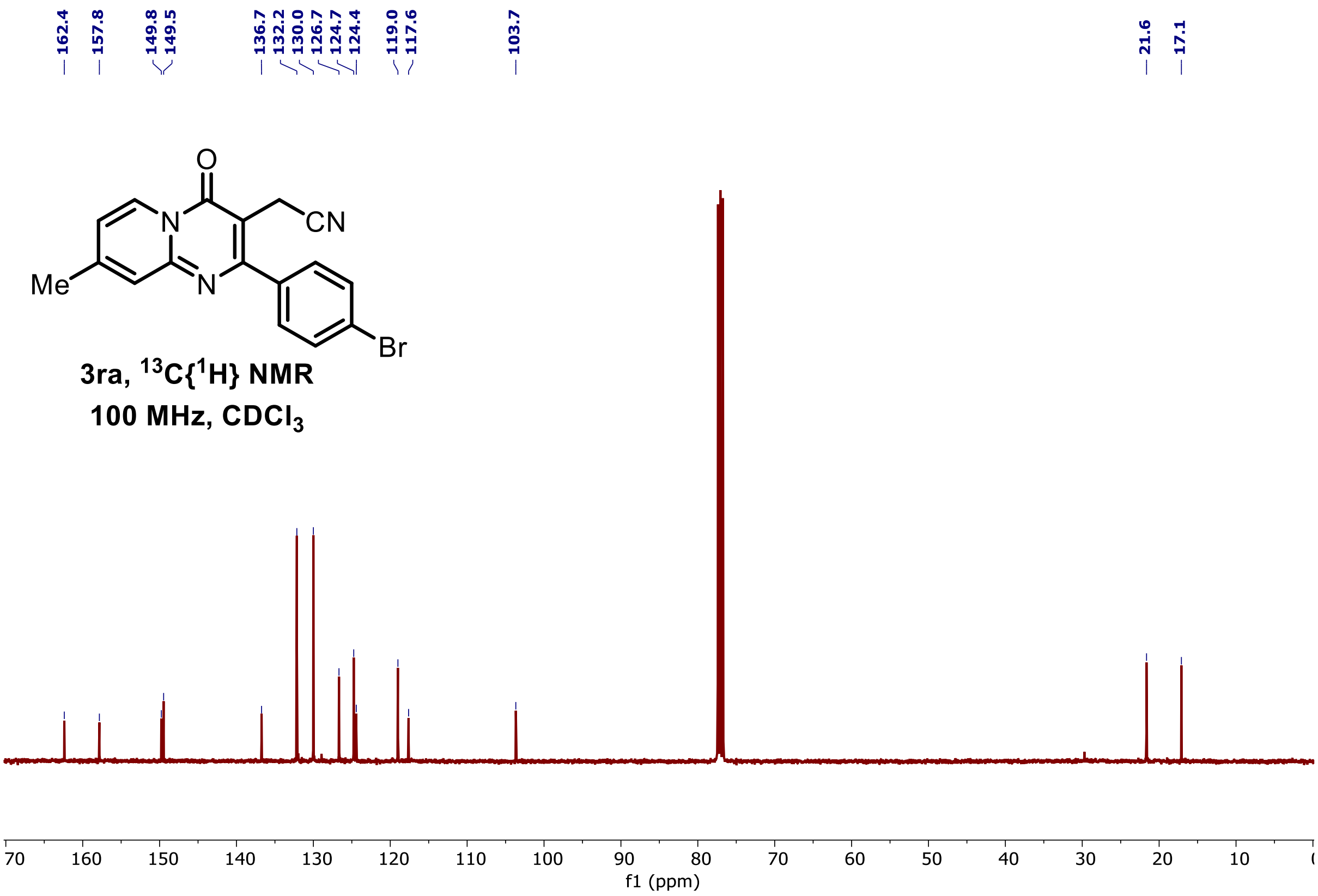
162.4
157.8
149.8
149.5
136.7
132.2
130.0
126.7
124.7
124.4
119.0
117.6
103.7
21.6
17.1
O
N
CN
Me
N
Br
3ra, 13C{1H} NMR
100 MHz, CDCl3
f1 (ppm)

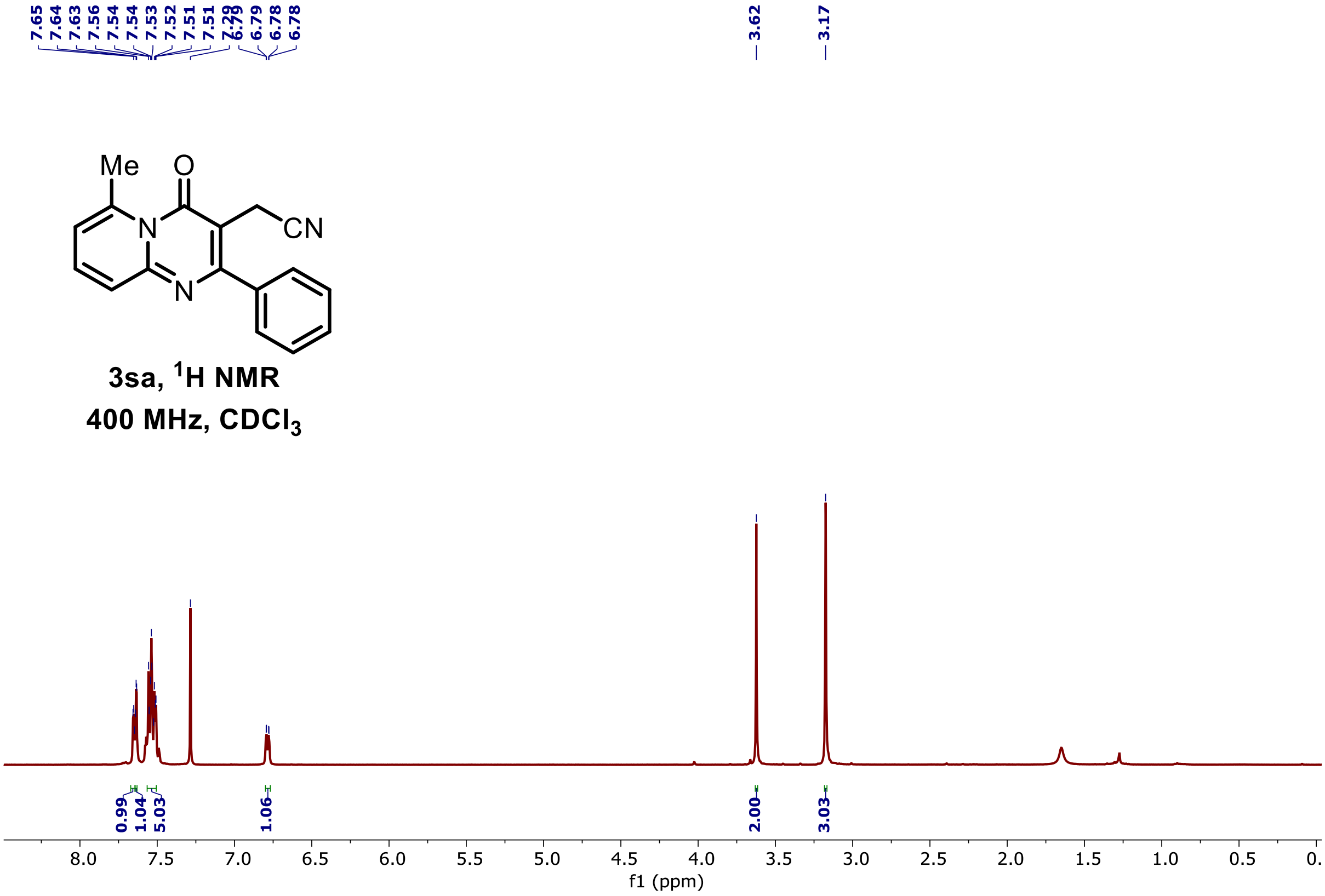
7.65
7.64
7.63
7.56
7.54
7.54
7.53
7.52
7.51
7.51
7.29
6.79
6.79
6.78
6.78
3.62
3.17
Me
O
N
CN
N
3sa, ^{1}H NMR
400 MHz, $CDCl_3$
0.99
1.04
5.03
1.06
2.00
3.03
8.0 7.5 7.0 6.5 6.0 5.5 5.0 4.5 4.0 3.5 3.0 2.5 2.0 1.5 1.0 0.5 0.
f1 (ppm)

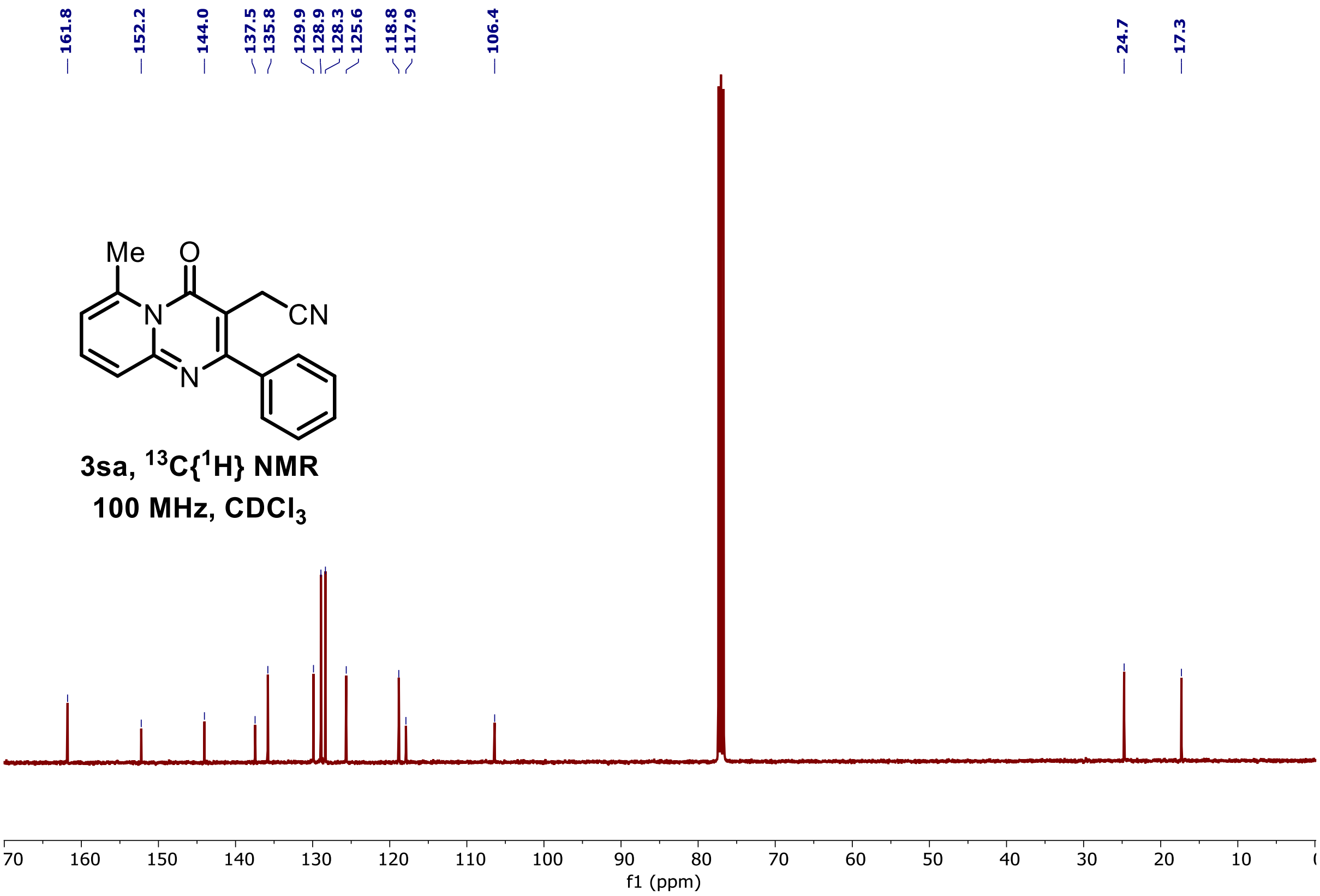
161.8
152.2
144.0
137.5
135.8
129.9
128.9
128.3
125.6
118.8
117.9
106.4
24.7
17.3
Me
O
N
CN
N
3sa, ^{13}C{^{1}H} NMR
100 MHz, $CDCl_3$
70 160 150 140 130 120 110 100 90 80 70 60 50 40 30 20 10
f1 (ppm)

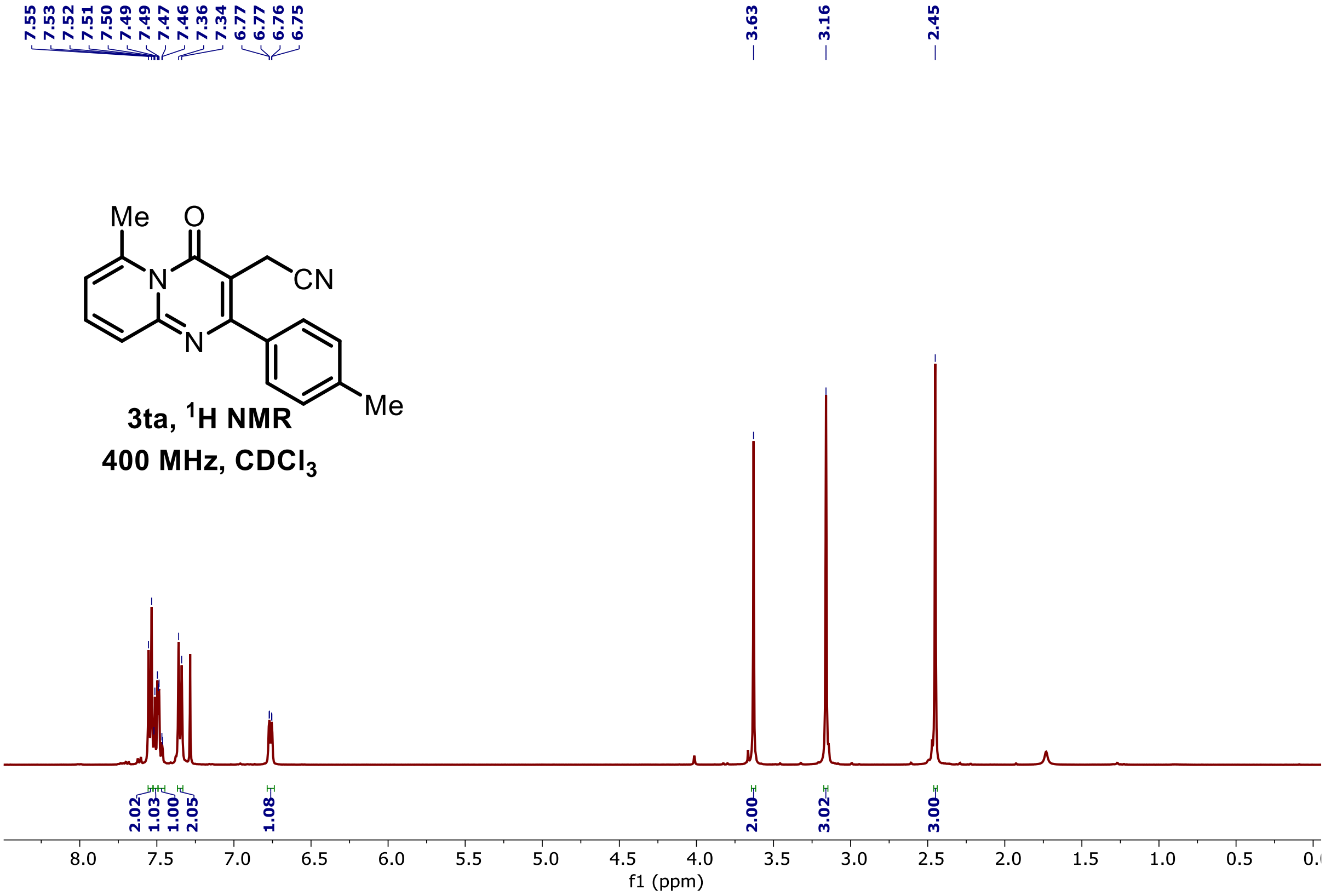
7.55
7.53
7.52
7.51
7.50
7.49
7.49
7.47
7.46
7.36
7.34
6.77
6.77
6.76
6.75
3.63
3.16
2.45
Me
O
N
CN
N
Me
3ta, ¹H NMR
400 MHz, CDCl3
2.02
1.03
1.00
2.05
1.08
2.00
3.02
3.00
f1 (ppm)

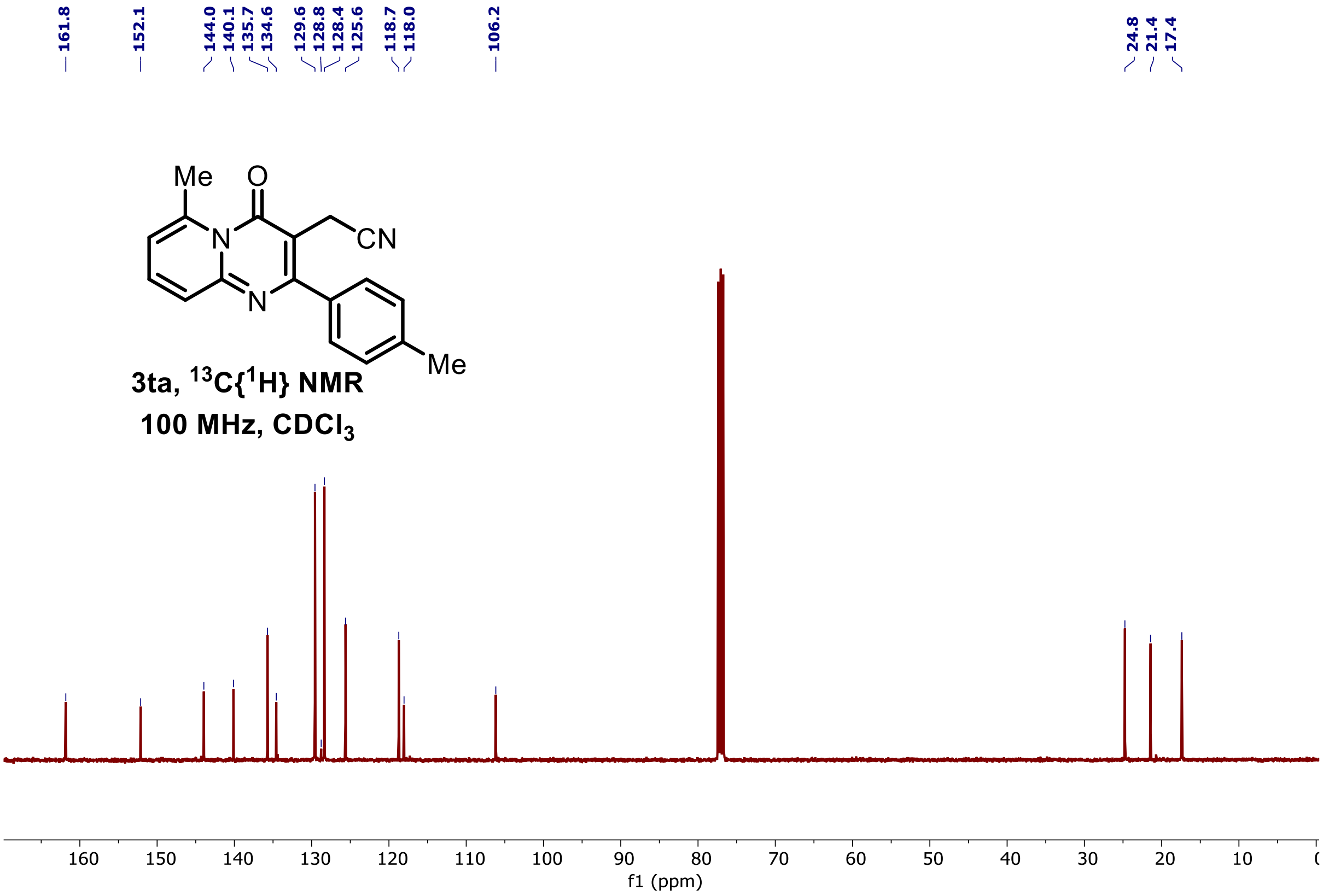
161.8
152.1
144.0
140.1
135.7
134.6
129.6
128.8
128.4
125.6
118.7
118.0
106.2
24.8
21.4
17.4
Me
O
N
CN
N
Me
3ta, ¹³C{¹H} NMR
100 MHz, CDCl3
f1 (ppm)

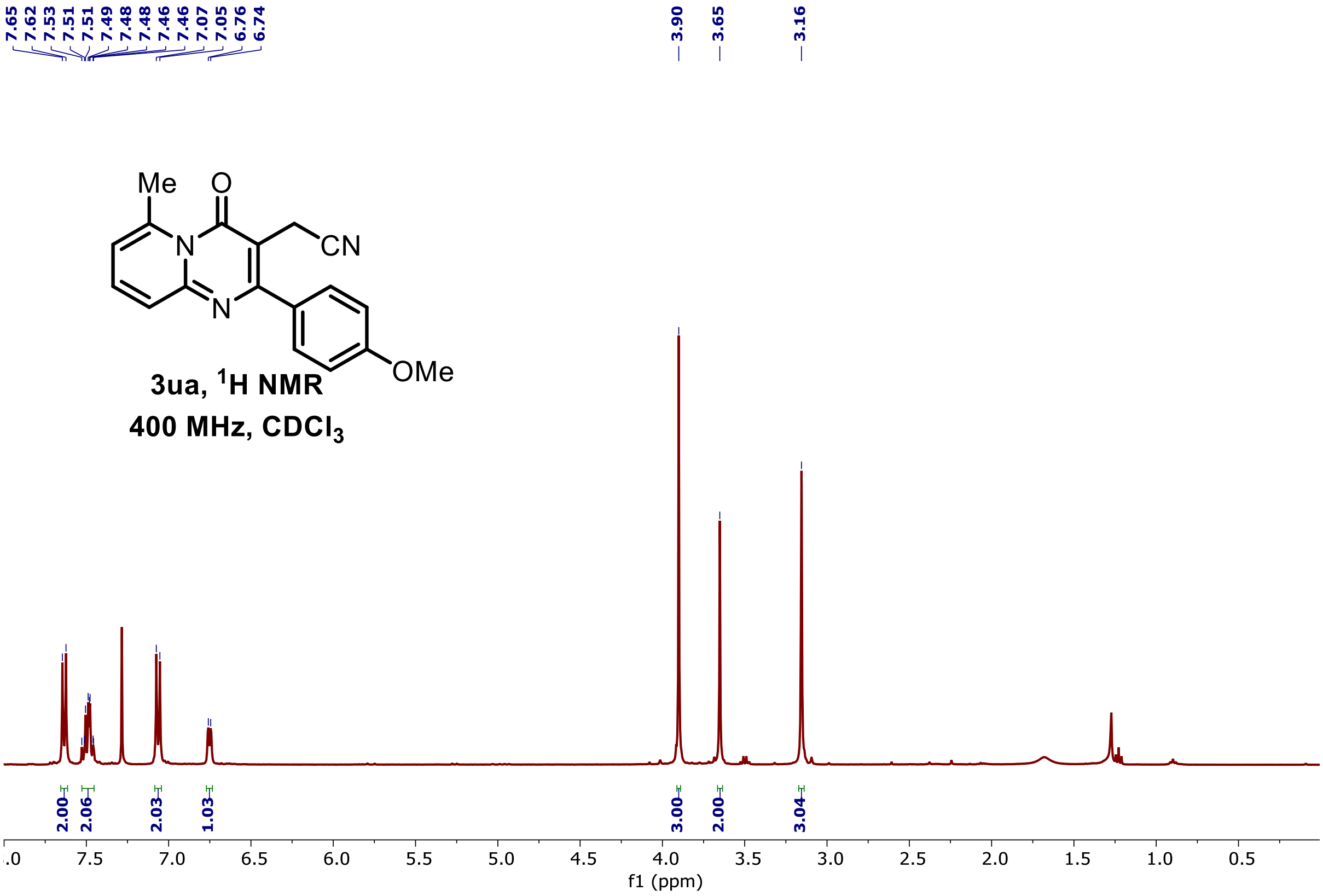
7.65 7.62 7.53 7.51 7.51 7.49 7.48 7.48 7.46 7.46 7.07 7.05 6.76 6.74
3.90
3.65
3.16
Me
O
N
CN
N
OMe
3ua, 1H NMR
400 MHz, CDCl3
2.00 2.06 2.03 1.03 3.00 2.00 3.04
f1 (ppm)

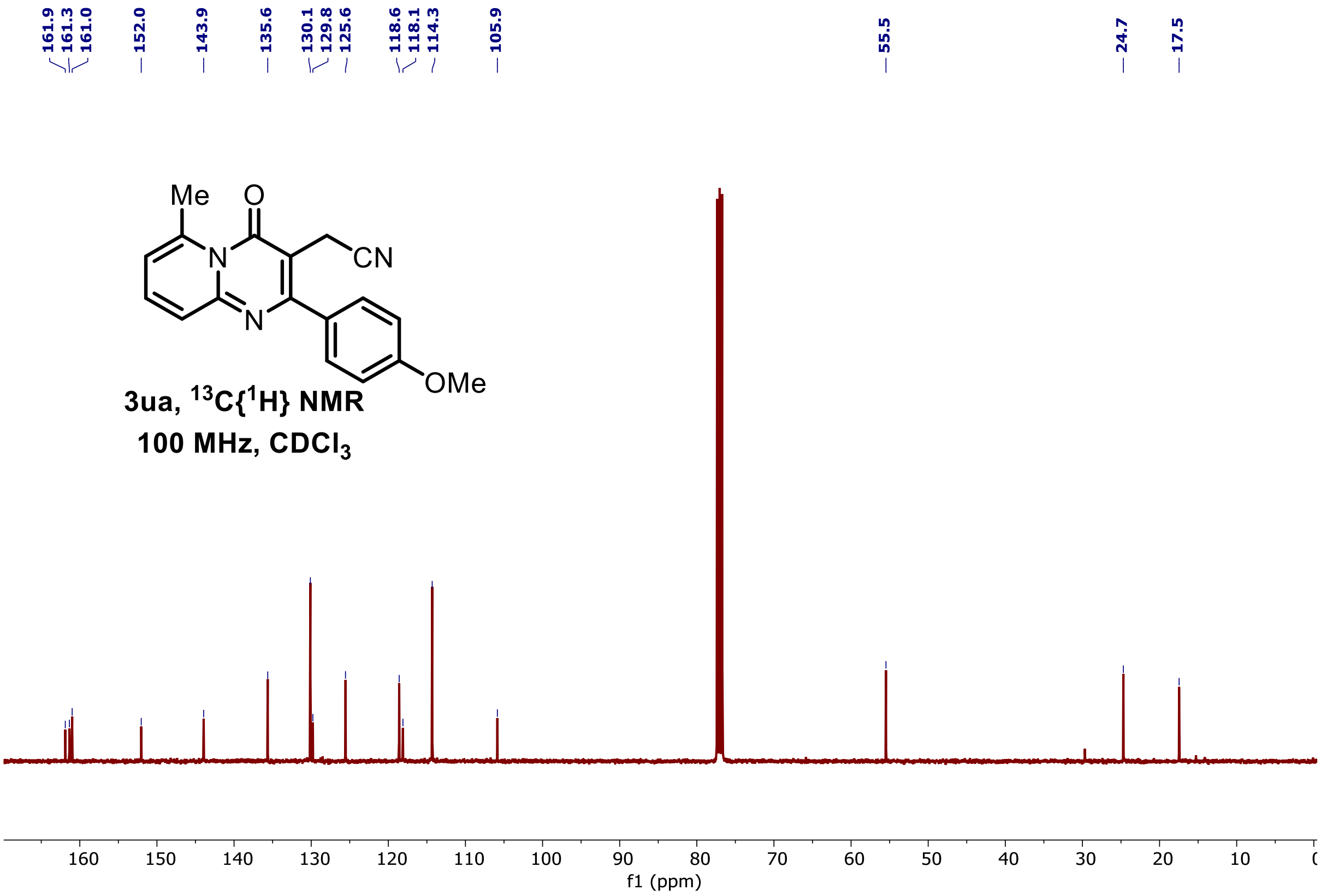
161.9 161.3 161.0
152.0
143.9
135.6
130.1 129.8 125.6
118.6 118.1 114.3
105.9
55.5
24.7
17.5
Me
O
N
CN
N
OMe
3ua, 13C{1H} NMR
100 MHz, CDCl3
f1 (ppm)

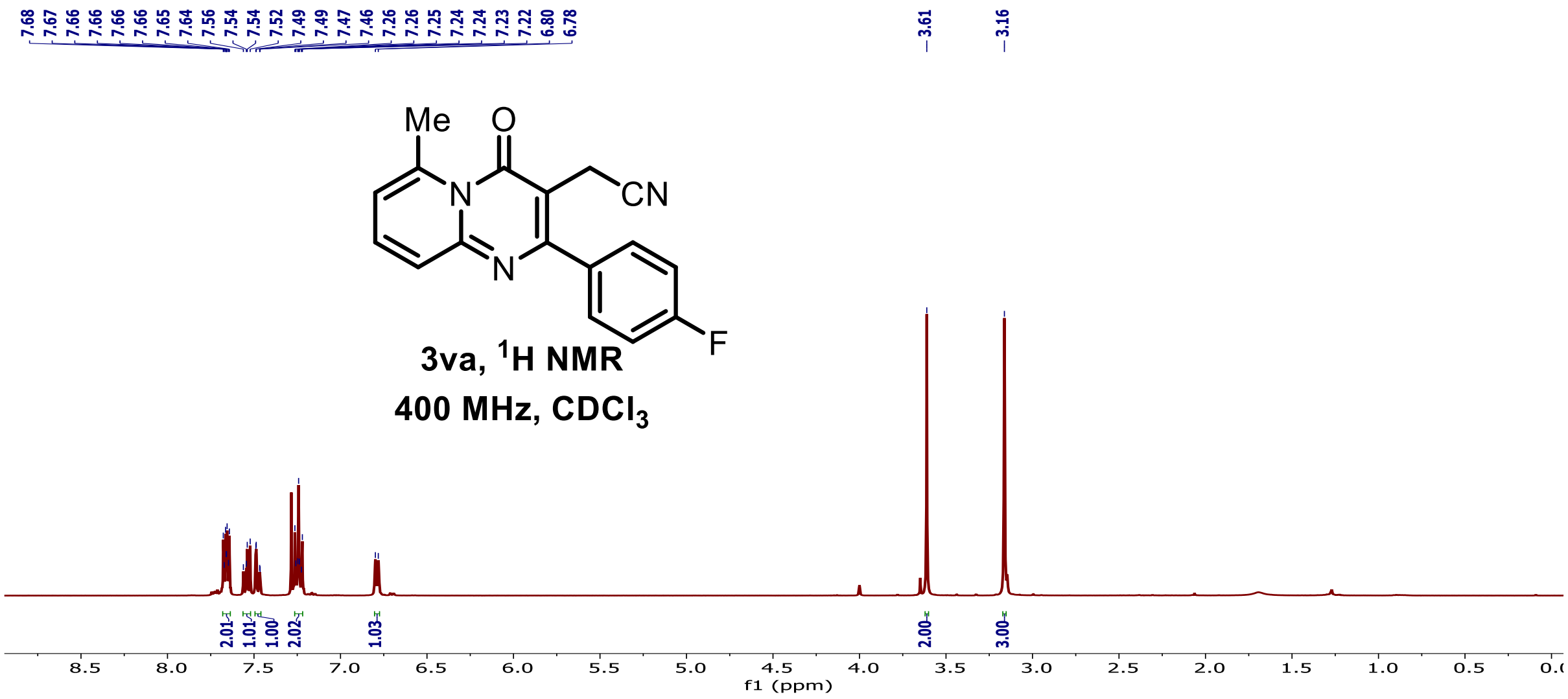
7.68
7.67
7.66
7.66
7.66
7.65
7.64
7.56
7.54
7.54
7.52
7.49
7.49
7.47
7.46
7.26
7.26
7.25
7.24
7.24
7.23
7.22
6.80
6.78
3.61
3.16
Me
O
N
CN
N
F
3va, 1H NMR
400 MHz, CDCl3
2.01
1.01
1.00
2.02
1.03
2.00
3.00
f1 (ppm)

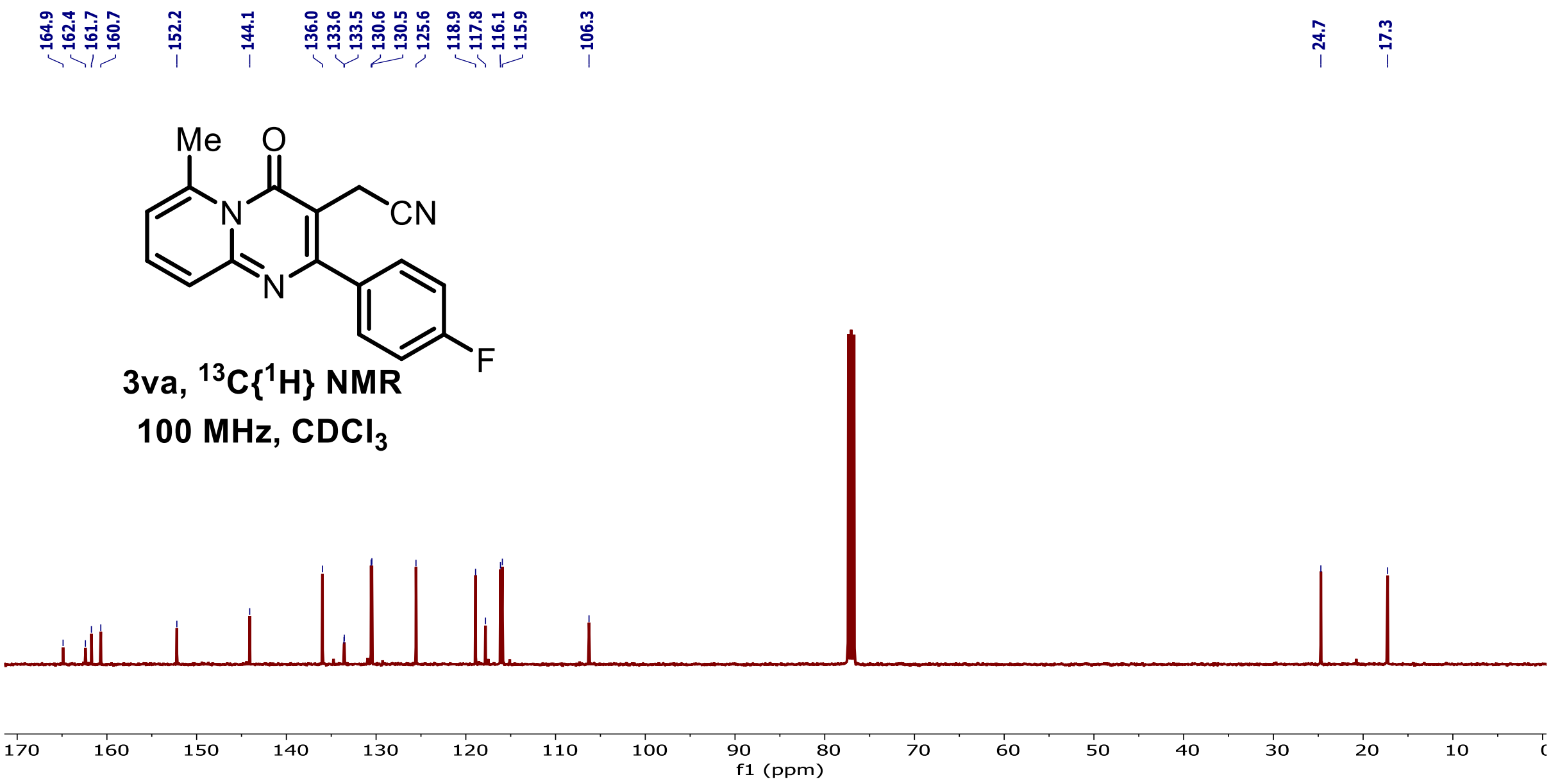
164.9
162.4
161.7
160.7
152.2
144.1
136.0
133.6
133.5
130.6
130.5
125.6
118.9
117.8
116.1
115.9
106.3
24.7
17.3
Me
O
N
CN
N
F
3va, 13C{1H} NMR
100 MHz, CDCl3
f1 (ppm)

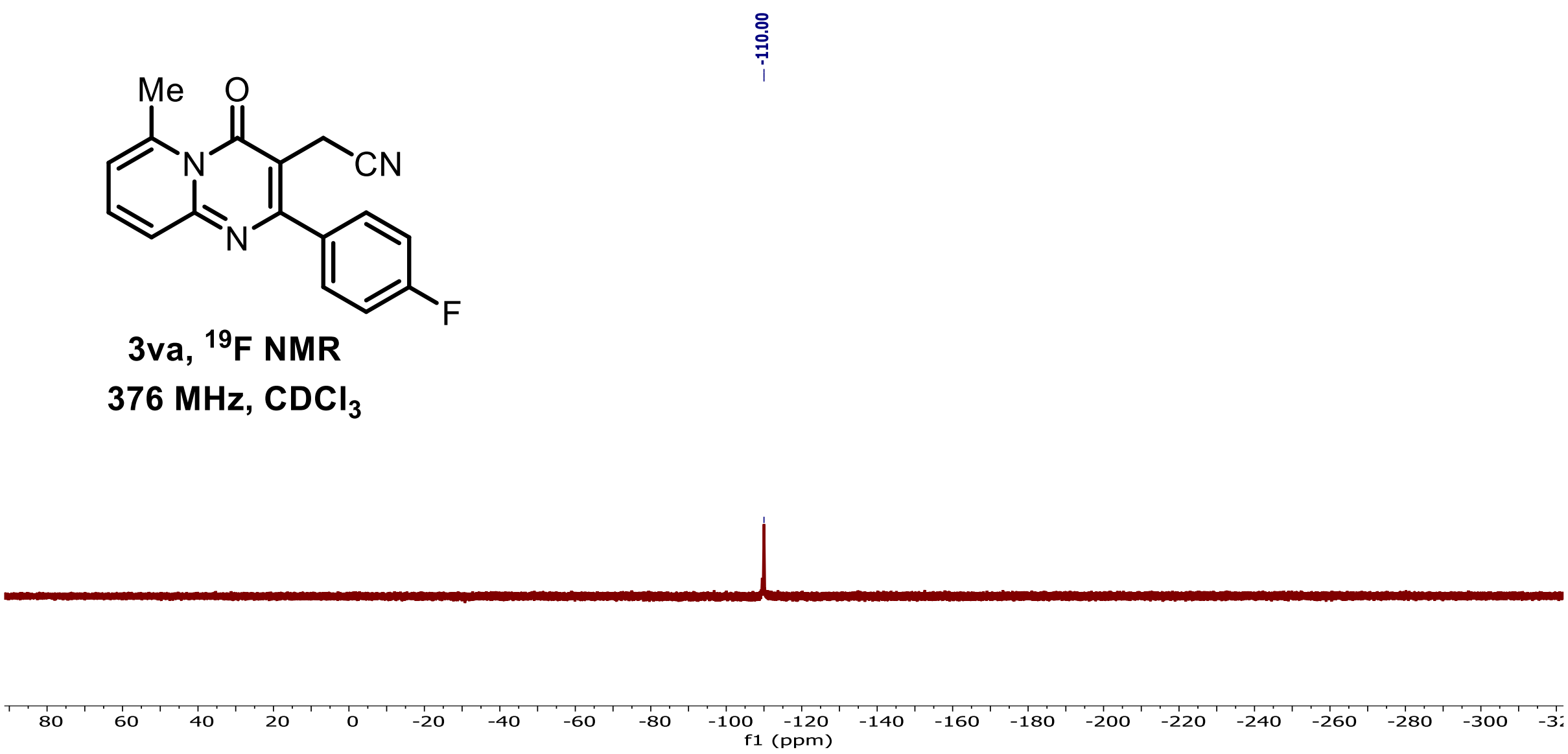
-110.00
Me
O
N
CN
N
F
3va, 19F NMR
376 MHz, CDCl3
f1 (ppm)

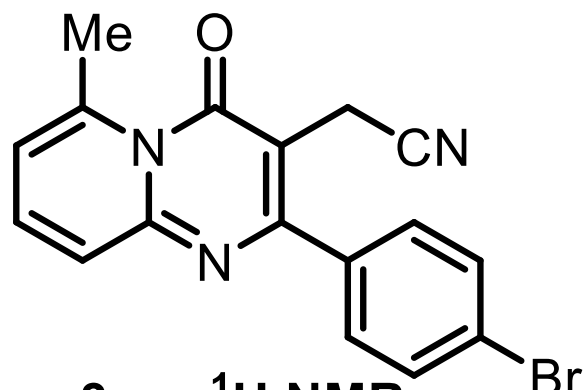

3wa, ¹H NMR
400 MHz, $CDCl_3$

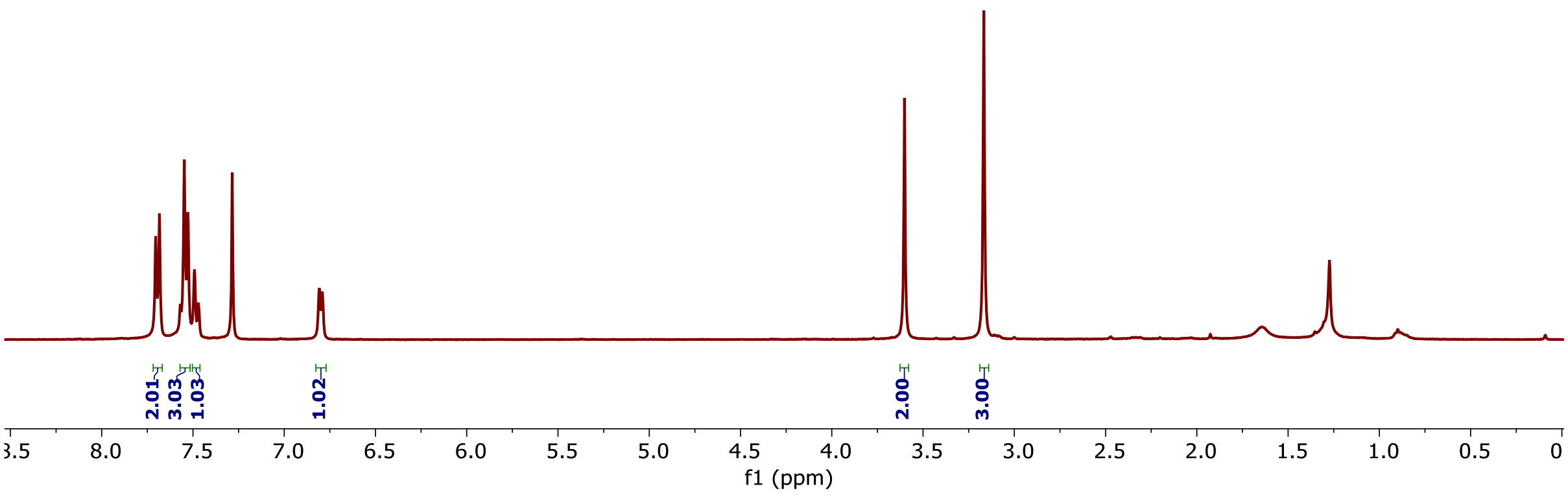

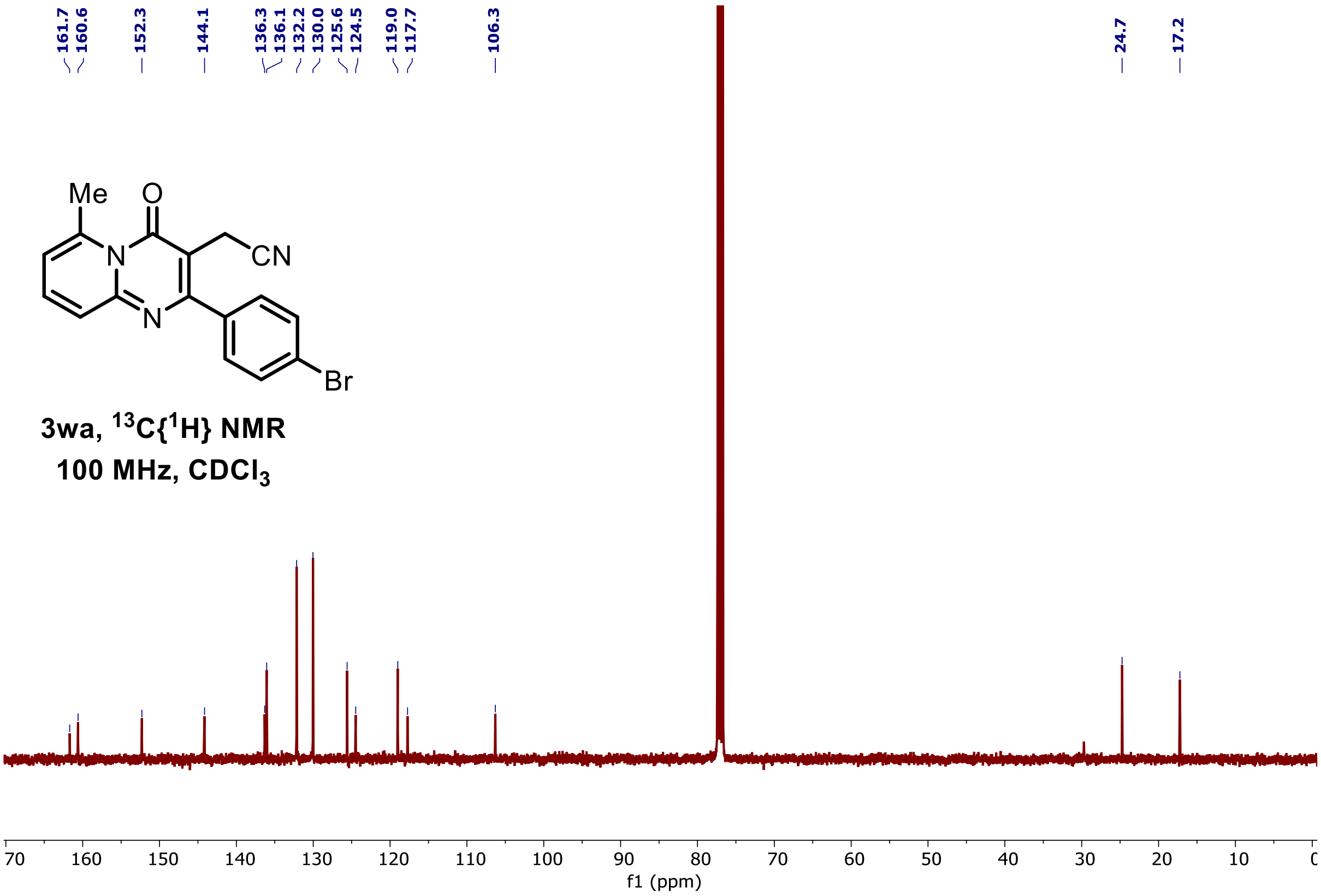

3wa, $^{13}C\{^1H\}$ NMR
100 MHz, $CDCl_3$

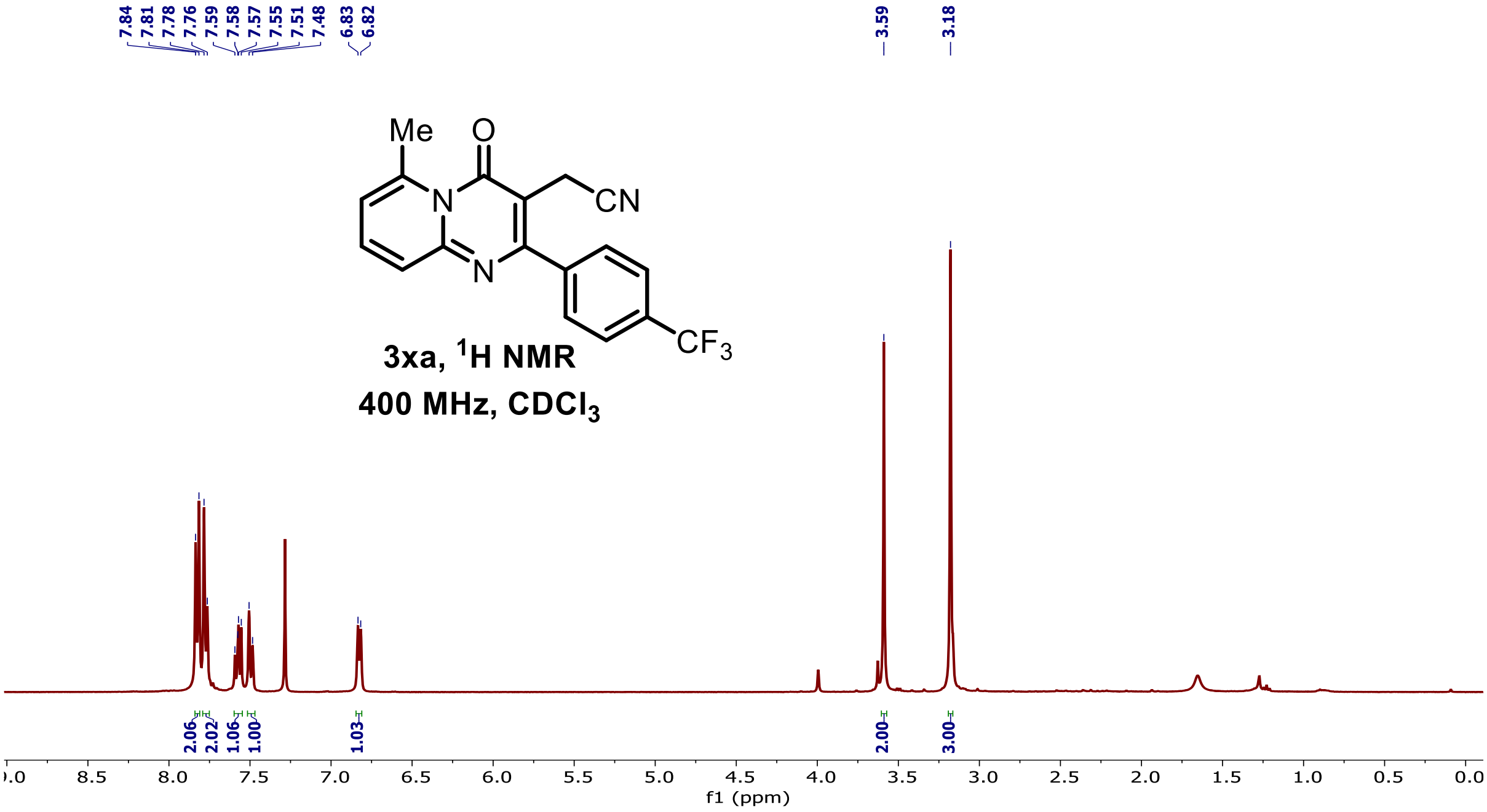
7.84
7.81
7.78
7.76
7.59
7.58
7.57
7.55
7.51
7.48
6.83
6.82
3.59
3.18
Me
O
N
CN
N
CF3
3xa, 1H NMR
400 MHz, CDCl3
2.06
2.02
1.06
1.00
1.03
2.00
3.00
f1 (ppm)

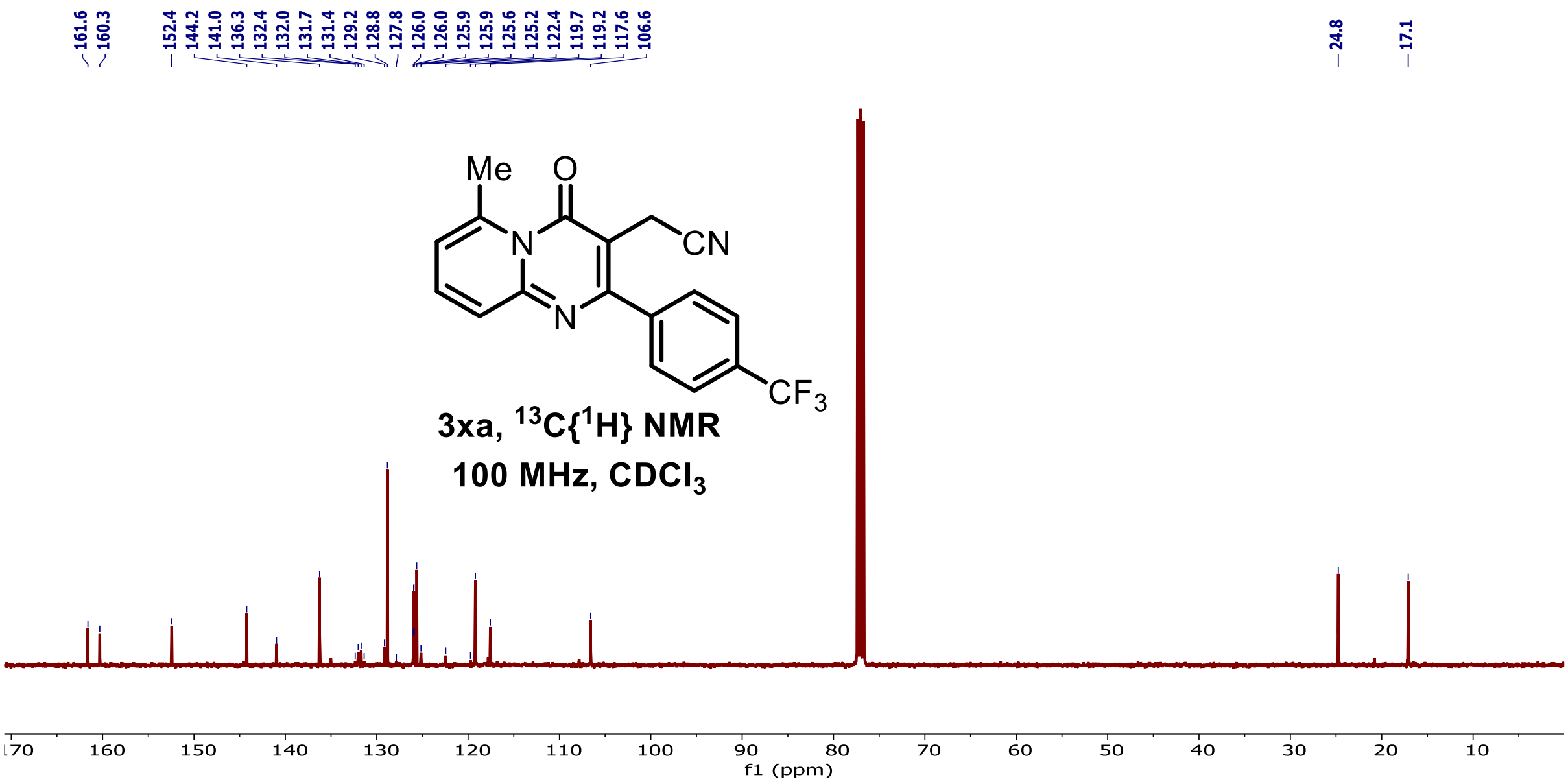
161.6
160.3
152.4
144.2
141.0
136.3
132.4
132.0
131.7
131.4
129.2
128.8
127.8
126.0
126.0
125.9
125.9
125.6
125.2
122.4
119.7
119.2
117.6
106.6
24.8
17.1
Me
O
N
CN
N
CF3
3xa, 13C{1H} NMR
100 MHz, CDCl3
f1 (ppm)

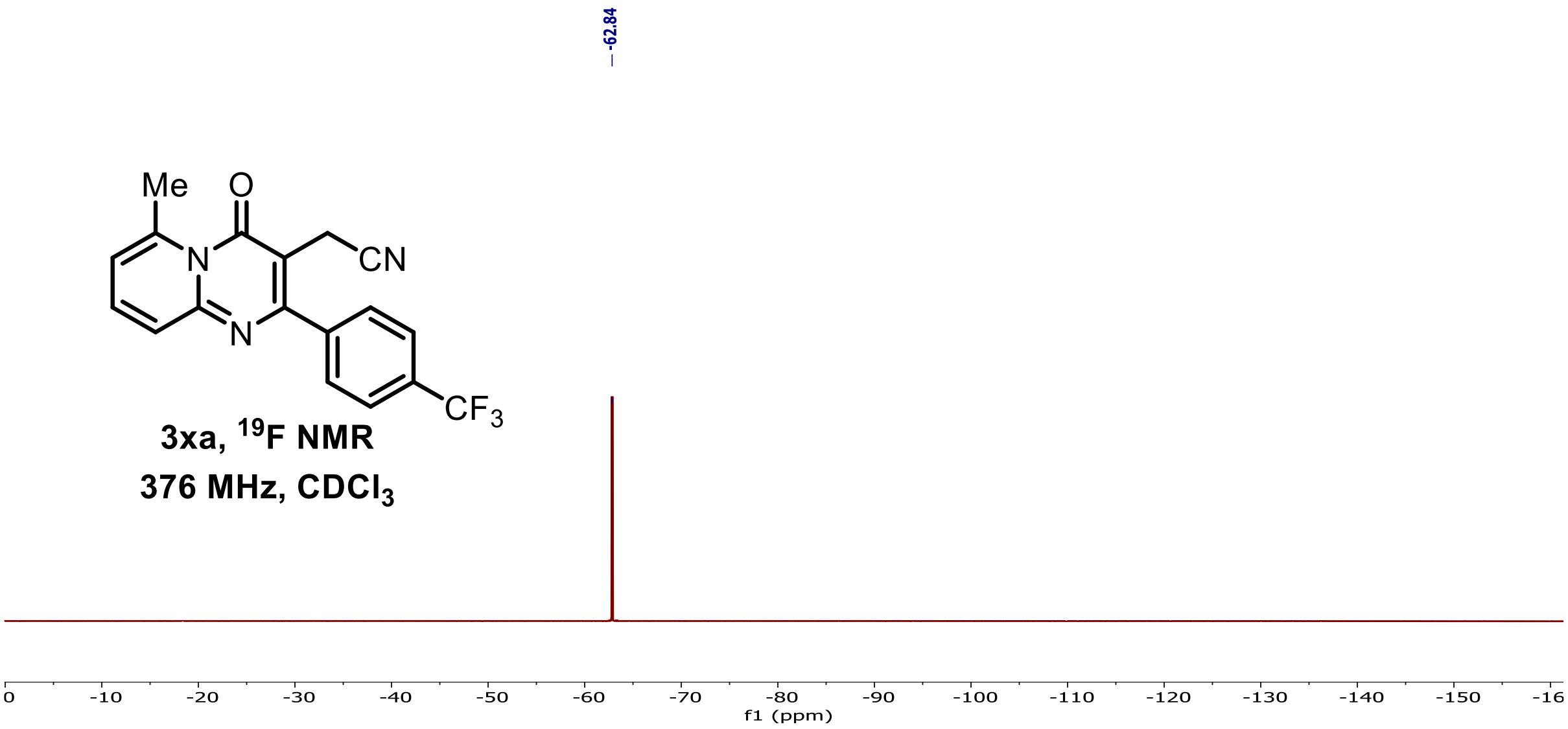
-62.84
Me
O
N
CN
N
CF3
3xa, 19F NMR
376 MHz, CDCl3
f1 (ppm)

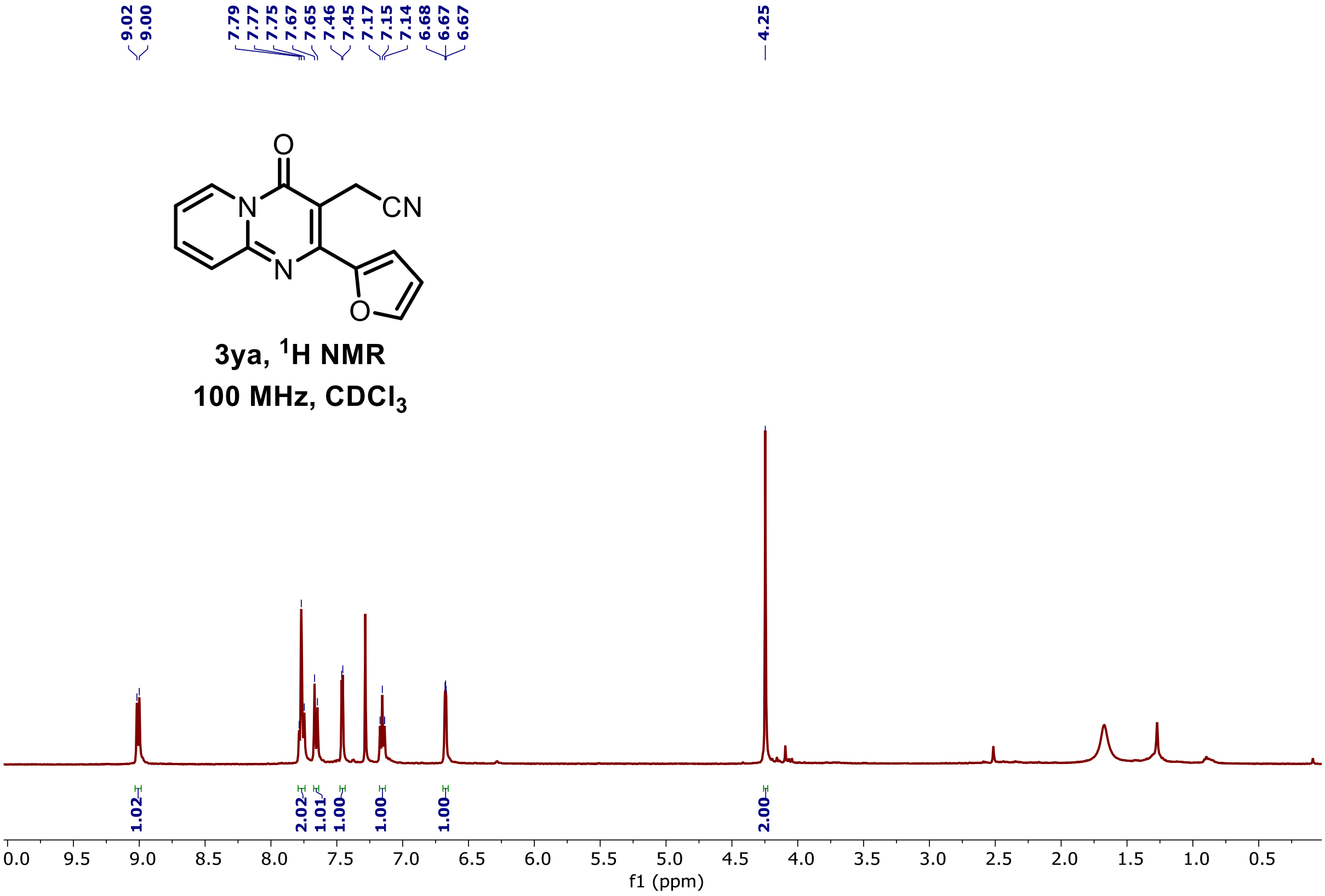
9.02
9.00
7.79
7.77
7.75
7.67
7.65
7.46
7.45
7.17
7.15
7.14
6.68
6.67
6.67
4.25
O
N
CN
N
O
3ya, 1H NMR
100 MHz, CDCl3
1.02
2.02
1.01
1.00
1.00
1.00
2.00
f1 (ppm)

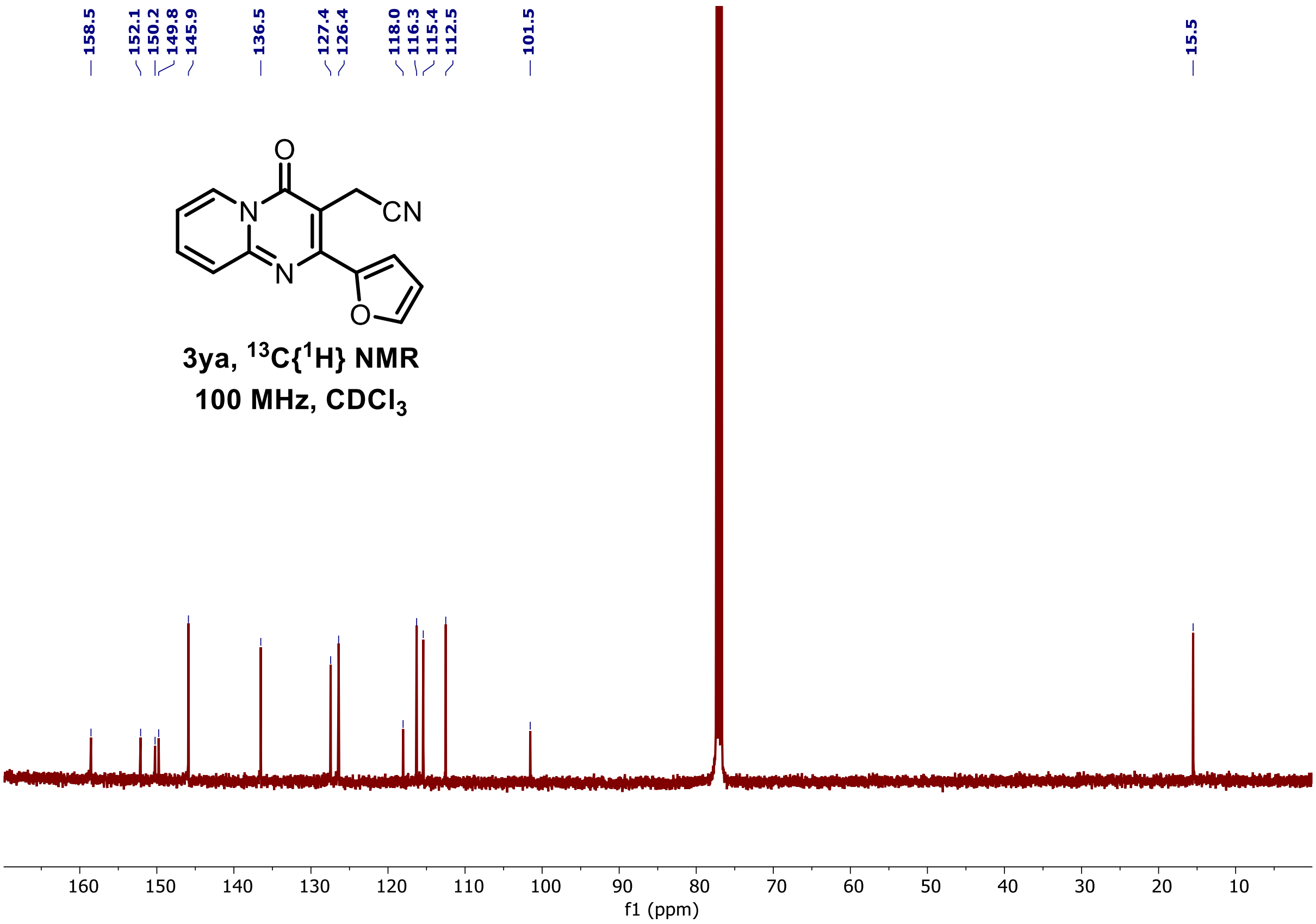
158.5
152.1
150.2
149.8
145.9
136.5
127.4
126.4
118.0
116.3
115.4
112.5
101.5
15.5
O
N
CN
N
O
3ya, 13C{1H} NMR
100 MHz, CDCl3
f1 (ppm)

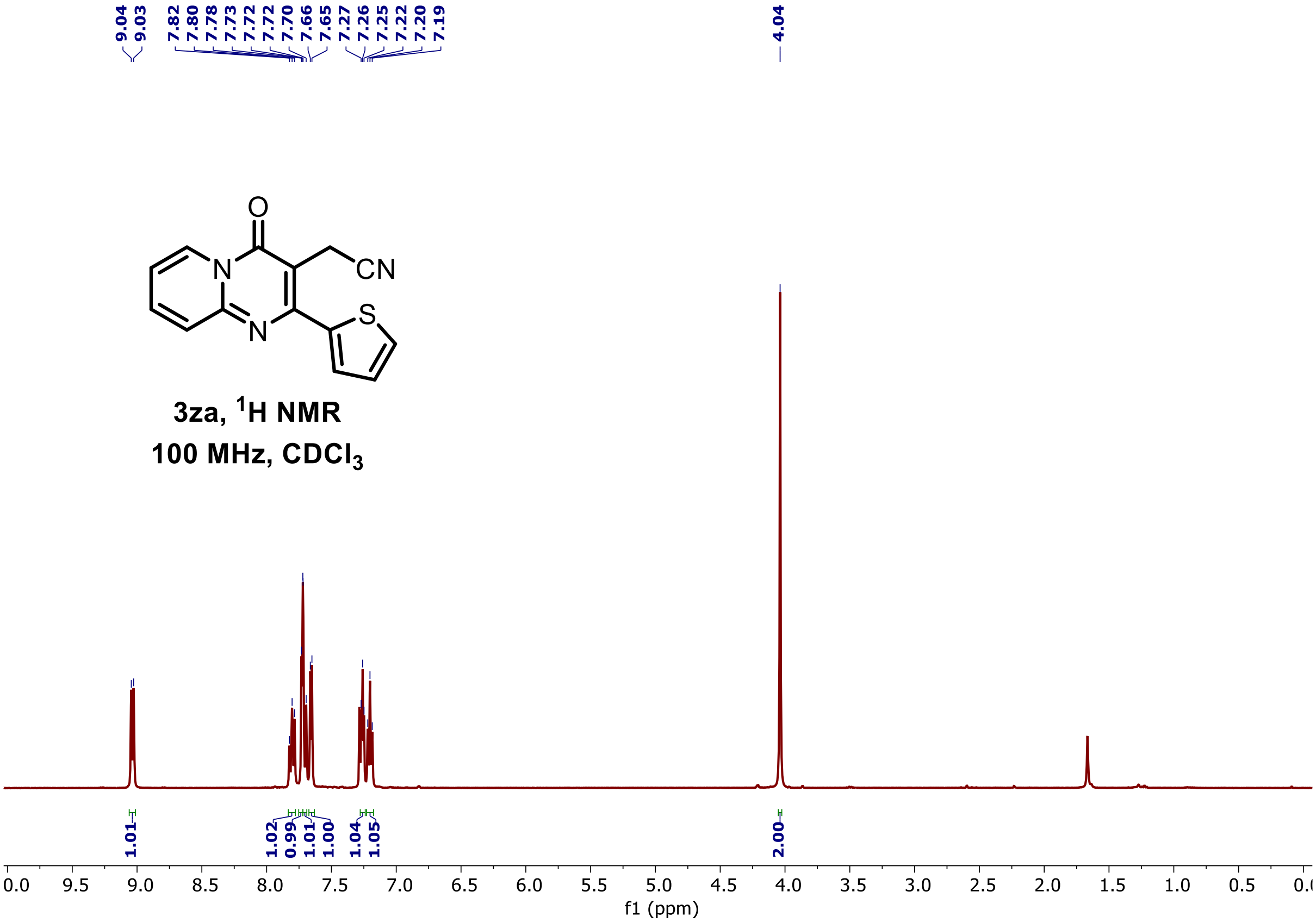
9.04
9.03
7.82
7.80
7.78
7.73
7.72
7.72
7.70
7.66
7.65
7.27
7.26
7.25
7.22
7.20
7.19
4.04
O
N
N
CN
S
3za, ^{1}H NMR
100 MHz, $CDCl_3$
1.01
1.02
0.99
1.01
1.00
1.04
1.05
2.00
f1 (ppm)

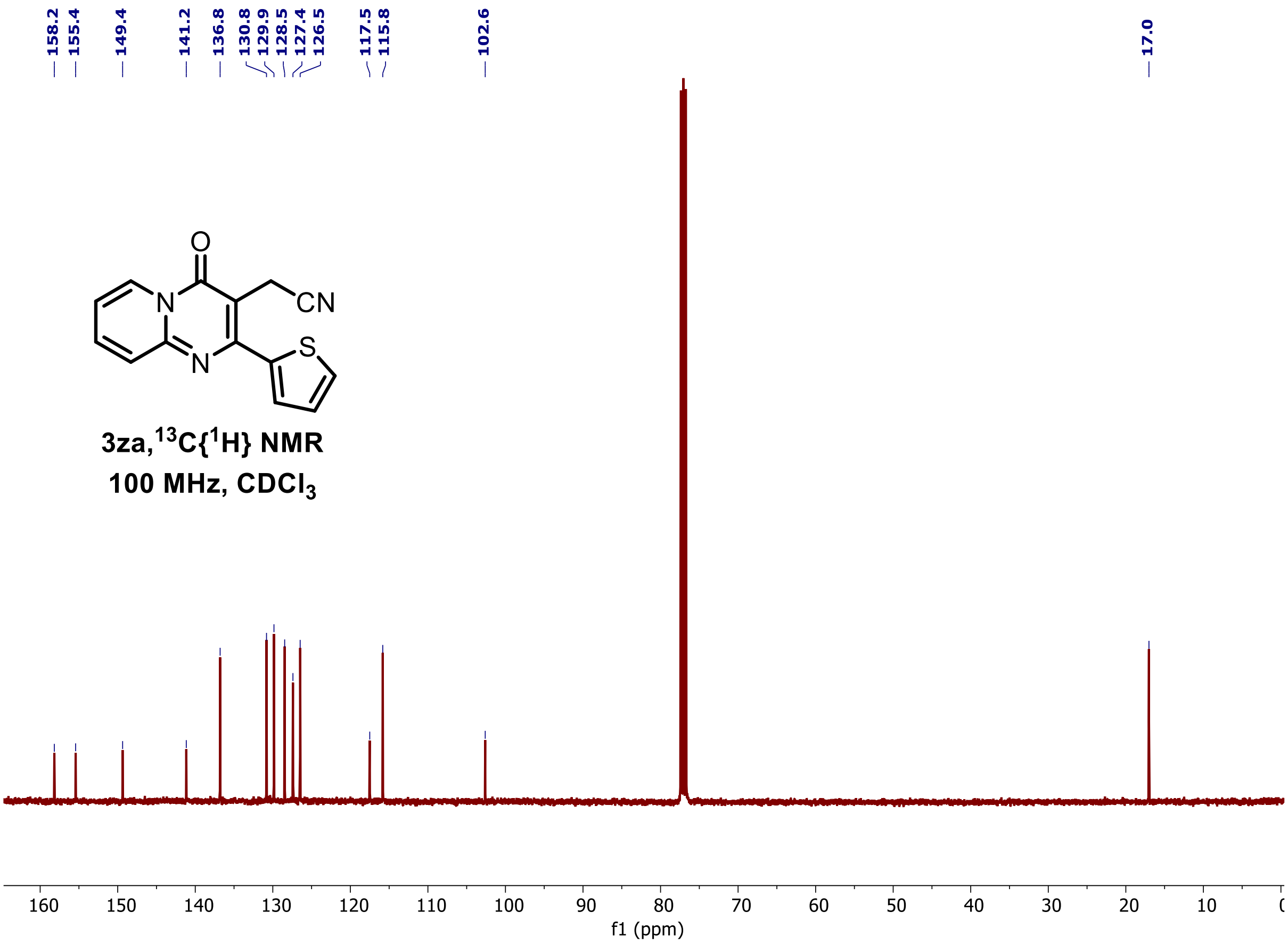
158.2
155.4
149.4
141.2
136.8
130.8
129.9
128.5
127.4
126.5
117.5
115.8
102.6
17.0
O
N
N
CN
S
3za, ^{13}C{^{1}H} NMR
100 MHz, $CDCl_3$
f1 (ppm)

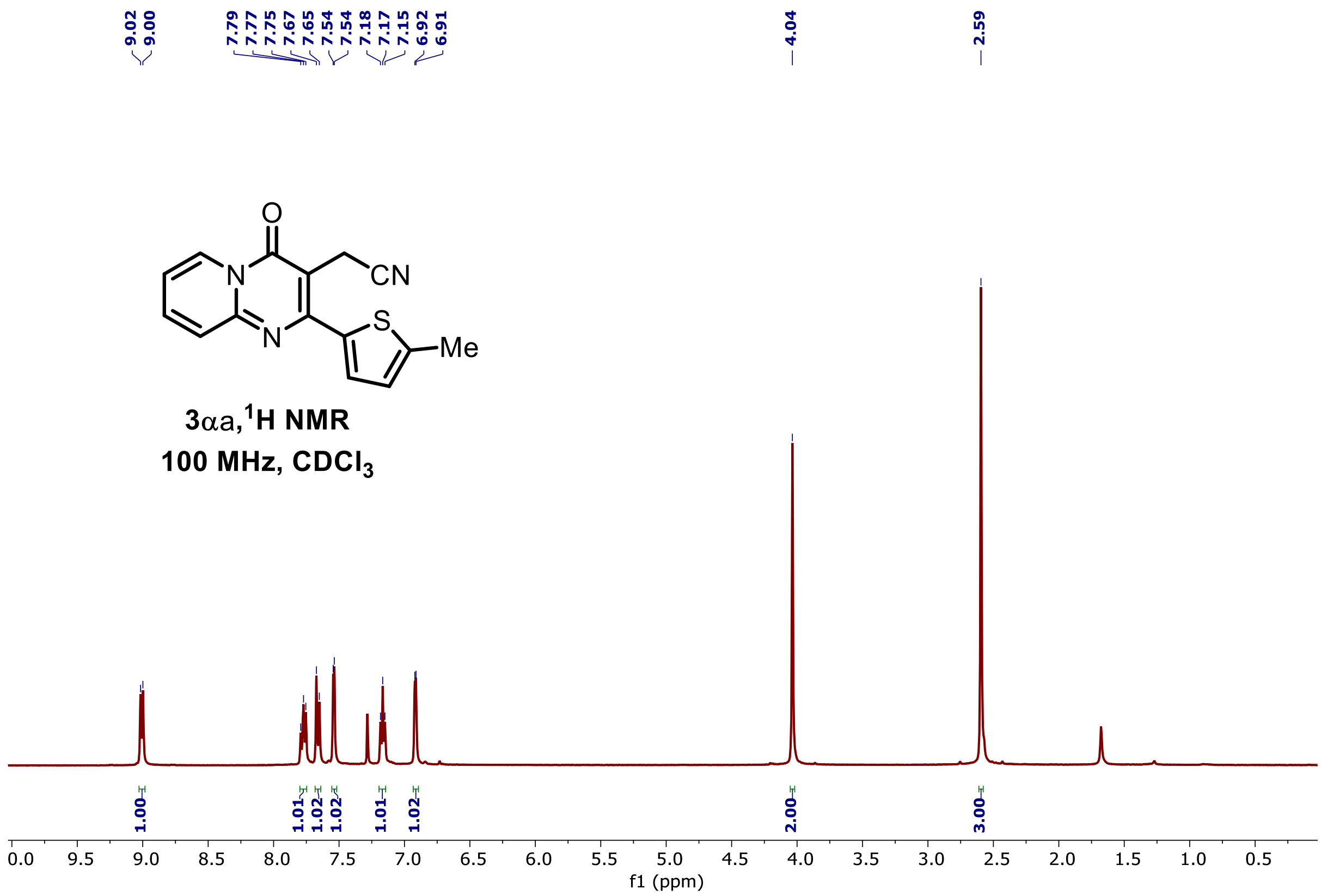
3αa, ^{1}H NMR
100 MHz, $CDCl_3$
f1 (ppm)

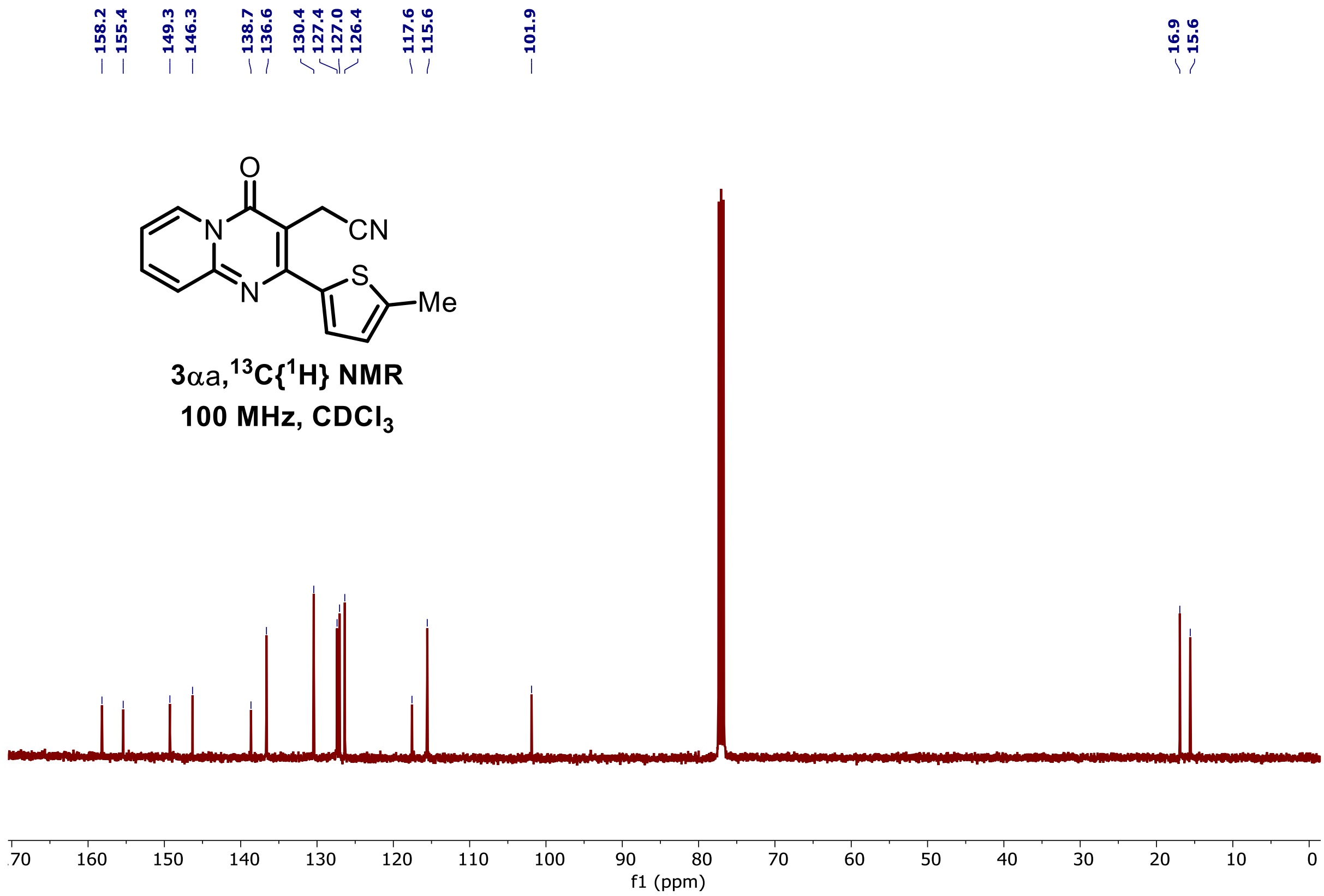
3αa, ^{13}C{^{1}H} NMR
100 MHz, $CDCl_3$
f1 (ppm)

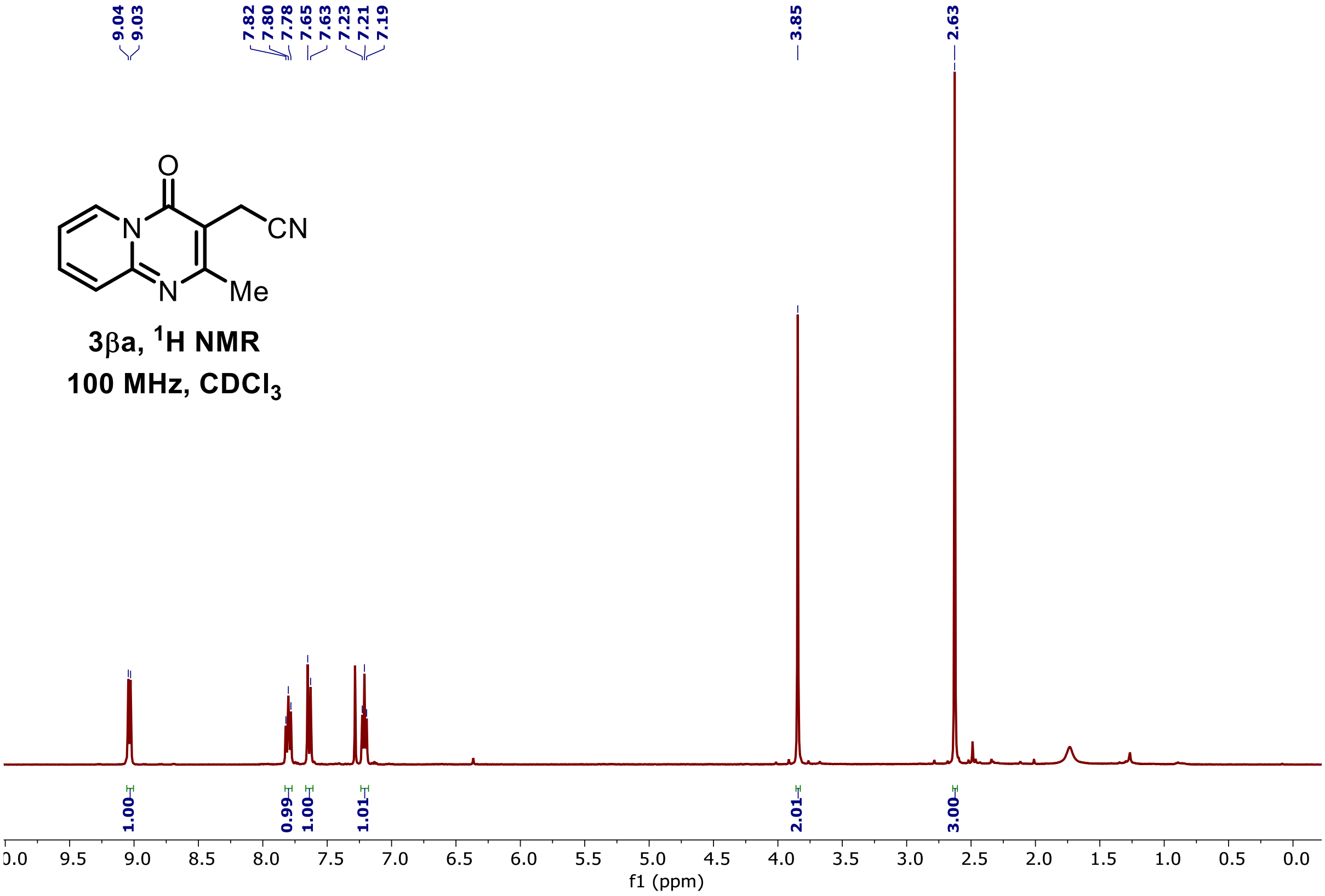
9.04
9.03
7.82
7.80
7.78
7.65
7.63
7.23
7.21
7.19
3.85
2.63
O
N
CN
N
Me
3βa, 1H NMR
100 MHz, CDCl3
1.00
0.99
1.00
1.01
2.01
3.00
f1 (ppm)

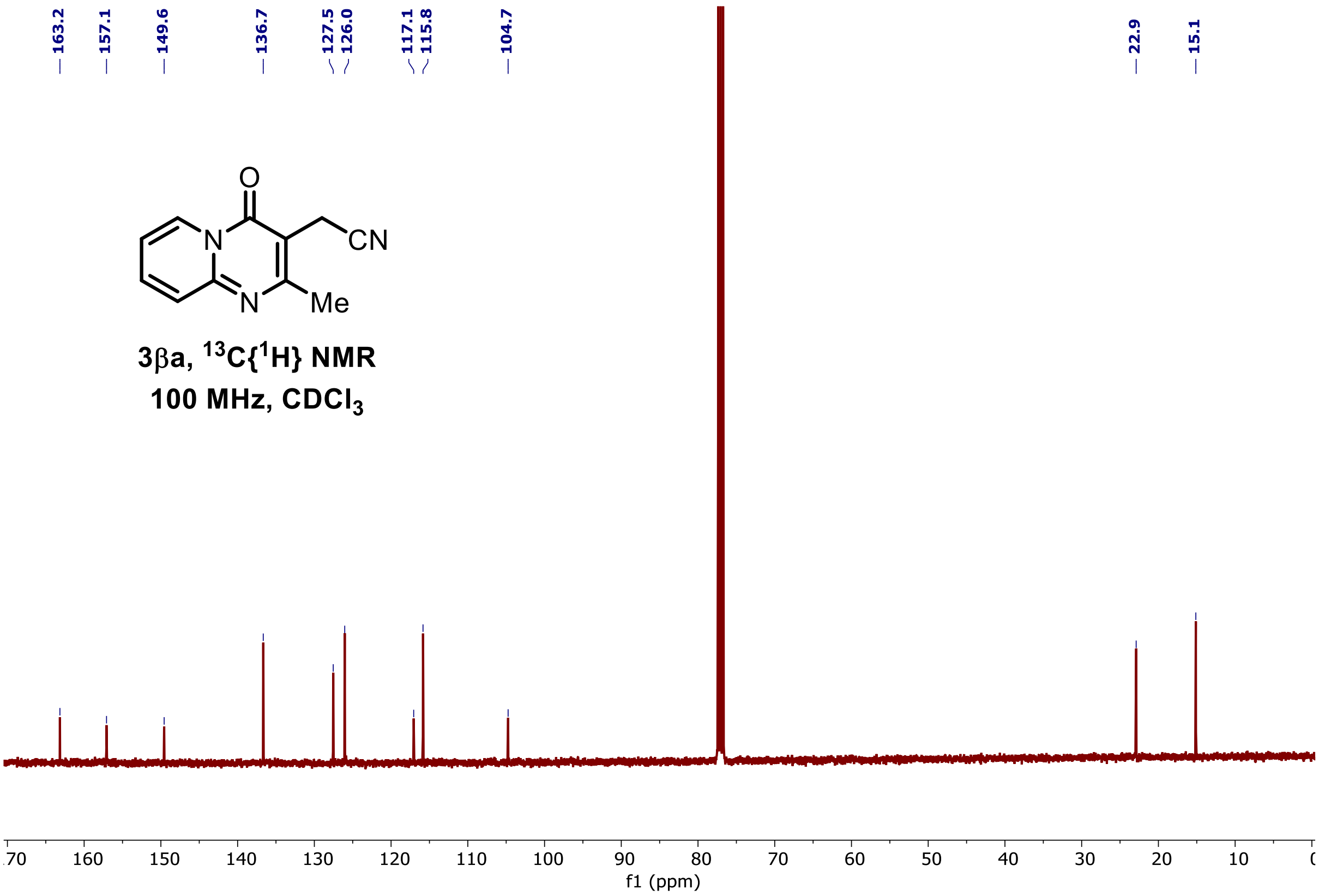
163.2
157.1
149.6
136.7
127.5
126.0
117.1
115.8
104.7
22.9
15.1
O
N
CN
N
Me
3βa, 13C{1H} NMR
100 MHz, CDCl3
f1 (ppm)

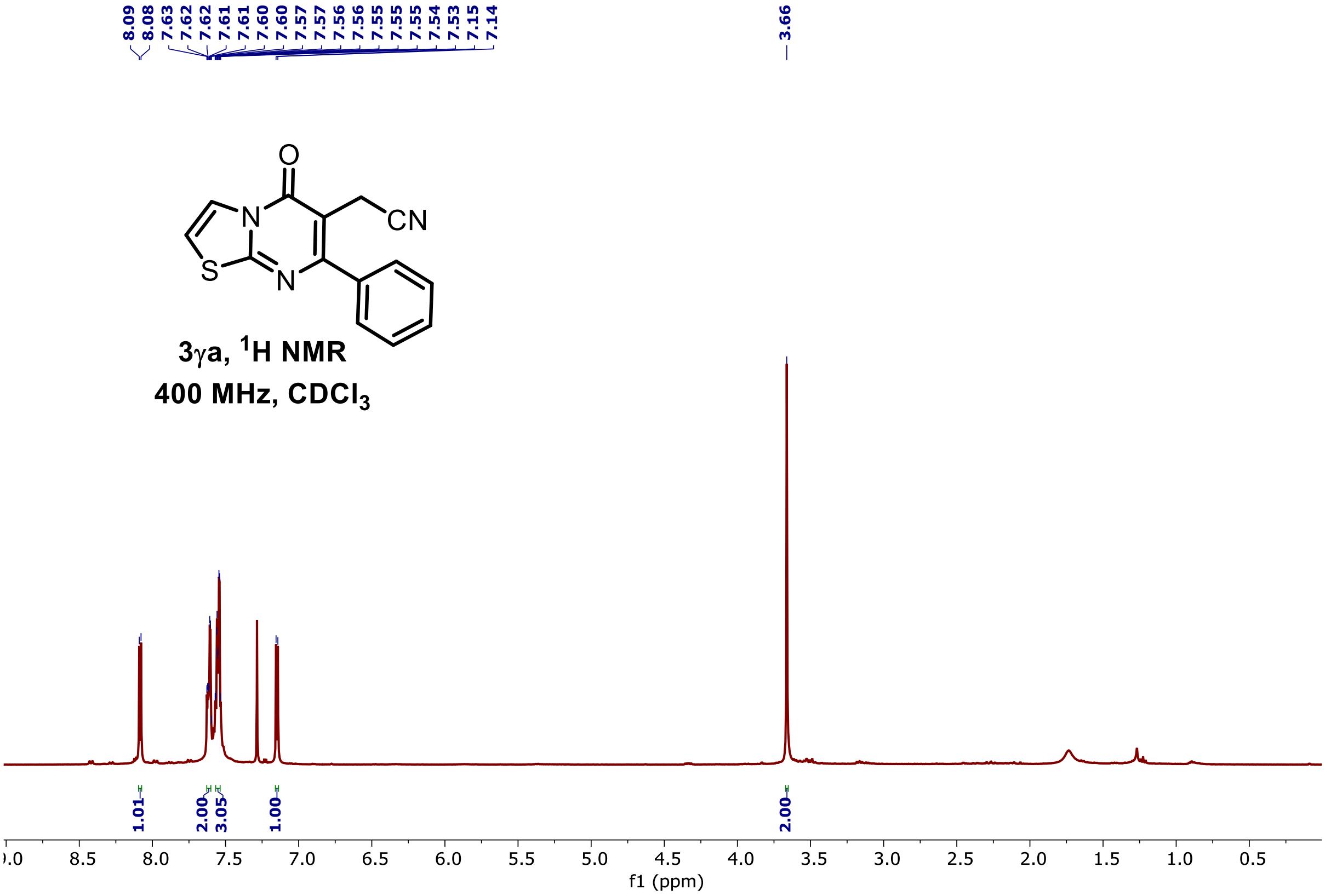

3γa, ^{1}H NMR
400 MHz, $CDCl_3$

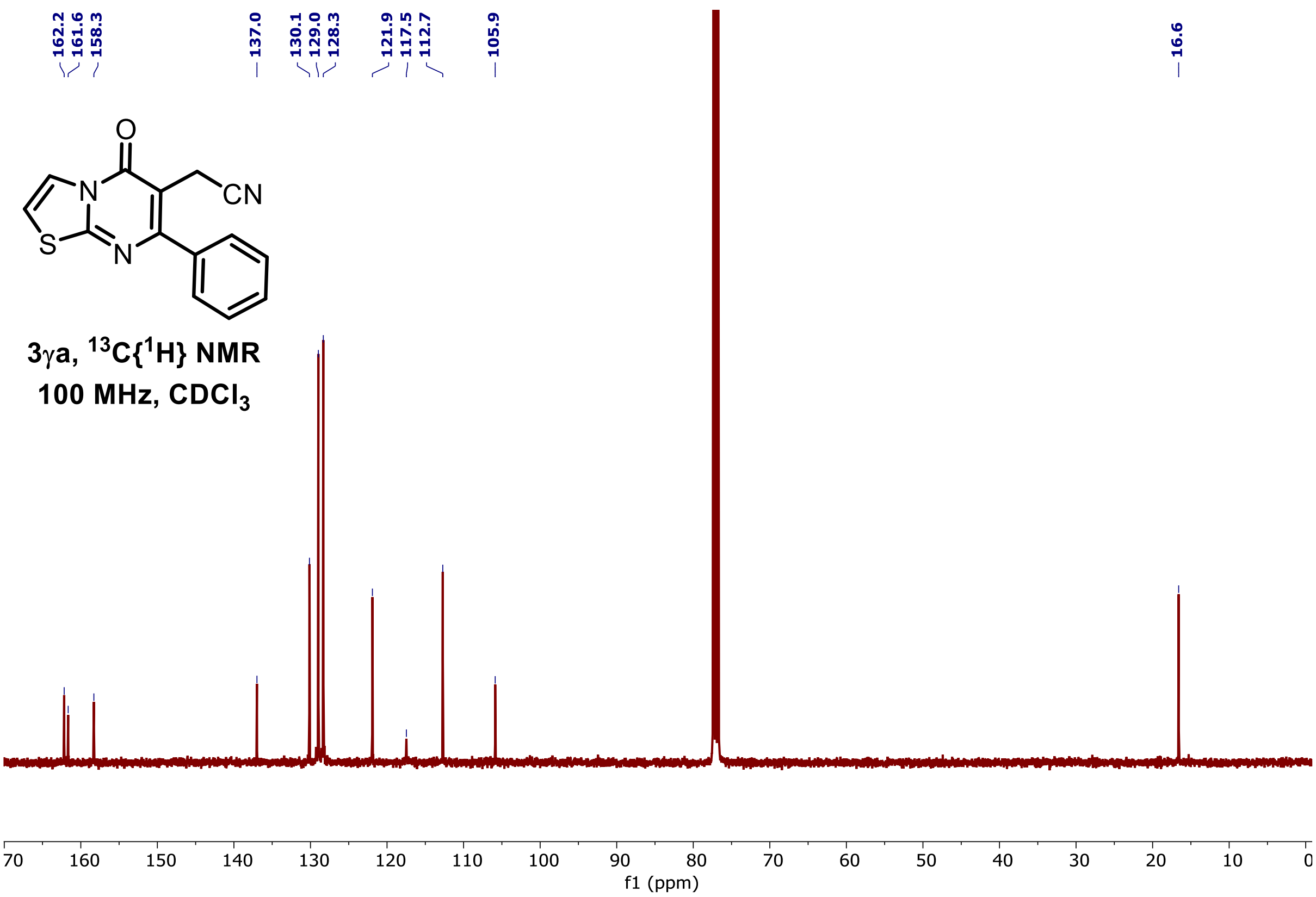

3γa, $^{13}C\{^1H\}$ NMR
100 MHz, $CDCl_3$

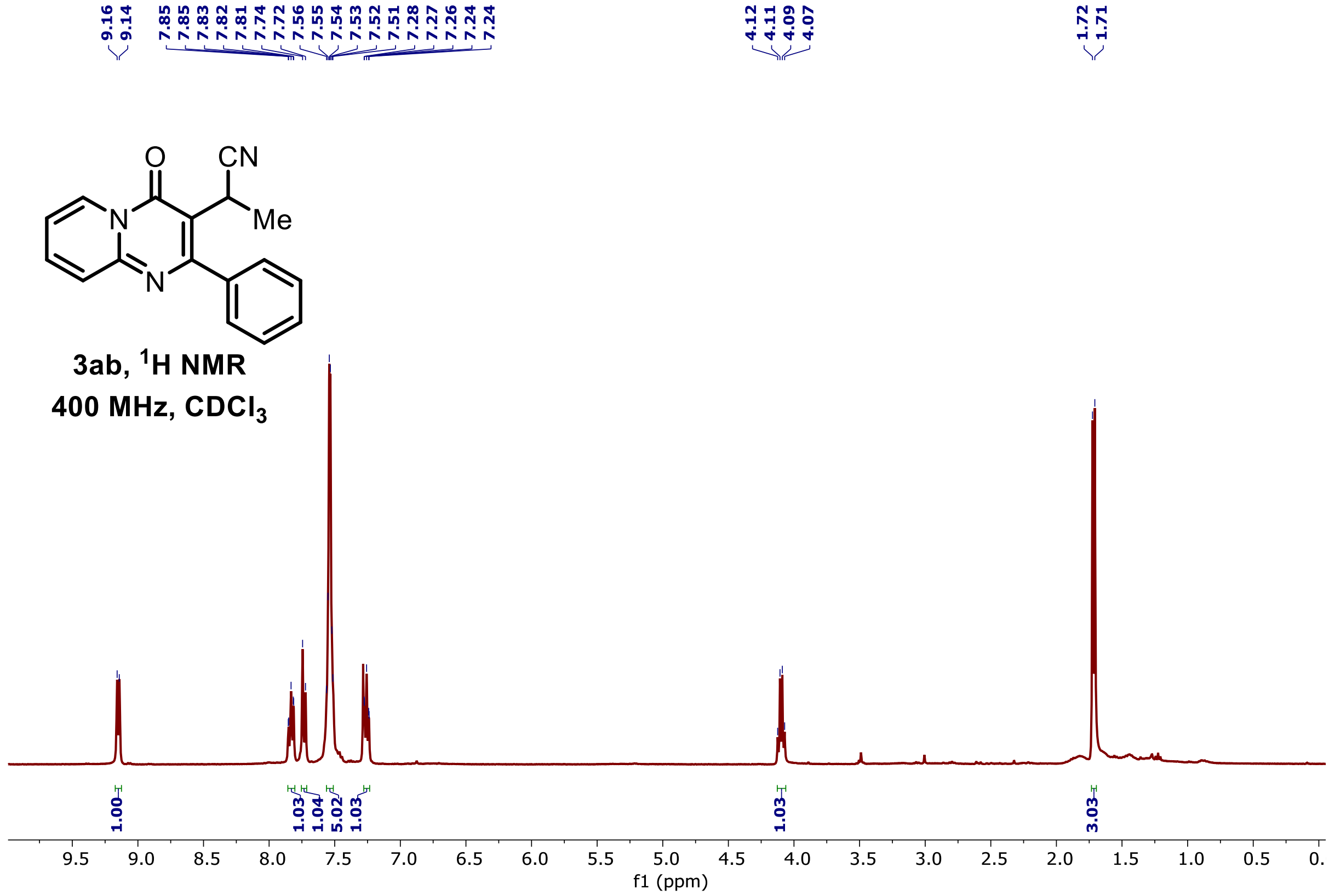
9.16
9.14
7.85
7.85
7.83
7.82
7.81
7.74
7.72
7.56
7.55
7.54
7.53
7.52
7.51
7.28
7.27
7.26
7.24
7.24
4.12
4.11
4.09
4.07
1.72
1.71
O
CN
N
Me
N
3ab, 1H NMR
400 MHz, CDCl3
1.00
1.03
1.04
5.02
1.03
1.03
3.03
9.5 9.0 8.5 8.0 7.5 7.0 6.5 6.0 5.5 5.0 4.5 4.0 3.5 3.0 2.5 2.0 1.5 1.0 0.5 0.
f1 (ppm)

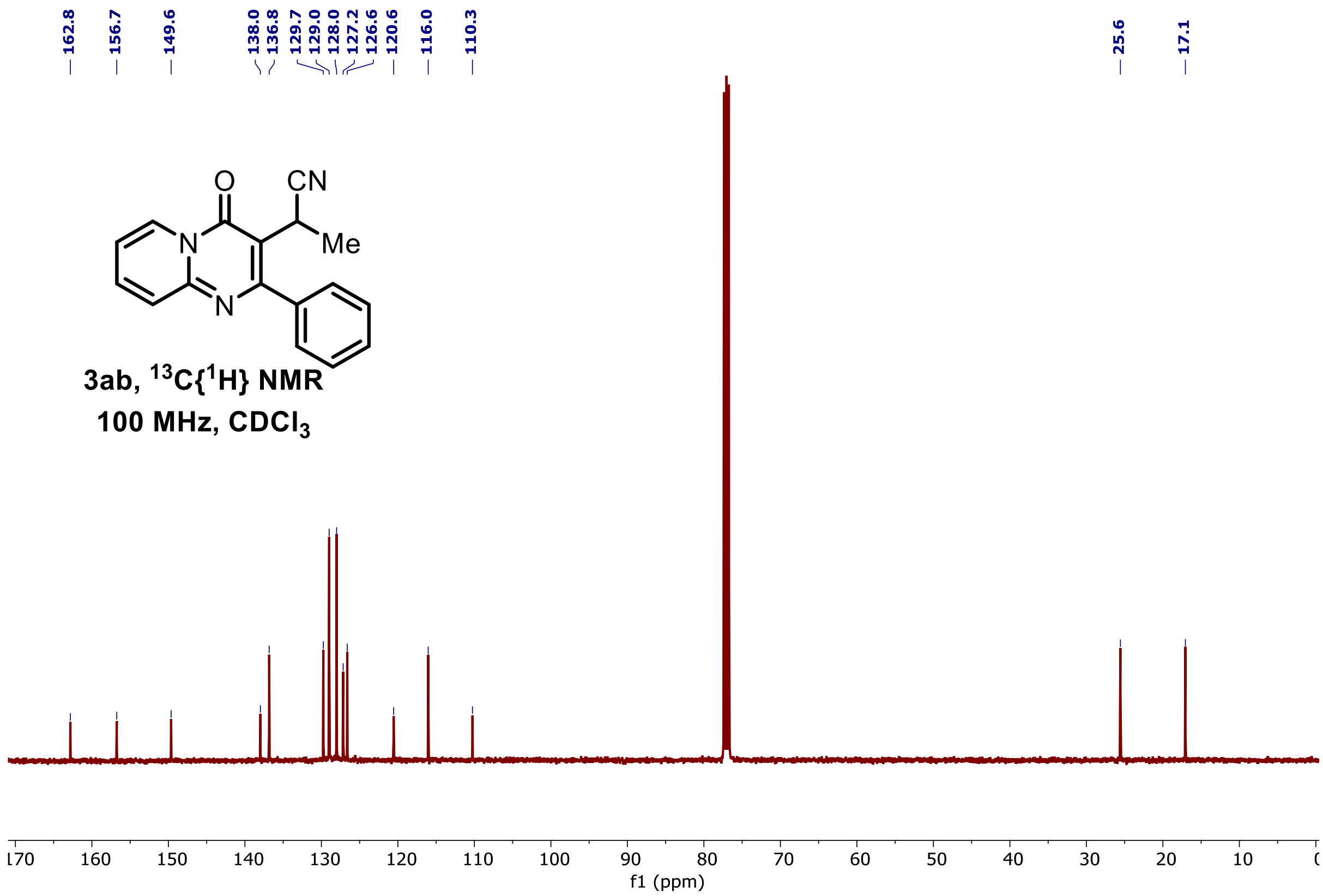
162.8
156.7
149.6
138.0
136.8
129.7
129.0
128.0
127.2
126.6
120.6
116.0
110.3
25.6
17.1
O
CN
N
Me
N
3ab, 13C{1H} NMR
100 MHz, CDCl3
170 160 150 140 130 120 110 100 90 80 70 60 50 40 30 20 10 0
f1 (ppm)

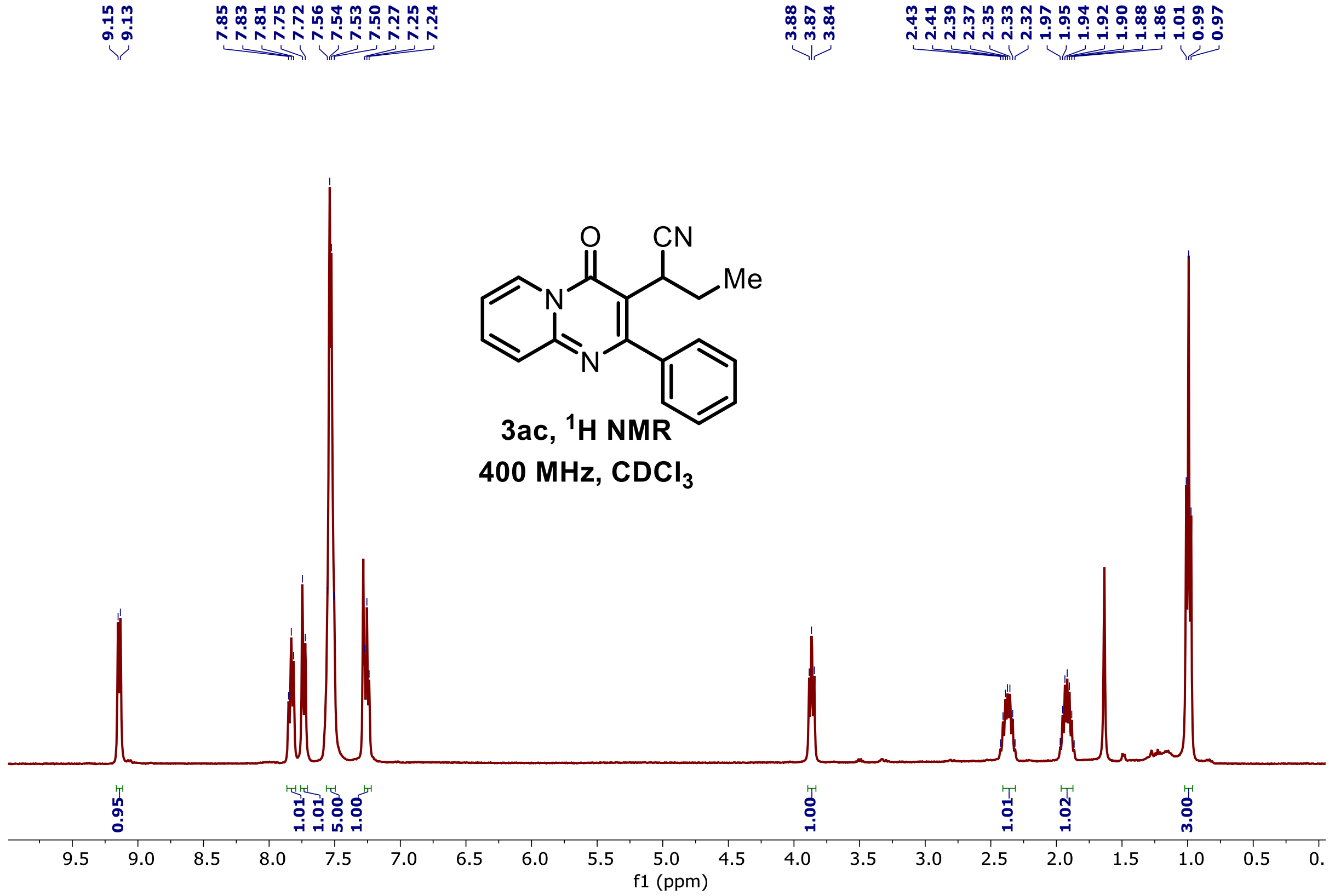
9.15 9.13 7.85 7.83 7.81 7.75 7.72 7.56 7.54 7.53 7.50 7.27 7.25 7.24 3.88 3.87 3.84 2.43 2.41 2.39 2.37 2.35 2.33 2.32 1.97 1.95 1.94 1.92 1.90 1.88 1.86 1.01 0.99 0.97
O
CN
Me
N
N
3ac, 1H NMR
400 MHz, CDCl3
0.95 1.01 1.01 5.00 1.00 1.00 1.01 1.02 3.00
f1 (ppm)

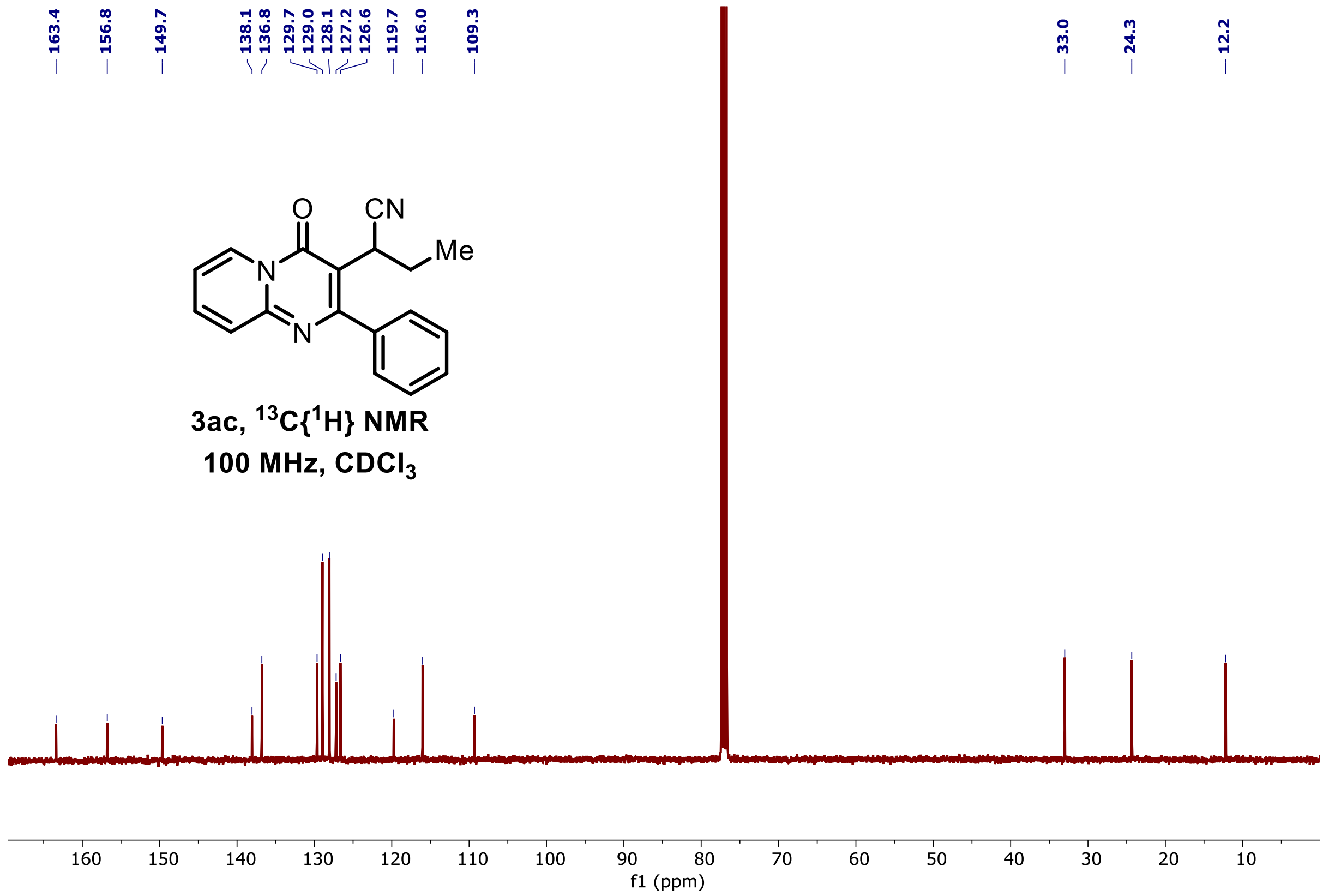
163.4 156.8 149.7 138.1 136.8 129.7 129.0 128.1 127.2 126.6 119.7 116.0 109.3 33.0 24.3 12.2
O
CN
Me
N
N
3ac, 13C{1H} NMR
100 MHz, CDCl3
f1 (ppm)

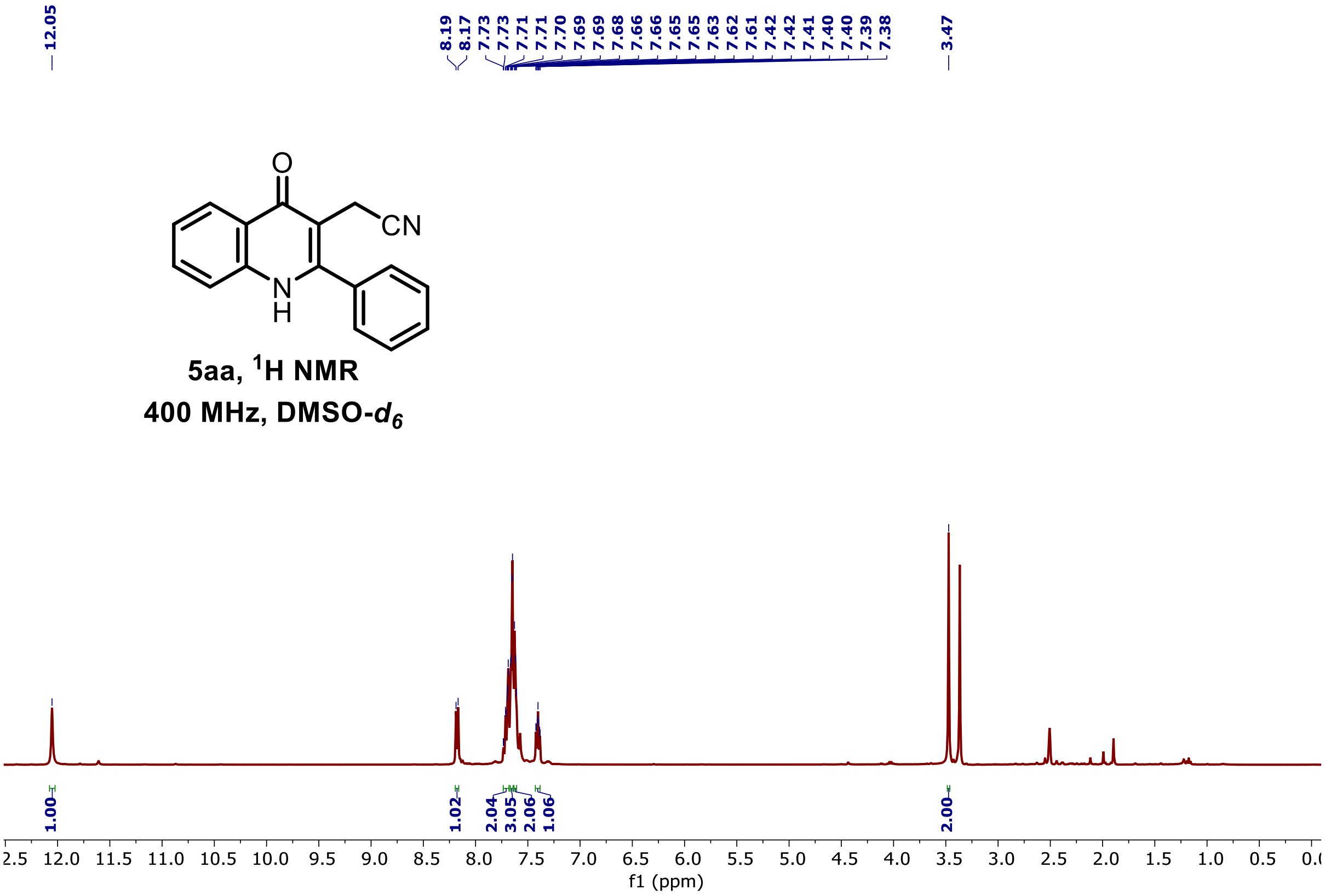
5aa, ¹H NMR
400 MHz, DMSO-d6
f1 (ppm)

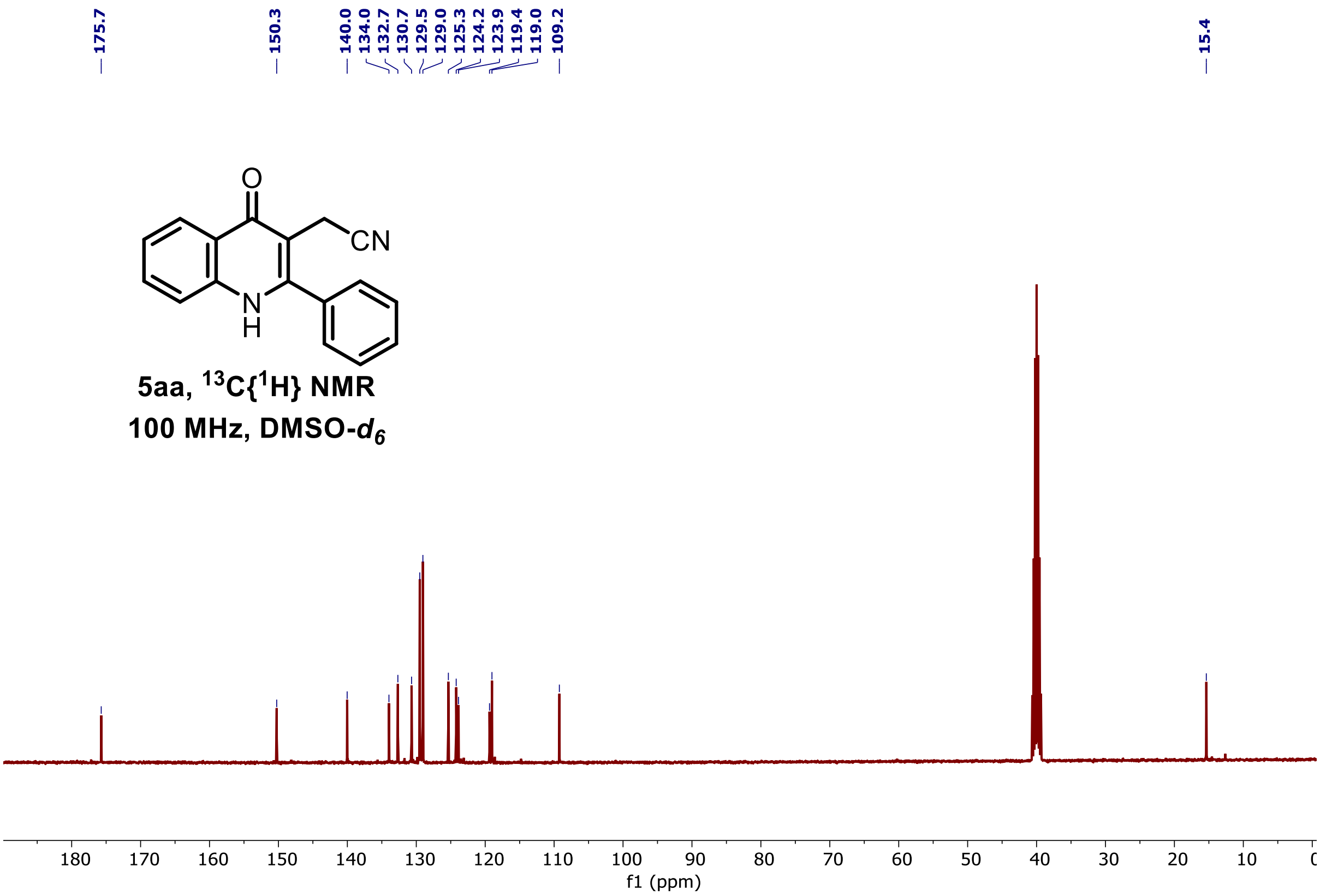
5aa, ¹³C{¹H} NMR
100 MHz, DMSO-d6
f1 (ppm)

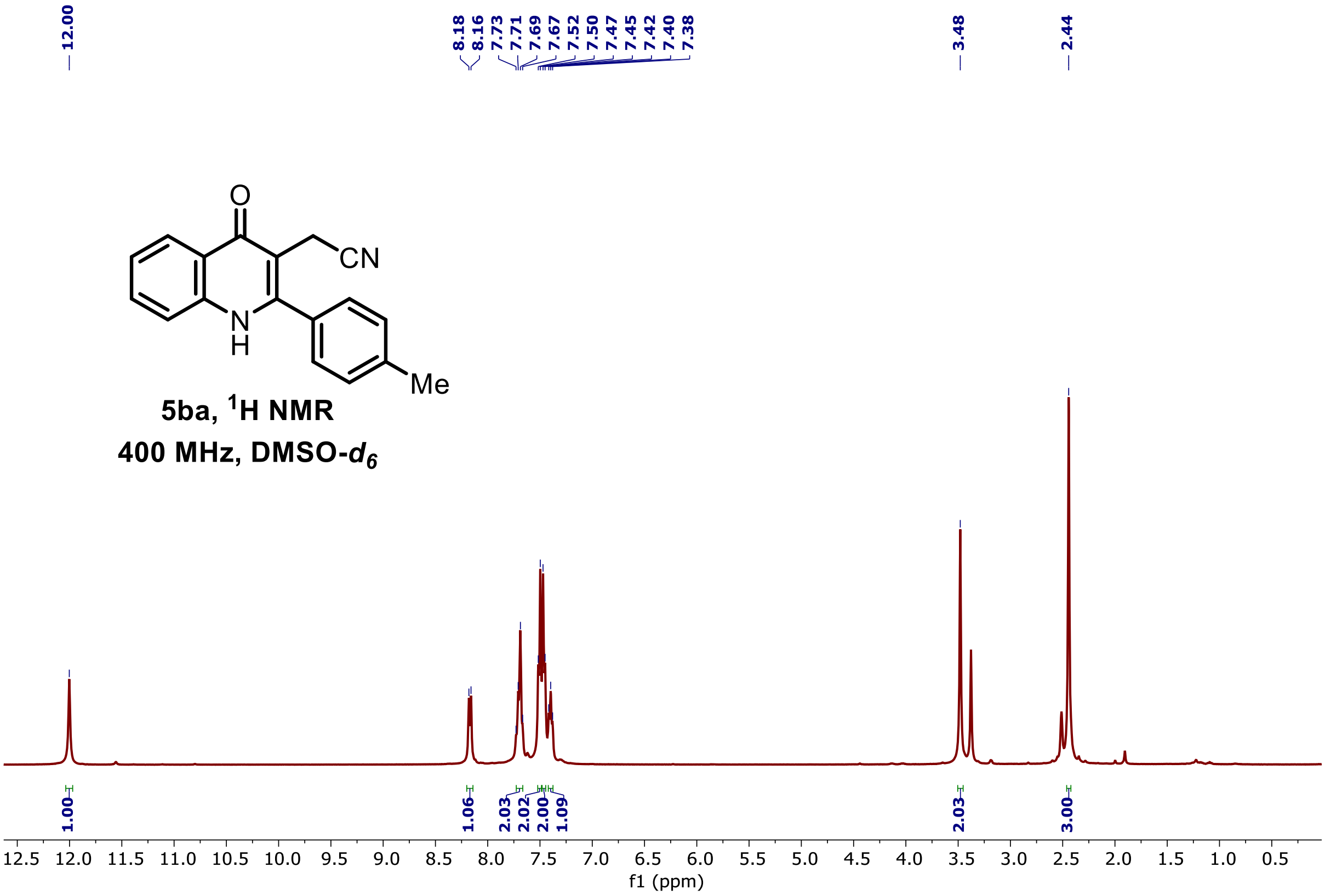
12.00
8.18
8.16
7.73
7.71
7.69
7.67
7.52
7.50
7.47
7.45
7.42
7.40
7.38
3.48
2.44
O
CN
N
H
Me
5ba, ^{1}H NMR
400 MHz, DMSO-d_6
1.00
1.06
2.03
2.02
2.00
1.09
2.03
3.00
12.5 12.0 11.5 11.0 10.5 10.0 9.5 9.0 8.5 8.0 7.5 7.0 6.5 6.0 5.5 5.0 4.5 4.0 3.5 3.0 2.5 2.0 1.5 1.0 0.5
f1 (ppm)

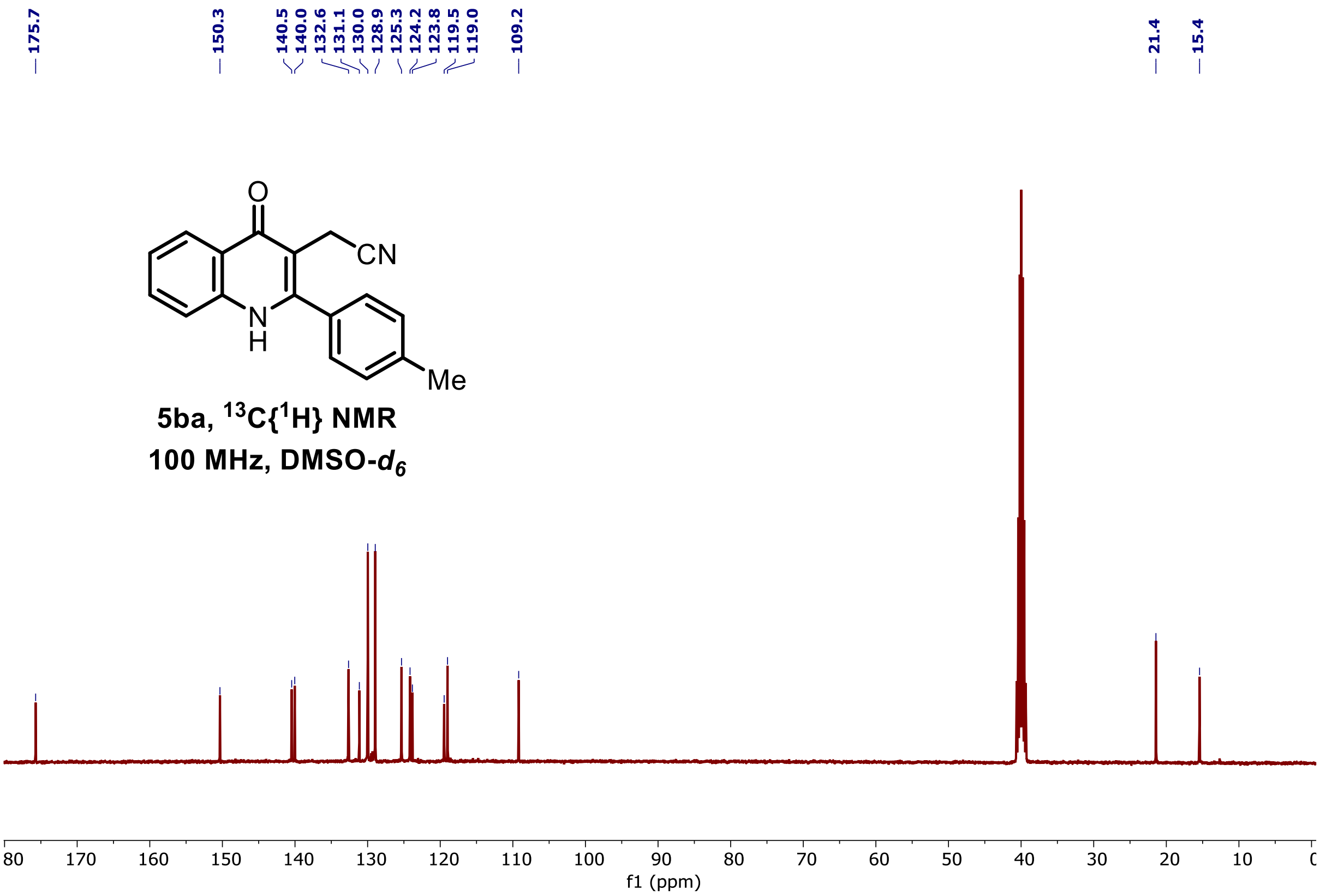
175.7
150.3
140.5
140.0
132.6
131.1
130.0
128.9
125.3
124.2
123.8
119.5
119.0
109.2
21.4
15.4
O
CN
N
H
Me
5ba, ^{13}C{^{1}H} NMR
100 MHz, DMSO-d_6
80 170 160 150 140 130 120 110 100 90 80 70 60 50 40 30 20 10 0
f1 (ppm)

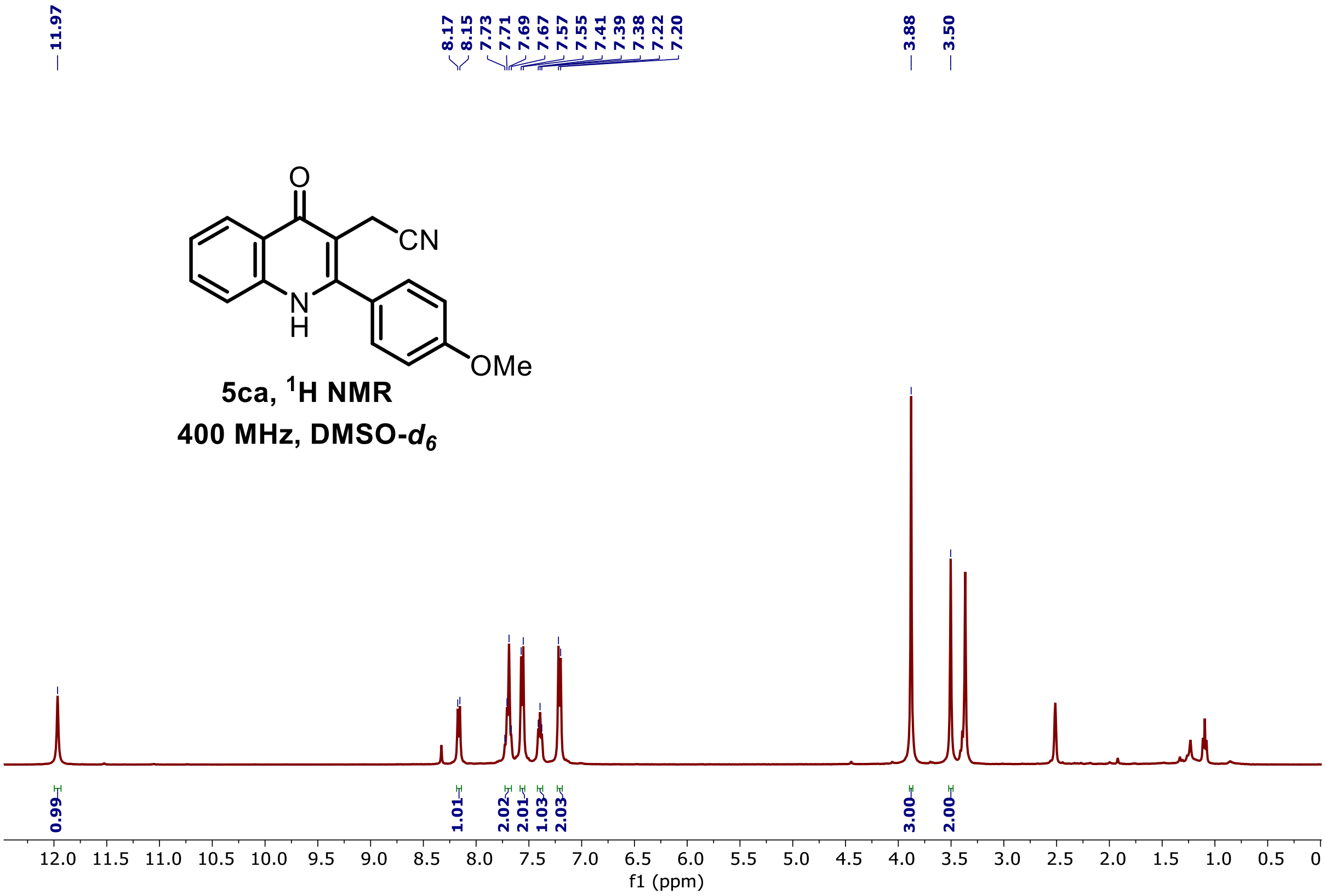
11.97
8.17
8.15
7.73
7.71
7.69
7.67
7.57
7.55
7.41
7.39
7.38
7.22
7.20
3.88
3.50
O
CN
N
H
OMe
5ca, 1H NMR
400 MHz, DMSO-d6
0.99
1.01
2.02
2.01
1.03
2.03
3.00
2.00
12.0 11.5 11.0 10.5 10.0 9.5 9.0 8.5 8.0 7.5 7.0 6.5 6.0 5.5 5.0 4.5 4.0 3.5 3.0 2.5 2.0 1.5 1.0 0.5 0
f1 (ppm)

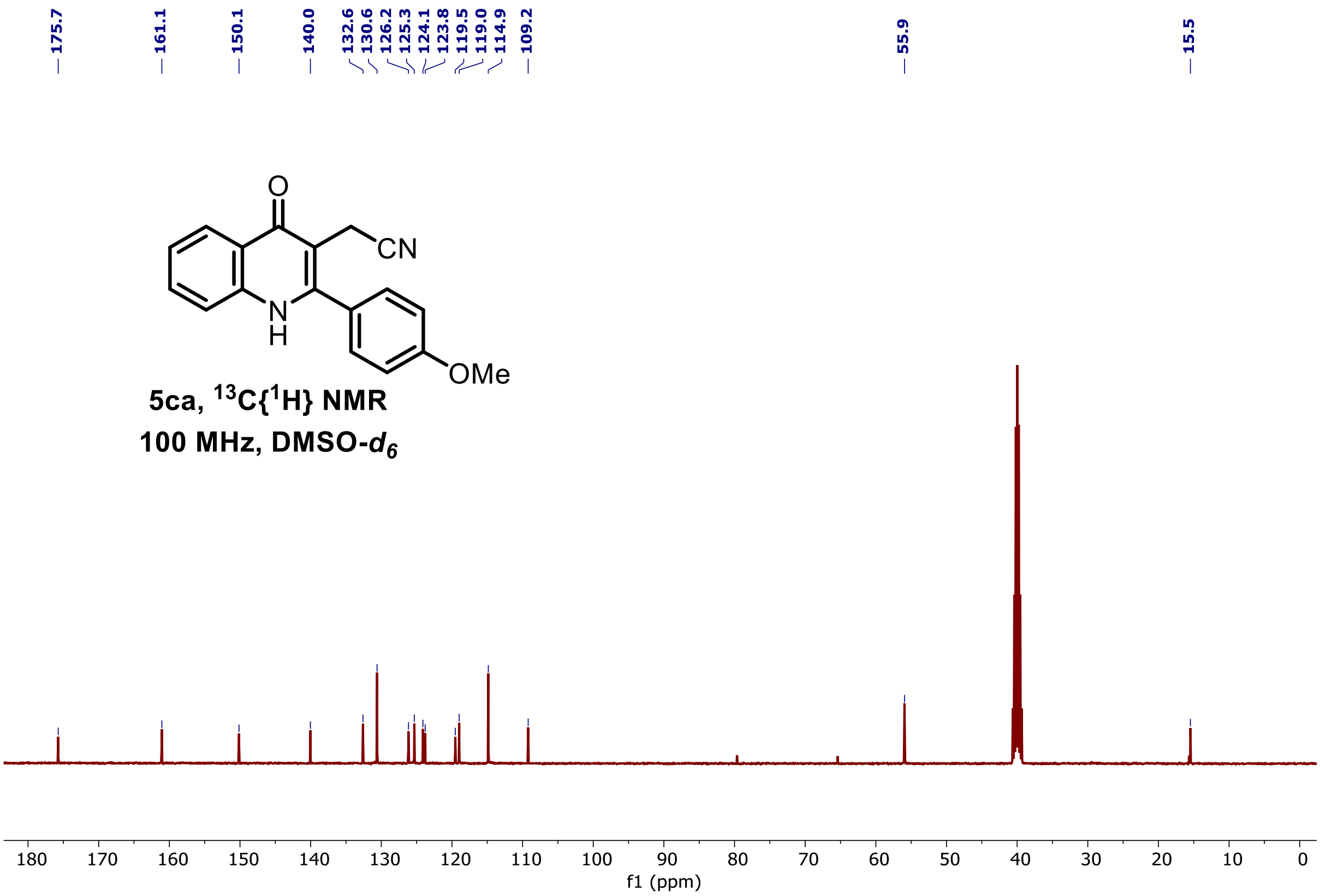
175.7
161.1
150.1
140.0
132.6
130.6
126.2
125.3
124.1
123.8
119.5
119.0
114.9
109.2
55.9
15.5
O
CN
N
H
OMe
5ca, 13C{1H} NMR
100 MHz, DMSO-d6
180 170 160 150 140 130 120 110 100 90 80 70 60 50 40 30 20 10 0
f1 (ppm)

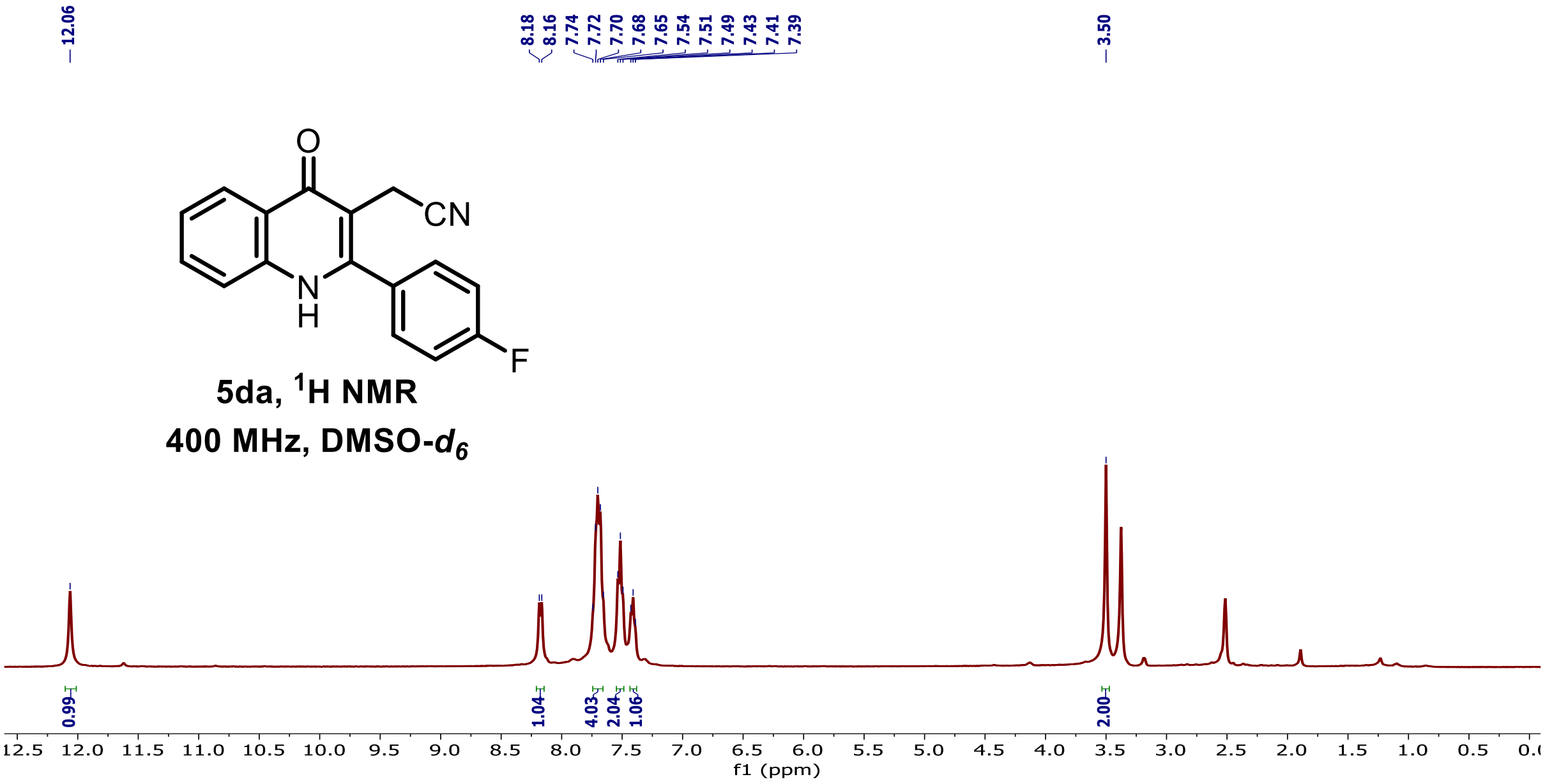
12.06
8.18
8.16
7.74
7.72
7.70
7.68
7.65
7.54
7.51
7.49
7.43
7.41
7.39
3.50
5da, 1H NMR
400 MHz, DMSO-d6
0.99
1.04
4.03
2.04
1.06
2.00
f1 (ppm)

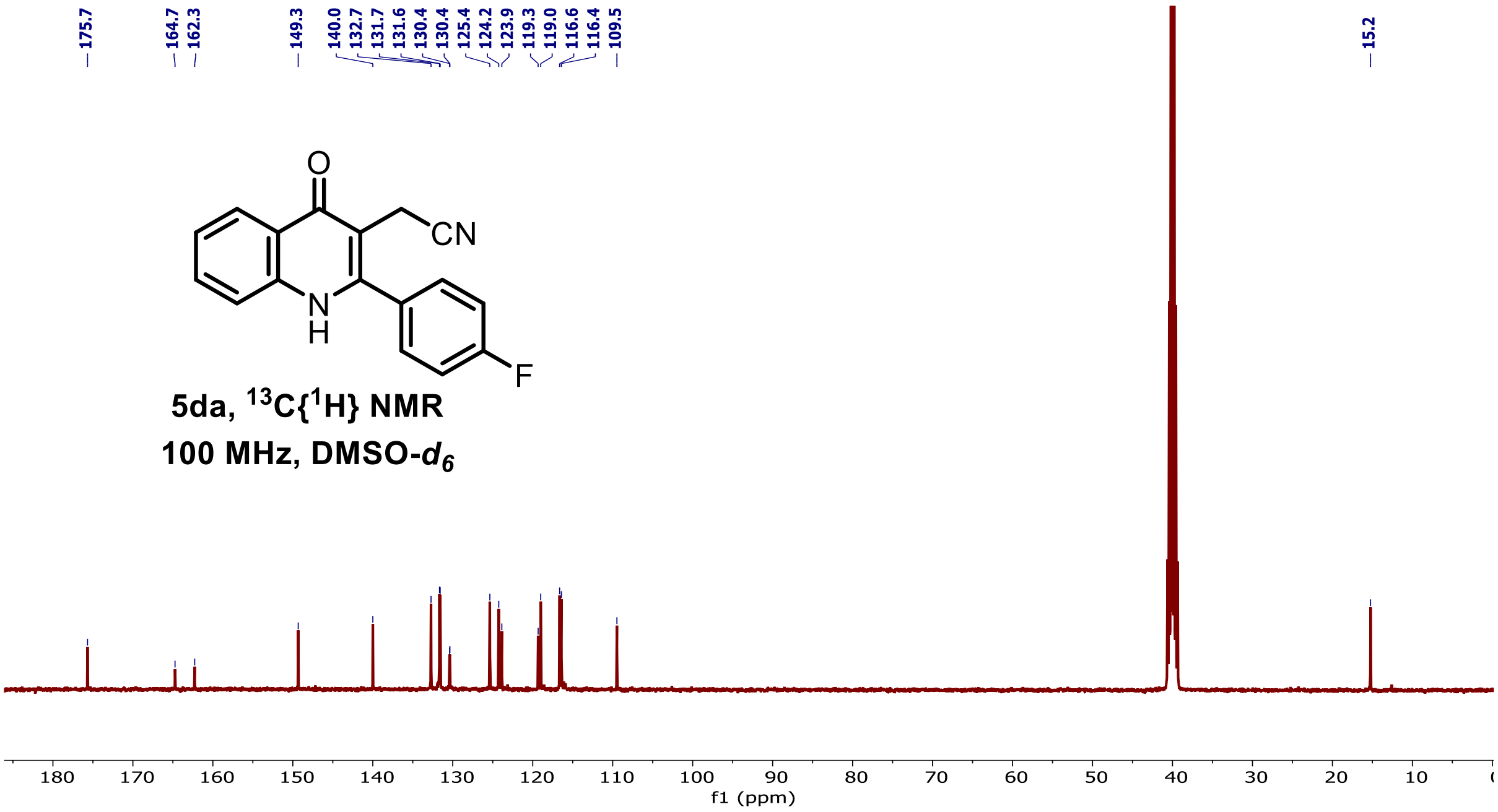
175.7
164.7
162.3
149.3
140.0
132.7
131.7
131.6
130.4
130.4
125.4
124.2
123.9
119.3
119.0
116.6
116.4
109.5
15.2
5da, 13C{1H} NMR
100 MHz, DMSO-d6
f1 (ppm)

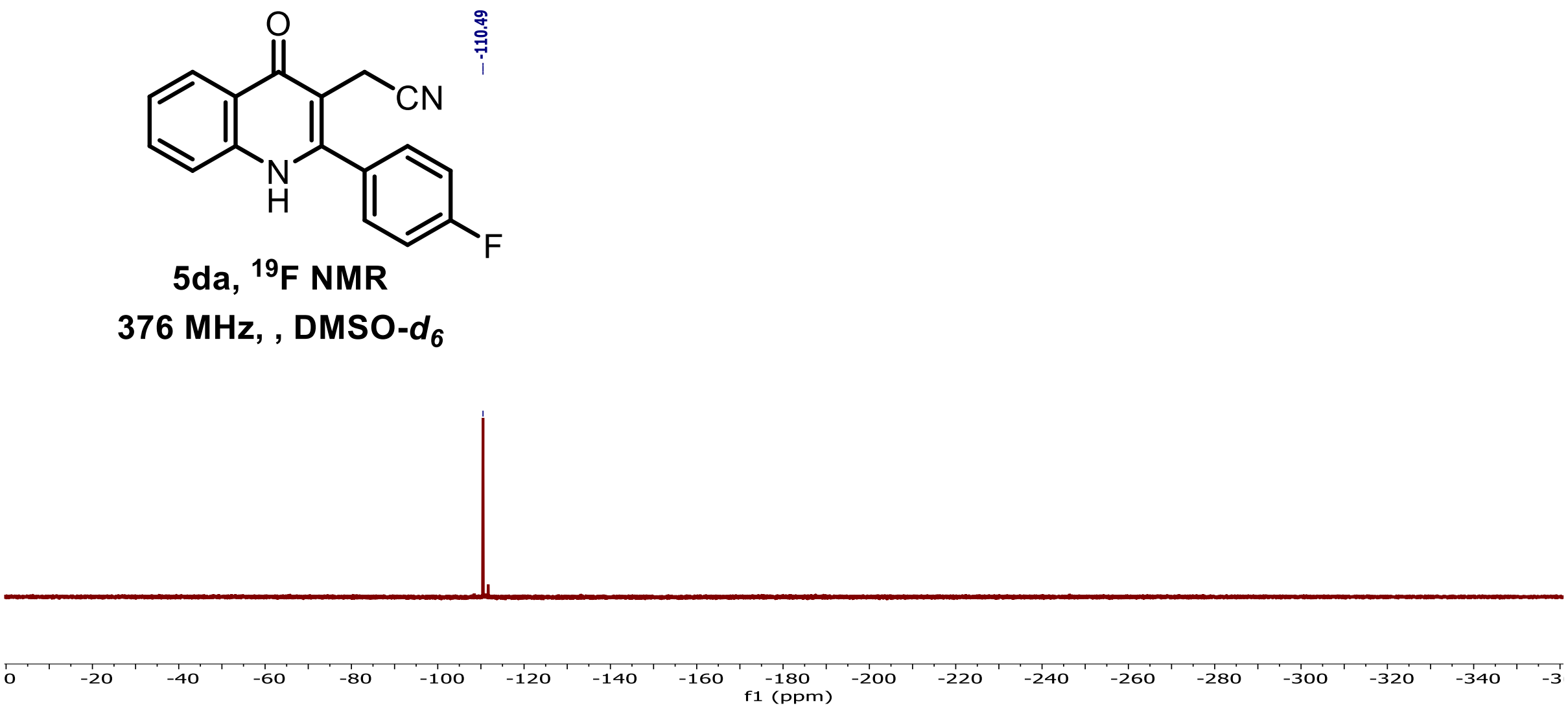
-110.49
5da, 19F NMR
376 MHz, , DMSO-d6
f1 (ppm)

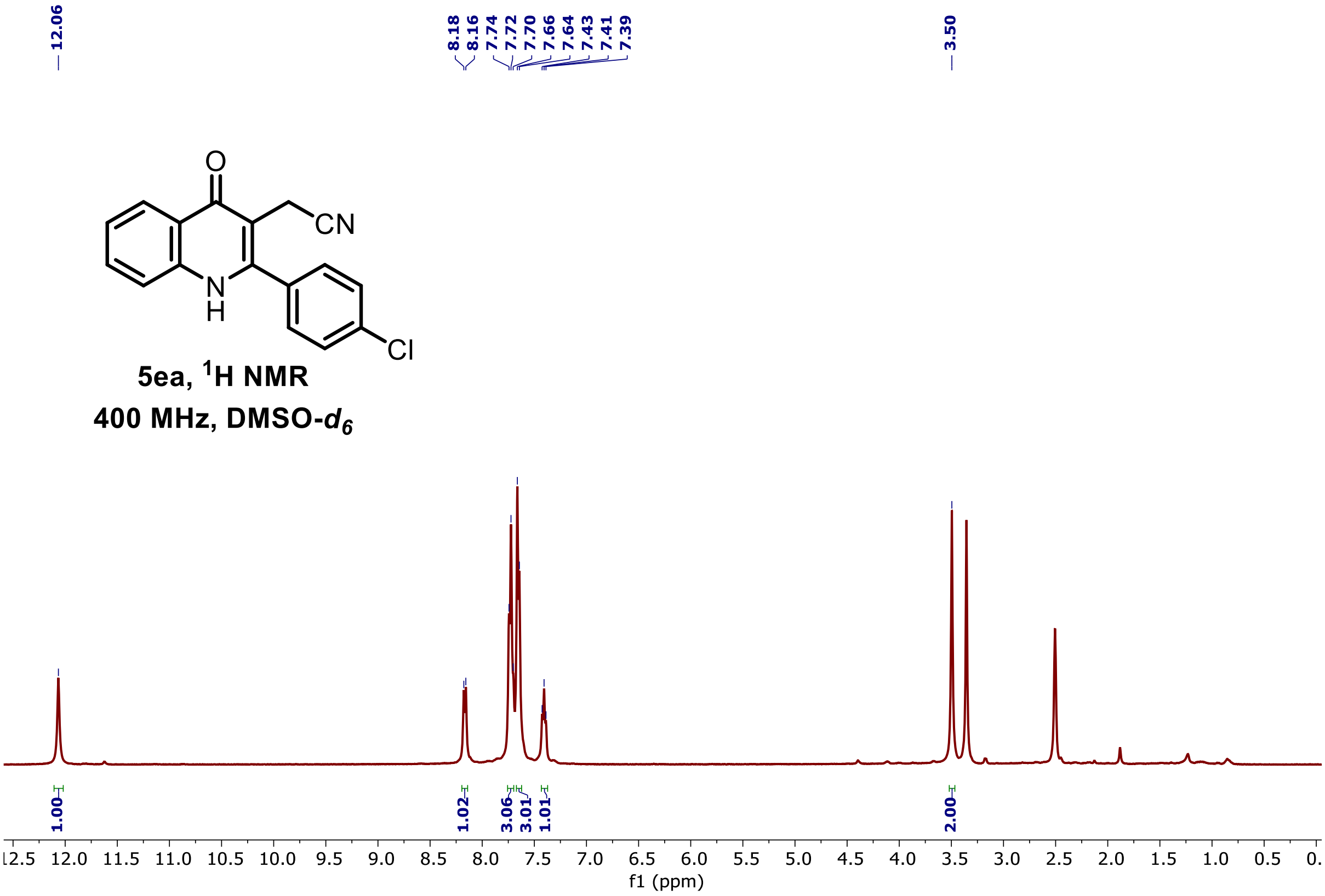
12.06
8.18
8.16
7.74
7.72
7.70
7.66
7.64
7.43
7.41
7.39
3.50
O
CN
N
H
Cl
5ea, ^{1}H NMR
400 MHz, DMSO-d_6
1.00
1.02
3.06
3.01
1.01
2.00
f1 (ppm)

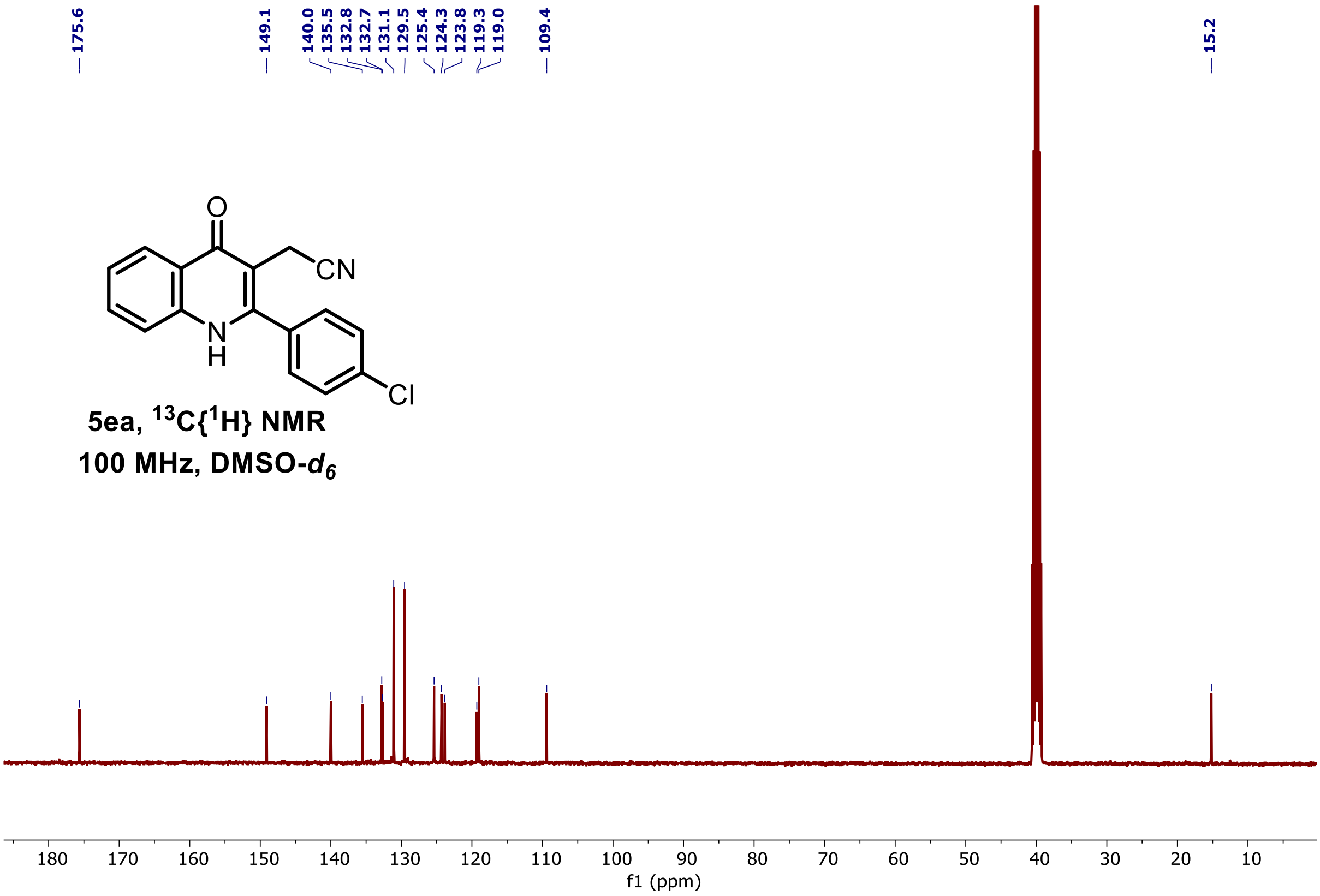
175.6
149.1
140.0
135.5
132.8
132.7
131.1
129.5
125.4
124.3
123.8
119.3
119.0
109.4
15.2
O
CN
N
H
Cl
5ea, ^{13}C{^{1}H} NMR
100 MHz, DMSO-d_6
f1 (ppm)

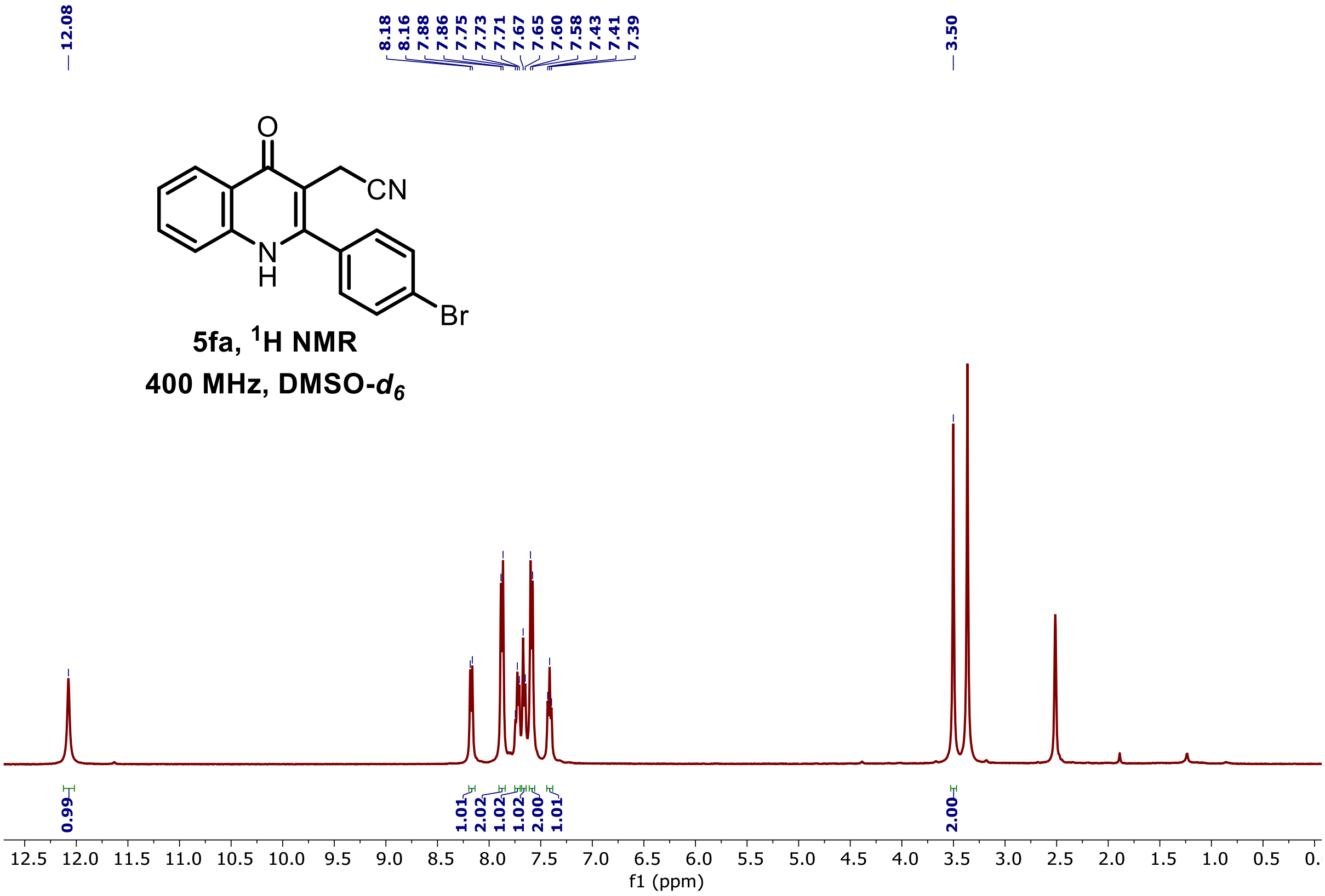

12.08
8.18 8.16 7.88 7.86 7.75 7.73 7.71 7.67 7.65 7.60 7.58 7.43 7.41 7.39
3.50
O
CN
N
H
Br
5fa, ^{1}H NMR
400 MHz, DMSO-d_6
0.99
1.01 2.02 1.02 1.02 2.00 1.01
2.00
12.5 12.0 11.5 11.0 10.5 10.0 9.5 9.0 8.5 8.0 7.5 7.0 6.5 6.0 5.5 5.0 4.5 4.0 3.5 3.0 2.5 2.0 1.5 1.0 0.5 0.
f1 (ppm)

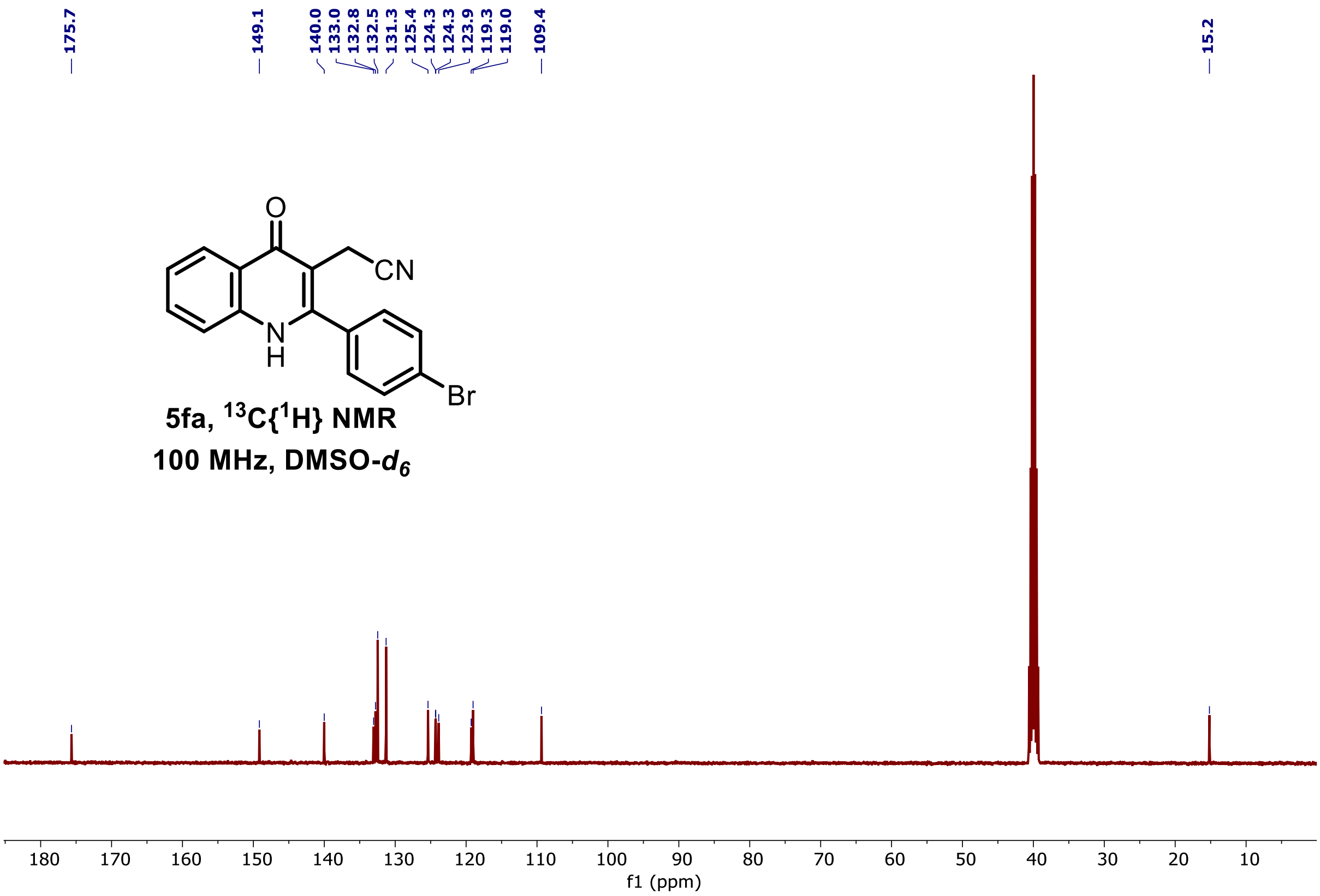

175.7
149.1
140.0 133.0 132.8 132.5 131.3 125.4 124.3 124.3 123.9 119.3 119.0
109.4
15.2
O
CN
N
H
Br
5fa, ^{13}C{^{1}H} NMR
100 MHz, DMSO-d_6
180 170 160 150 140 130 120 110 100 90 80 70 60 50 40 30 20 10
f1 (ppm)

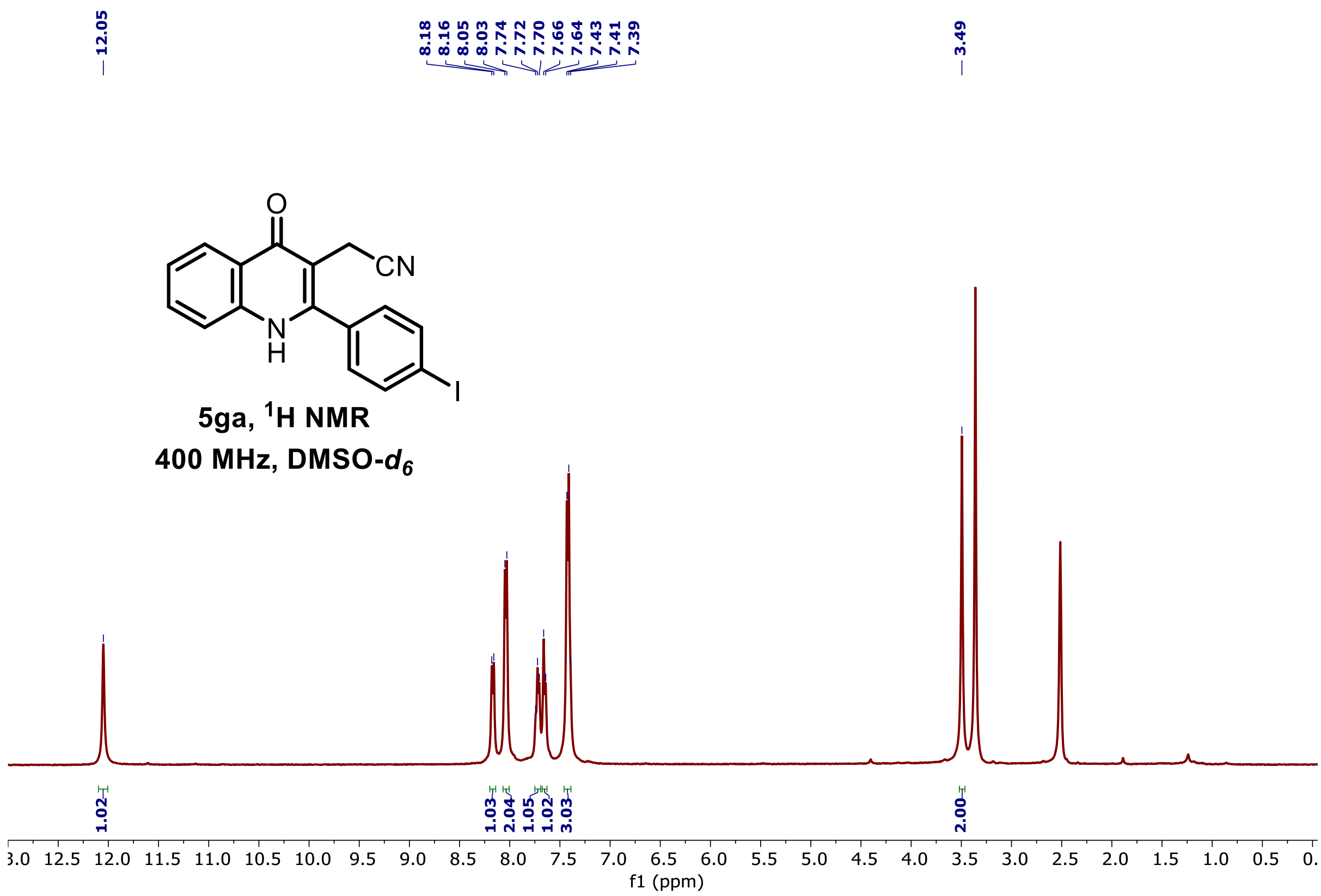
5ga, ¹H NMR
400 MHz, DMSO-d_6
12.05
8.18 8.16 8.05 8.03 7.74 7.72 7.70 7.66 7.64 7.43 7.41 7.39
3.49
1.02
1.03 2.04 1.05 1.02 3.03
2.00
f1 (ppm)

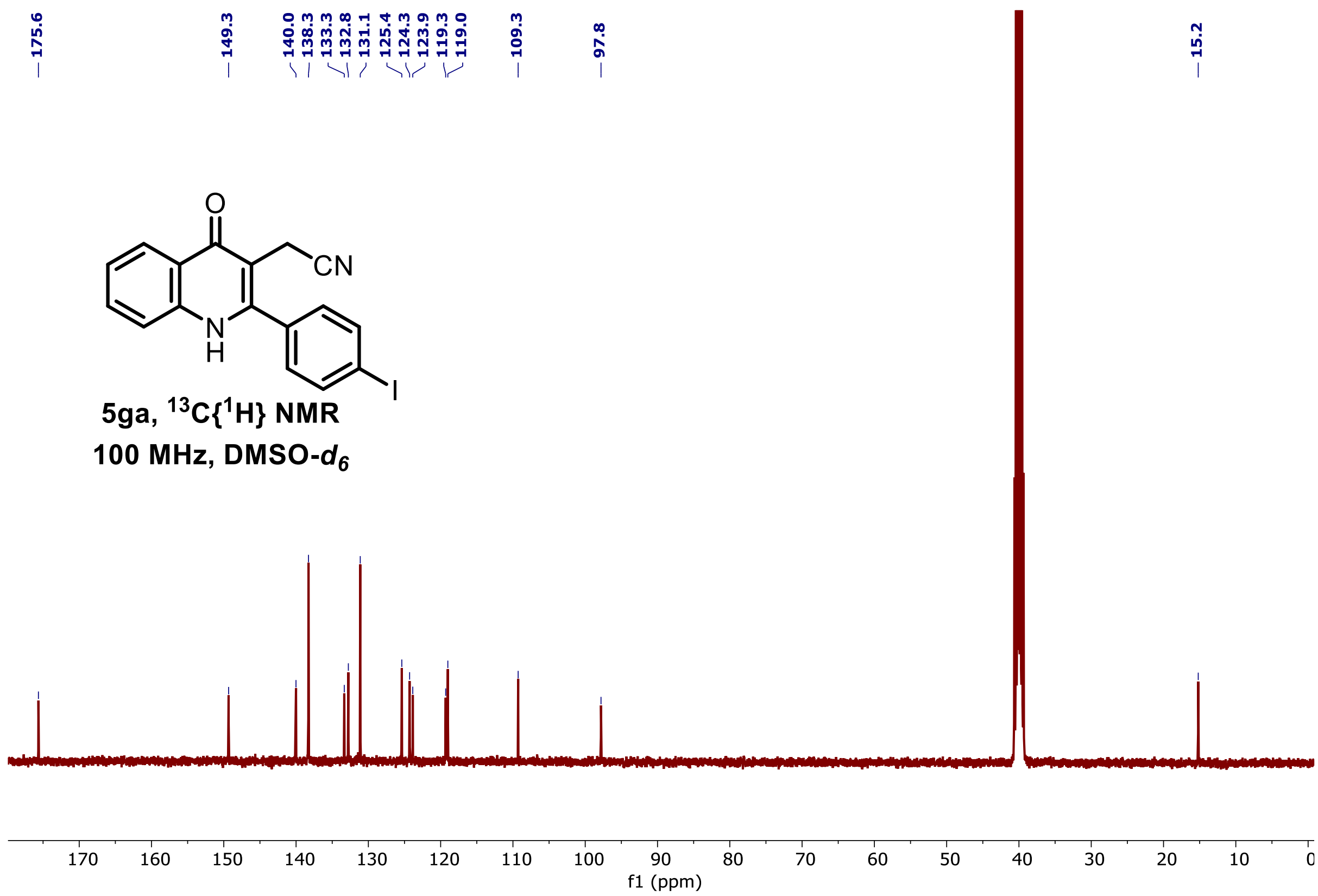
5ga, ¹³C{¹H} NMR
100 MHz, DMSO-d_6
175.6
149.3
140.0 138.3 133.3 132.8 131.1 125.4 124.3 123.9 119.3 119.0
109.3
97.8
15.2
f1 (ppm)

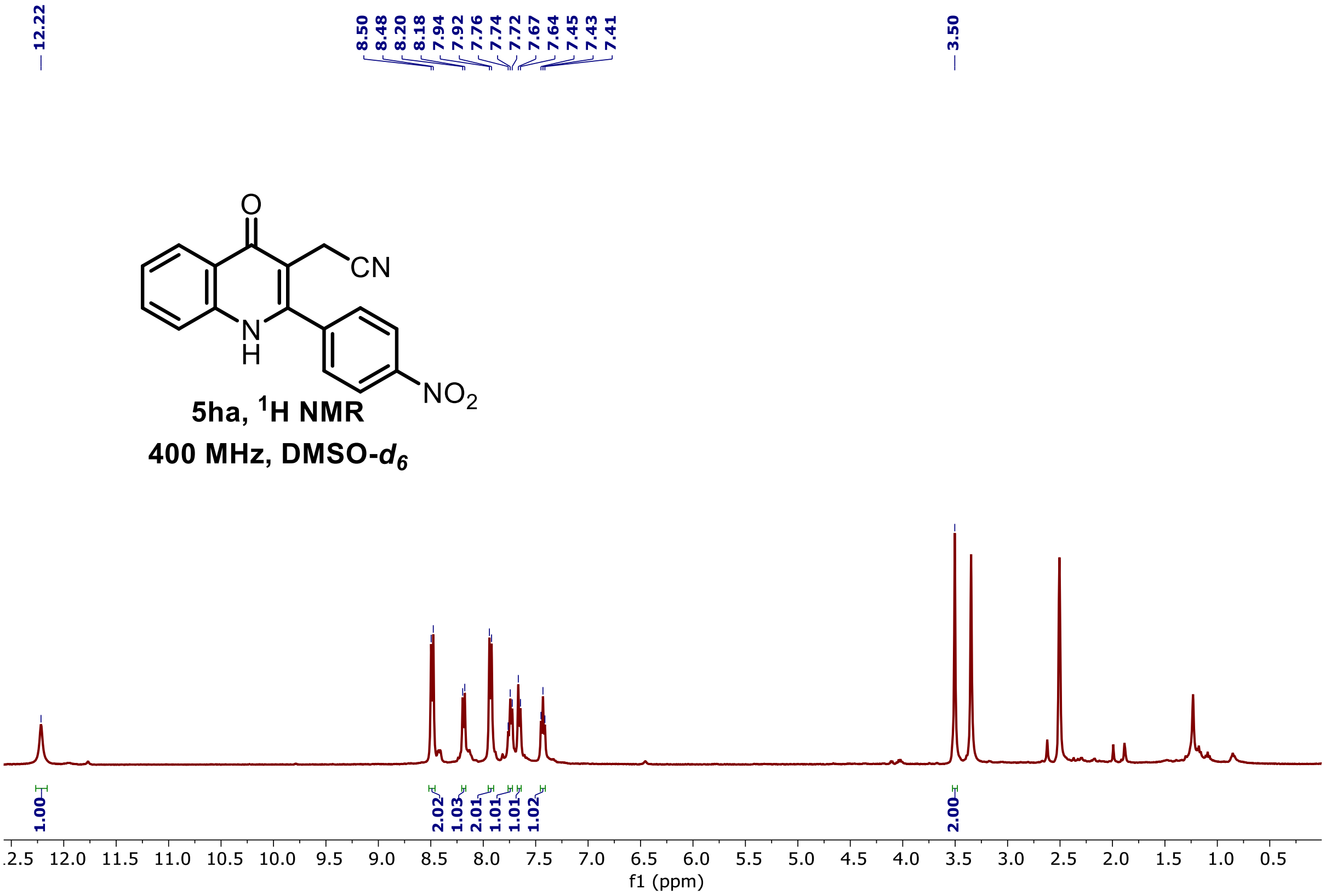
12.22
8.50 8.48 8.20 8.18 7.94 7.92 7.76 7.74 7.72 7.67 7.64 7.45 7.43 7.41
3.50
O
CN
N
H
NO2
5ha, 1H NMR
400 MHz, DMSO-d6
1.00
2.02 1.03 2.01 1.01 1.01 1.02
2.00
.2.5 12.0 11.5 11.0 10.5 10.0 9.5 9.0 8.5 8.0 7.5 7.0 6.5 6.0 5.5 5.0 4.5 4.0 3.5 3.0 2.5 2.0 1.5 1.0 0.5
f1 (ppm)

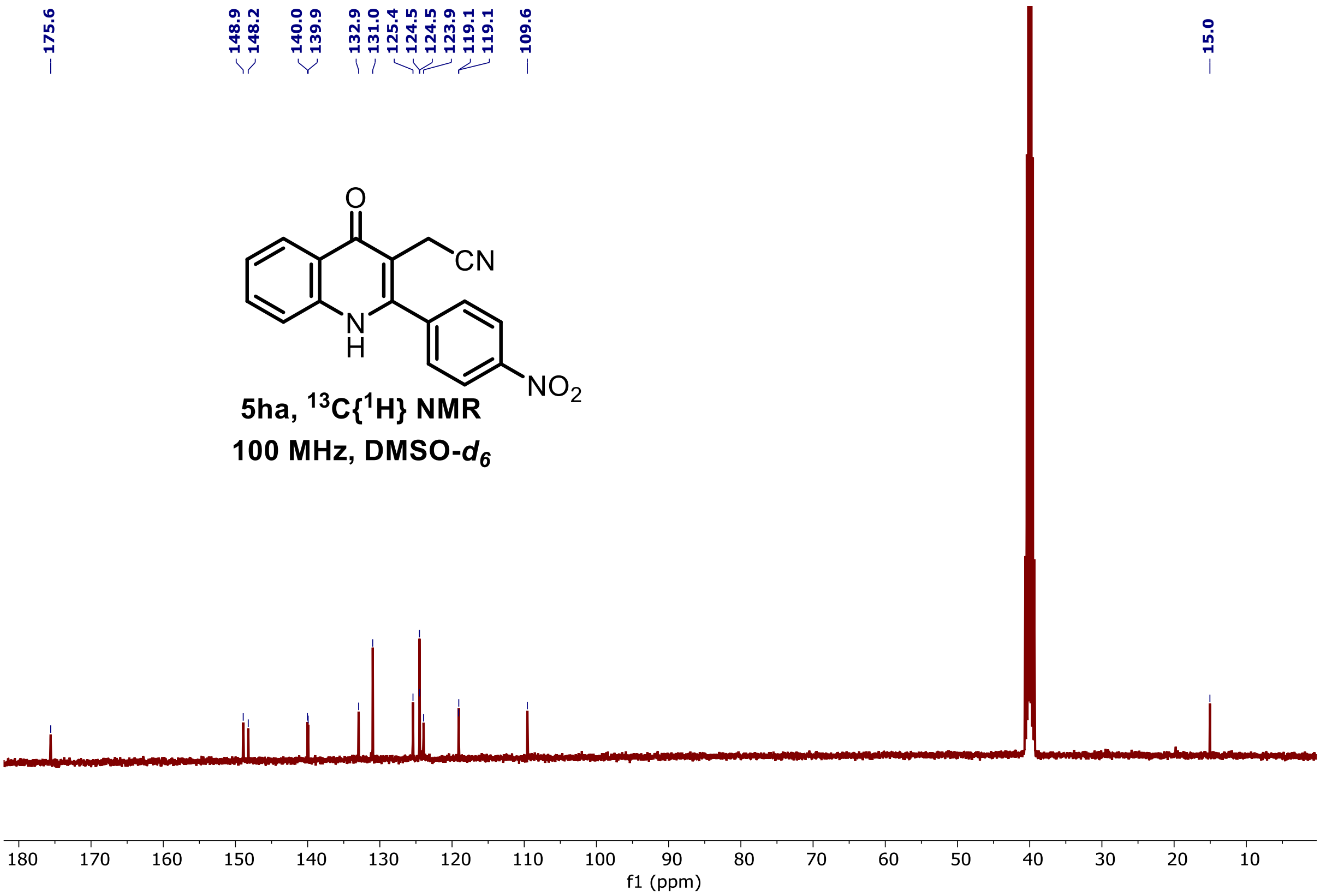
175.6
148.9 148.2
140.0 139.9
132.9 131.0 125.4 124.5 124.5 123.9 119.1 119.1
109.6
15.0
O
CN
N
H
NO2
5ha, 13C{1H} NMR
100 MHz, DMSO-d6
180 170 160 150 140 130 120 110 100 90 80 70 60 50 40 30 20 10
f1 (ppm)

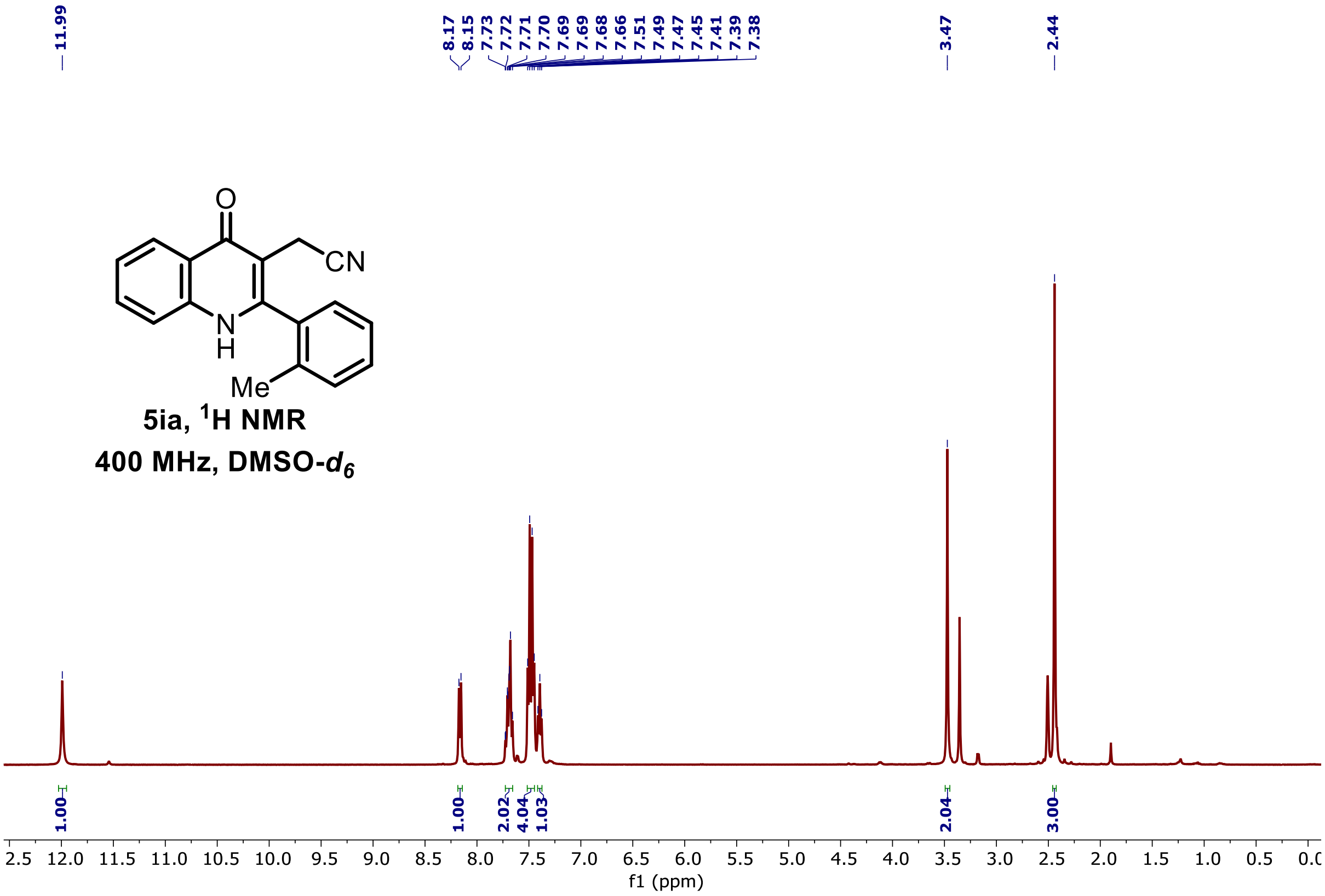
11.99
8.17
8.15
7.73
7.72
7.71
7.70
7.69
7.69
7.68
7.66
7.51
7.49
7.47
7.45
7.41
7.39
7.38
3.47
2.44
O
CN
N
H
Me
5ia, ^{1}H NMR
400 MHz, DMSO-d_6
1.00
1.00
2.02
4.04
1.03
2.04
3.00
2.5 12.0 11.5 11.0 10.5 10.0 9.5 9.0 8.5 8.0 7.5 7.0 6.5 6.0 5.5 5.0 4.5 4.0 3.5 3.0 2.5 2.0 1.5 1.0 0.5 0.0
f1 (ppm)

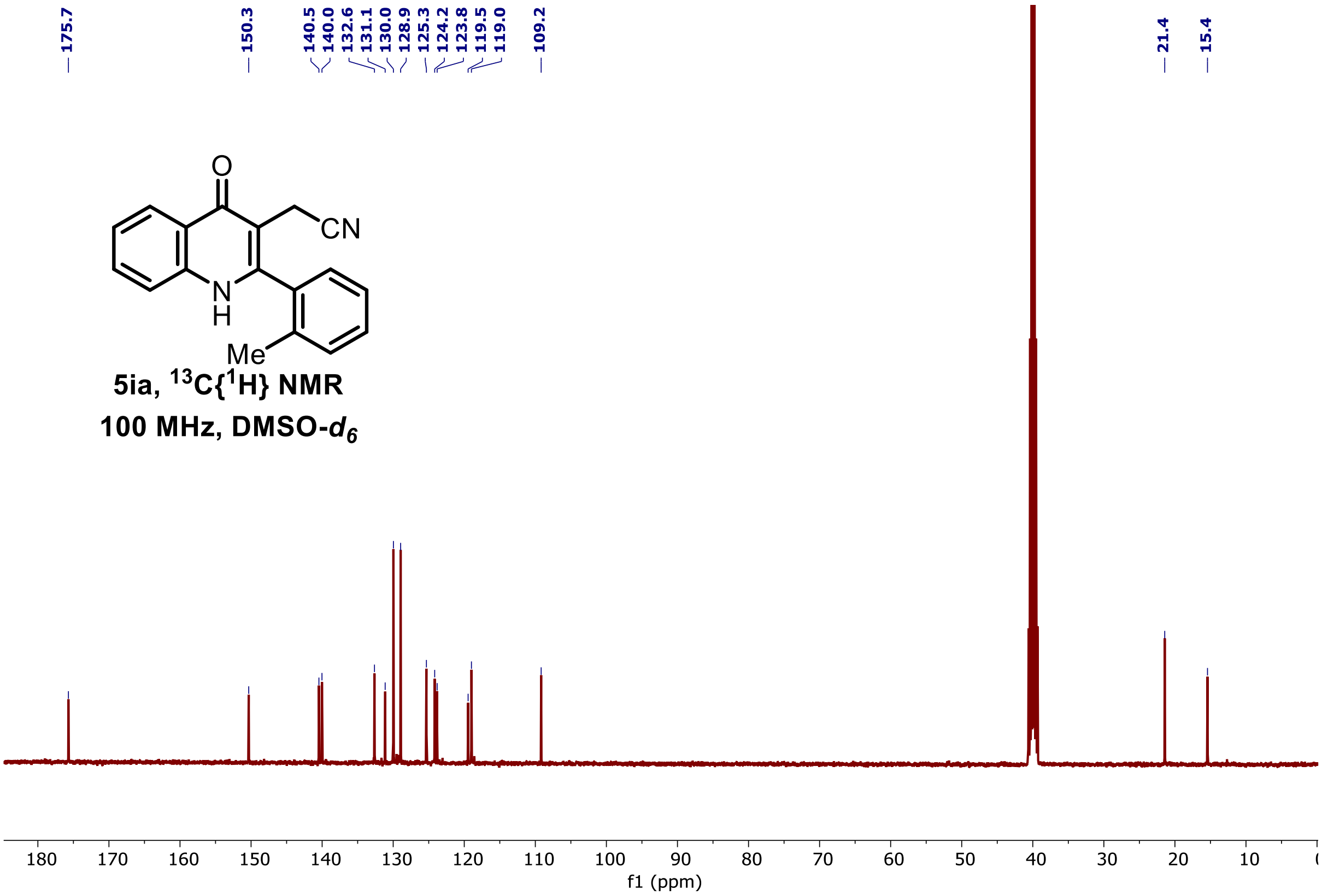
175.7
150.3
140.5
140.0
132.6
131.1
130.0
128.9
125.3
124.2
123.8
119.5
119.0
109.2
21.4
15.4
O
CN
N
H
Me
5ia, ^{13}C{^{1}H} NMR
100 MHz, DMSO-d_6
180 170 160 150 140 130 120 110 100 90 80 70 60 50 40 30 20 10
f1 (ppm)

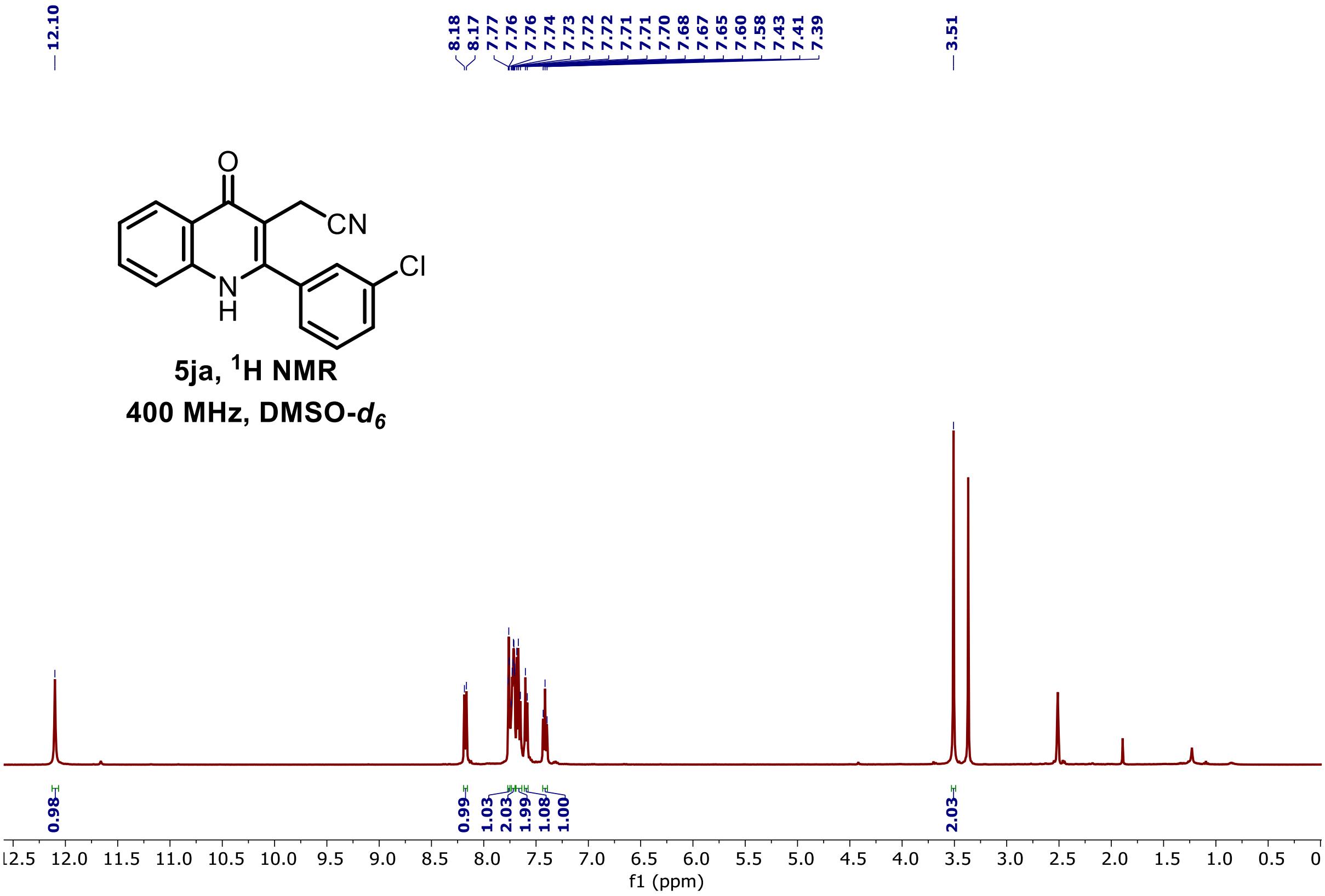
5ja, ¹H NMR
400 MHz, DMSO-d6
12.10
8.18
8.17
7.77
7.76
7.76
7.74
7.73
7.72
7.72
7.71
7.71
7.70
7.68
7.67
7.65
7.60
7.58
7.43
7.41
7.39
3.51
0.98
0.99
1.03
2.03
1.99
1.08
1.00
2.03
f1 (ppm)

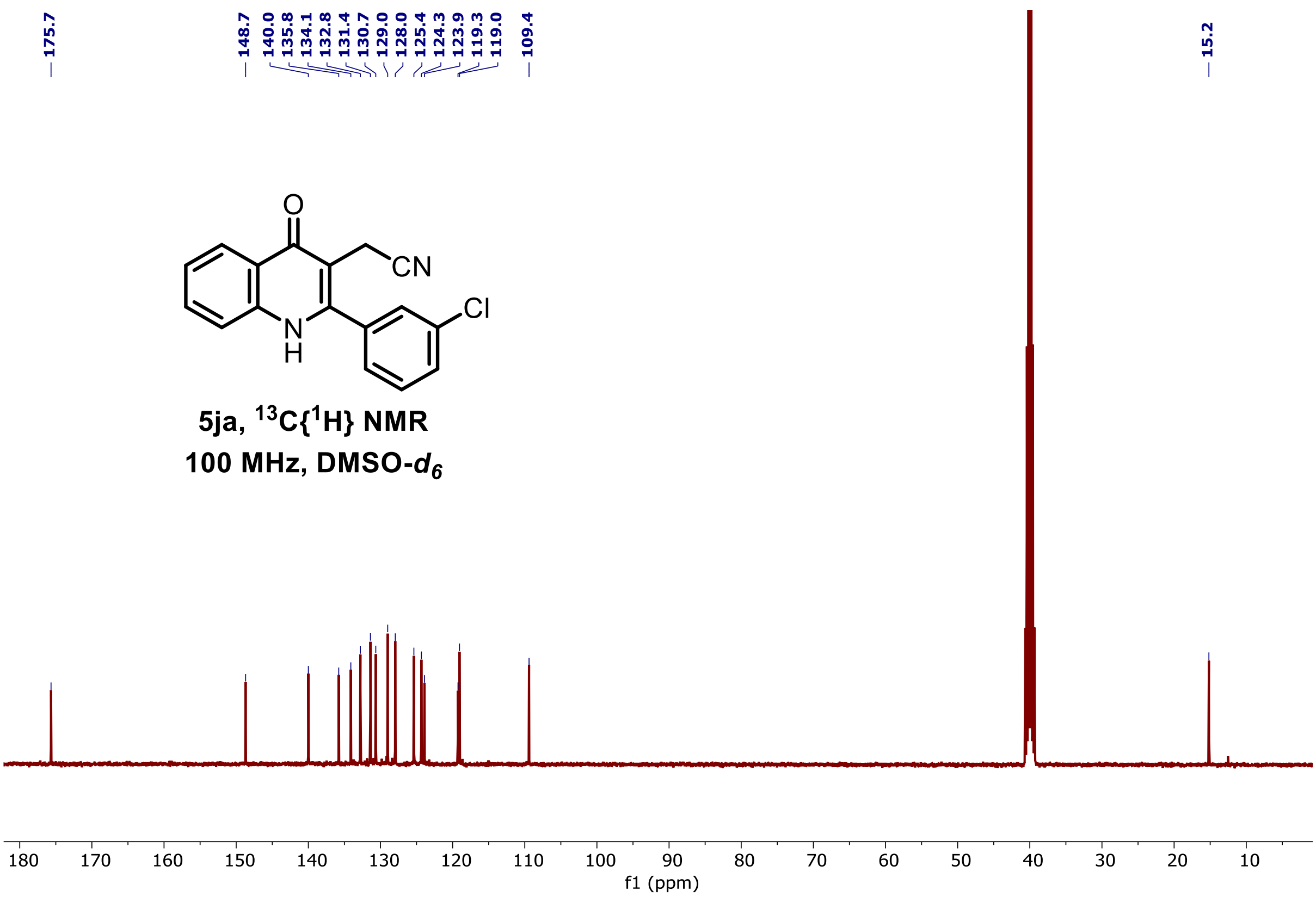
5ja, ¹³C{¹H} NMR
100 MHz, DMSO-d6
175.7
148.7
140.0
135.8
134.1
132.8
131.4
130.7
129.0
128.0
125.4
124.3
123.9
119.3
119.0
109.4
15.2
f1 (ppm)

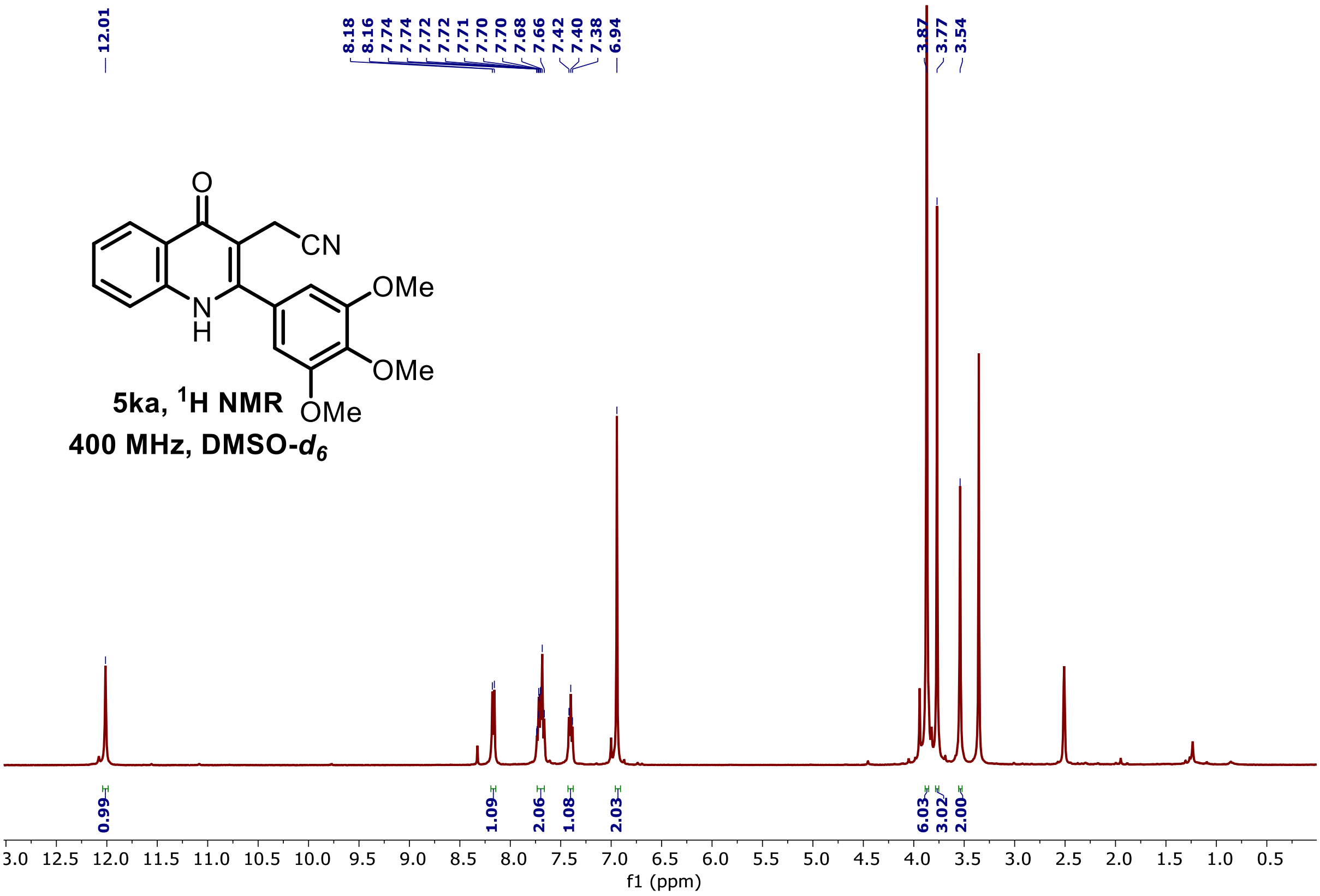
12.01
8.18
8.16
7.74
7.74
7.72
7.72
7.71
7.70
7.70
7.68
7.66
7.42
7.40
7.38
6.94
3.87
3.77
3.54
O
CN
OMe
OMe
OMe
N
H
5ka, 1H NMR
400 MHz, DMSO-d6
0.99
1.09
2.06
1.08
2.03
6.03
3.02
2.00
f1 (ppm)

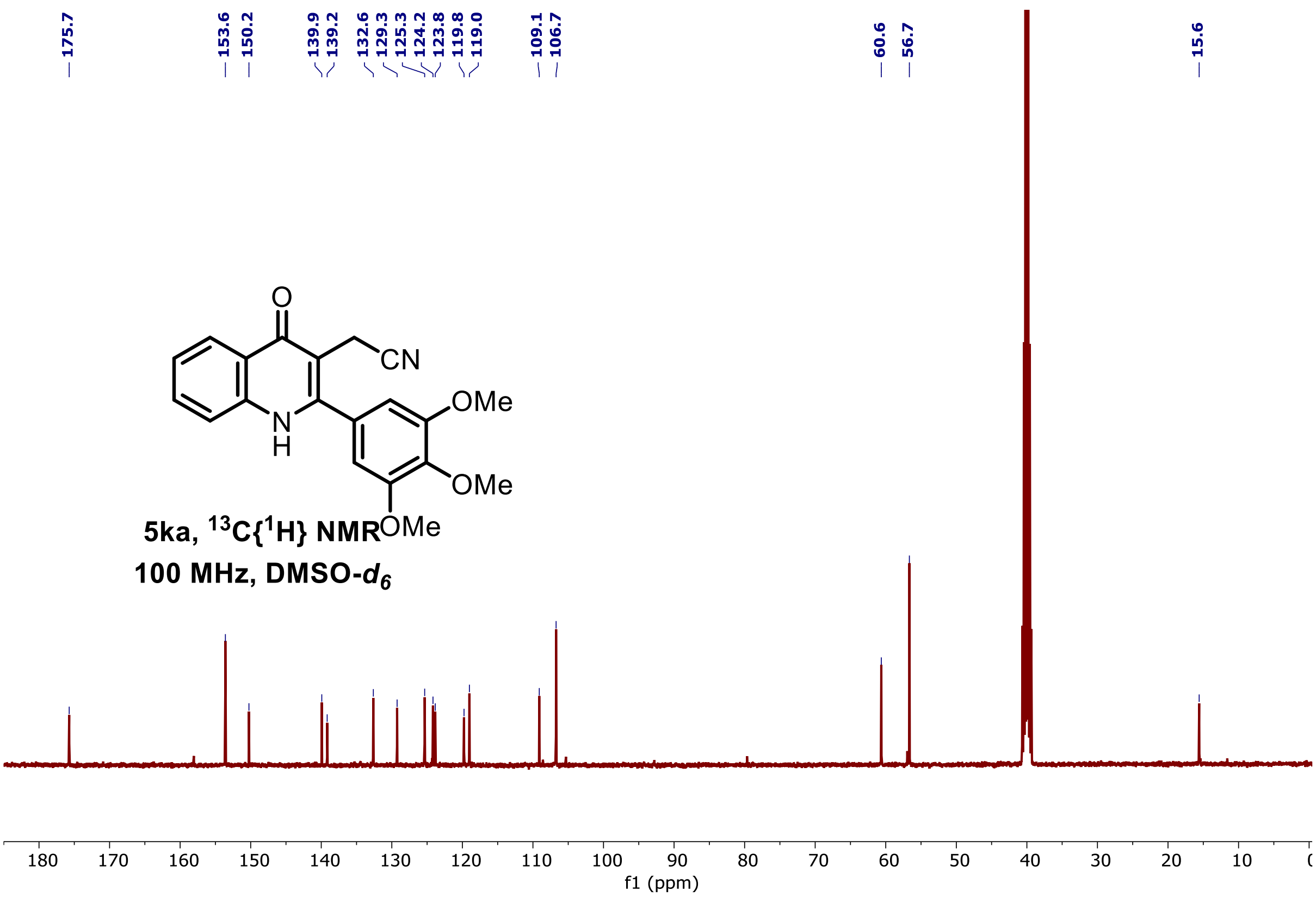
175.7
153.6
150.2
139.9
139.2
132.6
129.3
125.3
124.2
123.8
119.8
119.0
109.1
106.7
60.6
56.7
15.6
O
CN
OMe
OMe
OMe
N
H
5ka, 13C{1H} NMR
100 MHz, DMSO-d6
f1 (ppm)

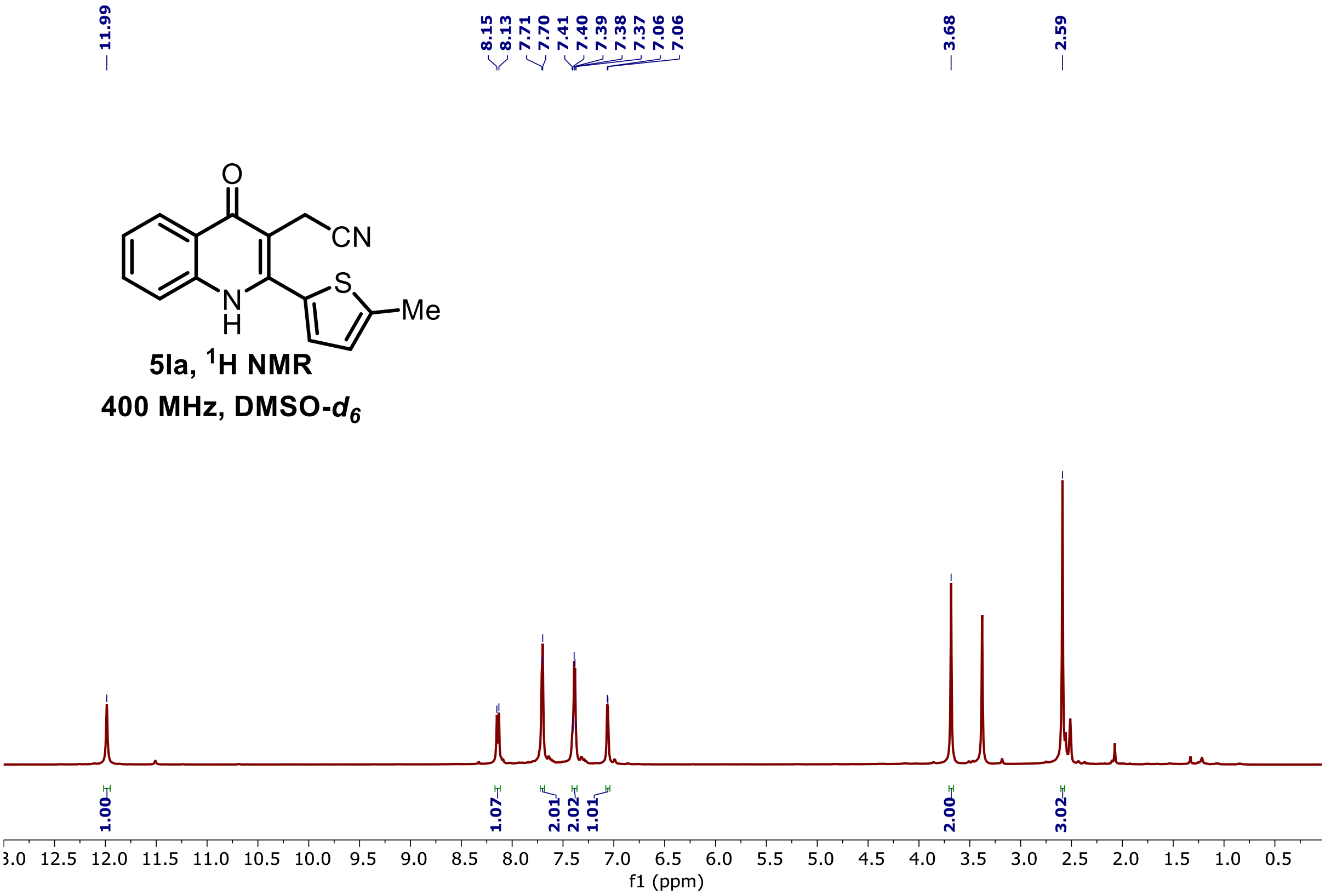
11.99
8.15 8.13 7.71 7.70 7.41 7.40 7.39 7.38 7.37 7.06 7.06
3.68
2.59
5la, ¹H NMR
400 MHz, DMSO-d6
1.00
1.07
2.01
2.02
1.01
2.00
3.02
f1 (ppm)

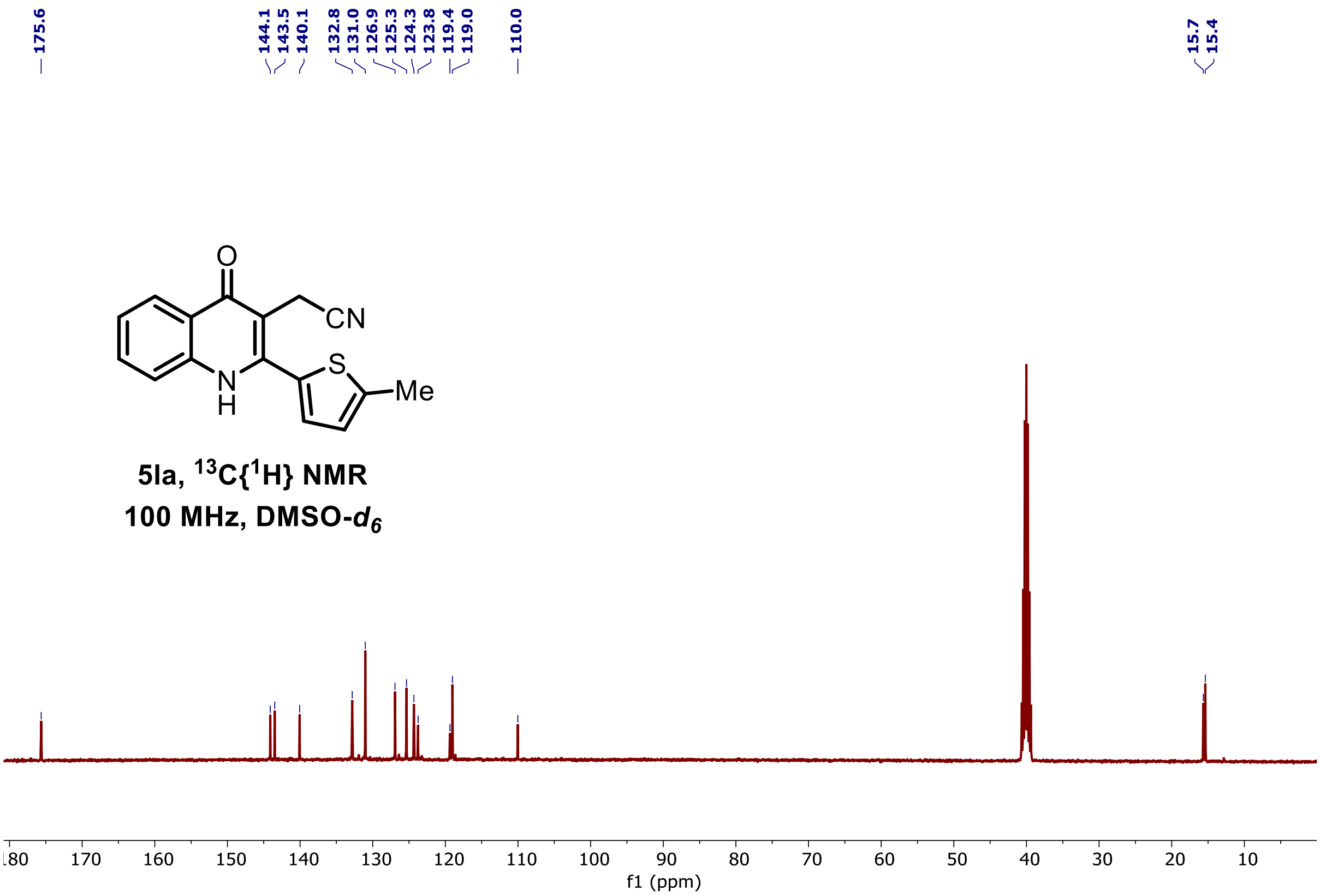
175.6
144.1 143.5 140.1 132.8 131.0 126.9 125.3 124.3 123.8 119.4 119.0
110.0
15.7 15.4
5la, ¹³C{¹H} NMR
100 MHz, DMSO-d6
f1 (ppm)

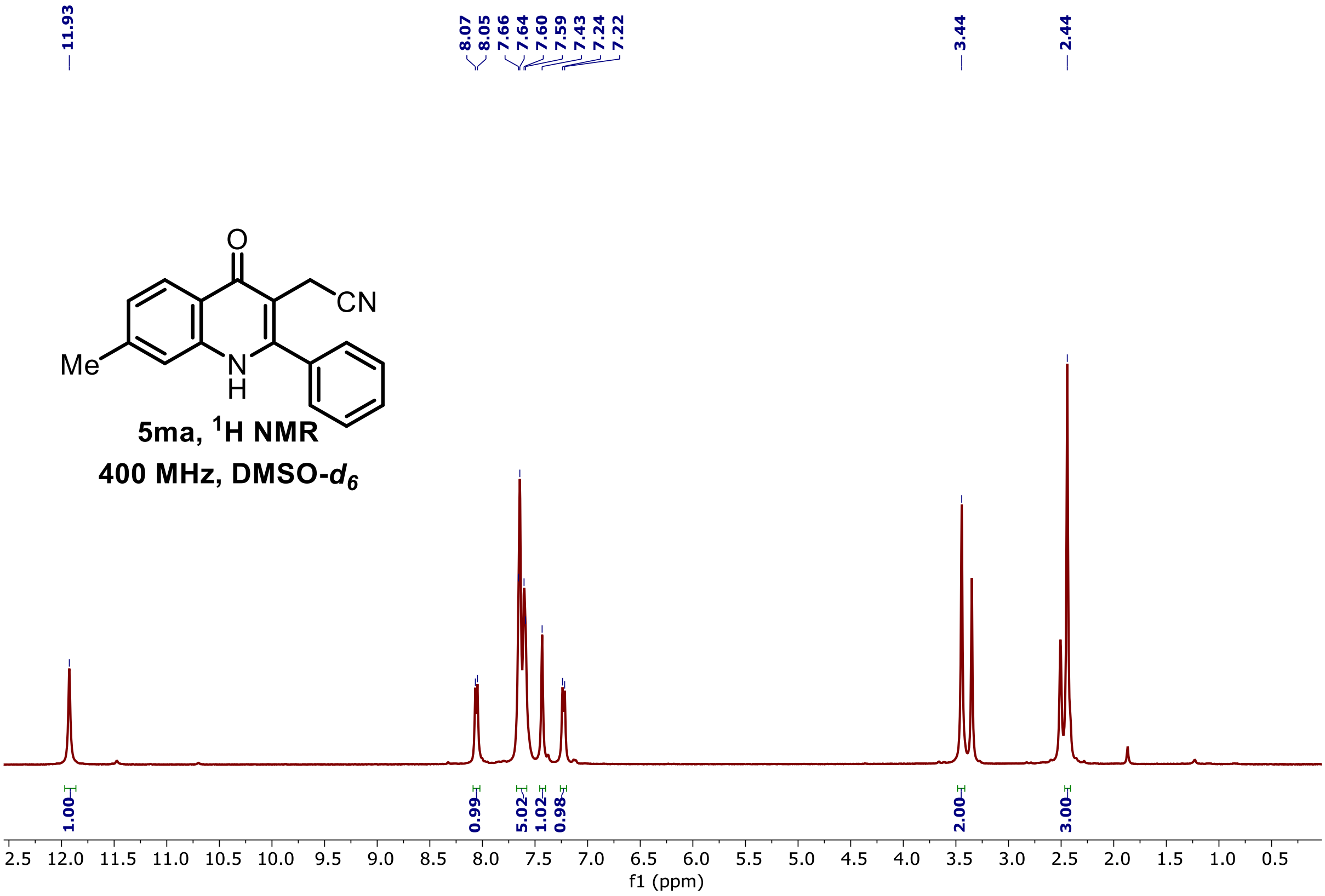
11.93
8.07
8.05
7.66
7.64
7.60
7.59
7.43
7.24
7.22
3.44
2.44
O
CN
Me
N
H
5ma, 1H NMR
400 MHz, DMSO-d6
1.00
0.99
5.02
1.02
0.98
2.00
3.00
f1 (ppm)

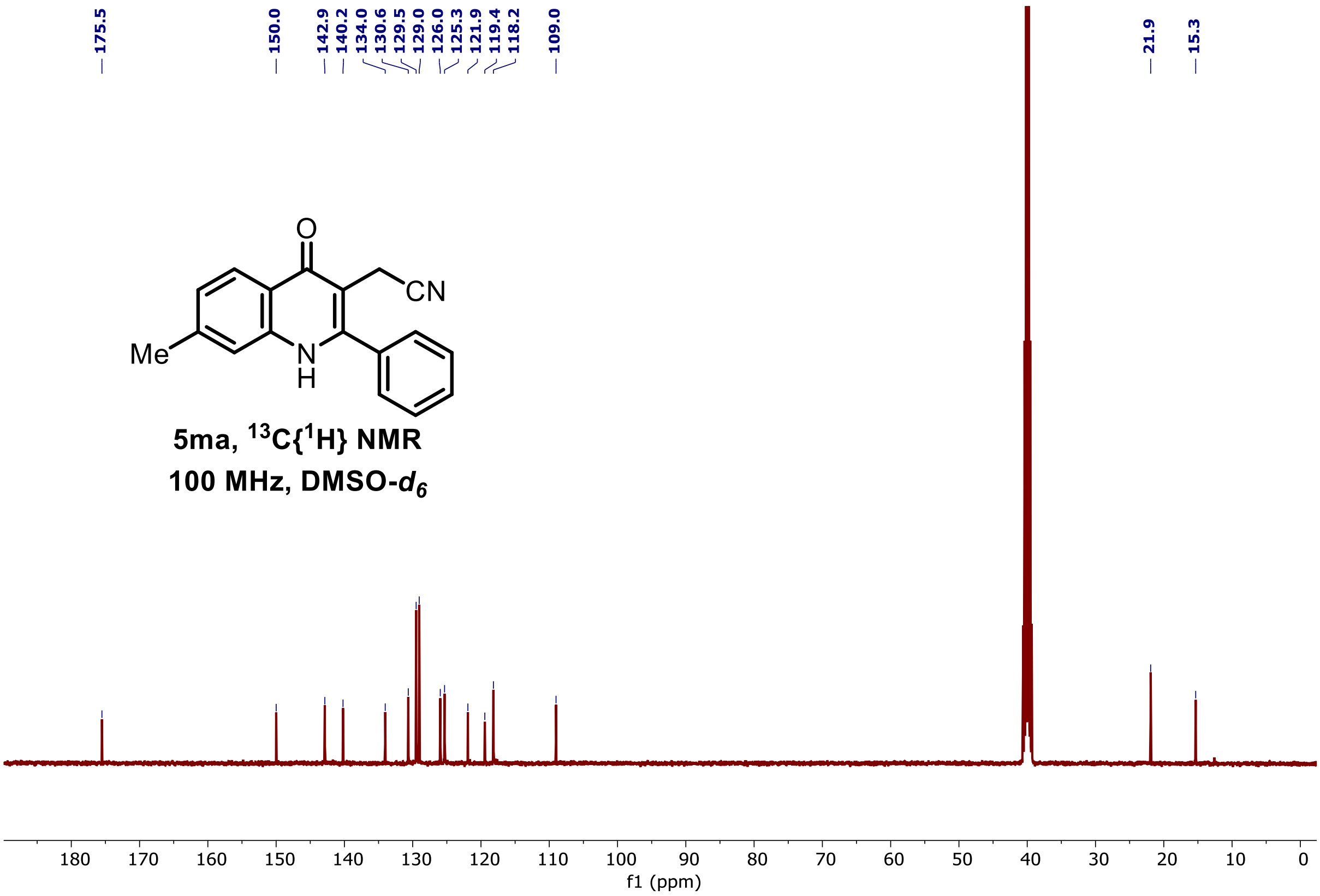
175.5
150.0
142.9
140.2
134.0
130.6
129.5
129.0
126.0
125.3
121.9
119.4
118.2
109.0
21.9
15.3
O
CN
Me
N
H
5ma, 13C{1H} NMR
100 MHz, DMSO-d6
f1 (ppm)

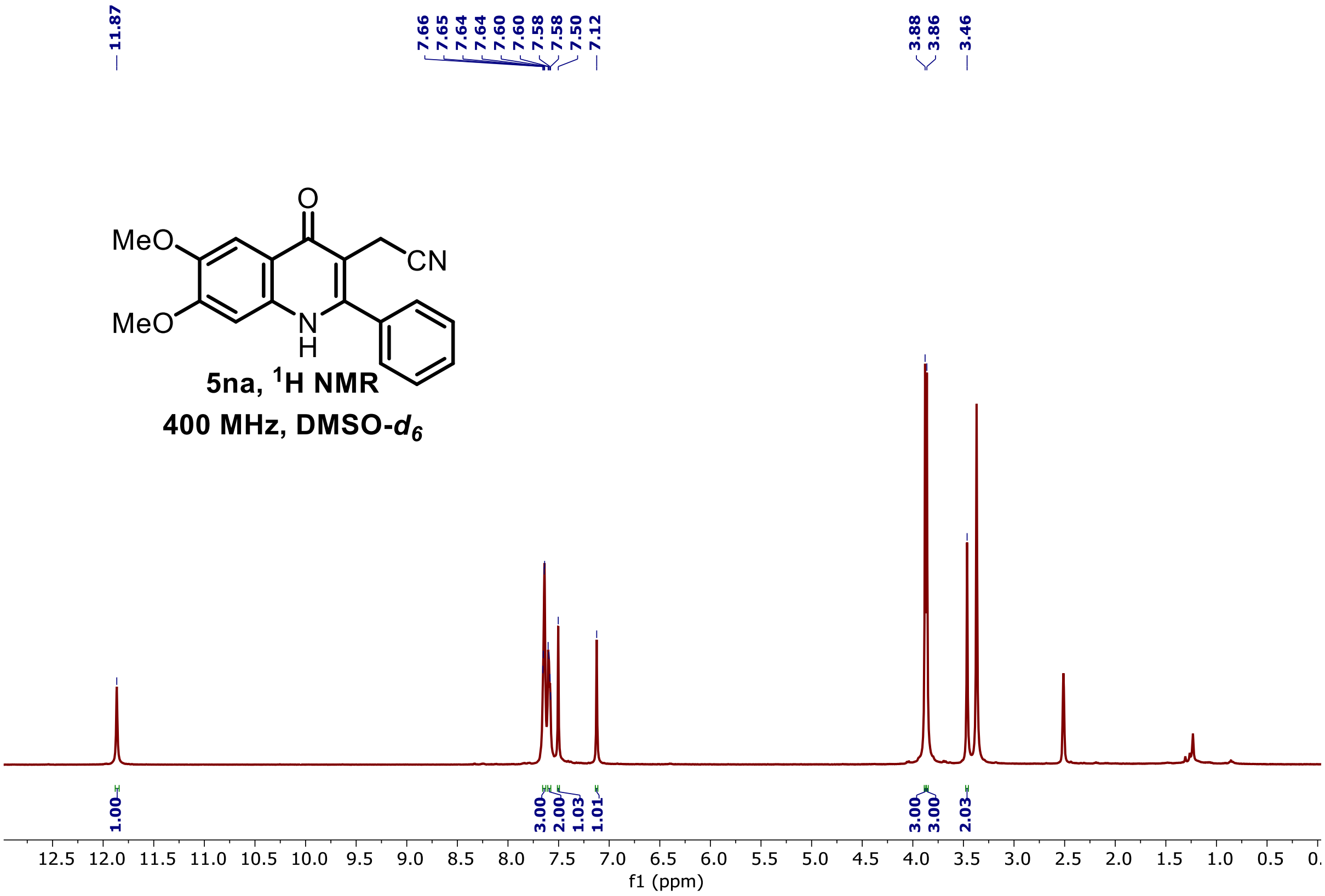
11.87
7.66
7.65
7.64
7.64
7.60
7.60
7.58
7.58
7.50
7.12
3.88
3.86
3.46
MeO
MeO
O
CN
N
H
5na, 1H NMR
400 MHz, DMSO-d6
1.00
3.00
2.00
1.03
1.01
3.00
3.00
2.03
12.5 12.0 11.5 11.0 10.5 10.0 9.5 9.0 8.5 8.0 7.5 7.0 6.5 6.0 5.5 5.0 4.5 4.0 3.5 3.0 2.5 2.0 1.5 1.0 0.5 0.
f1 (ppm)

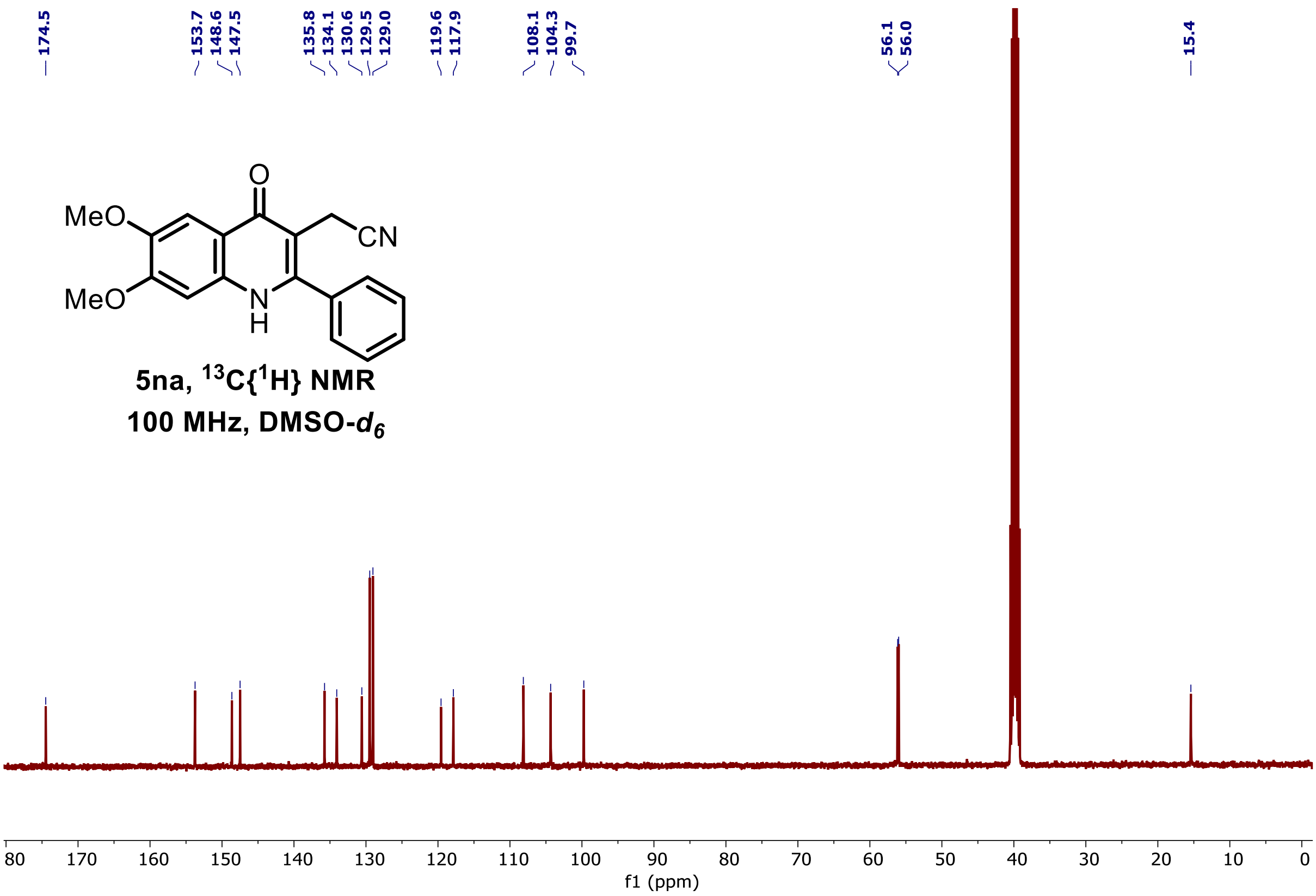
174.5
153.7
148.6
147.5
135.8
134.1
130.6
129.5
129.0
119.6
117.9
108.1
104.3
99.7
56.1
56.0
15.4
MeO
MeO
O
CN
N
H
5na, 13C{1H} NMR
100 MHz, DMSO-d6
80 170 160 150 140 130 120 110 100 90 80 70 60 50 40 30 20 10 0
f1 (ppm)

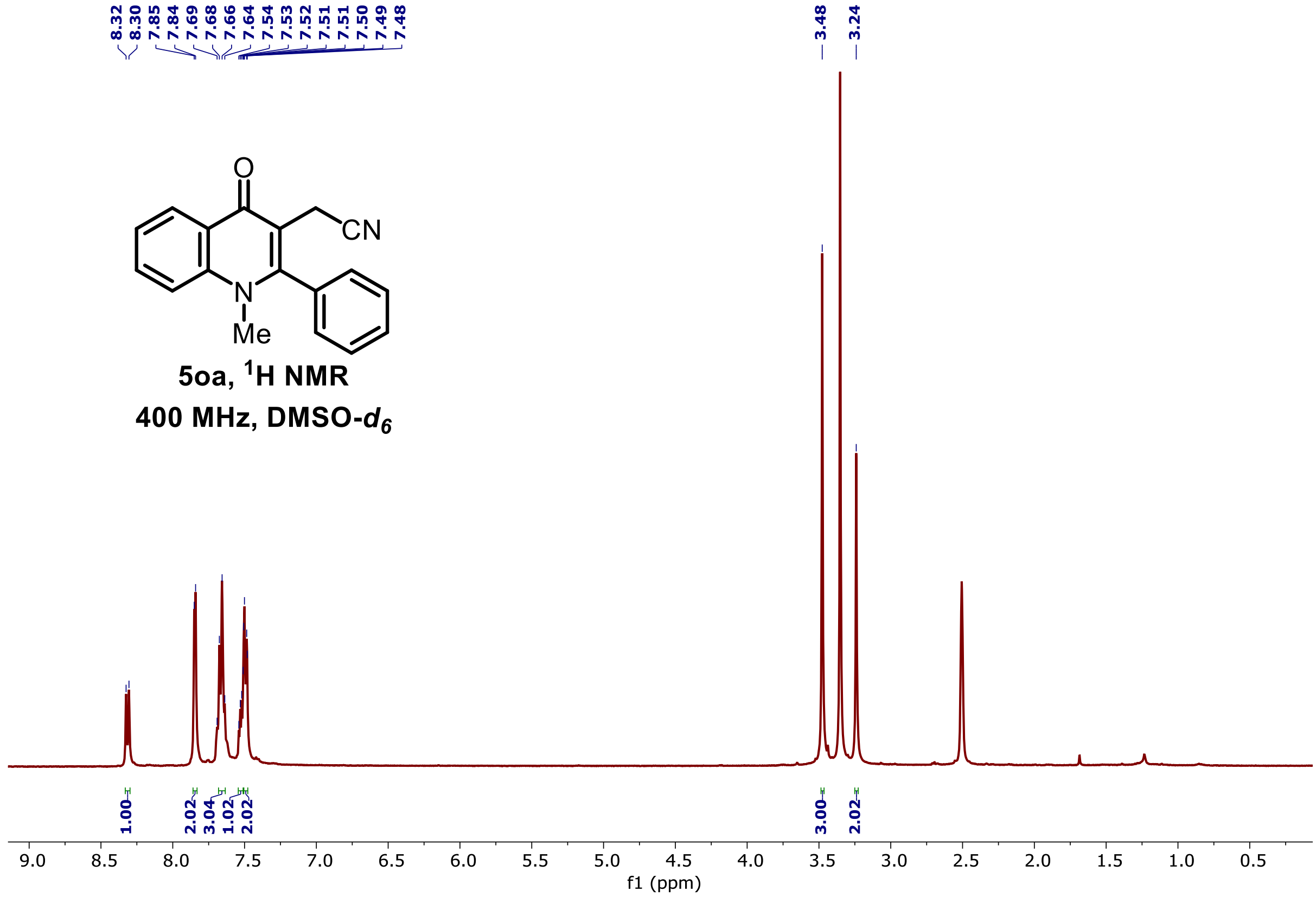

5oa, ^{1}H NMR

400 MHz, DMSO-*d*$_6$

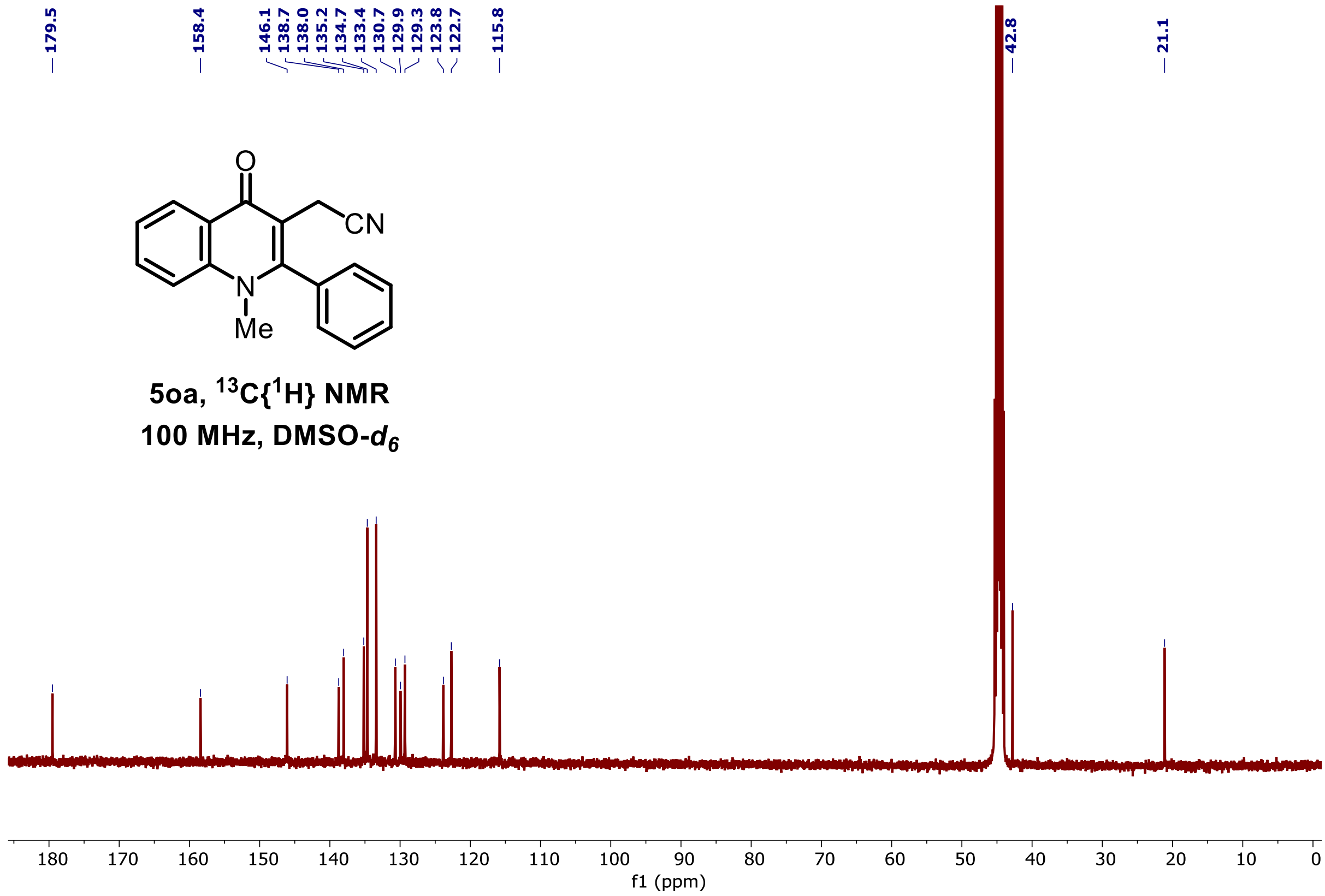

5oa, ^{13}C{^{1}H} NMR

100 MHz, DMSO-*d*$_6$

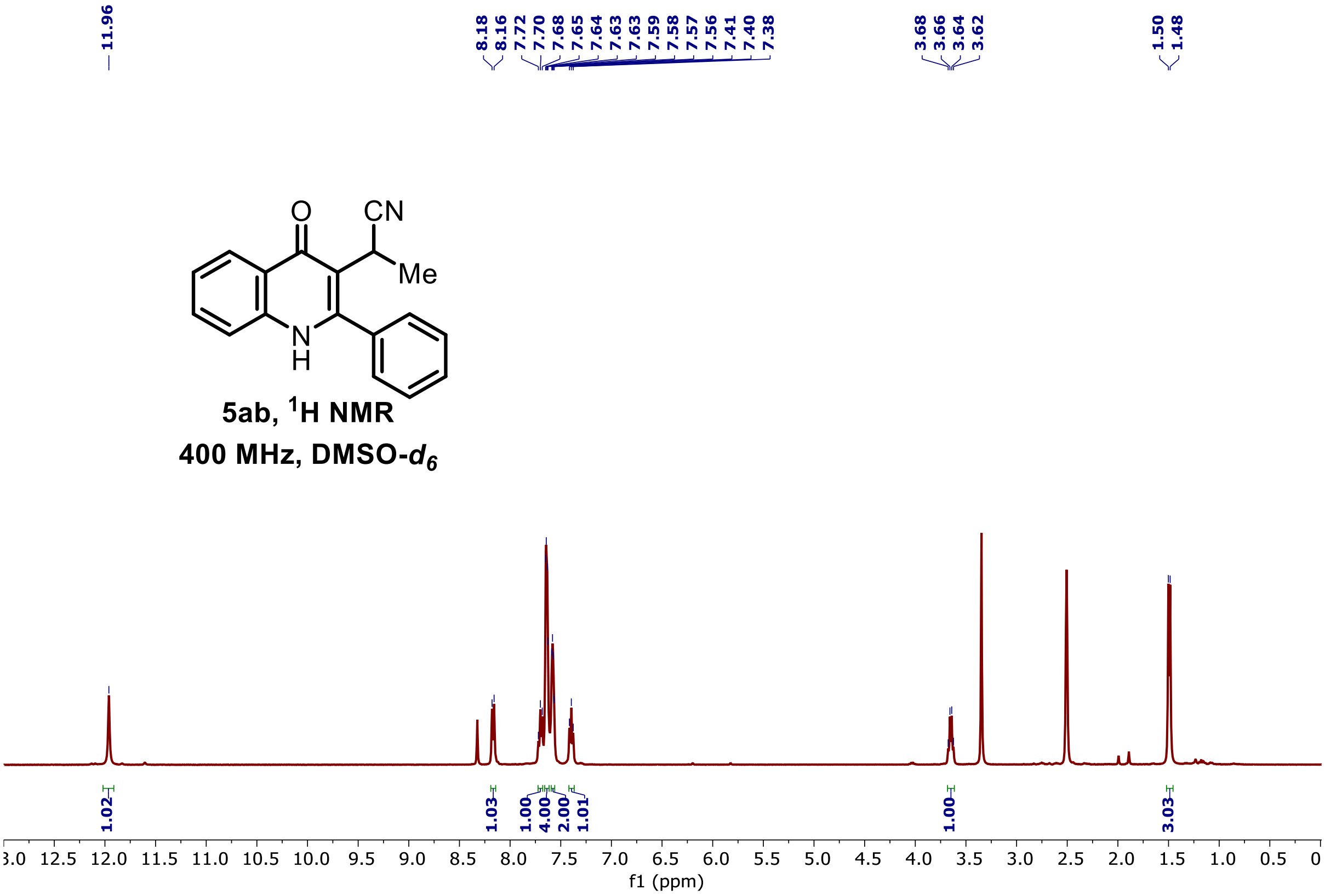

5ab, 1H NMR
400 MHz, DMSO-d6
f1 (ppm)

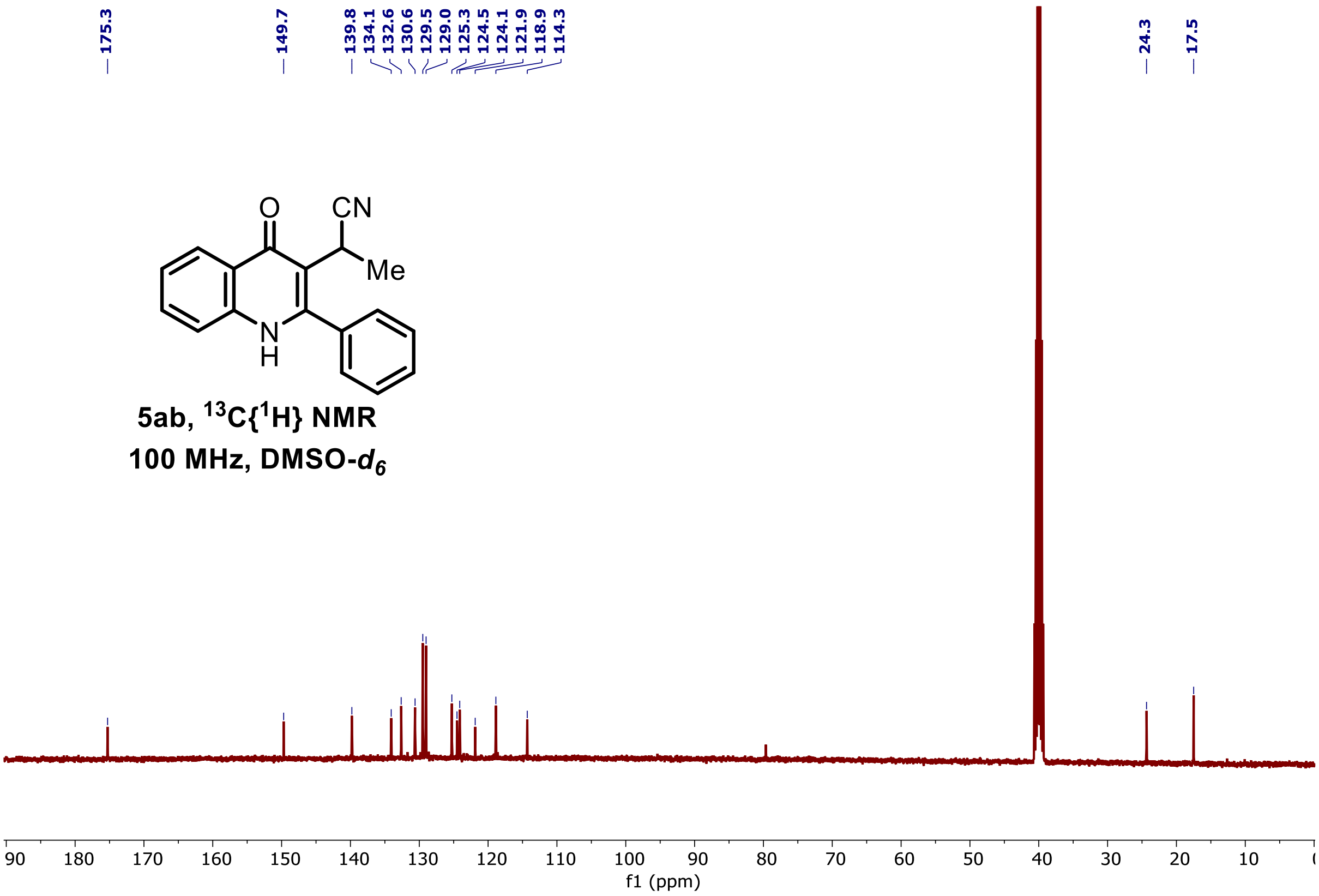

5ab, 13C{1H} NMR
100 MHz, DMSO-d6
f1 (ppm)

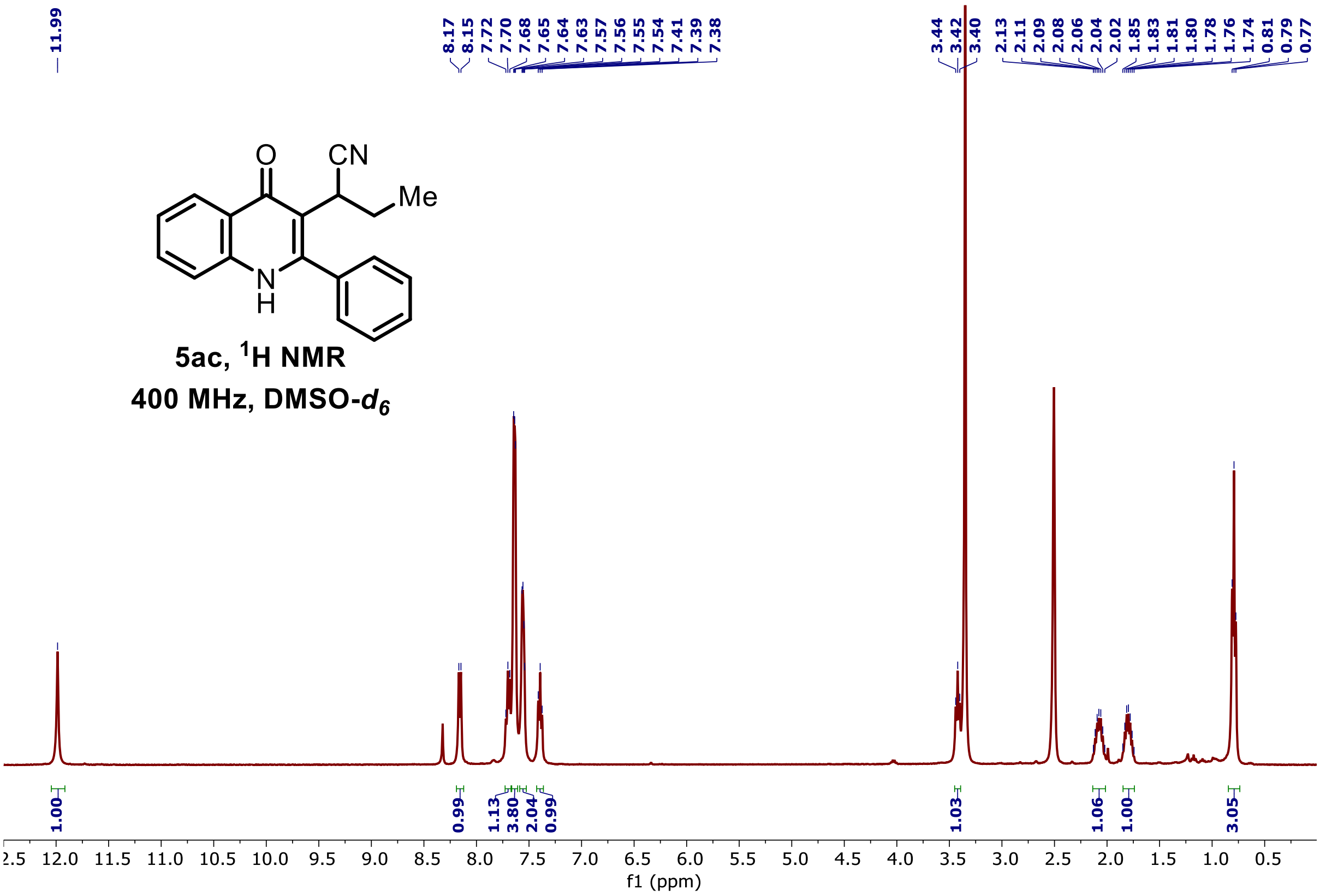

5ac, ^{1}H NMR
400 MHz, DMSO-d_6

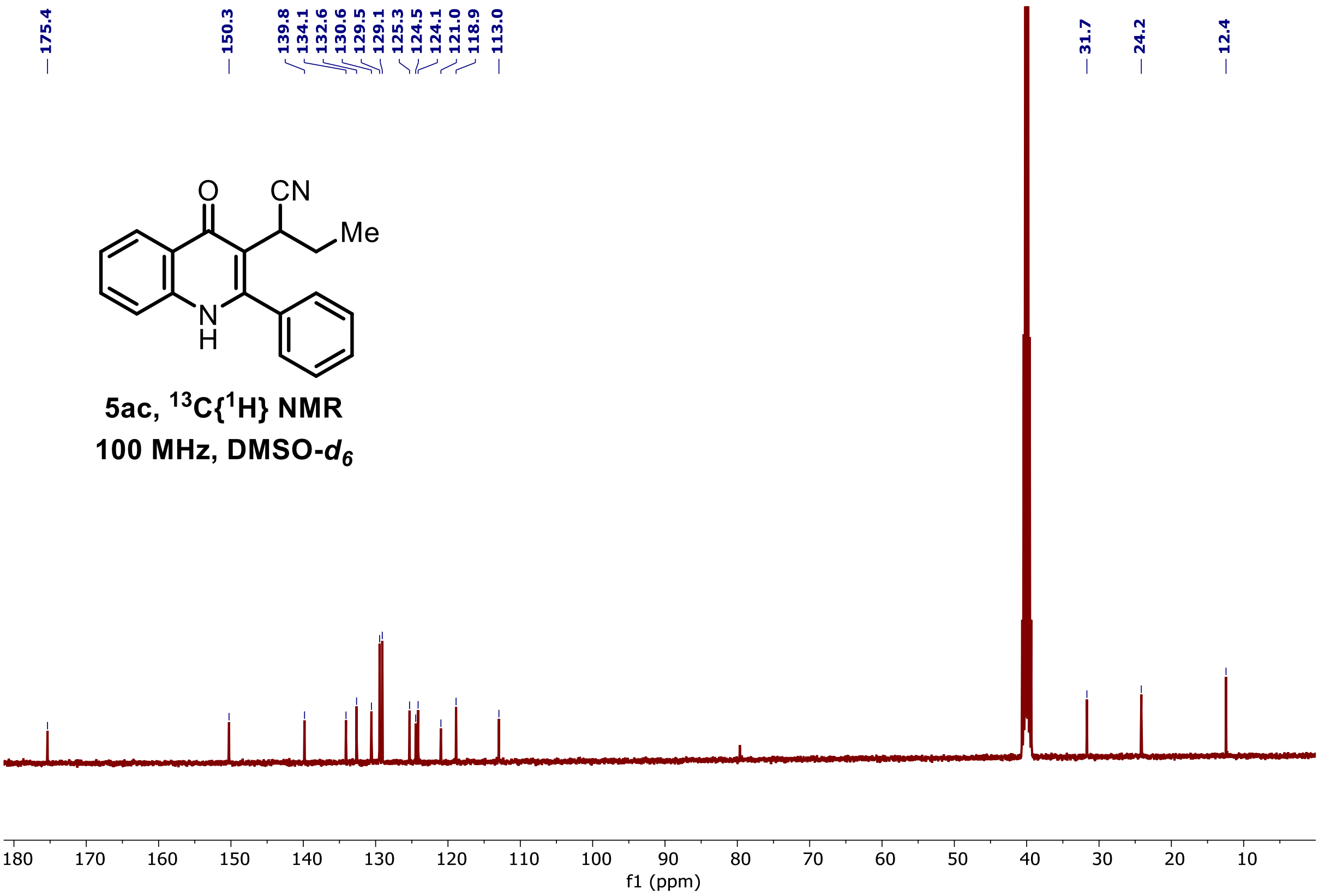

5ac, ^{13}C{^{1}H} NMR
100 MHz, DMSO-d_6

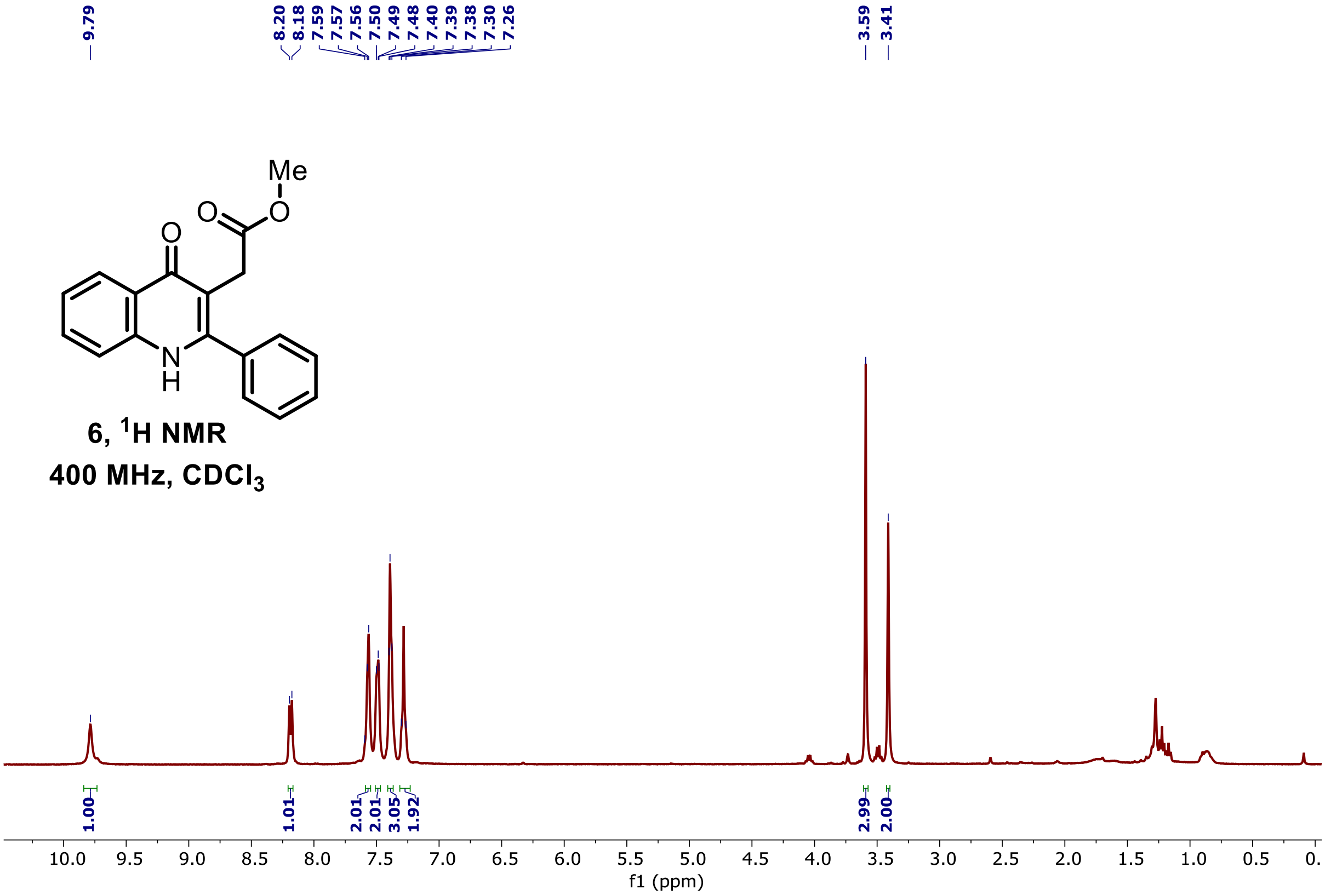
9.79
8.20
8.18
7.59
7.57
7.56
7.50
7.49
7.48
7.40
7.39
7.38
7.30
7.26
3.59
3.41
Me
O
O
O
N
H
6, 1H NMR
400 MHz, CDCl3
1.00
1.01
2.01
2.01
3.05
1.92
2.99
2.00
10.0 9.5 9.0 8.5 8.0 7.5 7.0 6.5 6.0 5.5 5.0 4.5 4.0 3.5 3.0 2.5 2.0 1.5 1.0 0.5 0.
f1 (ppm)

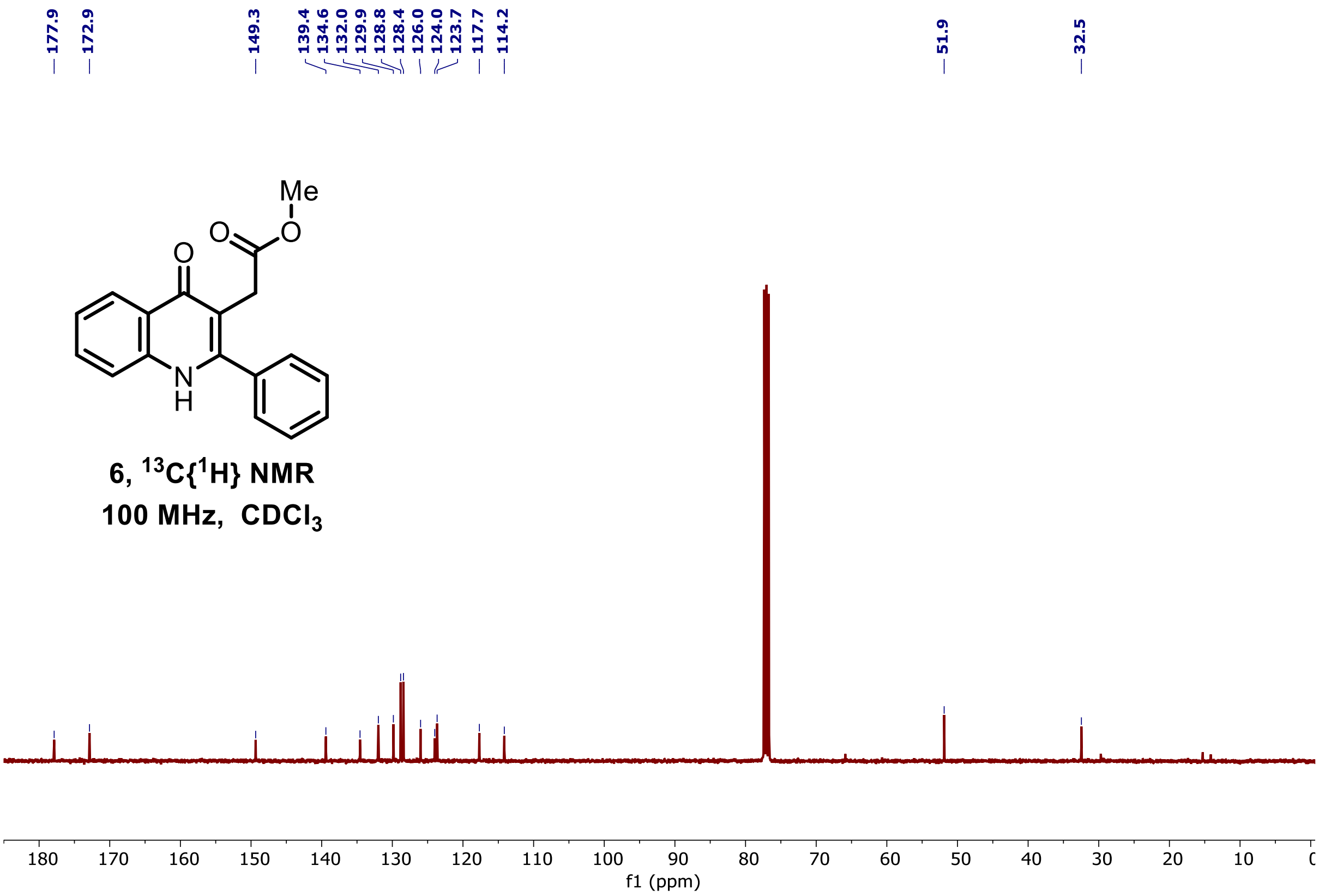
177.9
172.9
149.3
139.4
134.6
132.0
129.9
128.8
128.4
126.0
124.0
123.7
117.7
114.2
51.9
32.5
Me
O
O
O
N
H
6, 13C{1H} NMR
100 MHz, CDCl3
180 170 160 150 140 130 120 110 100 90 80 70 60 50 40 30 20 10 0
f1 (ppm)

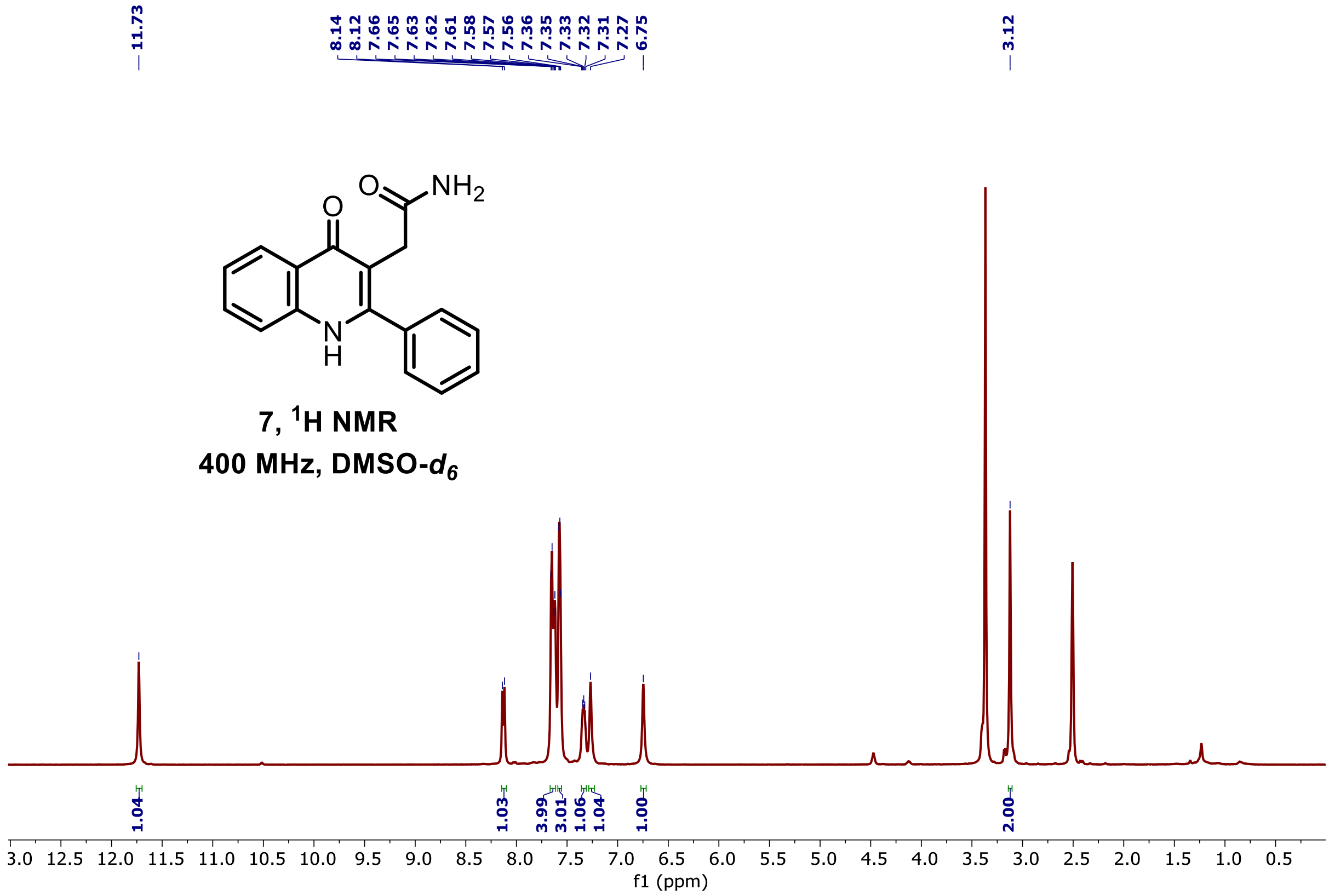
11.73
8.14
8.12
7.66
7.65
7.63
7.62
7.61
7.58
7.57
7.56
7.36
7.35
7.33
7.32
7.31
7.27
6.75
3.12
7, ¹H NMR
400 MHz, DMSO-d6
1.04
1.03
3.99
3.01
1.06
1.04
1.00
2.00
f1 (ppm)

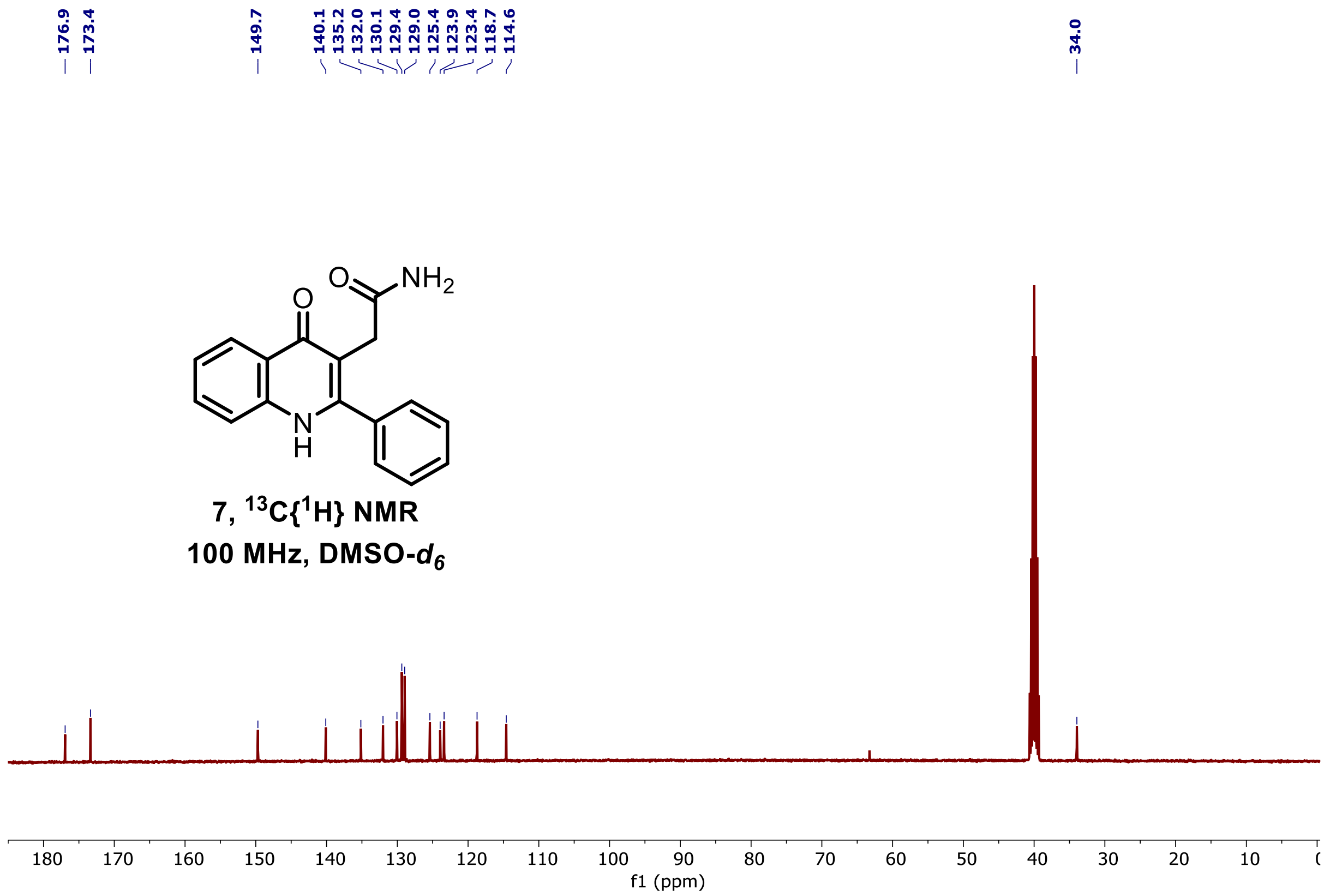
176.9
173.4
149.7
140.1
135.2
132.0
130.1
129.4
129.0
125.4
123.9
123.4
118.7
114.6
34.0
7, ¹³C{¹H} NMR
100 MHz, DMSO-d6
f1 (ppm)

5. Copies of HRMS DATA of adducts 8-10

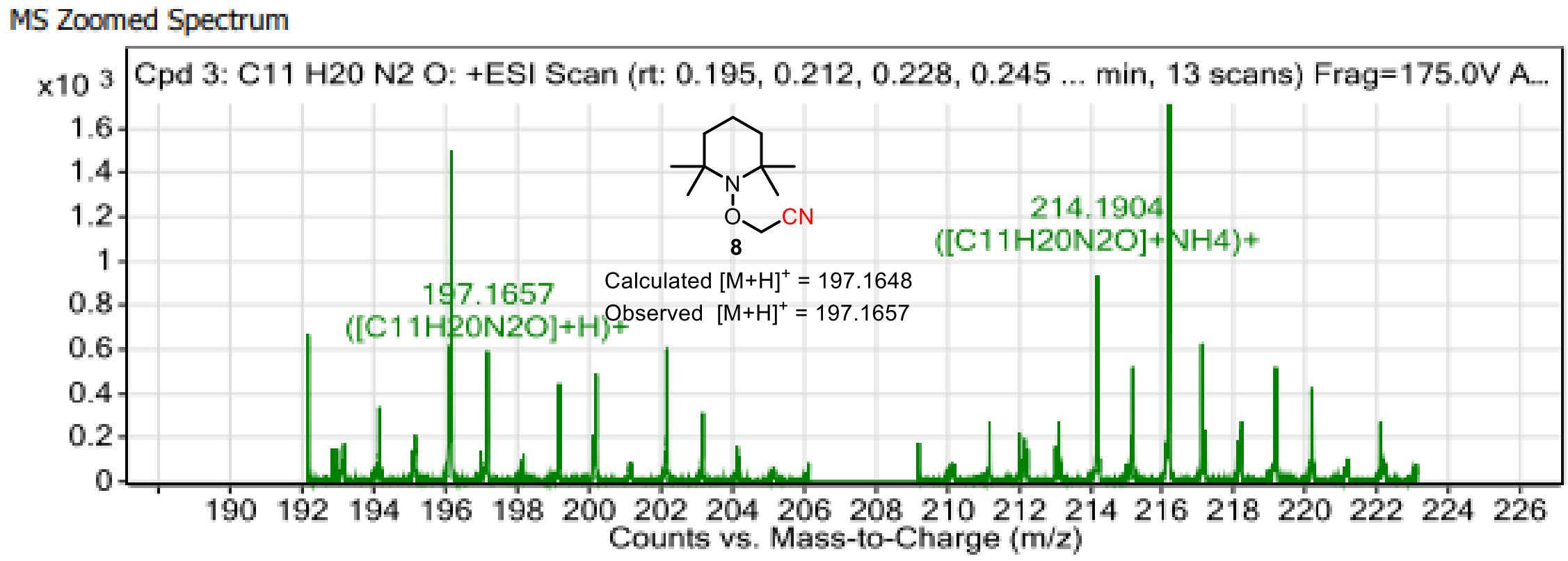

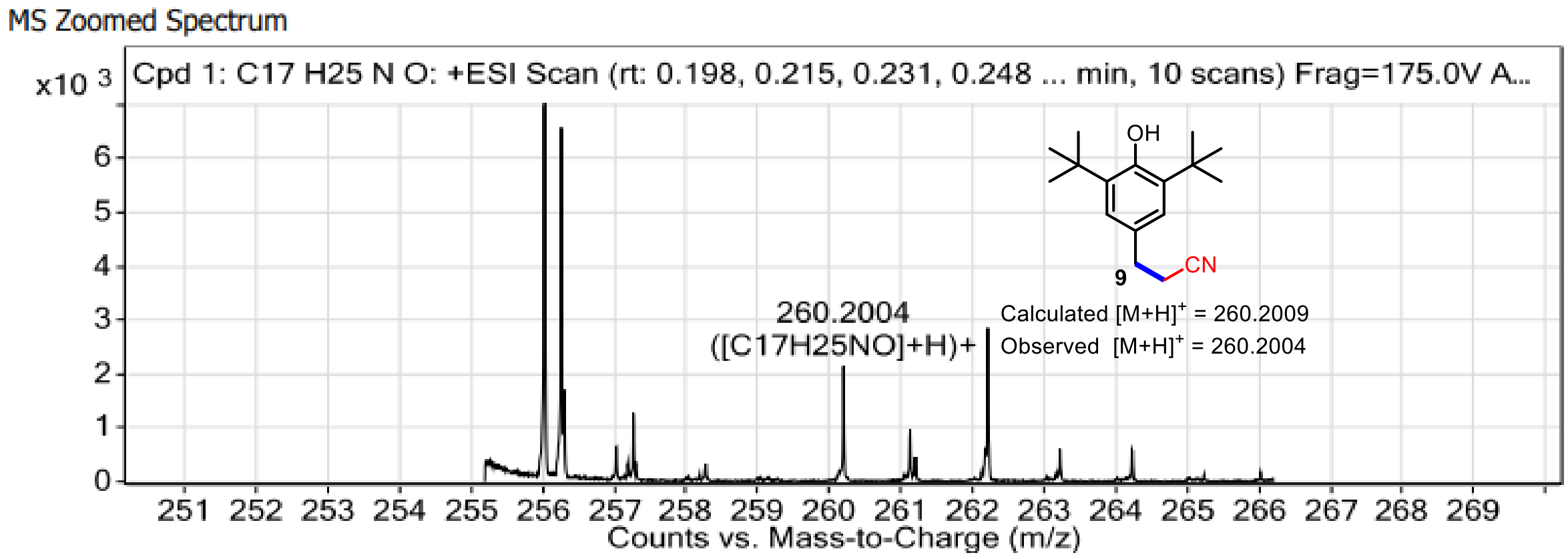

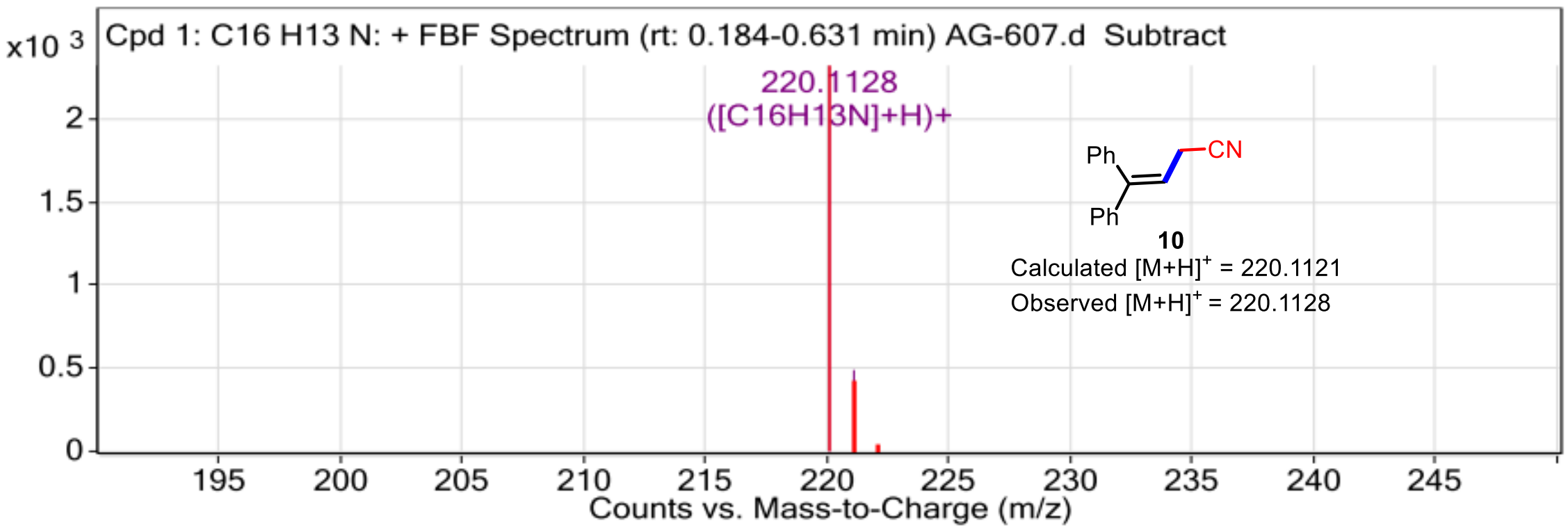

6. Kinetic isotope effect experiment

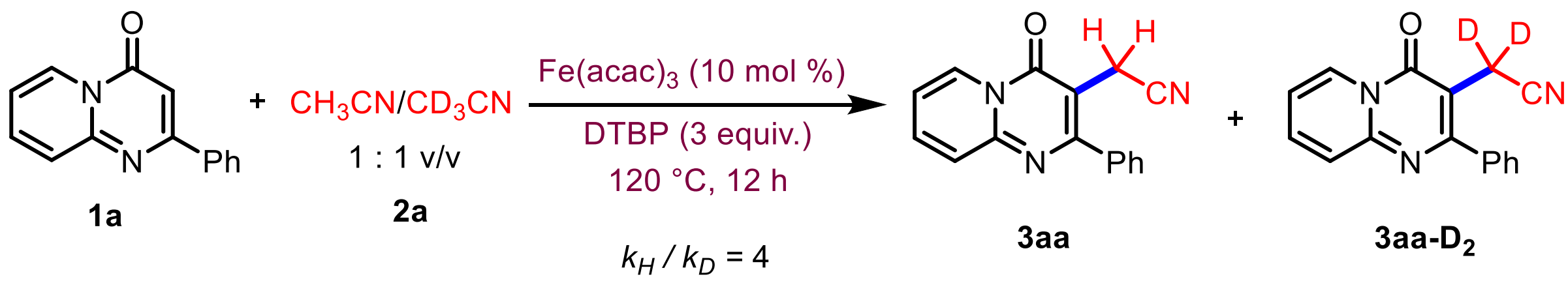

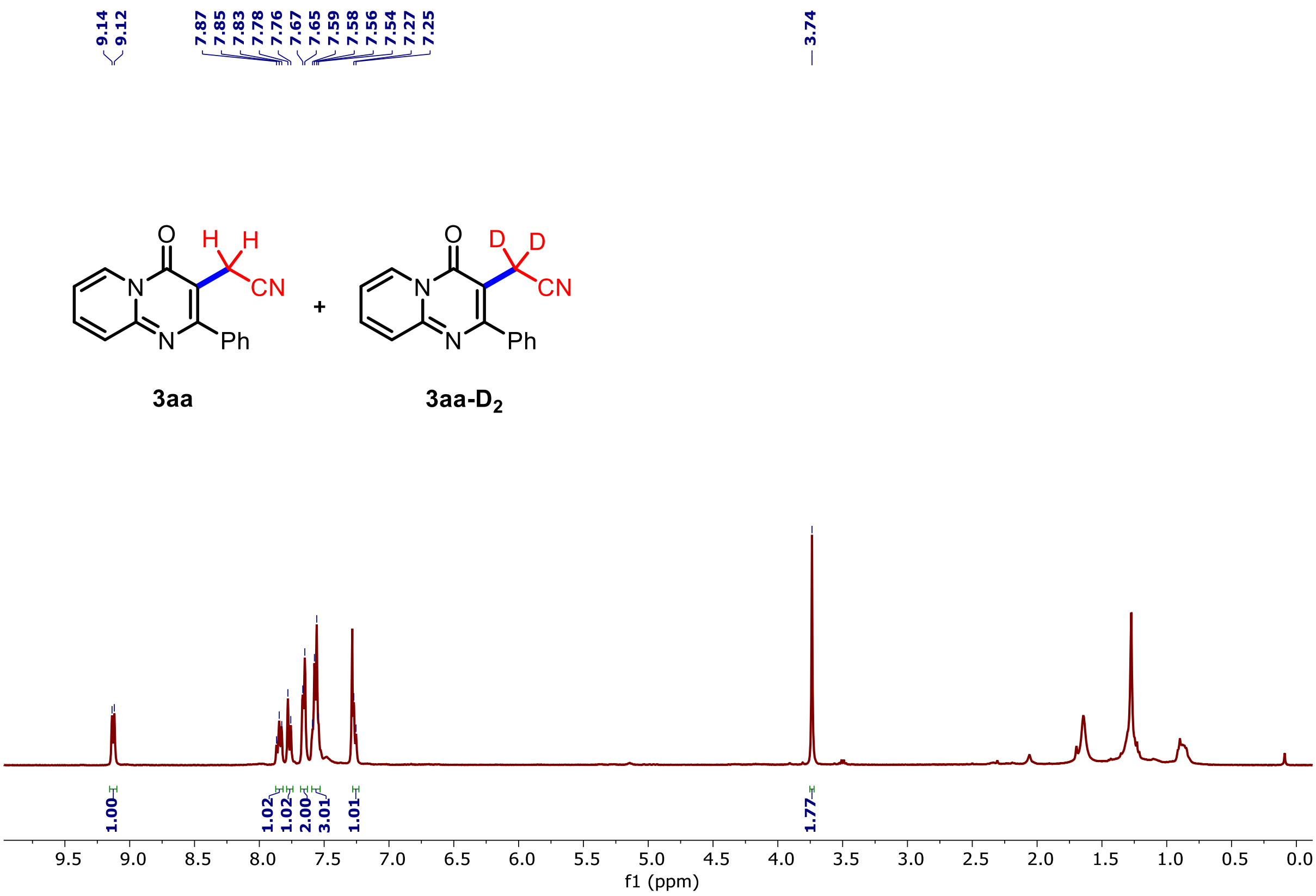

7. Single crystal X-ray diffraction data of 3ma and 5ba

Experimentation: A suitable crystal was selected and mounted on a XtaLAB Pro II AFC12 (RINC): Kappa dual home/near diffractometer. The crystal was kept at 100 K during data collection. Using Olex2[3]. The structure was solved with the ShelXT[4] structure solution program using Intrinsic phasing and refined with the ShelXL[5] refinement package using least squares minimisation.

Single crystals of **3la** [$C_{17}H_{13}N_3O$] were grown from the slow evaporation of chloroform: hexane solution (CCDC 2543349).

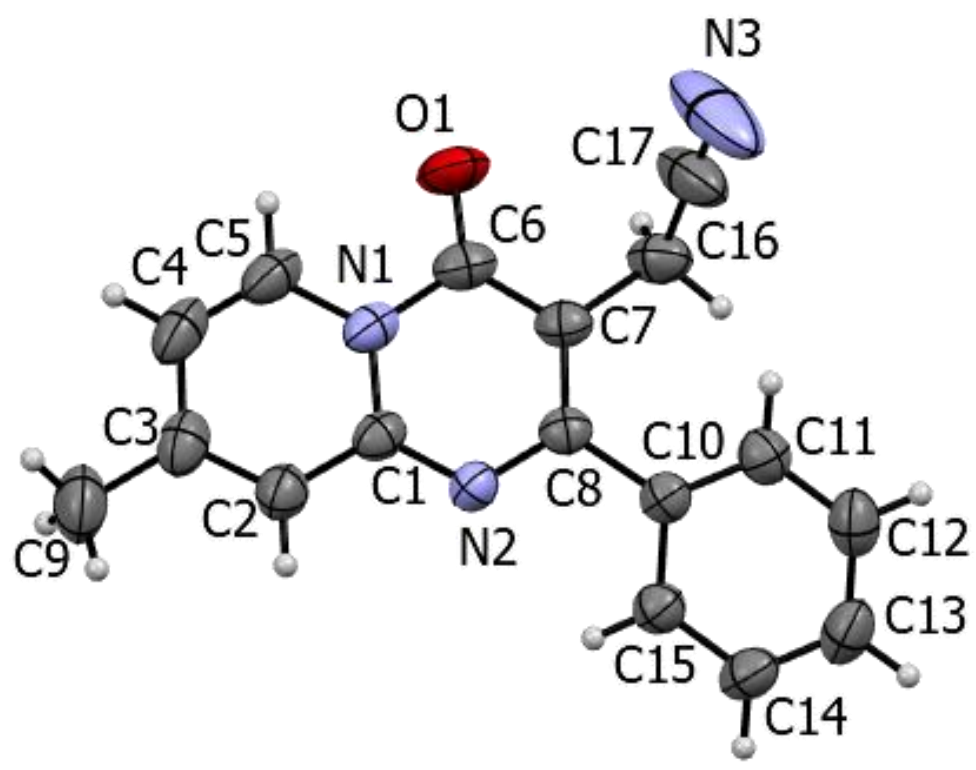

Figure S1. ORTEP diagram of compound **3ma**. Thermal ellipsoids are drawn at 50% probability level.

Table S1 Crystal data and structure refinement for 3ma.

Identification code	Exp-3la
Empirical formula	$C_{17}H_{13}N_3O$
Formula weight	275.30
Temperature/K	298
Crystal system	monoclinic
Space group	$P2_1/n$
a/Å	13.3949(4)
b/Å	7.1532(2)
c/Å	14.5909(4)
α/°	90
β/°	99.755(3)
γ/°	90
Volume/Å^3	1377.83(7)
Z	4
ρ_{calc}g/cm^3	1.327
μ/mm^{-1}	0.086
F(000)	576.0
Crystal size/mm^3	0.28 × 0.26 × 0.05
Radiation	Mo Kα (λ = 0.71073)
2Θ range for data collection/°	3.818 to 52.452
Index ranges	$-16 \le h \le 15$, $-8 \le k \le 8$, $-16 \le l \le 17$
Reflections collected	17544
Independent reflections	2623 [R_{int} = 0.0247, R_{sigma} = 0.0197]
Data/restraints/parameters	2623/0/191
Goodness-of-fit on F^2	1.053
Final R indexes [I>=2σ (I)]	R_1 = 0.0409, wR_2 = 0.1102
Final R indexes [all data]	R_1 = 0.0537, wR_2 = 0.1196
Largest diff. peak/hole / e Å^{-3}	0.17/-0.15

Single crystals of **5ba** [$C_{18}H_{14}N_2O$] were grown from the slow evaporation of chloroform: hexane solution (CCDC 2543354).

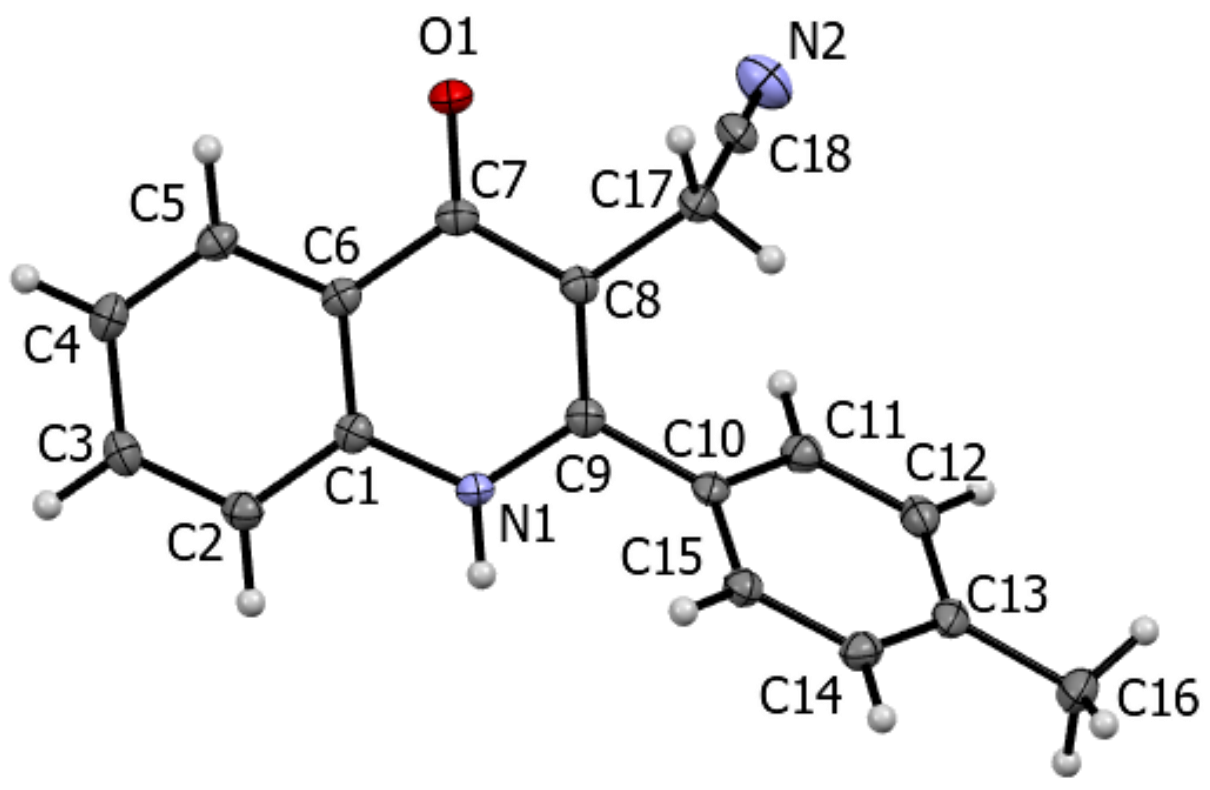

Figure S2. ORTEP diagram of compound **5ba**. Thermal ellipsoids are drawn at 50% probability level.

Table S2 Crystal data and structure refinement for 5ba.

Identification code	Exp-5ba
Empirical formula	$C_{18}H_{14}N_2O$
Formula weight	274.31
Temperature/K	100
Crystal system	triclinic
Space group	P-1
a/Å	6.9451(3)
b/Å	7.4036(4)
c/Å	14.3163(6)
α/°	80.658(4)
β/°	76.105(4)
γ/°	70.890(4)
Volume/Å^3	672.39(6)
Z	2
ρ_{calc}g/cm^3	1.355
μ/mm^{-1}	0.085
F(000)	288.0

Crystal size/mm^3	0.23 × 0.18 × 0.16
Radiation	Mo Kα (λ = 0.71073)
2Θ range for data collection/°	5.848 to 52.482
Index ranges	-8 ≤ h ≤ 8, -8 ≤ k ≤ 9, -17 ≤ l ≤ 17
Reflections collected	12778
Independent reflections	2541 [R_{int} = 0.0512, R_{sigma} = 0.0345]
Data/restraints/parameters	2541/0/191
Goodness-of-fit on F^2	1.074
Final R indexes [I>=2σ (I)]	R_1 = 0.0424, wR_2 = 0.1126
Final R indexes [all data]	R_1 = 0.0480, wR_2 = 0.1183
Largest diff. peak/hole / e $Å^{-3}$	0.20/-0.24

8. References:

[1] C. La Motta, S. Sartini, L. Mugnaini, F. Simorini, S. Taliani, S. Salerno, A. M. Marini, F. Da Settimo, A. Lavecchia, E. Novellino, M. Cantore, P. Failli, M. Ciuffi, *J. Med. Chem.* **2007**, *50*, 4917-4927.

[2] L. Dong, X. Wang, Y. Nie, S. Yu, H. Li, Q. Zhao, Z. Fan, Y. Wang, X. Tan, Z. Yu, *Eur. J. Org. Chem.* **2022**, *2022*, e202200842.

[3] O. V. Dolomanov, L. J. Bourhis, R. J. Gildea, J. A. K. Howard, H. Puschmann, *J. Appl. Cryst.* **2009**, *42*, 339-341.

[4] G. Sheldrick, *Acta Cryst. A* **2015**, *71*, 3-8.

[5] G. Sheldrick, *Acta Cryst. C* **2015**, *71*, 3-8.
